# N-terminal Proteomics Assisted Profiling of the Unexplored Translation Initiation Landscape in Arabidopsis thaliana * [Fn FN2]

**DOI:** 10.1074/mcp.M116.066662

**Published:** 2017-04-21

**Authors:** Patrick Willems, Elvis Ndah, Veronique Jonckheere, Simon Stael, Adriaan Sticker, Lennart Martens, Frank Van Breusegem, Kris Gevaert, Petra Van Damme

**Affiliations:** From the ‡VIB/UGent Center for Plant Systems Biology, 9052 Ghent, Belgium;; §Ghent University, Department of Plant Biotechnology and Bioinformatics, 9052 Ghent;; ¶VIB/UGent Center for Medical Biotechnology, 9000 Ghent, Belgium;; ‖Ghent University, Department of Biochemistry, 9000 Ghent, Belgium;; **Ghent University, Department of Mathematical Modeling, Statistics and Bioinformatics, 9000 Ghent, Belgium

## Abstract

Proteogenomics is an emerging research field yet lacking a uniform method of analysis. Proteogenomic studies in which N-terminal proteomics and ribosome profiling are combined, suggest that a high number of protein start sites are currently missing in genome annotations. We constructed a proteogenomic pipeline specific for the analysis of N-terminal proteomics data, with the aim of discovering novel translational start sites outside annotated protein coding regions. In summary, unidentified MS/MS spectra were matched to a specific N-terminal peptide library encompassing protein N termini encoded in the *Arabidopsis thaliana* genome. After a stringent false discovery rate filtering, 117 protein N termini compliant with N-terminal methionine excision specificity and indicative of translation initiation were found. These include N-terminal protein extensions and translation from transposable elements and pseudogenes. Gene prediction provided supporting protein-coding models for approximately half of the protein N termini. Besides the prediction of functional domains (partially) contained within the newly predicted ORFs, further supporting evidence of translation was found in the recently released Araport11 genome re-annotation of Arabidopsis and computational translations of sequences stored in public repositories. Most interestingly, complementary evidence by ribosome profiling was found for 23 protein N termini. Finally, by analyzing protein N-terminal peptides, an *in silico* analysis demonstrates the applicability of our N-terminal proteogenomics strategy in revealing protein-coding potential in species with well- and poorly-annotated genomes.

Proteogenomics is an interdisciplinary research field combining proteomics, transcriptomics, and genomics with the aim of delineating protein-coding regions in genomes, thereby aiding protein discovery and genome annotation (1, 2). Such strategies have identified new variants of proteins, termed proteoforms (3), which arise from nucleotide polymorphisms (4–6), alternative translation initiation (*i.e.* N-terminal (Nt ^1^)-proteoforms (7, 8)), splicing (5, 6, 9), frame-shifts (10) and post-translational modifications (6). Proteogenomic strategies vary depending on the experimental data used and the annotation depth of the studied model system (11). Important for proteomics-driven proteogenomics are customized protein databases that allow for more accurate protein identification using tandem mass spectrometry (MS/MS) data, thereby leading to the refinement of protein-coding gene segments and the discovery of novel gene products. In Arabidopsis, previous proteogenomic studies reported on the use of a protein sequence database based on six-frame translation (6-FT) of the entire genome (12, 13), which was searched in parallel with *ab initio* predicted genes in case of Castellana *et al.* (12). Overall, these efforts resulted in the reclassification of 99 pseudogenes into protein-coding genes, next to the refinement of existing gene structures in the TAIR9 genome release (12–14).

Besides 6-FT or genome-based gene prediction, OMICS data can also aid in the rational design of customized protein databases (2, 15). By providing direct evidence of *in vivo* protein synthesis, the sequencing of ribosome-protected mRNA fragments by ribosome profiling (ribo-seq) serves such a purpose. In eukaryotes, ribosomes can be specifically halted at translation initiation sites (TIS) using initiation-specific translation inhibitors (*e.g.* lactimidomycin and harringtonine; 16, 17). By depleting for elongating ribosomes, this approach allows mapping of the translation initiation landscape and, concomitantly, ORF delineation (16–18). We previously used such ribo-seq data to generate customized databases for MS/MS searches, resulting in the identification of proteoforms initiating at near-cognate start sites, N-terminally truncated and extended proteoforms, translation products of upstream ORFs as well as previously unannotated proteins (8, 19–21).

Whereas shotgun proteomic data have been primarily used for proteogenomic studies, data originating from subproteome analysis have proven to be resourceful as well. For instance, a peptidomic workflow that enriches for small proteins and peptides was used for the discovery of protein-coding small ORFs in human (22, 23). In Arabidopsis, a proteogenomic study (12) made use of enriched phosphopeptides as these often originate from low abundant proteins that can be absent in shotgun proteomics data (24). Further, positional proteomics, enriching for peptides holding protein N termini that can be considered as proxies of translation initiation, has been used for discovering and refining protein-coding gene structures in mouse and human cells (8, 18–20), as well as in bacteria (25–27) and archaea (28, 29). Previously, we presented PROTEOFORMER, a tool which allows for the creation of protein sequence databases for proteomics-based identification based on translation initiation data obtained by ribosome profiling (8). All TIS identified by ribo-seq can then be matched with Nt-proteomics data (8, 18, 19) to improve protein identification rates.

Although entire genome translation databases are criticized because they suffer from the “needle in the haystack” problem (2, 20, 30), especially in the case of eukaryotes, a rationalized reduction of database size benefits the sensitivity for identifying novel peptides or proteins (2, 30). Here, we constructed an Nt peptide database tailored for searching Nt-proteomics data and permitting genome-wide searches for TIS without causing a drastic increase in the peptide search space. After applying data and feature dependent selection criteria, several newly identified N termini were confirmed by ribosome profiling data and other types of supportive metadata.

## EXPERIMENTAL PROCEDURES

### 

#### 

##### A. thaliana Cell Suspension Cultures

*A. thaliana* cell suspension cultures ecotype Landsberg erecta (Plant Systems Biology-Light, Arabidopsis Biological Resource Center stock CCL84841) were cultured as described (31). The cells were subcultured every week in fresh medium at a 1:10 dilution in 500 ml conical flasks and shaken at 125 rpm at 25 °C in an orbital shaker under continuous light (50 μE). Two days after subculturing, cell suspensions were harvested for ribosome profiling or proteomics analyses as described below.

##### N-terminal COFRADIC and LC-MS/MS Analyses

For Nt-COFRADIC analysis, 50 ml of cell suspensions at an 1–2% packed cell volume (PCV) were collected on a Whatman® membrane filter with nylon pore size 0.45 μm using sintered glass filtration, followed by a ice-cold PBS wash. Collected cells were subjected to snap freezing in liquid nitrogen and frozen cell pellets were ground into a fine powder using a liquid nitrogen cooled pestle and mortar. The frozen powder was thawed in 10 ml ice-cold buffer (50 mm sodium phosphate pH 7.5, 100 mm NaCl and 1 × cOmplete™, EDTA-free protease inhibitor mixture (Roche, Basel, Switzerland), left on ice for 10 min and the mixture was subjected to one additional cycle of freeze-thawing. Cell debris was eliminated by centrifugation at 16,000 × *g* for 15 min at 4 °C. The supernatant was recovered and the protein concentration determined using the DC Protein Assay Kit from Bio-Rad (Munich, Germany). For all proteome analyses performed, 3 mg of protein material (corresponding to about 1 ml of lysate) was subjected to Nt-COFRADIC analysis as described previously (32) however, in the case of the endoproteinases Glu-C, Asp-N, and chymotrypsin digests, no strong cation exchange (SCX) pre-fractionation was performed. In one of the two tryptic replicates, the SCX prefractionation was omitted. enable the assignment of *in vivo* Nt-acetylation events, prior to digestion, all primary protein amines were blocked using an *N*-hydroxysuccinimide ester of ^13^C_2_D_3_-acetate. Proteomes were digested overnight at 37 °C using mass spectrometry grade trypsin (enzyme/substrate of 1/100, w/w; Promega, Madison, WI), chymotrypsin (1/60, w/w; Promega), endoproteinase Glu-C (1/75, w/w; Thermo Fisher Scientific, Bremen, Germany) or endoproteinase Asp-N (1/200, w/w; Promega) while mixing at 550 rpm. The resulting peptide mixtures were enriched for N-terminal peptides by diagonal chromatography as part of the actual COFRADIC sorting procedure. More specifically, in between two identical reverse-phase peptide separations, internal peptides are reacted with 2,4,6-trinitrobenzenesulfonic acid (TNBS), rendering them more hydrophobic and thereby causing them to shift away from the unmodified N-terminal peptides during the second chromatographic separation. By the addition of H_2_O_2_ to a f.c. of 0.5% for 30′ at 30 °C, a methionine oxidation step was also introduced between the first RP-HPLC separation and the series of secondary RP-HPLC separations, thereby shifting all methionine-containing Nt-peptides to earlier elution times allowing their enrichment (33).

The obtained fractions enriched for protein N termini were introduced into an LC-MS/MS system; the Ultimate 3000 (Dionex, Amsterdam, The Netherlands) in-line connected to an LTQ Orbitrap XL mass spectrometer (Thermo Fisher Scientific) and LC-MS/MS analysis was performed as described previously (34, 35).

##### MS/MS (customized) Database Searches and Data Storage

##### Standard Database Searches

MS/MS peak lists were searched in parallel using three mass spectrometry search engines and with identical parameter settings when possible. A multistage search strategy was used: MS/MS spectra were first searched against the Arabidopsis proteome database (TAIR10, containing 35,386 entries; http://www.arabidopsis.org/), and unidentified spectra were used as input for a second MS/MS search against a customized peptide database (see next paragraph).

The search engines used were COMET (36; version 2016.01 rev. 2), Crux (37; version 2.1.16866) and MS-GF+ (38; version 2016.06.29). Mass tolerance on precursor ions was set to 10 ppm and on fragment ions to 0.5 Da. Peptide length was 7 to 40 amino acids. Semispecific enzyme settings adjusted to the enzyme and to the available options were used in the search engines (see Table I). ^13^C_2_D_3_-acetylation of lysine side-chains, S-carbamidomethylation of cysteine (+ 57.02 Da) and methionine oxidation to methionine-sulfoxide (+ 15.99 Da) were set as fixed modifications. Variable modifications were ^13^C_2_D_3_-acetylation (+ 47.04 Da) and acetylation (+ 42.01 Da) of peptide N termini. Pyroglutamate formation of Nt-glutamine (- 17.03 Da) was additionally set as a variable modification. Per LC-MS/MS run, the three resulting identification files were parsed and the peptide-to-spectrum matches (PSMs) and their respective scores were obtained. Although for COMET and Crux the cross-correlation score (XCorr) was parsed, in the case of MS-GF+ the expectation value (e-value) was used. The false discovery rate (FDR) was estimated upon searching a concatenated target-decoy database, generated by adding reverted protein sequences to the target database (39). We used the FDR values as search engine-independent scores to combine results from the different search engines using the combined FDR score method described by Jones *et al.* (40). Only PSMs with a FDR score ≤ 0.01 for an individual search engine or a combined FDR score ≤ 0.01 across multiple search engines were further considered. To correct for the fact that certain PSM sets shared between search engines do not contain decoy hits for FDR estimation (40), PSMs are required to have a FDR score ≤ 0.05 for at least one of the individual search engine results.

**Table I:** Enzyme specificity settings. For all searches, enzyme specificity was restricted to one terminus (semi-specificity), in case of COMET this could specifically be set to the peptide C-terminus. For COMET and Crux, the respective cleavage syntax used was specified where ↓ denotes cleavage and X represents any amino acid. ^1^Cleavage after Lys is prohibited given the acylated side-chains of Lys upon executing the Nt-COFRADIC protocol (60)

Sequencing Protease	COMET	Crux	MS-GF+	Missed cleavages
Trypsin^1^	R↓X	R↓X	ArgC	2
GluC	DE↓X	DE↓X	GluC	3
Chymotrypsin	FWYLM↓X	FWYLM↓X	Chymotrypsin	3
AspN	X↓D	X↓D	AspN	3

All mass spectrometry proteomics data and search results have been deposited to the ProteomeXchange Consortium via the PRIDE (41) partner repository with the data set identifier PXD004896 and project name“N-terminomics Proteogenomics” (http://ww.ebi.ac.uk/pride/archive/projects/PXD004896).

##### Customized N-terminal Peptide Database Searches

A 6-FT database of the Arabidopsis genome (TAIR10 release) was created using the “getorf” function from the European Molecular Biology Open Software Suite (EMBOSS, 42). ORFs, delineated by start and stop codons, were required to have a minimum length of 24 nucleotides (*i.e.* leading to at least 8 amino acids). Alternative translation was allowed from “CUG” and “GUG” codons, as besides canonical translation initiation at “AUG,” these near-cognate start codons represent commonly used start codons in eukaryotes and are decoded to iMet (43). In addition, an in-house developed Perl script was used to generate and add shorter protein sequences arising from downstream (AUG) TIS. Besides the 6-FT translation, (spliced) gene models were predicted with Augustus 2.5.5 (44, 45) and corresponding ORFs were delineated. Here, the pre-trained model for Arabidopsis was used, allowing alternative transcript prediction from evidence and probabilistic sampling (100 iterations). All predicted protein sequences were concatenated to the 6-FT translation derived protein sequence database.

Instead of searching full length translated ORF protein sequences, we reduced the search space by filtering out the anticipated protein Nt-peptide sequences using an in-house developed Perl script. First, we extracted all Nt-peptide sequences (8–30 AA in length) of all the *in silico* translated ORFs considering the specificities of the protease used (Table I). Note that a maximum peptide length of 30 amino acids was considered as peptides with lengths ≤ 30 amino acids represented the majority (99%) of TAIR10 identified peptides (supplemental Fig. S1). Although no missed cleavages were allowed for the semi-ArgC peptide library, one missed cleavage was allowed for the semi-AspN/GluC/chymotrypsin libraries. According to the rules of initiator methionine (iMet) processing (46), in case of iMet-starting N termini followed by amino acids with a small gyration radius, Ala, Cys, Gly, Pro, Ser, Thr, or Val, the Nt-peptide sequence with its iMet removed was additionally considered. In the last phase, the peptide library was matched with the TAIR10 proteome and cRAP database (common Repository of Adventitious Proteins, http://www.thegpm.org/crap/) and only nonmatching peptides were retained. During peptide sequence matching, no discrimination was made between Ile or Leu given their identical masses. A detailed overview of the number of N termini and corresponding ORF sequences during the construction of the semi-ArgC Nt-peptide library is given in supplementalFig. S2.

For the proteogenomic search, TAIR10 unidentified spectra were re-indexed using an in-house Perl script. Search parameters were almost identical as described above however, enzyme settings were set to no cleavage to enable the identification of only full-length peptides stored in the customized database. Novel peptides with a FDR score ≤ 0.01 were filtered as described for the TAIR10 searches. Importantly, for the customized searches, only PSMs identified by at least two search engines were considered.

##### Ribosome Profiling

For ribosome profiling, 10 ml of cell suspensions at 4% packed cell volume (PCV) were incubated with either 50 μm lactimidomycin (LTM; 47, 48) or 100 μg/ml cycloheximide (CHX) (Sigma, St. Louis, MI) at 25 °C for 5 and 30 min respectively. Subsequently, cells were collected on a Whatman® membrane filter with nylon pore size 0.45 μm using sintered glass filtration, followed by a wash with ice-cold PBS with 100 μg/ml CHX added. The cells were then subjected to snap freezing in liquid nitrogen and frozen cell pellets (about 400 mg) were ground into a fine powder using a liquid nitrogen cooled pestle and mortar. The frozen powder was re-suspended and thawed in 1.3 ml ice-cold lysis buffer for polysome isolation (10 mm Tris-HCl, pH 7.4, 5 mm MgCl_2_, 100 mm KCl, 1% Triton X-100, 2 mm dithiothreitol (DTT), 100 μg/ml CHX, cOmplete™, EDTA-free protease inhibitor mixture (Roche), vortexed and left on ice for 10 min with periodical agitation. Lysates were subsequently passed through QIAshredder spin columns (Qiagen, Hilden, Germany) and cell debris was removed by centrifugation at 16,000 × *g* for 10 min at 4 °C. The supernatant was subjected to RNase I (Thermo Fisher Scientific) digestion using 1500 U RNase I (about 200 U per mg of protein). Digestion of polysomes proceeded for 45 min at 25 °C with gentle agitation at 400 rpm and the reaction was stopped by adding 600 U of SUPERase●In™ RNase Inhibitor (Thermo Fisher Scientific). Subsequent steps were performed as described (21) with minor adjustments. Ribosome protected fragments with sizes ranging from 26–34 nucleotides were extracted from 3 × 20 μg of RNA. Ribosomal RNA derived contaminants were depleted from size-selected and dephosphorylated RNA fragments using magnetic Ribo-Zero™ rRNA Removal Kits for plant leaf tissue (Illumina, San Diego, CA) according to the manufacturer's instructions. Samples were amplified by PCR using compatible primers (forward sequencing primer: 5′-AATGATACGGCGACCACCGAGATCTACAC-3′, indexed reverse primer 1: 5′-CAAGCAGAAGACGGCATACGAGATACTGATGTGACTGGAGTTCAGACGTGTGCTCTTCCG-3′ (Forward index (5′→3′) ATCAGT) and indexed reverse primer 2: 5′-CAAGCAGAAGACGGCATACGAGATGTCAGCGTGACTGGAGTTCAGACGTGTGCTCTTCCG (Forward index (5′→3′) GCTGAC). The resulting ribosome-profiling libraries were duplexed (samples treated with the same inhibitor, LTM or CHX, were joined) and the obtained cDNA libraries were sequenced on a HiSeq 200 instrument (Illumina) to yield 50 bp single-end reads.

##### Ribosome Profiling Data Analysis

The by ribosome profiling sequenced reads of the CHX and LTM libraries were processed using PROTEOFORMER (8). The tool to clip adaptor sequences was set to fastx_clipper and contaminating sequences were eliminated by allowing PROTOFORMER to discard all sequences that align to the *Arabidopsis thaliana* rRNA and tRNA sequences. For the delineation of TIS, the minimum threshold R_LTM_-R_CHX_ was set at 0.03 and a minimum profile coverage of 10 for all TIS categories. All other parameters were set at their default values.

##### MS^2^PIP

The MS^2^PIP prediction server (49) was used to compute the Pearson correlation coefficient between the theoretical (CID model) and experimental spectra of identified TAIR10 database annotated N-terminal peptides (*i.e.* position 1 or 2) and Nt-peptides identified in the proteogenomic search. Discerning tryptic from nontryptic peptides and different charge states, the median Pearson correlations of the database annotated Nt-peptides were used as thresholds to filter proteogenomic peptides.

##### TermiN*ator3*

To predict the Nt modification status, Termi*N*ator3 (http://bioweb.i2bc.paris-saclay.fr/terminator3/; 46, 50) was used. As input, the identified peptide sequences containing the iMet were used, and the parameters were set for plant nuclear genomes without leader peptide removal.

##### Protein-level Annotation Resources

The UniProt knowledge base (UniProtKB, http://www.uniprot.org/, 51) and Entrez Protein, part of the National Center for Biotechnology Information (NCBI) database (https://www.ncbi.nlm.nih.gov/protein) were queried for the novel Nt-peptides. Novel identified Nt-peptides were matched to both resources while making no distinction between the isobaric amino acids Ile and Leu. Besides curated proteins, both resources contain computationally annotated proteins that largely stem from *in silico* translations of deposited nucleotide sequences. For instance, TrEMBL (52) is a subdivision of UniProtKB that stores 62,333 predicted protein sequences derived from EMBL nucleotide translations (accessed October 2016). In addition, Entrez Protein contains 287,230 protein sequences for *Arabidopsis thaliana*, of which 151,231 originate from *in silico* translations of sequences stored in GenBank (53, accessed October 2016). In addition, to identify protein domains in novel discovered proteoforms or proteins, we used the online InterPro (54) sequence search (http://www.ebi.ac.uk/interpro/search/sequence-search) on default settings.

##### Integrative Genome Browser and Sequencing Data Availability

Ribo-seq sequencing data have been deposited in NCBI's Gene Expression Omnibus and are accessible through GEO Series accession number GSE88790 (https://www.ncbi.nlm.nih.gov/geo/query/acc.cgi?acc=GSE88790). For visualization, the Arabidopsis TAIR10 genome was loaded in the Integrative Genome Browser (IGV version 2.3.66; 55). Gene annotation from Araport (June 2016, GTF-format), the Augustus predicted gene models (GTF-format) and the identified peptides (BED-format) were loaded as additional tracks.

##### Sequence Conservation

Genome alignments of *Arabidopsis thaliana* to *Arabidopsis lyrata* and *Brassica rapa* were downloaded from EnsemblPlants release 33 (ftp://ftp.ensemblgenomes.org/pub/plants/release-33/maf/). The aligned sequence starting from the genomic start codon coordinates of the Nt-peptides was parsed, if present in an alignment block. For the 56 aligned sequences with a near-cognate or canonical start codon, an *in silico* translation was performed using the getorf function from the European Molecular Biology Open Software Suite (EMBOSS, 42). No translation on the reverse strand was considered and the minimum ORF nucleotide size was set to six nucleotides. *In silico* translated proteins were filtered for peptides starting from the conserved start codon and having a minimum length of the Arabidopsis identified peptides, or the first exon in case of a Nt-peptide over-spanning a splice junction.

The TIS sequence context for *A. thaliana* was determined by extracting nucleotide frequencies for −5 to +4 positions of all TAIR10 protein-coding gene models (35,386 entries). After omitting 6,602 redundant start codons, because of alternative transcripts starting from the same TIS, nucleotide frequencies were calculated and plotted (Weblogo, http://weblogo.berkeley.edu/; 56). For TIS with a single genomic location (111 out of 117), a TIS sequence context score was calculated using the TAIR10 nucleotide frequencies by the method described by Grzegorski *et al.* (57). Briefly, TAIR10 nucleotide frequencies of the nucleotides present at the −5 to −1 and +4 positions in a given TIS context are summed up to determine the TIS context score. For the newly identified TIS giving rise to proteins starting with consecutive methionines, only the first start codon was considered.

##### Experimental Design and Statistical Rationale

In total, five Nt-COFRADIC analyses (*i.e.* two tryptic replicates and a single proteome analysis in case of AspN, GluC and chymotrypsin digested samples) were performed with proteomes extracted from *A. thaliana* cell suspension cultures ecotype Landsberg erecta (PSB-L). The focus in this paper lies on peptide and protein identification: in this aspect, the different Nt-COFRADIC analyses were regarded as technical replicates. Further, in the TAIR10 reference searches, 81,340 peptide-spectrum-matches yielded 44,103 N termini originating from 7972 proteins and thus on average, each N terminus was identified by two spectra. Ribo-seq analysis of LTM and CHX-treated cultures was performed on cell culture material that was obtained in parallel.

## RESULTS

### 

#### 

##### Identification of Protein N-terminal Peptides

Protein Nt-peptides were enriched from Arabidopsis cell culture proteomes by Nt-COFRADIC (32). Isolated proteomes were digested with either trypsin, chymotrypsin or the endoproteinases GluC or AspN, to increase the chances of identifying Nt-peptides and thus to increase the overall proteome coverage (58, 59). Importantly, Nt-peptides isolated by means of Nt-COFRADIC are readily distinguished from all other peptides as they are *in vivo* or *in vitro* acylated at their alpha-amine. To account for possible Nt-protein processing events, we made use of semispecific enzyme search settings for MS/MS searches (60), inevitably causing an increase of the peptide search space (supplemental Fig. S3). To increase the number of identified Nt-peptides, all MS/MS data were searched in parallel with three search engines: COMET (36), Crux (37) and MS-GF+ (38). Results from individual searches were aggregated by calculating the combined FDR score as described by Jones *et al.* (40).

**Fig. 1.**
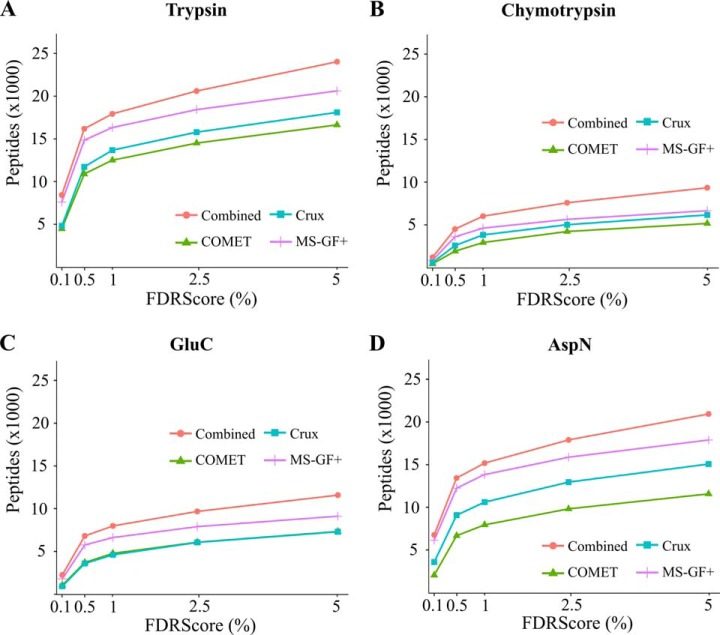
**Number of unique peptide sequences identified for (*A*) trypsin, (*B*) chymotrypsin, (*C*) endoproteinase GluC, and (*D*) endoproteinase AspN digested proteome samples, plotted against different FDR score thresholds (*x* axis).** The combined (red dots) and the individual search engine results are shown (see legend).

Peptides reported by individual and multiple search engines (FDR score ≤ 0.01) are shown in Fig. 1. For all four proteases, the combined search results complemented and increased the total number of identifications. Taken together, all PSMs (supplemental Fig. S4) led to the identification of 50,901 target peptides and 1723 decoy peptides (peptide FDR = 0.03). Overall, MS-GF+ performed the best in terms of identifications for all samples compared with other search engines. The four proteases used showed differences in their cleaving efficiency, reflected by the frequency of missed cleavages observed. As anticipated, trypsin is more efficient (92.6% of peptides had no missed cleavage) than chymotrypsin and endoproteinase GluC, both for which often peptides are identified with higher number of missed cleavages (61; supplemental Fig. S5).

##### Protein N Termini and Their Modifications

Nt-peptides can be grouped into different classes according to their Nt-modification. Generally, a large portion of Nt-peptides are *in vivo* Nt-acetylated (32), as this is a ubiquitous co-translational modification in higher eukaryotes (62). In all our setups analyzed, *in vitro* ^13^C_2_D_3_-acetylation was used (34, 35, 63). When only considering Nt-peptides matching a single protein, 28,677 (35.3%) spectra matched *in vitro* Nt-acetylated peptides, whereas 19,686 (24.2%) spectra matched *in vivo* acetylated peptides (supplemental Table S1). Note that the high number (37.9%) of non-Nt-peptides can be explained by the fact that no SCX step pre-enriching for Nt-peptides was used for nontryptic digested samples (63) and that for one of the two tryptic replicates no SCX pre-fractionation was performed to favor identification of His-containing peptides.

Nt-peptides can also be categorized according to their starting position in protein sequences. Whereas 81% of the *in vitro* Nt-acetylated peptides are located downstream in the corresponding protein sequence, most *in vivo* Nt-acetylated peptides are database-annotated protein N termini (16,590 from 19,686 [84%], supplemental Table S1). This observation is in line with our previous studies reporting on 80 to 90% of TIS-indicative N termini from proteins of higher eukaryotes being *in vivo* Nt-acetylated (64). Further, Nt-methionine excision (NME) at protein N termini by methionine aminopeptidases (MetAPs) represent an omnipresent co-translational process (46). NME can be accurately predicted based on a protein sequence by tools such as Termi*N*ator3 (46, 50). An earlier study of protein N-terminal modifications in Arabidopsis demonstrated the NME prediction of Termi*N*ator3 to be highly accurate (65). This was also the case for our experimentally observed protein N termini, as 21,982 of the 22,090 PSMs (99%) matched the Termi*N*ator3 NME prediction.

##### Generation of a Customized N-terminal Peptide Sequence Database for Proteogenomics in Arabidopsis

**Fig. 2.**
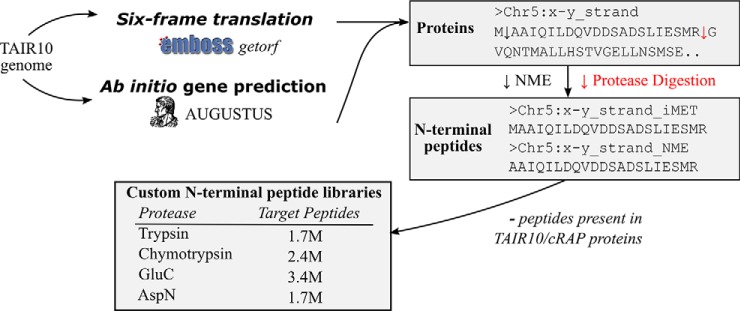
**Generation of customized Nt-peptide libraries.** A six-frame translation of the TAIR10 genome was performed by the EMBOSS getorf program (42) or subjected to *ab initio* gene prediction by Augustus 2.5.5 (44, 45). Resulting protein sequences were *in silico* digested by any of the four proteases used. Only Nt-peptides starting at position 1 or 2, considering the NME rule, were retained. Peptides matching the TAIR10 proteome or the cRAP database (common Repository of Adventitious Proteins, http://www.thegpm.org/crap/) were omitted. The resulting number of non-redundant target peptide sequences are shown for every protease.

For our proteogenomic investigation, we further applied a multistage data analysis strategy. Following the canonical TAIR10 database search, unidentified MS/MS spectra were searched against a customized database. Considering searches using TAIR10, 22,090 out of 48,363 (45.7%) PSMs corresponded to Nt-acetylated protein N termini (see supplemental Table S1). Given the positional information provided by protein N termini, we compiled protease-specific *in silico* libraries of Nt-peptides corresponding to all theoretical translation start sites residing in the genome (Fig. 2). Thus, instead of searching full length protein sequences from a 6-FT of the genome, we drastically reduced the search space by only focusing on protein N termini. Integration of ribosome profiling and (Nt-) proteomic data in higher eukaryotes already pointed to translation starting from these codons (8, 17–19) and evidence for this type of alternative translation was previously also found in Arabidopsis (43). Therefore, we also included peptides to start from these near-cognate start codons, which are translated to iMet. Further, we performed *ab initio* gene prediction using the generalized hidden Markov model based software tool Augustus (44, 45). This extends the lists of putative ORFs, and thus protein Nt-peptides, by additionally considering Nt-peptides over-spanning a transcript splice site. For all ORFs, we extracted the “semi-digested” Nt-peptides (if compatible) according to the specificities of the proteases used. This resulted in four protease-specific peptide databases encompassing 1.7 to 3.4 million target Nt-peptides. For comparison, a semi-ArgC digested TAIR10 six-frame translated genome (start-to-stop translation, no missed cleavages) yields about 79 million such peptides. Similar as for the TAIR10 searches, a concatenated target-decoy database was used to enable FDR estimation.

##### Identification of Translation Initiation Sites Not Annotated in TAIR10

**Fig. 3.**
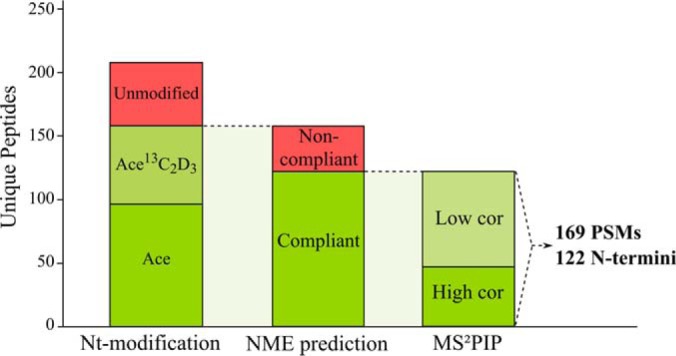
**Peptide identifications pointing to novel TIS.** Of all identified novel peptides, solely Nt-modified peptides, *i.e. in vitro* or *in vivo* Nt-acetylated and NME compliant N termini were considered, resulting in a total of 122 novel Nt-peptides (169 PSMs; supplemental Data Set S1). As additional support, MS^2^PIP Pearson correlations were computed, where high correlation indicates a correlation higher than the median correlation observed for spectra matching TAIR10 database annotated N termini (supplemental Fig. S6).

The TAIR10 unidentified MS/MS spectra were searched against the customized Nt-peptide databases. Here, we applied more stringent selection criteria than those used for the initial TAIR10 searches, by requiring a PSM to be identified by at least two out of three search engines. In total, 208 peptides (259 PSMs, FDR score ≤ 0.01, Fig. 3) were identified of which 96 peptides (46.2%, 136 PSMs) were *in vivo* Nt-acetylated, 62 peptides (29.8%, 72 PSMs) were *in vitro* Nt-acetylated and 50 had a Nt-free N terminus (24%, 51 PSMs). We analyzed how these 158 novel blocked Nt-peptides matched the NME rules as predicted by Termi*N*ator3 (46, 50), which proved highly accurate for the TAIR10 Nt-peptides (99%, see above). Next to the requirement of *in vivo* or *in vitro* acetylation, NME specificity rules served as another filtering step to assess peptides reporting novel translation start sites. In total, 122 out of 158 Nt-peptides (77%, 169 out of 208 PSMs, Fig. 3) matched the NME prediction by Termi*N*ator3. Detailed information of the 169 PSMs, such as identification score and NME prediction, is available in supplemental Data Set S1, whereas the annotated spectra are provided in supplemental Data Set S2. The 122 identified Nt-peptides correspond to 117 unique protein N termini pointing to possible unannotated TIS that are listed in Supplemental Data set 3. Note that for 5 TIS, two Nt-peptide sequences of different length were identified, which resulted from digestion by different proteases (see supplemental Table S2). To further assess the reliability of the PSMs, the MS^2^PIP prediction server (49) was used to compute the correlation between theoretical and observed MS^2^ spectra for TAIR10 database annotated and novel Nt-peptides. The average distribution of the tryptic Nt-peptides was found to be in line with those reported in the original publication (49). Of note however lower correlations were observed in case of the nontryptic spectra (supplemental Fig. S6), (in part) explained by the fact that the available MS^2^PIP models are trained on tryptic data only. In total there were 65, novel Nt-peptides with a correlation higher than the median correlation observed for TAIR10 spectra, the latter considered high confident spectra (supplemental Fig. S6).

##### Newly Identified Protein N Termini Reveal Alternative Gene Model Structures

**Fig. 4.**
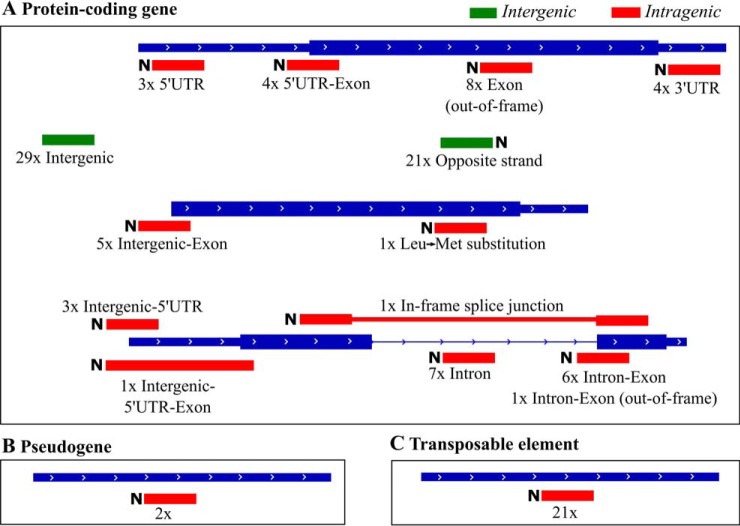
**Positioning of new Nt-peptides in relation to TAIR10 protein-coding gene models (*A*), pseudogenes (*B*) or transposable elements (*C*).** Intergenic Nt-peptides, *i.e.* not overlapping with a TAIR10 transcript, are shown in green, whereas intragenic Nt-peptides are shown in red. The number of identified peptides as well as the N terminus of a peptide (‘N′) are indicated, except for intergenic Nt-peptides.

In proteogenomics, novel peptides can be organized in different types according to their relationship with existing gene models. Manual inspection of all 117 novel TIS locations (see “Location TAIR10” section in supplemental Data Set S3), indicated that 50 were located at intergenic regions (Fig. 4*A*). The other TIS were intragenic, either (partly) overlapping with a TAIR10 protein-coding gene model (44 TIS, Fig. 4*A*), a pseudogene (2 TIS, Fig. 4*B*) or a transposable element gene (21 TIS, Fig. 4*B*).

The interpretation of novel TIS in relation to annotated protein-coding gene models (Fig. 4*A*) can be complex, especially in eukaryotes when considering (alternatively) spliced transcripts. For instance, a TIS encoded upstream of a protein-coding gene could be an indicator of a novel upstream ORF (uORF), a novel exon or an in-frame extension of the first exon. In this regard, gene models predicted by Augustus during the peptide library construction give additional support. Of the 117 novel TIS, 27 (23%) correspond to a protein N terminus encoded by an Augustus predicted gene model (supplemental Table S3, see ‘Gene model’ section in supplemental Data Set S3). The TIS not predicted by Augustus were used as extrinsic information, or hints, for another round of gene prediction by Augustus (44, 45). This resulted in an additional 32 Nt-peptides supported by at least one predicted Augustus gene model. Thus, in total, approximately half of the Nt-peptides (59 out of 117) matched to Augustus predicted gene models.

Next to Augustus gene predictions, we also considered the Araport11 annotation (66) hosted by the Arabidopsis Information Portal (https://www.araport.org/), which was released during manuscript writing. Araport11 is a completely new reannotation of the Arabidopsis genome based on RNA-seq experiments and contains, among others, several new splice variants of previously TAIR10 annotated proteins. Of all novel, non-TAIR10 annotated TIS, 17 corresponded to annotated protein start sites in Araport11 (supplemental Table S3, see Gene Model section supplemental Data Set S3). These 17 alternative gene models were also predicted by Augustus without (13 models) or with providing genomic coordinates of the identified Nt-peptides of our proteogenomic study as hints (4 models).

**Fig. 5.**
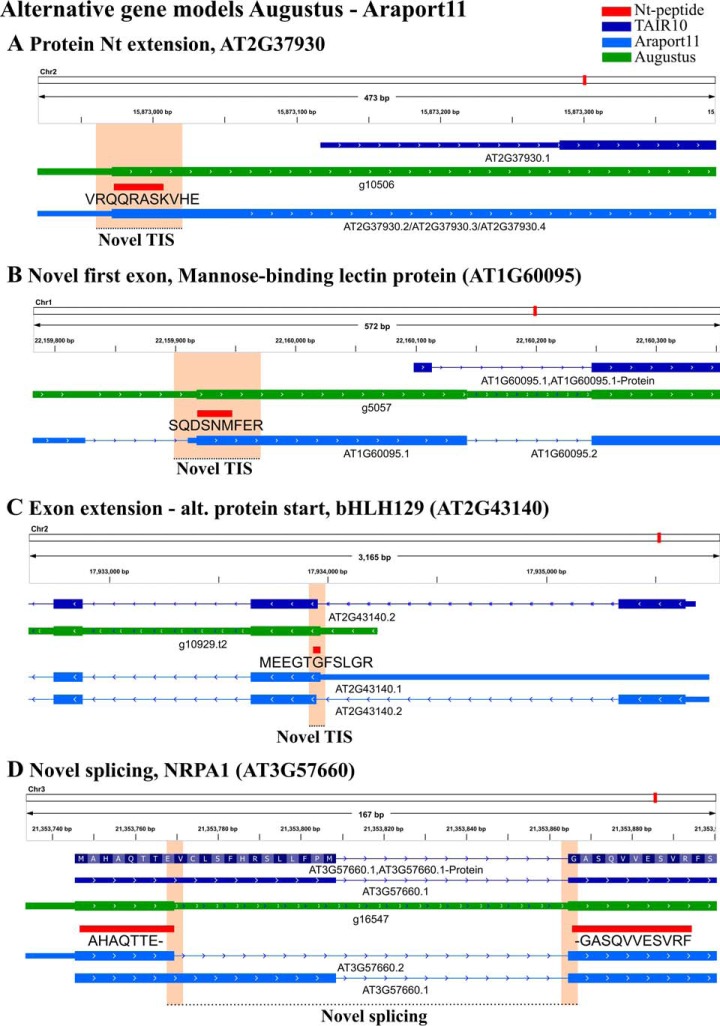
**Novel Nt-peptides matching Augustus predicted gene models and Araport11 annotations.** TAIR10, Augustus predicted and Araport11 annotated gene models in addition to the Nt-peptide sequences identified were loaded as tracks in the Integrative Genome Viewer (IGV; 55). Nt-peptide identifications are shown that hint to the expression of Nt-protein extensions (*A–C*), originate from translation initiation at a novel upstream exon (*B*) an exon extension (*C*) and a newly identified exon-exon splicing event (*D*).

Both the Araport11 and Augustus gene models facilitated the interpretation of Nt-peptide or TIS locations, as demonstrated in Fig. 5. For instance, the TAIR10 intergenic-located peptide “VRQQRASKVHE,” suggests an in-frame extension of the first exon with a length of 104 amino acids matching a predicted Augustus gene model and 3 Araport11 protein-coding models (Fig. 5*A*). Similarly, the Nt-peptide “SQDSNMFER” was located intergenically and upstream of a TAIR10 annotated protein (Fig. 5*B*). However, the peptide is out-of-frame with the annotated protein, and instead of an in-frame extension, gene models point to the presence of a splice site, giving rise to a novel first exon of the TAIR10 protein. Unlike the previous peptides, “MEEGTGFSLGR” starts in an intronic region of a TAIR10 gene model, extending an annotated exon (Fig. 5*C*). The Augustus gene model, predicted after providing hints, was identical to AT2G43140.1 of Araport11 and suggests an alternative start position which is in-frame and a few bp upstream of the TAIR10-annotated second exon of bHLH129. Lastly, the peptide “AHAQTTEGASQVVESVRF” spans a TAIR10-unannotated splice junction of NUCLEAR RNA POLYMERASE A1 (Fig. 5*D*). Thus rather than a novel TIS, we found a novel Nt-peptide pointing to the use of a novel splice site predicted by Augustus.

##### Complementary Evidence of Translation Initiation by Ribosome Footprinting

**Fig. 6.**
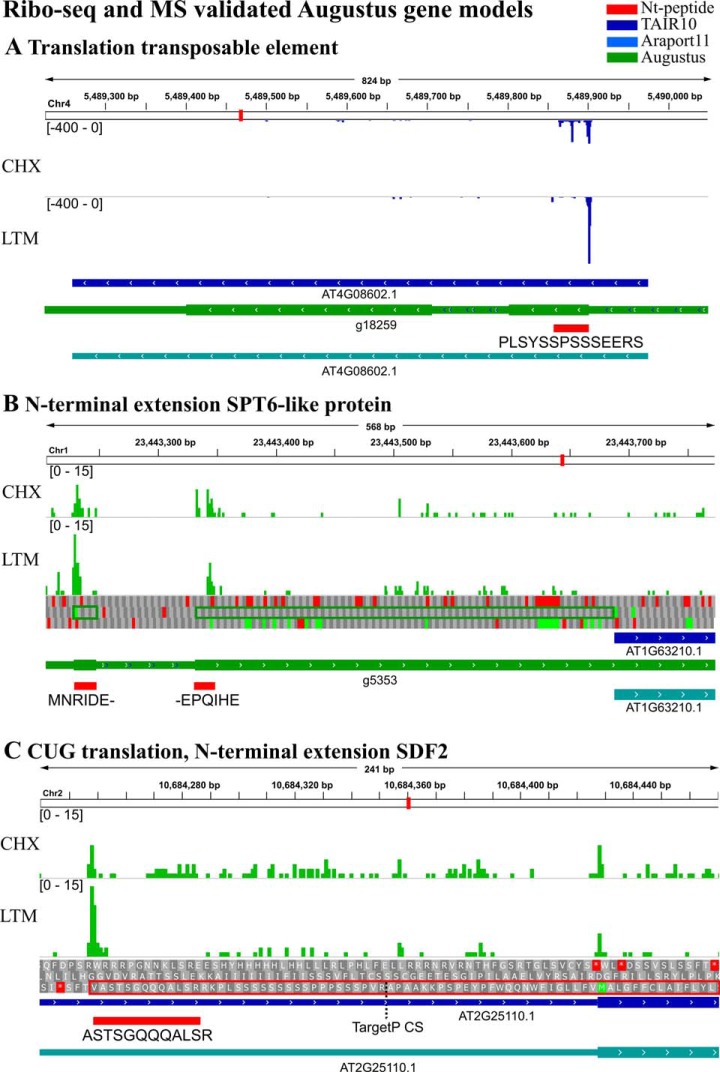
**Ribo-seq supported Augustus gene models with matching Nt-peptides.** Cycloheximide (CHX) and lactimidomycin (LTM) coverage are displayed according the mapped strand (green: forward strand, blue: reverse strand). TAIR10, Araport11 gene models, and the identified peptides were loaded as tracks in IGV (55). *A*, Translation evidence of an annotated transposable element. *B*, Augustus predicted protein extension indicative of a novel splice site (green rectangle). *C*, Alternative translation giving rise to an Nt-extension of SDF2. The cleavage site (CS) prediction by TargetP (71, 72) is indicated by a dotted line.

Next to the proteomic analysis performed, samples obtained from Arabidopsis cell cultures were used for ribosome footprinting (ribo-seq) to gain complementary evidence of translation initiation events identified by our proteogenomics approach. More specifically, we employed lactimidomycin (LTM) and cycloheximide (CHX) treatments to perform genome-wide identification of TIS (16, 17). We then used the PROTEOFORMER package (8) to call TIS sites on the predicted Augustus gene models. Evidence of translation initiation was found for 14 Nt-peptide supported TIS (see Ribo-seq section in supplemental Data Set S3). Two peptides, “ARVKDSSGEY” and “PLSYSSPSSSEERS,” matched 7 and 2 gene models respectively, all located at transposable elements. One of the gene models matching PLSYSSPSSSEERS (Fig. 6*A*) was proposed by Hanada *et al.* (67) to encode for a small ORF (sORF5540) with high coding potential. Besides, matching proteomic and ribo-seq evidence of protein synthesis was found in case of 5 other transposable elements (supplemental Data Set S3). In addition, 7 Nt-protein extensions were found, which extend the first exon in-frame. The longest extension was found for the Augustus gene model g5353, with an extension up to 125 amino acids (Fig. 6*B*, green rectangle) and containing a splice site over-spanned by the Nt-peptide “MNRIDEEPQIHE.” Interestingly, this predicted protein-coding gene was not found in Araport11, despite having clear ribo-seq and proteomic evidence. Furthermore, an InterPro (54) sequence search reveals a Spt6 acidic Nt-domain (IPR028083) inside the extension (supplemental Fig. S7). This matches the known annotation of the extended TAIR10 gene AT1G63210, which was described to be a SPT6-like homolog as it contains the conserved Interact-With-Spt6 binding domain but lacks the C-terminal extension with WG/GW repeats present in AtSPT6 (68).

As our standard PROTEOFORMER pipeline relies on reference transcriptome mapping, only in the case of Augustus predicted TIS, ribo-seq matching TIS evidence could potentially be found. However, some novel Nt-peptides, for which no Augustus gene model was predicted, do appear to have matching ribo-seq data. One of them is the peptide “ASTSGQQQALSR,” which is translated from a CUG start codon putatively giving rise to a 60 amino acid extension of STROMAL CELL-DERIVED FACTOR 2-LIKE PROTEIN PRECURSUR (SDF2; Fig. 6*C*). This alternative upstream TIS shows higher LTM coverage than the annotated start and extends the CHX density, which is present from the TIS along the 5′ UTR toward the annotated start codon (Fig. 6*C*). The SDF2 protein is part of a complex involved in the unfolded protein response in the endoplasmic reticulum (69) and was shown to be important for the proper accumulation of pathogen-associated molecular pattern receptors (70). The discovered 60 amino acid extension form is predicted by TargetP (71, 72) to contain a 35 amino acid long chloroplast targeting peptide (> 90% specificity cut-off). Next to the Nt-protein extension of SDF2, manual inspection revealed translation initiation at an additional 8 TIS matched by novel Nt-peptides (supplemental Data Set S3).

##### Sequence Conservation of Translation Initiation Sites

**Fig. 7.**
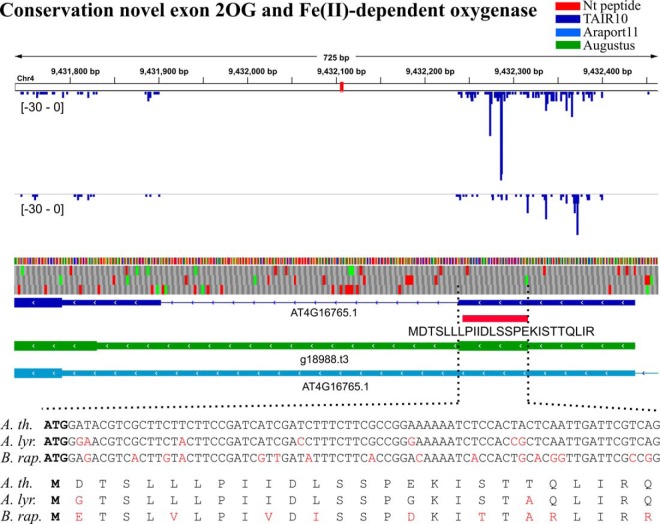
**Sequence conservation of start codon and novel Nt-peptide mapping an upstream exon of the gene encoding 2OG and Fe(II)-dependent oxygenase (AT4G16765).** Cycloheximide (CHX) and lactimidomycin (LTM) coverage are displayed for the reverse strand (blue). TAIR10, Araport11 gene models, and the identified peptides are loaded as tracks in IGV (55). The genomic sequence alignment (*A. thaliana versus A. lyrata,* and *B. rapa*, EnsemblPlants release 33) of the first exon is displayed, the start codons were printed in bold. Translation of aligned sequences to amino acids of which the iMet are indicated in bold. Non-identical nucleotides or amino acids were displayed in red.

Functional regions such as protein-coding sequences in the genome are typically conserved. We checked the conservation of the start codons and adjacent subsequences encoding the novel Nt-peptides by inspecting genome alignments of *Arabidopsis thaliana* to *Brassicaceae* species *Arabidopsis lyrata*and *Brassica rapa.* Of the 111 Nt-peptide sequences with a unique genomic location, 97 and 78 resided in *A. lyrata* and *B. rapa* genomic aligned regions respectively (see Sequence Conservation section in supplemental Data Set S3). Furthermore, for *A. lyrata*, 75 aligned sequences (77%) started with near-cognate or canonical start codons, whereas in *B. rapa*, this was the case for 46 sequences (59%). Taken together, 40 TIS were conserved in both *A. lyrata* and *B. rapa* aligned sequences. We further examined whether the sequence following the 40 conserved start codons maintained the potential to encode an orthologous Nt-peptide. *In silico* translation showed that for 28 aligned sequences (70%) such an orthologous peptide, similar to our identified Nt-peptide, is encoded (supplemental Data Set S3). This was for instance the case for the Nt-peptide “MDTSLLLPIIDLSSPEKISTTQLIR,” a 25 amino acid-long peptide almost spanning the entire length of an exon predicted using extrinsic information (26 amino acids, Fig. 7). This novel predicted exon was highly conserved in *A. lyrata* and *B. rapa* only undergoing minor nucleotide and amino acid changes (indicated in red). Manual inspection shows accompanying CHX coverage and to a minor extent LTM coverage coinciding with the Nt-peptide. When considering the set of 28 conserved TIS, 10 had supporting ribo-seq coverage (supplemental Data Set S3).

**Fig. 8.**
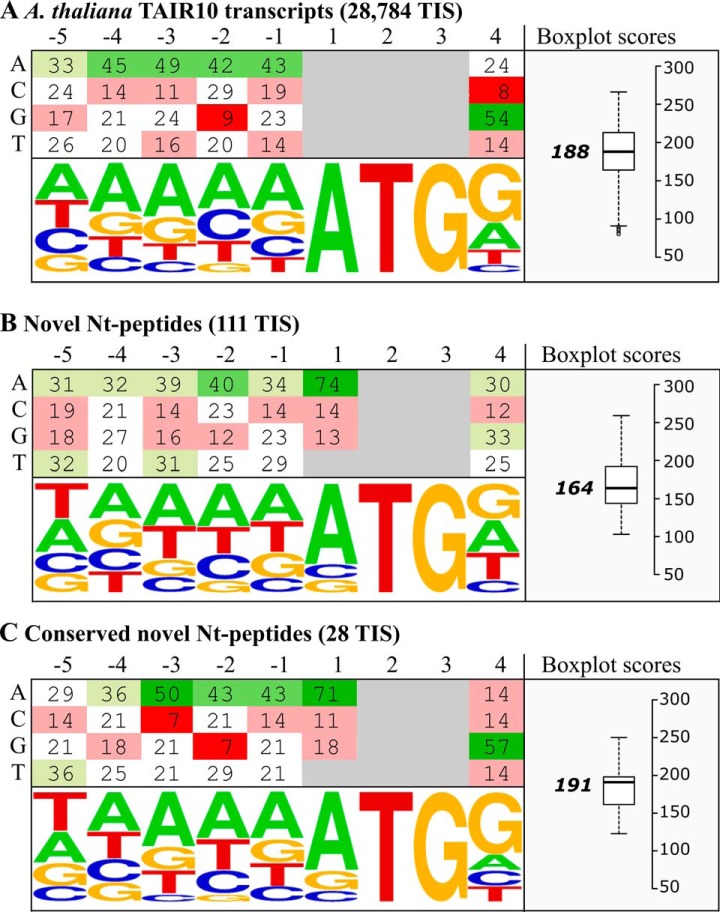
**TIS context sequence frequency and scoring.** Nucleotide frequency plots of the TIS sequence context (positions −5 to +4) of (*A*) all TAIR10 transcripts (28,784 nonredundant TIS), *B*, the novel Nt-peptides (111 TIS) and (*C*) Nt-peptides conserved in *Brassicaceae* (28 TIS). Nucleotide frequency matrices and plots were displayed (left), as well as boxplots showing the nucleotide context score distribution for the respective TIS.

Typically, efficient translation initiation requires the start codon to be embedded in a specific sequence context known as the KOZAK motif (73, 74). Highly efficient TIS typically contain an ATG start codon and a purine at the −3 and G at the +4 nucleotide positions (73, 74). A translation efficiency study in Arabidopsis suggests that adenine residues in nucleotide positions −1 to −5 favor translation initiation (75). When constructing a frequency plot of nucleotide positions −5 till +4 for all TAIR10 transcripts (Fig. 8*A*) and the novel Nt-peptides (Fig. 8*B*), A-residues are prominent in nucleotide positions −1 to −5 and purines (A/G) in position +4. Regarding the start codon distribution of the novel Nt-peptides, 25% were initiated from a near-cognate start codon. To more efficiently score the TIS context conservation of our novel Nt-peptides, we used a sequence context scoring method as described by Grzegorski *et al.* (57). For a given TIS context, the TAIR10 nucleotide frequencies of positions −1 to −5 and +4 were summed up to define a sequence context score. For instance, the most frequent, and thus highest scoring, TIS context sequence was “AAAAAATGG,” giving rise to a score of 266 (= 33 + 45 + 49 + 42 + 43 + 54). When applying this scoring formula to the 111 novel Nt-peptide supported TIS (see KOZAK Sequence section in supplemental Data Set S3), with a unique genomic location, a score distribution relatively lower than the TAIR10 transcripts was observed (median score of 164 compared with 188, Fig. 8*A*–8*B*). However, when considering only the 28 conserved TIS (*i.e.* a conserved start codon and N-terminal peptide coding capacity in *Brassicaceae* (see above)), a score distribution comparable to TAIR10 transcripts was observed (median score of 191 compared with 188, Fig. 8*C*), suggesting that conserved novel TIS contexts are likely embedded in more translational efficient TIS context like TAIR10 TIS contexts. An observation which is at least partly attributable to an elevated frequency of guanine residues at position +4 (57% compared with 33%) and higher frequencies of adenosine residues at positions −4 to −1.

##### Comparison with Other Protein-level Annotation Resources

We here used TAIR10 as the reference proteome as it is the standard annotation for Arabidopsis protein research. In addition, we consulted other databases such as the UniProt knowledge base (UniProtKB, http://www.uniprot.org/, 51) and Entrez Protein, part of the National Center for Biotechnology Information (NCBI) database (https://www.ncbi.nlm.nih.gov/protein). Next to manually curated protein entries (Swiss-Prot (52) for UniProtKB and RefSeq (76) for NCBI), both resources contain computationally annotated protein sequences. Both resources were used to search for additional evidence for our novel Nt-peptides, and thus to possibly provide additional proof for their existence. In total, 33 Nt-peptide supported TIS matched Entrez Protein entries, of which 25 were also present in UniProt/TrEMBL (see Gene Model section in supplemental Data Set S3). All 33 Nt-peptides had an Augustus predicted gene model and for 18 translational evidence was obtained by ribo-seq (Table II). We further considered how TIS sequence conservation related to the other meta-data at hand. To this end, we selected the 28 TIS that were conserved in *Brassicaceae* (Fig. 8*C*, supplemental Data Set S3). Interestingly, 12 conserved TIS had no Augustus predicted model and one of them had matching ribo-seq evidence (Table II), among which the Nt-peptide “ASTSGQQQALSR” (Fig. 6*C*). Also note that high MS^2^PIP correlations typically corresponded to Nt-peptides with matching meta-data, indicating its value to gain additional confidence in proteogenomic analysis. Further inspection of the Nt-peptides and accompanying meta-data is available in supplemental Data Set S3.

**Table II:**
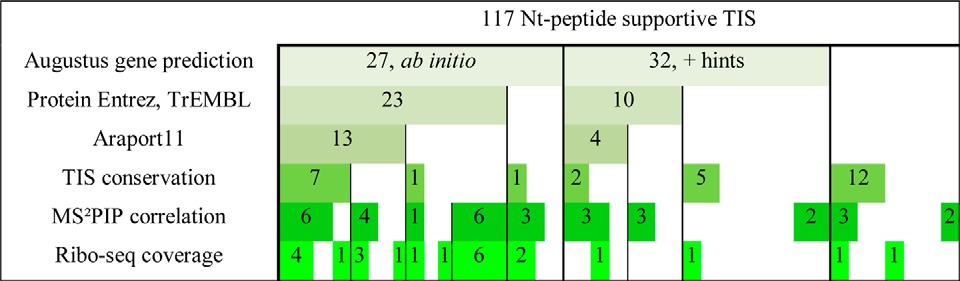
Matching support for the 117 novel TIS. For 59 Nt-peptides a corresponding gene model was predicted by Augustus (44, 45) either with (32 TIS, “+ hints”) or without (27 TIS, “ab initio”) extrinsic information. In addition, 33 TIS matched an entry from Protein Entrez (https://www.ncbi.nlm.nih.gov/protein) or TrEMBL (52), of which 17 were annotated in Araport11 (http://www.araport.org/; 66). Further, 28 TIS were evolutionary conserved in Brassicaceae species. For 65 Nt-peptides, a high MS^2^PIP correlation was observed for at least 1 PSM (supplemental Fig. S6). Lastly, ribo-seq evidence was found for 23 Nt-peptide supported TIS (marked bright-green). Corresponding meta-data can be found in the supplemental Data Set S3

## DISCUSSION

Our previous N-terminomics studies revealed a frequent occurrence of alternative translation initiation events in human, mouse and yeast among others (18, 19, 21, 77, 78). To enable the detection of database non-annotated TIS, we performed a proteogenomic analysis tailored for N-terminomics data. Using a multistage search strategy, TAIR10 unidentified MS/MS spectra were searched against a peptide library encompassing all theoretical Nt-peptides starting from canonical and near-cognate start codons (Fig. 2). This yielded 117 novel TIS identifications from Nt-peptides fulfilling NME enzymatic rules (Fig. 3). More than half (73/117, 62.3%) of all non-annotated N termini identified had supportive meta-data obtained by means of ribo-seq, TIS conservation analysis and non-TAIR10 transcript and protein resources (Table II).

Central to our analysis was the creation of a genome-derived Nt-library, encompassing all six-frame translated or *ab initio* predicted protein N termini (Fig. 2, supplemental Fig. S2). Prediction was done by Augustus (44, 45) and allowed the addition of possible N termini over-spanning transcript splice sites. As nearly half (46%) of the TAIR10-matched spectra reported peptides at the protein N terminus (position 1 or 2; supplemental Table S1), such positional restriction is justified and offers a drastic reduction in search size from which the sensitivity and the specificity of detecting novel peptides/proteins benefit (2, 30). A limitation of using such a library is that spectra matching downstream Nt-peptides and thus potential proxies of downstream translation initiation events (after position 2; *i.e.* Nt-truncated proteoforms) are ignored. However, this would require a customized protein sequence database with a semienzymatic specificity that would cause a drastic increase of peptide search space (79; supplemental Fig. S3), making a genome-wide exploration too exhaustive. Customized Nt-peptide databases were previously used to match shotgun spectra in *Drosophila melanogaster* and yeast proteome samples (80). Here, Nt-peptides derived from a three-frame translation 100 bp downstream from annotated protein starts were appended to the reference proteome to identify novel downstream translation start sites. In addition, also neo-Nt-peptides originating from processing during subcellular targeting were considered. However, the analysis mainly focused on the presence of truncated forms because of alternative translation initiation and proteolytic events, as for instance Nt-extensions and translation products of novel genes remained unexplored. Furthermore, novel events were filtered with a global FDR and lacked a subset FDR in case of novel peptides, a requirement which has been put forward as a minimal guideline (2). Nevertheless, it is well demonstrated that a large fraction (22%) of unannotated protein start sites is evident in Drosophila (80), an observation in line with our previous N-terminomics studies in yeast, human and mouse (19–21, 77, 78).

In the plant research community, TAIR genome annotation is most widely used. For instance, when inspecting 110 Arabidopsis proteomic data sets available in the PRIDE repository (40) for which a searched protein database could be retrieved, 89% of the data sets (98 accessions) used TAIR annotation, whereas the remainder used UniProt protein databases. In addition, a previous N-terminomics study pointed to an increased identification rate when searching TAIR10 as compared with previous releases of UniProt/Swiss-Prot (81), as TAIR10 was found to be far more comprehensive. Of the 117 novel Nt-peptide supported TIS identified by our proteogenomic approach, about half (67/117, 57%) overlapped with existing TAIR10 gene models, even though such intragenic peptides only represent a small fraction in the customized peptide libraries; *i.e.* 1.2% of the trypsin Nt-peptide library (19,746 of 1,732,969 entries). To help with the interpretation of the novel Nt-peptides about existing gene models, Augustus (44, 45) gene predictions proved very resourceful. In total, 59 Augustus gene models with a matching Nt-peptide were present, of which 27 without extrinsic information and 32 after providing the genomic coordinates of identified Nt-peptides as hints. Next to gene prediction, we considered a recent complete re-annotation of the *Arabidopsis thaliana* genome, Araport11, based on the analysis of 113 tissue-specific RNA-Seq data sets (66). In total, 17 Nt-peptides matched such Araport11 protein-coding gene models, often representing new splice variants missing in TAIR10. Further manual inspection demonstrated various alternative proteoforms such as; Nt-extensions encoded by the first exon (Fig. 5*A*), novel upstream exons (Fig. 5*B*), start and an extension of the second exon (Fig. 5*C*) and alternative or wrongly annotated splicing events (Fig. 5*D*). Note that such splice site over-spanning Nt-peptides would be missed if solely searching a 6-FT database, though could potentially be identified by including Augustus *ab initio* gene predictions (Fig. 2).

In contrast to our previous studies integrating ribo-seq and N-terminomics data (19–21, 77), the customized database was based on 6-FT genome translation and *ab initio* gene prediction. Thus, ribosome profiling data served as *a posteriori* confirmation of our proteogenomic identifications. In total, there was translational evidence supporting 23 protein starts, including translation of transposable elements, protein Nt-extensions and the presence of novel exons (Fig. 6, supplemental Data Set S3). Based on the prediction of domain structures, some of these confirmed extensions could reveal direct functional evidence. Increased protein-level support for the novel Nt-peptides increased the proportion of ribo-seq validated TIS (Table II). For instance, 18 out of 33 Nt-peptides (55%) matching Protein Entrez entries had ribo-seq coverage, whereas for Araport11 matching Nt-peptides, this was 10 out of 17 (59%; Table II). Note that also in a four cases, ribo-seq evidence was found for Nt-peptides without a predicted gene model. For instance, we observed translation from the near-cognate start codon ‘GUG’ upstream of the SDF2 protein (Fig. 6*C*). Furthermore, it was evolutionary conserved in *Brassicaceae* and had a favorable TIS nucleotide context (score 195, supplemental Data Set S3). Next to SDF2, an alternative start was also found for NUCLEOSOME ASSEMBLY PROTEIN1 from the near-cognate start codon CUG, otherwise giving rise to Leu in the TAIR10 annotated protein. Note that standard TIS identification based on ribosome profiling data requires matching transcript annotation, meaning that such TIS would remain unnoticed (8).

Proteogenomics is an emerging research field yet lacking a uniform method of analysis and with various strategies having their unique strengths and weaknesses (15, 82). Here, we demonstrate some unique strengths and opportunities using Nt-proteomics data for proteogenomics. Next to applying a FDR threshold, additional filtering criteria next to available meta-data can be used to prioritize novel N termini. Similar to the TAIR10 annotated search results, the majority of novel peptides were *in vivo*/*in vitro* labeled by an acetyl group (158 out of 208 peptides). Besides this, other protein Nt-modifications such as NME, predicted by tools such as Termi*N*ator3 (46, 50), serve as other effective filters to discriminate true positives viewing the > 99% prediction accuracy of NME in previous (65) and our TAIR10 search results. Initially, the predictive tool was already shown to achieve an accuracy of more than 95% for Arabidopsis experimental data (50). In total, 77% (122/158) of the Nt-peptides fulfilled the NME specificity rules, a number lower than that of the initial TAIR10 searches, again highlighting the challenge to discriminate true positives in proteogenomic analysis (2). Next to interesting features for data interpretation and analysis, Nt-proteomics is also interesting for the detection of low abundant proteins. Nt-COFRADIC for instance, leads to less complex peptide mixtures to be analyzed, which are further analyzed over several LC-MS/MS runs (32, 60). Thus, at the expense of increased LC-MS/MS time, this will facilitate the detection of low abundant proteins or modified forms thereof.

Also at a nucleotide level, Nt-data provides specific analysis opportunities. Non-annotated downstream ‘AUG’ start codons were demonstrated before to be under negative selection (83). When consulting genome alignments of *A. thaliana* to *A. lyrata* and *B. rapa*, 40 TIS were shown to maintain a canonical or near-cognate start codon in both *Brassicaceae* species. Furthermore, 28 TIS had the capacity to encode an orthologous Nt-peptide, such as in the case of the N-terminal extension for a 2-oxoglutarate and Fe(II)-dependent oxygenase (Fig. 7). Next to conservation analysis, the TIS nucleotide context was also investigated as this is an important determinant for the efficiency of translation initiation (73–75). Adenine residues were prevalent in the 5 nucleotides preceding the start codons of novel Nt-peptides (Fig. 8), a feature shown to promote efficient translation initiation (75). Using a TIS context scoring method (57), we demonstrated that the 28 conserved TIS scored similarly as the annotated TAIR10 transcripts (Fig. 8). Of these 28 evolutionary-conserved TIS, 19 had additional supportive meta-data, of which 9 ribo-seq evidence (Table II). This suggests that TIS context conservation can aid in prioritizing novel Nt-peptides.

To improve the number of peptide identifications, we combined the results of three search engines; COMET (36), Crux (37), and MS-GF+ (38). Despite the availability of several tools to combine search results (84), aggregating results from database searches using semi-specific protease settings with user-specified Nt-modifications proved to be a difficult task and is sometimes limited to individual search algorithms. Therefore, we implemented the FDR score algorithm described by Jones *et al.*, which uses the FDR as a search engine-independent scoring system (40). Note that the FDR score was used before in a proteogenomic study to combine results of two customized database searches (30). Of the search tools themselves, MS-GF+ performed best (Fig. 3), which was anticipated given its applicability to diverse types of spectral data and experimental protocols (38) and its earlier reported performance on Nt proteome data (85, 86). The fact that the customized database search was performed on TAIR10 unidentified spectra, caused the FDR to be estimated on the subset of novel Nt-peptides. Note that when applying a less stringent global FDR, considerably more identified peptides would be retrieved. In comparison to the TAIR10 searches, more stringent selection criteria were used for novel peptides by requiring peptides to be identified by at least two search engines. As an additional confidence measure, the correlation between theoretical and observed spectra, for TAIR10 and novel peptides, were computed by the MS^2^PIP server (49). Inspecting the novel peptides with a higher or equal correlation than the median correlation of TAIR10 spectra learns this to be a valuable threshold. For instance only for two of the resulting 65 MS^2^PIP supported spectra, no additional metadata was found. Furthermore, 18 out of 23 ribo-seq (78%) supported TIS had a corresponding PSM with high Pearson correlation (Table II, supplemental Data Set S3). Taken together, this suggests MS^2^PIP prediction to be a useful additional asset for confidence estimation of proteogenomic peptides.

Finally, to test the performance of our proteogenomic approach for unannotated species, we performed an *ab initio* MS/MS search, assuming we only have the genome sequence of Arabidopsis available. We used MS-GF+ to search all MS/MS spectra derived from the trypsin-digested proteome (Fig. 1*A*) against 1.2 million full-length Nt-peptides resulting from the 6-FT from canonical start codons with a minimal ORF length of 8 amino acids. Unlike our proteogenomic peptide libraries, we did not exclude peptide sequences matching TAIR10 proteins that were otherwise filtered out (Fig. 2). This is important as it allows us to assess the recovery of TAIR10 protein sequences. In total, 2368 peptides were identified (peptide Q-value < 0.5%, supplemental Fig. S8) of which 1653 (70%) matched a TAIR10 protein start (position 1 or 2). In comparison, this represents up to 61% of protein starts identified in a TAIR10 semi-digested search (MS-GF+, peptide Q-value < 0.5%). In addition, 272 (11%) peptides were not identified using TAIR10 as database search space. Of these, 24 (8.8%) peptides were present in the Protein Entrez database and some had complementary ribo-seq evidence as described earlier. Thus, in total ∼90% of all *ab initio* identified peptides successfully recovered the database annotated N termini of TAIR10 proteins. Although we do not advise such search to replace routine searches for well-annotated species, it could serve as a valuable starting point for proteome characterization in unannotated species. Similarly, ribosome profiling represents a powerful and complementary approach to improve genome annotation (87) with both techniques serving as direct sources of protein synthesis evidence for unannotated organisms.

## DATA AVAILABILITY

All mass spectrometry proteomics data and search results have been deposited to the ProteomeXchange Consortium via the PRIDE (41) partner repository with the data set identifier PXD004896 and project name “N-terminomics Proteogenomics” (http://www.ebi.ac.uk/pride/archive/projects/PXD004896). Ribo-seq sequencing data have been deposited in NCBI's Gene Expression Omnibus and are accessible through GEO Series accession GSE88790 (https://www.ncbi.nlm.nih.gov/geo/query/acc.cgi?acc=GSE88790).

## Supplementary Material

Supplemental Data

## Footnotes

^1^ The abbreviations used are:

Nt: N-terminal
6-FT: six-frame translation
COFRADIC: combined fractional diagonal chromatography
EMBOSS: European molecular biology open software suite
FDR: false discovery rate
HARR: harringtonine
IGV: integrative genome viewer
iMet: initiator Methionine
LTM: lactimidomycin
NME: N-terminal methionine excision
PCV: packed cell volume
PSM: peptide-to-spectrum match
SCX: strong cation exchange
TAILS: terminal amine isotopic labeling of substrates
TIS: translation initiation site
uORF: upstream ORF.

## References

[1] Jaffe J. D., Berg H. C., and Church G. M. (2004) Proteogenomic mapping as a complementary method to perform genome annotation. Proteomics 4, 59–77 14730672 10.1002/pmic.200300511

[2] Nesvizhskii A. I. (2014) Proteogenomics: concepts, applications and computational strategies. Nat. Methods 11, 1114–1125 25357241 10.1038/nmeth.3144 PMC4392723

[3] Smith L. M., Kelleher N. L., and Consortium for Top Down Proteomics (2013) Proteoform: a single term describing protein complexity. Nat. Methods 10, 186–187 23443629 10.1038/nmeth.2369 PMC4114032

[4] Zhang B., Wang J., Wang X., Zhu J., Liu Q., Shi Z., Chambers M. C., Zimmerman L. J., Shaddox K. F., Kim S., Davies S. R., Wang S., Wang P., Kinsinger C. R., Rivers R. C., Rodriguez H., Townsend R. R., Ellis M. J., Carr S. A., Tabb D. L., Coffey R. J., Slebos R. J., Liebler D. C., and NCI CPTAC (2014) Proteogenomic characterization of human colon and rectal cancer. Nature 513, 382–387 25043054 10.1038/nature13438 PMC4249766

[5] Ruggles K. V., Tang Z., Wang X., Grover H., Askenazi M., Teubl J., Cao S., McLellan M. D., Clauser K. R., Tabb D. L., Mertins P., Slebos R., Erdmann-Gilmore P., Li S., Gunawardena H. P., Xie L., Liu T., Zhou J. Y., Sun S., Hoadley K. A., Perou C. M., Chen X., Davies S. R., Maher C. A., Kinsinger C. R., Rodland K. D., Zhang H., Zhang Z., Ding L., Townsend R. R., Rodriguez H., Chan D., Smith R. D., Liebler D. C., Carr S. A., Payne S., Ellis M. J., and Fenyo D. (2016) An analysis of the sensitivity of proteogenomic mapping of somatic mutations and novel splicing events in cancer. Mol. Cell. Proteomics 15, 1060–1071 26631509 10.1074/mcp.M115.056226 PMC4813688

[6] Cesnik A. J., Shortreed M. R., Sheynkman G. M., Frey B. L., and Smith L. M. (2016) Human proteomic variation revealed by combining RNA-Seq proteogenomics and global post-translational modification (G-PTM) search strategy. J. Proteome Res. 15, 800–808 26704769 10.1021/acs.jproteome.5b00817 PMC4779408

[7] Gawron D., Gevaert K., and Van Damme P. (2014) The proteome under translational control. Proteomics 14, 2647–2662 25263132 10.1002/pmic.201400165

[8] Crappe J., Ndah E., Koch A., Steyaert S., Gawron D., De Keulenaer S., De Meester E., De Meyer T., Van Criekinge W., Van Damme P., and Menschaert G. (2015) PROTEOFORMER: deep proteome coverage through ribosome profiling and MS integration. Nucleic Acids Res. 43, e29 25510491 10.1093/nar/gku1283 PMC4357689

[9] Li H. D., Menon R., Omenn G. S., and Guan Y. (2014) Revisiting the identification of canonical splice isoforms through integration of functional genomics and proteomics evidence. Proteomics 14, 2709–2718 25265570 10.1002/pmic.201400170 PMC4372202

[10] Feng Y., Chien K. Y., Chen H. L., and Chiu C. H. (2012) Pseudogene recoding revealed from proteomic analysis of salmonella serovars. J. Proteome Res. 11, 1715–1719 22296100 10.1021/pr200904c

[11] Zhang K., Fu Y., Zeng W. F., He K., Chi H., Liu C., Li Y. C., Gao Y., Xu P., and He S. M. (2015) A note on the false discovery rate of novel peptides in proteogenomics. Bioinformatics 31, 3249–3253 26076724 10.1093/bioinformatics/btv340 PMC4595894

[12] Castellana N. E., Payne S. H., Shen Z., Stanke M., Bafna V., and Briggs S. P. (2008) Discovery and revision of Arabidopsis genes by proteogenomics. Proc. Natl. Acad. Sci. U.S.A. 105, 21034–21038 19098097 10.1073/pnas.0811066106 PMC2605632

[13] Baerenfaller K., Grossmann J., Grobei M. A., Hull R., Hirsch-Hoffmann M., Yalovsky S., Zimmermann P., Grossniklaus U., Gruissem W., and Baginsky S. (2008) Genome-scale proteomics reveals Arabidopsis thaliana gene models and proteome dynamics. Science 320, 938–941 18436743 10.1126/science.1157956

[14] Lamesch P., Berardini T. Z., Li D., Swarbreck D., Wilks C., Sasidharan R., Muller R., Dreher K., Alexander D. L., Garcia-Hernandez M., Karthikeyan A. S., Lee C. H., Nelson W. D., Ploetz L., Singh S., Wensel A., and Huala E. (2012) The Arabidopsis Information Resource (TAIR): improved gene annotation and new tools. Nucleic Acids Res. 40, D1202–D1210 22140109 10.1093/nar/gkr1090 PMC3245047

[15] Menschaert G., and Fenyo D. (2015) Proteogenomics from a bioinformatics angle: A growing field. Mass Spectrom. Rev. 9999, 1–16 10.1002/mas.21483 PMC6101030 26670565

[16] Lee S., Liu B., Lee S., Huang S. X., Shen B., and Qian S. B. (2012) Global mapping of translation initiation sites in mammalian cells at single-nucleotide resolution. Proc. Natl. Acad. Sci. U.S.A. 109, E2424–E2432 22927429 10.1073/pnas.1207846109 PMC3443142

[17] Ingolia N. T., Lareau L. F., and Weissman J. S. (2011) Ribosome profiling of mouse embryonic stem cells reveals the complexity and dynamics of mammalian proteomes. Cell 147, 789–802 22056041 10.1016/j.cell.2011.10.002 PMC3225288

[18] Raj A., Wang S. H., Shim H., Harpak A., Li Y. I., Engelmann B., Stephens M., Gilad Y., and Pritchard J. K. (2016) Thousands of novel translated open reading frames in humans inferred by ribosome footprint profiling. Elife 5, e13328 27232982 10.7554/eLife.13328 PMC4940163

[19] Menschaert G., Van Criekinge W., Notelaers T., Koch A., Crappe J., Gevaert K., and Van Damme P. (2013) Deep proteome coverage based on ribosome profiling aids mass spectrometry-based protein and peptide discovery and provides evidence of alternative translation products and near-cognate translation initiation events. Mol. Cell. Proteomics 12, 1780–1790 23429522 10.1074/mcp.M113.027540 PMC3708165

[20] Koch A., Gawron D., Steyaert S., Ndah E., Crappe J., De Keulenaer S., De Meester E., Ma M., Shen B., Gevaert K., Van Criekinge W., Van Damme P., and Menschaert G. (2014) A proteogenomics approach integrating proteomics and ribosome profiling increases the efficiency of protein identification and enables the discovery of alternative translation start sites. Proteomics 14, 2688–2698 25156699 10.1002/pmic.201400180 PMC4391000

[21] Gawron D., Ndah E., Gevaert K., and Van Damme P. (2016) Positional proteomics reveals differences in N-terminal proteoform stability. Mol. Syst. Biol. 12, 858 26893308 10.15252/msb.20156662 PMC4770386

[22] Slavoff S. A., Mitchell A. J., Schwaid A. G., Cabili M. N., Ma J., Levin J. Z., Karger A. D., Budnik B. A., Rinn J. L., and Saghatelian A. (2013) Peptidomic discovery of short open reading frame-encoded peptides in human cells. Nat. Chem. Biol. 9, 59–64 23160002 10.1038/nchembio.1120 PMC3625679

[23] Ma J., Diedrich J. K., Jungreis I., Donaldson C., Vaughan J., Kellis M., Yates J. R. 3rd, and Saghatelian A. (2016) Improved identification and analysis of small open reading frame encoded polypeptides. Anal. Chem. 88, 3967–3975 27010111 10.1021/acs.analchem.6b00191 PMC4939623

[24] Vu L. D., Stes E., Van Bel M., Nelissen H., Maddelein D., Inze D., Coppens F., Martens L., Gevaert K., and De Smet I. (2016) Up-to-date workflow for plant (phospho)proteomics identifies differential drought-responsive phosphorylation events in maize leaves. J. Proteome Res. 15, 4304–4317 27643528 10.1021/acs.jproteome.6b00348

[25] Baudet M., Ortet P., Gaillard J. C., Fernandez B., Guerin P., Enjalbal C., Subra G., de Groot A., Barakat M., Dedieu A., and Armengaud J. (2010) Proteomics-based refinement of Deinococcus deserti genome annotation reveals an unwonted use of non-canonical translation initiation codons. Mol. Cell. Proteomics 9, 415–426 19875382 10.1074/mcp.M900359-MCP200 PMC2830850

[26] Bland C., Hartmann E. M., Christie-Oleza J. A., Fernandez B., and Armengaud J. (2014) N-Terminal-oriented proteogenomics of the marine bacterium roseobacter denitrificans Och114 using N-Succinimidyloxycarbonylmethyl)tris(2,4,6-trimethoxyphenyl)phosphonium bromide (TMPP) labeling and diagonal chromatography. Mol. Cell. Proteomics 13, 1369–1381 24536027 10.1074/mcp.O113.032854 PMC4014292

[27] Gallien S., Perrodou E., Carapito C., Deshayes C., Reyrat J. M., Van Dorsselaer A., Poch O., Schaeffer C., and Lecompte O. (2009) Ortho-proteogenomics: multiple proteomes investigation through orthology and a new MS-based protocol. Genome Res. 19, 128–135 18955433 10.1101/gr.081901.108 PMC2612966

[28] Yamazaki S., Yamazaki J., Nishijima K., Otsuka R., Mise M., Ishikawa H., Sasaki K., Tago S., and Isono K. (2006) Proteome analysis of an aerobic hyperthermophilic crenarchaeon, Aeropyrum pernix K1. Mol. Cell. Proteomics 5, 811–823 16455681 10.1074/mcp.M500312-MCP200

[29] Aivaliotis M., Gevaert K., Falb M., Tebbe A., Konstantinidis K., Bisle B., Klein C., Martens L., Staes A., Timmerman E., Van Damme J., Siedler F., Pfeiffer F., Vandekerckhove J., and Oesterhelt D. (2007) Large-scale identification of N-terminal peptides in the halophilic archaea Halobacterium salinarum and Natronomonas pharaonis. J. Proteome Res. 6, 2195–2204 17444671 10.1021/pr0700347

[30] Blakeley P., Overton I. M., and Hubbard S. J. (2012) Addressing statistical biases in nucleotide-derived protein databases for proteogenomic search strategies. J. Proteome Res. 11, 5221–5234 23025403 10.1021/pr300411q PMC3703792

[31] Van Leene J., Stals H., Eeckhout D., Persiau G., Van De Slijke E., Van Isterdael G., De Clercq A., Bonnet E., Laukens K., Remmerie N., Henderickx K., De Vijlder T., Abdelkrim A., Pharazyn A., Van Onckelen H., Inze D., Witters E., and De Jaeger G. (2007) A tandem affinity purification-based technology platform to study the cell cycle interactome in Arabidopsis thaliana. Mol. Cell. Proteomics 6, 1226–1238 17426018 10.1074/mcp.M700078-MCP200

[32] Staes A., Impens F., Van Damme P., Ruttens B., Goethals M., Demol H., Timmerman E., Vandekerckhove J., and Gevaert K. (2011) Selecting protein N-terminal peptides by combined fractional diagonal chromatography. Nat. Protoc. 6, 1130–1141 21799483 10.1038/nprot.2011.355

[33] Van Damme P., Van Damme J., Demol H., Staes A., Vandekerckhove J., and Gevaert K. (2009) A review of COFRADIC techniques targeting protein N-terminal acetylation. BMC Proceedings 3, S6 10.1186/1753-6561-3-S6-S6 PMC2722099 19660099

[34] Arnesen T., Van Damme P., Polevoda B., Helsens K., Evjenth R., Colaert N., Varhaug J. E., Vandekerckhove J., Lillehaug J. R., Sherman F., and Gevaert K. (2009) Proteomics analyses reveal the evolutionary conservation and divergence of N-terminal acetyltransferases from yeast and humans. Proc. Natl. Acad. Sci. U.S.A. 106, 8157–8162 19420222 10.1073/pnas.0901931106 PMC2688859

[35] Van Damme P., Hole K., Pimenta-Marques A., Helsens K., Vandekerckhove J., Martinho R. G., Gevaert K., and Arnesen T. (2011) NatF contributes to an evolutionary shift in protein N-terminal acetylation and is important for normal chromosome segregation. PLoS Genet. 7, e1002169 21750686 10.1371/journal.pgen.1002169 PMC3131286

[36] Eng J. K., Jahan T. A., and Hoopmann M. R. (2013) Comet: an open-source MS/MS sequence database search tool. Proteomics 13, 22–24 23148064 10.1002/pmic.201200439

[37] Park C. Y., Klammer A. A., Kall L., MacCoss M. J., and Noble W. S. (2008) Rapid and accurate peptide identification from tandem mass spectra. J. Proteome Res. 7, 3022–3027 18505281 10.1021/pr800127y PMC2667385

[38] Kim S., and Pevzner P. A. (2014) MS-GF+ makes progress towards a universal database search tool for proteomics. Nat. Commun. 5, 5277 25358478 10.1038/ncomms6277 PMC5036525

[39] Elias J. E., and Gygi S. P. (2007) Target-decoy search strategy for increased confidence in large-scale protein identifications by mass spectrometry. Nat. Methods 4, 207–214 17327847 10.1038/nmeth1019

[40] Jones A. R., Siepen J. A., Hubbard S. J., and Paton N. W. (2009) Improving sensitivity in proteome studies by analysis of false discovery rates for multiple search engines. Proteomics 9, 1220–1229 19253293 10.1002/pmic.200800473 PMC2899855

[41] Vizcaino J. A., Csordas A., del-Toro N., Dianes J. A., Griss J., Lavidas I., Mayer G., Perez-Riverol Y., Reisinger F., Ternent T., Xu Q. W., Wang R., and Hermjakob H. (2016) 2016 update of the PRIDE database and its related tools. Nucleic Acids Res. 44, D447–D456 26527722 10.1093/nar/gkv1145 PMC4702828

[42] Rice P., Longden I., and Bleasby A. (2000) EMBOSS: the European Molecular Biology Open Software Suite. Trends Genet. 16, 276–277 10827456 10.1016/s0168-9525(00)02024-2

[43] Wamboldt Y., Mohammed S., Elowsky C., Wittgren C., de Paula W. B., and Mackenzie S. A. (2009) Participation of leaky ribosome scanning in protein dual targeting by alternative translation initiation in higher plants. Plant Cell 21, 157–167 19182105 10.1105/tpc.108.063644 PMC2648075

[44] Stanke M., Keller O., Gunduz I., Hayes A., Waack S., and Morgenstern B. (2006) AUGUSTUS: ab initio prediction of alternative transcripts. Nucleic Acids Res. 34, W435–W439 16845043 10.1093/nar/gkl200 PMC1538822

[45] Stanke M., and Waack S. (2003) Gene prediction with a hidden Markov model and a new intron submodel. Bioinformatics 19, ii215–ii225 14534192 10.1093/bioinformatics/btg1080

[46] Frottin F., Martinez A., Peynot P., Mitra S., Holz R. C., Giglione C., and Meinnel T. (2006) The proteomics of N-terminal methionine cleavage. Mol. Cell. Proteomics 5, 2336–2349 16963780 10.1074/mcp.M600225-MCP200

[47] Ju J., Lim S. K., Jiang H., Seo J. W., and Shen B. (2005) Iso-migrastatin congeners from Streptomyces platensis and generation of a glutarimide polyketide library featuring the dorrigocin, lactimidomycin, migrastatin, and NK30424 scaffolds. J. Am. Chem. Soc. 127, 11930–11931 16117518 10.1021/ja053118u

[48] Schneider-Poetsch T., Ju J., Eyler D. E., Dang Y., Bhat S., Merrick W. C., Green R., Shen B., and Liu J. O. (2010) Inhibition of eukaryotic translation elongation by cycloheximide and lactimidomycin. Nat. Chem. Biol. 6, 209–217 20118940 10.1038/nchembio.304 PMC2831214

[49] Degroeve S., Maddelein D., and Martens L. (2015) MS2PIP prediction server: compute and visualize MS2 peak intensity predictions for CID and HCD fragmentation. Nucleic Acids Res. 43, W326–W330 25990723 10.1093/nar/gkv542 PMC4489309

[50] Martinez A., Traverso J. A., Valot B., Ferro M., Espagne C., Ephritikhine G., Zivy M., Giglione C., and Meinnel T. (2008) Extent of N-terminal modifications in cytosolic proteins from eukaryotes. Proteomics 8, 2809–2831 18655050 10.1002/pmic.200701191

[51] UniProt Consortium (2015) UniProt: a hub for protein information. Nucleic Acids Res. 43, D204–D212 25348405 10.1093/nar/gku989 PMC4384041

[52] Bairoch A., and Apweiler R. (1996) The SWISS-PROT protein sequence data bank and its new supplement TREMBL. Nucleic Acids Res. 24, 21–25 8594581 10.1093/nar/24.1.21 PMC145613

[53] Clark K., Karsch-Mizrachi I., Lipman D. J., Ostell J., and Sayers E. W. (2016) GenBank. GenBank. Nucleic Acids Res. 44, D67–D72 26590407 10.1093/nar/gkv1276 PMC4702903

[54] Apweiler R., Attwood T. K., Bairoch A., Bateman A., Birney E., Biswas M., Bucher P., Cerutti L., Corpet F., Croning M. D., Durbin R., Falquet L., Fleischmann W., Gouzy J., Hermjakob H., Hulo N., Jonassen I., Kahn D., Kanapin A., Karavidopoulou Y., Lopez R., Marx B., Mulder N. J., Oinn T. M., Pagni M., Servant F., Sigrist C. J., Zdobnov E. M., and InterPro Consortium (2000) InterPro–an integrated documentation resource for protein families, domains and functional sites. Bioinformatics 16, 1145–1150 11159333 10.1093/bioinformatics/16.12.1145

[55] Robinson J. T., Thorvaldsdottir H., Winckler W., Guttman M., Lander E. S., Getz G., and Mesirov J. P. (2011) Integrative genomics viewer. Nat. Biotechnol. 29, 24–26 21221095 10.1038/nbt.1754 PMC3346182

[56] Crooks G. E., Hon G., Chandonia J. M., and Brenner S. E. (2004) WebLogo: a sequence logo generator. Genome Res. 14, 1188–1190 15173120 10.1101/gr.849004 PMC419797

[57] Grzegorski S. J., Chiari E. F., Robbins A., Kish P. E., and Kahana A. (2014) Natural variability of Kozak sequences correlates with function in a zebrafish model. PLoS ONE 9, e108475 25248153 10.1371/journal.pone.0108475 PMC4172775

[58] Swaney D. L., Wenger C. D., and Coon J. J. (2010) Value of using multiple proteases for large-scale mass spectrometry-based proteomics. J. Proteome Res. 9, 1323–1329 20113005 10.1021/pr900863u PMC2833215

[59] Vogtle F. N., Wortelkamp S., Zahedi R. P., Becker D., Leidhold C., Gevaert K., Kellermann J., Voos W., Sickmann A., Pfanner N., and Meisinger C. (2009) Global analysis of the mitochondrial N-proteome identifies a processing peptidase critical for protein stability. Cell 139, 428–439 19837041 10.1016/j.cell.2009.07.045

[60] Gevaert K., Goethals M., Martens L., Van Damme J., Staes A., Thomas G. R., and Vandekerckhove J. (2003) Exploring proteomes and analyzing protein processing by mass spectrometric identification of sorted N-terminal peptides. Nat. Biotechnol. 21, 566–569 12665801 10.1038/nbt810

[61] Giansanti P., Tsiatsiani L., Low T. Y., and Heck A. J. (2016) Six alternative proteases for mass spectrometry-based proteomics beyond trypsin. Nat. Protoc. 11, 993–1006 27123950 10.1038/nprot.2016.057

[62] Meinnel T., Peynot P., and Giglione C. (2005) Processed N-termini of mature proteins in higher eukaryotes and their major contribution to dynamic proteomics. Biochimie 87, 701–712 16054524 10.1016/j.biochi.2005.03.011

[63] Staes A., Van Damme P., Helsens K., Demol H., Vandekerckhove J., and Gevaert K. (2008) Improved recovery of proteome-informative, protein N-terminal peptides by combined fractional diagonal chromatography (COFRADIC). Proteomics 8, 1362–1370 18318009 10.1002/pmic.200700950

[64] Van Damme P., Hole K., Pimenta-Marques A., Helsens K., Vandekerckhove J., Martinho R. G., Gevaert K., and Arnesen T. (2011) NatF contributes to an evolutionary shift in protein N-terminal acetylation and is important for normal chromosome segregation. PLoS Genet. 7, e1002169 21750686 10.1371/journal.pgen.1002169 PMC3131286

[65] Bienvenut W. V., Sumpton D., Martinez A., Lilla S., Espagne C., Meinnel T., and Giglione C. (2012) Comparative large scale characterization of plant versus mammal proteins reveals similar and idiosyncratic N-alpha-acetylation features. Mol. Cell. Proteomics 11, M111 015131 10.1074/mcp.M111.015131 PMC3433923 22223895

[66] Krishnakumar V., Hanlon M. R., Contrino S., Ferlanti E. S., Karamycheva S., Kim M., Rosen B. D., Cheng C. Y., Moreira W., Mock S. A., Stubbs J., Sullivan J. M., Krampis K., Miller J. R., Micklem G., Vaughn M., and Town C. D. (2015) Araport: the Arabidopsis information portal. Nucleic Acids Res. 43, D1003–D1009 25414324 10.1093/nar/gku1200 PMC4383980

[67] Hanada K., Higuchi-Takeuchi M., Okamoto M., Yoshizumi T., Shimizu M., Nakaminami K., Nishi R., Ohashi C., Iida K., Tanaka M., Horii Y., Kawashima M., Matsui K., Toyoda T., Shinozaki K., Seki M., and Matsui M. (2013) Small open reading frames associated with morphogenesis are hidden in plant genomes. Proc. Natl. Acad. Sci. U.S.A. 110, 2395–2400 23341627 10.1073/pnas.1213958110 PMC3568369

[68] Gu X. L., Wang H., Huang H., and Cui X. F. (2012) SPT6L encoding a putative WG/GW-repeat protein regulates apical-basal polarity of embryo in Arabidopsis. Mol. Plant 5, 249–259 21948524 10.1093/mp/ssr073

[69] Schott A., Ravaud S., Keller S., Radzimanowski J., Viotti C., Hillmer S., Sinning I., and Strahl S. (2010) Arabidopsis stromal-derived Factor2 (SDF2) is a crucial target of the unfolded protein response in the endoplasmic reticulum. J. Biol. Chem. 285, 18113–18121 20378538 10.1074/jbc.M110.117176 PMC2878572

[70] Nekrasov V., Li J., Batoux M., Roux M., Chu Z. H., Lacombe S., Rougon A., Bittel P., Kiss-Papp M., Chinchilla D., van Esse H. P., Jorda L., Schwessinger B., Nicaise V., Thomma B. P., Molina A., Jones J. D., and Zipfel C. (2009) Control of the pattern-recognition receptor EFR by an ER protein complex in plant immunity. EMBO J. 28, 3428–3438 19763086 10.1038/emboj.2009.262 PMC2776097

[71] Nielsen H., Engelbrecht J., Brunak S., and von Heijne G. (1997) Identification of prokaryotic and eukaryotic signal peptides and prediction of their cleavage sites. Protein Eng. 10, 1–6 10.1093/protein/10.1.1 9051728

[72] Emanuelsson O., Nielsen H., Brunak S., and von Heijne G. (2000) Predicting subcellular localization of proteins based on their N-terminal amino acid sequence. J. Mol. Biol. 300, 1005–1016 10891285 10.1006/jmbi.2000.3903

[73] Kozak M. (1986) Point mutations define a sequence flanking the AUG initiator codon that modulates translation by eukaryotic ribosomes. Cell 44, 283–292 3943125 10.1016/0092-8674(86)90762-2

[74] Kozak M. (1987) An analysis of 5′-noncoding sequences from 699 vertebrate messenger RNAs. Nucleic Acids Res. 15, 8125–8148 3313277 10.1093/nar/15.20.8125 PMC306349

[75] Kim Y., Lee G., Jeon E., Sohn E. J., Lee Y., Kang H., Lee D. W., Kim D. H., and Hwang I. (2014) The immediate upstream region of the 5′-UTR from the AUG start codon has a pronounced effect on the translational efficiency in Arabidopsis thaliana. Nucleic Acids Res. 42, 485–498 24084084 10.1093/nar/gkt864 PMC3874180

[76] O'Leary N. A., Wright M. W., Brister J. R., Ciufo S., Haddad D., McVeigh R., Rajput B., Robbertse B., Smith-White B., Ako-Adjei D., Astashyn A., Badretdin A., Bao Y., Blinkova O., Brover V., Chetvernin V., Choi J., Cox E., Ermolaeva O., Farrell C. M., Goldfarb T., Gupta T., Haft D., Hatcher E., Hlavina W., Joardar V. S., Kodali V. K., Li W., Maglott D., Masterson P., McGarvey K. M., Murphy M. R., O'Neill K., Pujar S., Rangwala S. H., Rausch D., Riddick L. D., Schoch C., Shkeda A., Storz S. S., Sun H., Thibaud-Nissen F., Tolstoy I., Tully R. E., Vatsan A. R., Wallin C., Webb D., Wu W., Landrum M. J., Kimchi A., Tatusova T., DiCuccio M., Kitts P., Murphy T. D., and Pruitt K. D. (2016) Reference sequence (RefSeq) database at NCBI: current status, taxonomic expansion, and functional annotation. Nucleic Acids Res. 44, D733–D745 26553804 10.1093/nar/gkv1189 PMC4702849

[77] Van Damme P., Gawron D., Van Criekinge W., and Menschaert G. (2014) N-terminal proteomics and ribosome profiling provide a comprehensive view of the alternative translation initiation landscape in mice and men. Mol. Cell. Proteomics 13, 1245–1261 24623590 10.1074/mcp.M113.036442 PMC4014282

[78] Helsens K., Van Damme P., Degroeve S., Martens L., Arnesen T., Vandekerckhove J., and Gevaert K. (2011) Bioinformatics analysis of a Saccharomyces cerevisiae N-terminal proteome provides evidence of alternative translation initiation and post-translational N-terminal acetylation. J. Proteome Res. 10, 3578–3589 21619078 10.1021/pr2002325

[79] Eng J. K., Searle B. C., Clauser K. R., and Tabb D. L. (2011) A face in the crowd: recognizing peptides through database search. Mol. Cell. Proteomics 10, R111 009522 10.1074/mcp.R111.009522 PMC3226415 21876205

[80] Lycette B. E., Glickman J. W., Roth S. J., Cram A. E., Kim T. H., Krizanc D., and Weir M. P. (2016) N-Terminal Peptide Detection with Optimized Peptide-Spectrum Matching and Streamlined Sequence Libraries. J. Proteome Res. 15, 2891–2899 27498768 10.1021/acs.jproteome.5b00996 PMC7379158

[81] Venne A. S., Solari F. A., Faden F., Paretti T., Dissmeyer N., and Zahedi R. P. (2015) An improved workflow for quantitative N-terminal charge-based fractional diagonal chromatography (ChaFRADIC) to study proteolytic events in Arabidopsis thaliana. Proteomics 15, 2458–2469 26010716 10.1002/pmic.201500014

[82] Dimitrakopoulos L., Prassas I., Diamandis E. P., Nesvizhskii A., Kislinger T., Jaffe J., and Drabovich A. (2016) Proteogenomics: opportunities and caveats. Clin. Chem. 62, 551–557 26817480 10.1373/clinchem.2015.247858

[83] Bazykin G. A., and Kochetov A. V. (2011) Alternative translation start sites are conserved in eukaryotic genomes. Nucleic Acids Res. 39, 567–577 20864444 10.1093/nar/gkq806 PMC3025576

[84] Shteynberg D., Nesvizhskii A. I., Moritz R. L., and Deutsch E. W. (2013) Combining results of multiple search engines in proteomics. Mol. Cell. Proteomics 12, 2383–2393 23720762 10.1074/mcp.R113.027797 PMC3769318

[85] Lange P. F., Huesgen P. F., Nguyen K., and Overall C. M. (2014) Annotating N termini for the human proteome project: N termini and Nalpha-acetylation status differentiate stable cleaved protein species from degradation remnants in the human erythrocyte proteome. J. Proteome Res. 13, 2028–2044 24555563 10.1021/pr401191w PMC3979129

[86] Fournier C. T., Cherny J. J., Truncali K., Robbins-Pianka A., Lin M. S., Krizanc D., and Weir M. P. (2012) Amino termini of many yeast proteins map to downstream start codons. J. Proteome Res. 11, 5712–5719 23140384 10.1021/pr300538f PMC3519384

[87] Hsu P. Y., Calviello L., Wu H. L., Li F. W., Rothfels C. J., Ohler U., and Benfey P. N. (2016) Super-resolution ribosome profiling reveals unannotated translation events in Arabidopsis. Proc. Natl. Acad. Sci. U.S.A. 113, E7126–E7135 10.1073/pnas.1614788113 PMC5111709 27791167

