# Supplementary material for: N-terminal Proteomics Assisted Profiling of the Unexplored Translation Initiation Landscape in Arabidopsis thaliana
Source: Mol Cell Proteomics. 2017 Apr 21;16(6):1064–80. doi: 10.1074/mcp.M116.066662 (PMC5461538; doi:10.1074/mcp.M116.066662)
Supplement: Supplemental Data [file supp_16_6_1064__index.html]

N-terminal proteomics assisted profiling of the unexplored translation initiation landscape in Arabidopsis thaliana — N-terminal Proteomics Assisted Profiling of the Unexplored Translation Initiation Landscape in Arabidopsis thaliana — N-terminal Proteomics Based Proteogenomics — Supplemental Data 

# N-terminal Proteomics Assisted Profiling of the Unexplored Translation Initiation Landscape in Arabidopsis thaliana

## Supplemental Data

- Supplemental Dataset 3 (.xlsx, 41 KB) - Overview of all meta-data for the 117 novel TIS.
- Supplemental Figures and Tables (.pdf, 1.4 MB) - Supplemental Figures S1-S8 and Supplemental Tables S1-S3.
- Supplemental Dataset 2 (.pdf, 527 KB) - Annotated MS/MS spectra matching novel Nt-peptides
- Supplemental Dataset 1 (.xlsx, 49 KB) - Overview of 169 PSMs identified novel and filtered Nt-peptides.
