# Supplementary material for: N-terminal Proteomics Assisted Profiling of the Unexplored Translation Initiation Landscape in Arabidopsis thaliana
Source: Mol Cell Proteomics. 2017 Apr 21;16(6):1064–80. doi: 10.1074/mcp.M116.066662 (PMC5461538; doi:10.1074/mcp.M116.066662)
Supplement: Supplemental Data [file 10.1074_M116.066662_mcp.M116.066662-2.pdf]

SUPPLEMENTAL MATERIAL

The following supplemental materials are available.

Supplemental Figure S1. Histogram of peptide lengths in the TAIR10 searches.

Supplemental Figure S2. Generation of customized ArgC Nt-peptide library**.**

Supplemental Figure S3. Comparison full- and semi-digested peptide search spaces.

Supplemental Figure S4. TAIR10 identification rates for individual database searching and multiple database search strategy.

Supplemental Figure S5. Missed cleavages in TAIR10 searches.

Supplemental Figure S6. MS^2PIP correlations for spectra matching TAIR10 database annotated and novel N-termini.

Supplemental Figure S7. InterPro sequence search for the 125 amino acid extended version of AT1G63210.1, as indicated by the Nt-peptide 'MNRIDEEPQIHE'.

Supplemental Figure S8. Recovery of TAIR10 proteome using Nt-peptide libraries.

Supplemental Table S1. Identified classes of N-termini.

Supplemental Table S2. Novel TIS identified by different (overlapping) peptide sequences due to the different proteolytic digestions used.

Supplemental Table S3. Allocation of novel TIS to Augustus predicted or Araport11 annotated protein-coding gene models.

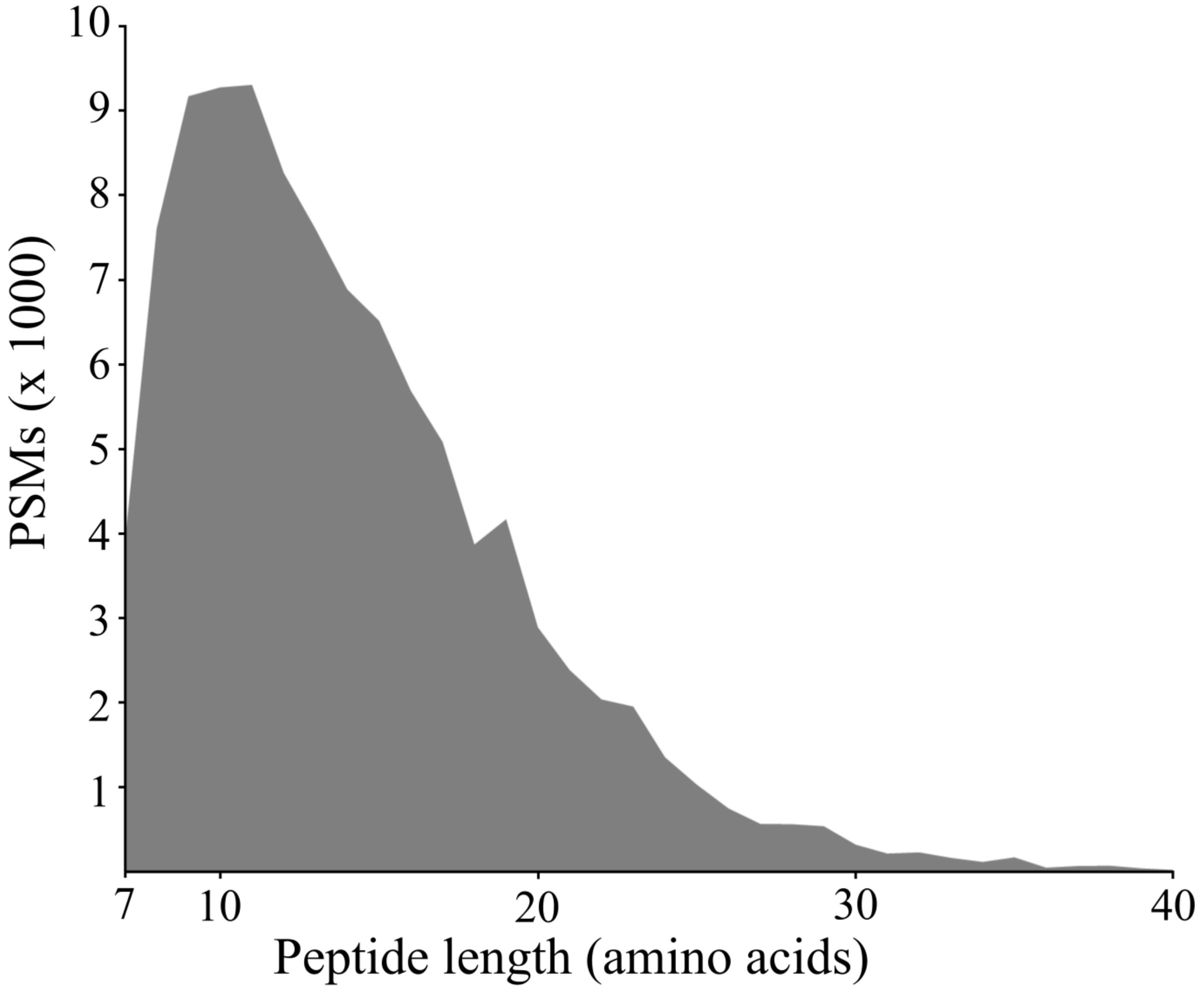

Supplemental Figure S1. Histogram of peptide lengths of PSMs (FDR score ≤ 0.01) in the TAIR10 searches.

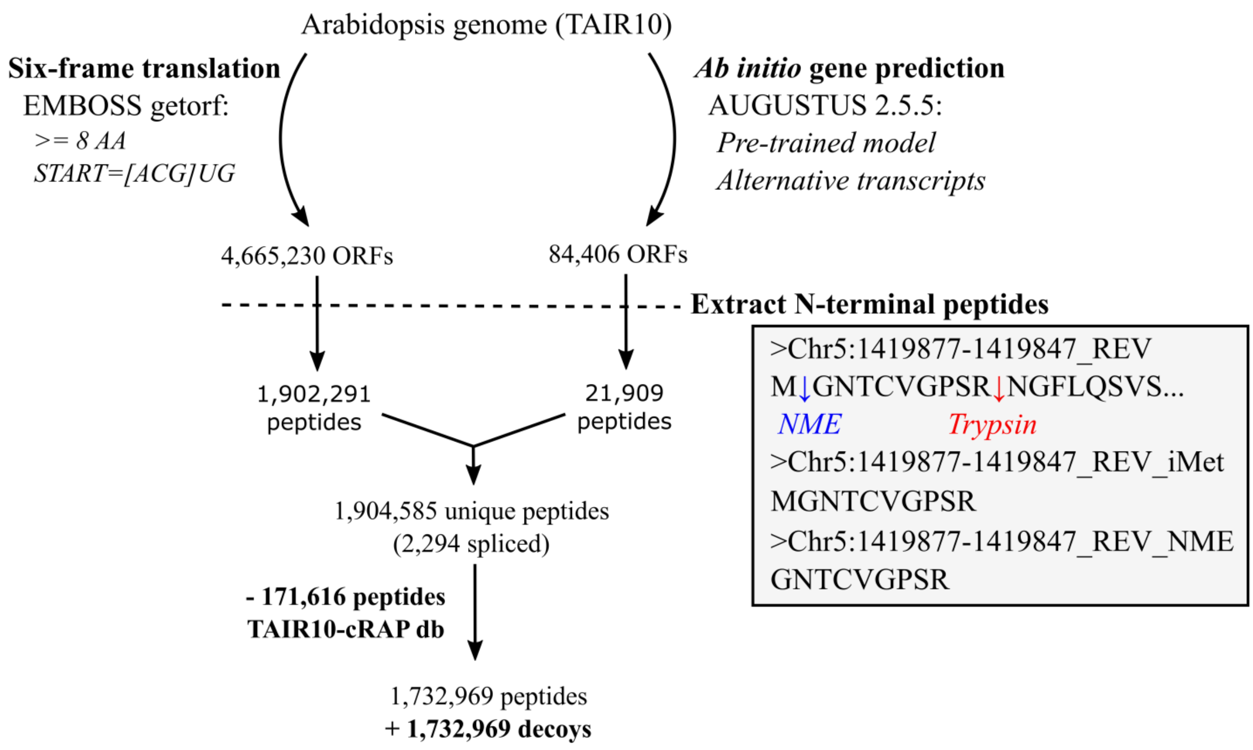

Supplemental Figure S2. Generation of a customized ArgC Nt-peptide library**.** The Arabidopsis genome (TAIR10) was *in silico* translated using the EMBOSS getorf program (42). Next to 6-FT, Augustus (44, 45) was used to predict (spliced) ORFs. From both sources of ORFs, semi-ArgC Nt-peptides were extracted (length 8 – 30AA, trypsin cleavage indicated by red arrow) whenever possible. If the iMet was followed by Ala, Cys, Pro, Ser, Thr or Val, the iMet excised form was additionally considered (black arrow). In total, this resulted in nearly 2 million unique Nt-peptides. Of these, 171,616 peptide sequences corresponded to protein entries in the TAIR10 database and cRAP database (common Repository of Adventitious Proteins, http://www.thegpm.org/crap/) were omitted. This trims down the final peptide library to 1,732,969 semi-ArgC Nt-peptides.

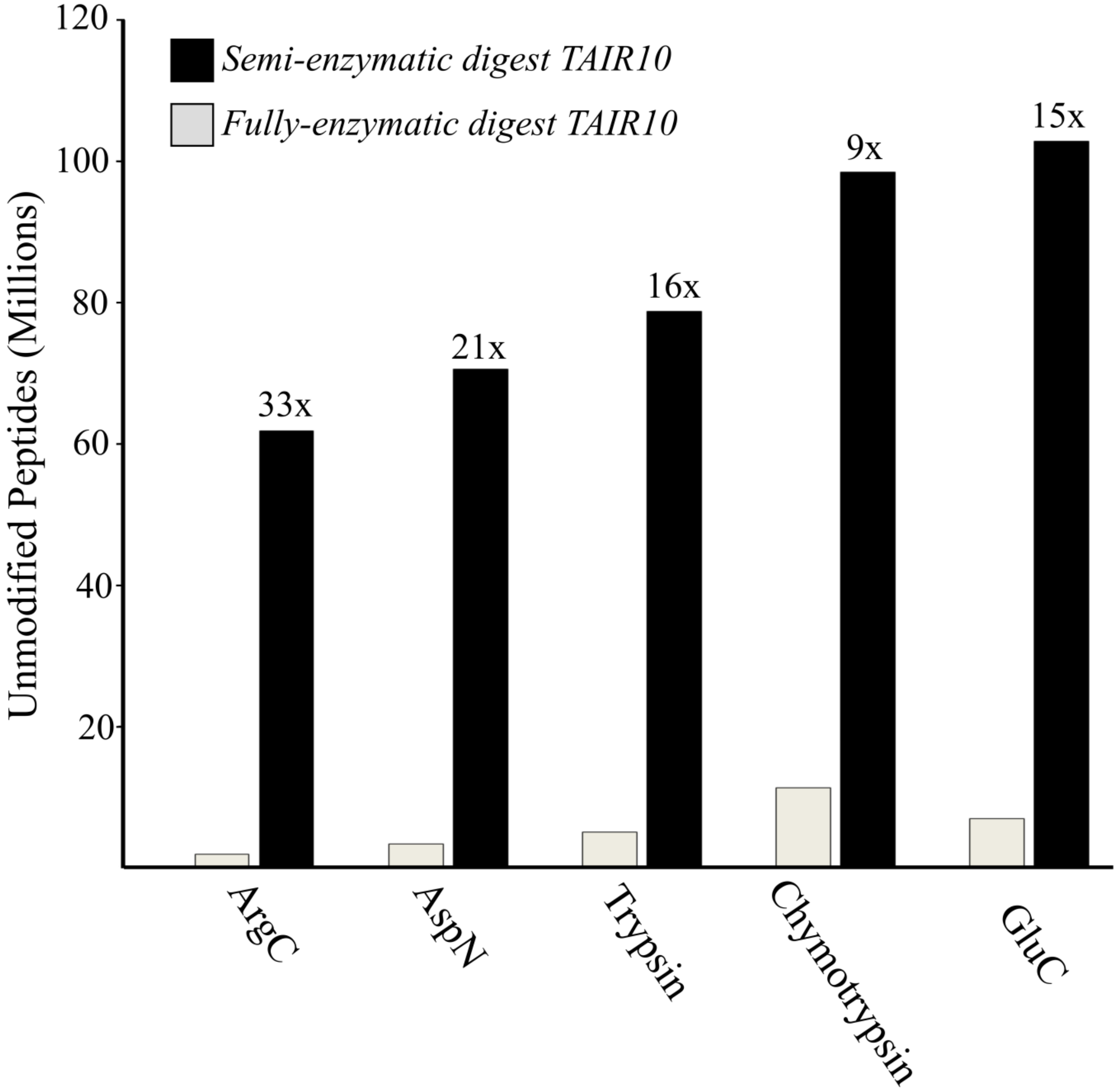

Supplemental Figure S3. The number of unmodified peptides (y-axis) after *in silico* digestion of a reverse target-decoy TAIR10 proteome with full- and semi-enzymatic specificities (grey and black bars respectively, protease specificities see Table 1). The generate-peptides function of the Crux toolkit (37) was used to calculate the number of unmodified peptides. The fold change increase of search space for partial digestion compared to full digestion was indicated. Peptide length was 7 to 40 AA (molecular mass between 600 – 5,500 Da), allowing 2 missed cleavages for Arg-C and trypsin and 3 missed cleavages for AspN, chymotrypsin and GluC.

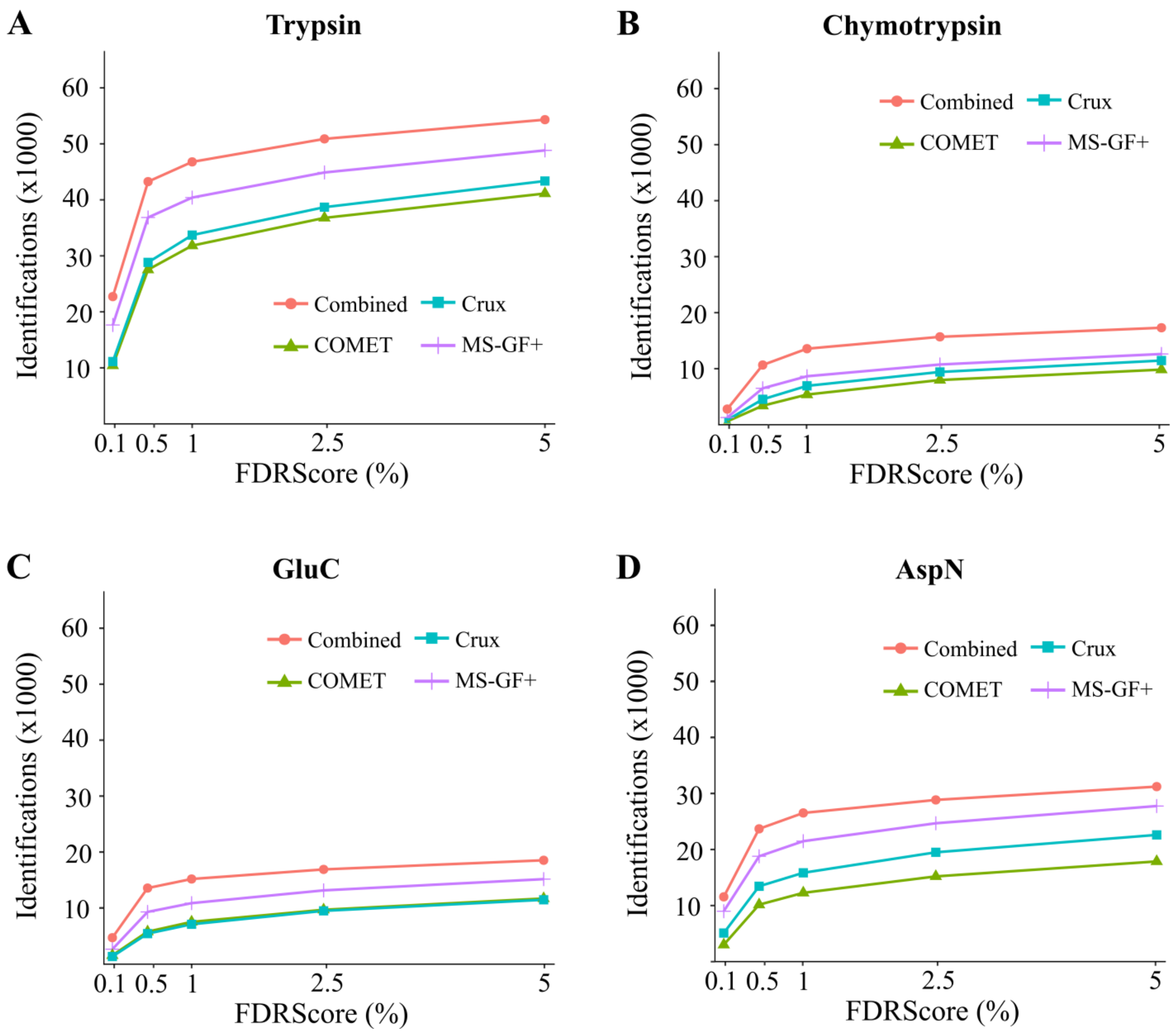

Supplemental Figure S4. Identification rates for (A) trypsin, (B) chymotrypsin, (C) GluC and (D) AspN digested samples plotted against different FDR score thresholds (x-axis, PSM-level). The combined (red dots) and individual search engine results were displayed (see legend).

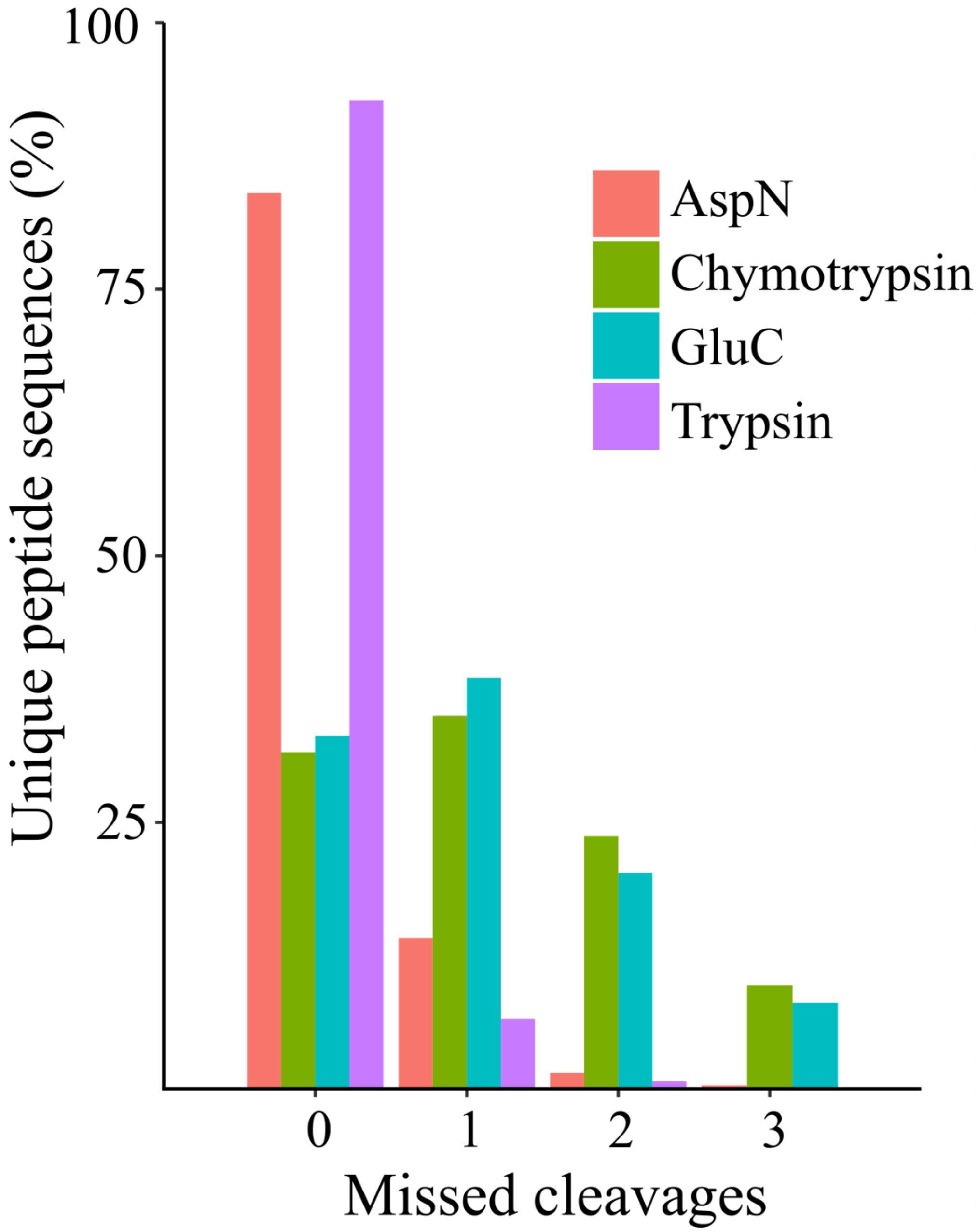

Supplemental Figure S5. Percentage missed cleavages per digestive protease used. The number of uniquely identified peptide sequences (FDR score ≤ 0.01) were displayed and the number of missed cleavages indicated. Note that for the trypsin digested datasets, a semi-ArgC specificity with a maximum of two missed cleavages was used.

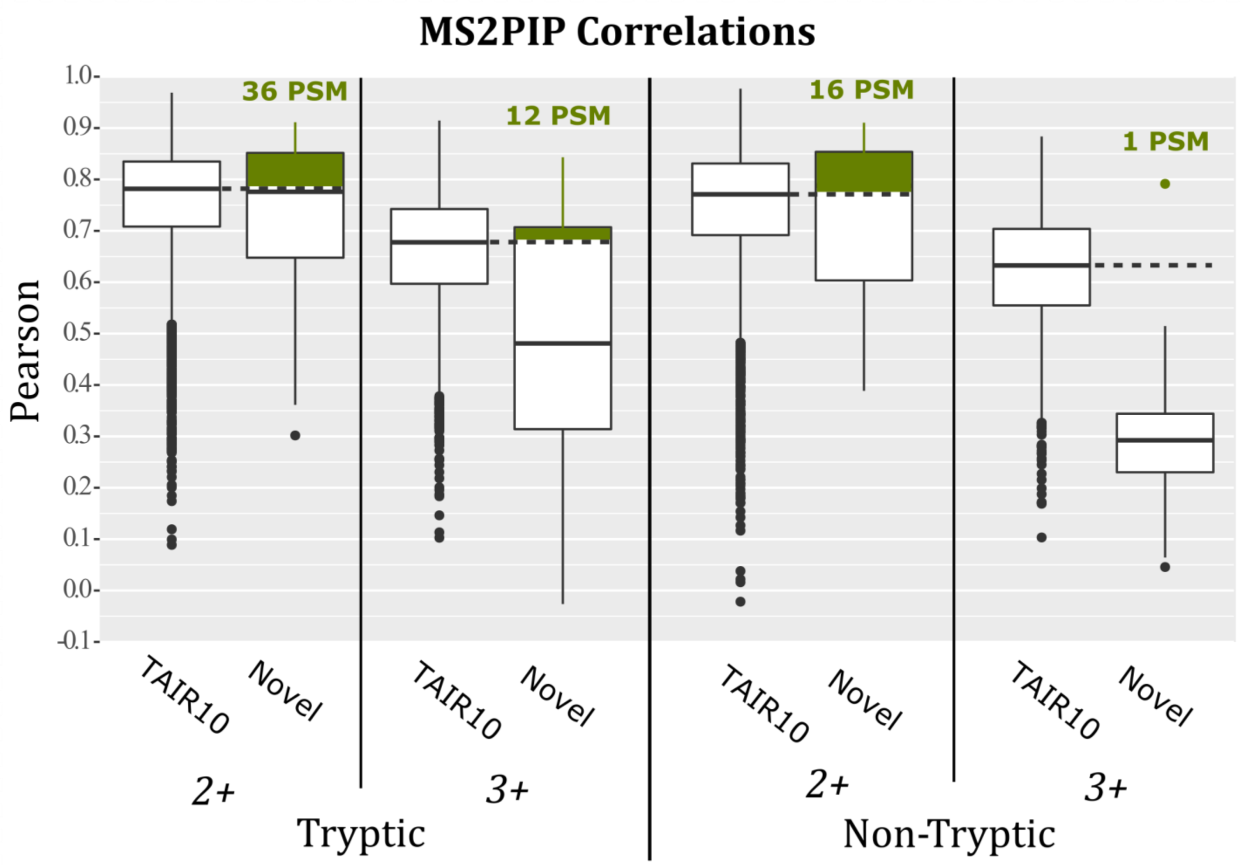

Supplemental Figure S6. MS²PIP correlations for spectra matching TAIR10 database annotated and novel N-termini. The MS²PIP prediction server (49) was used to compute Pearson correlation coefficients of theoretical (CID model) and observed spectra spectra matching TAIR10 database annotated N-termini (position 1 or 2) and the 169 proteogenomic PSMs maching novel Nt-peptide identifications (Supplemental Dataset 1).

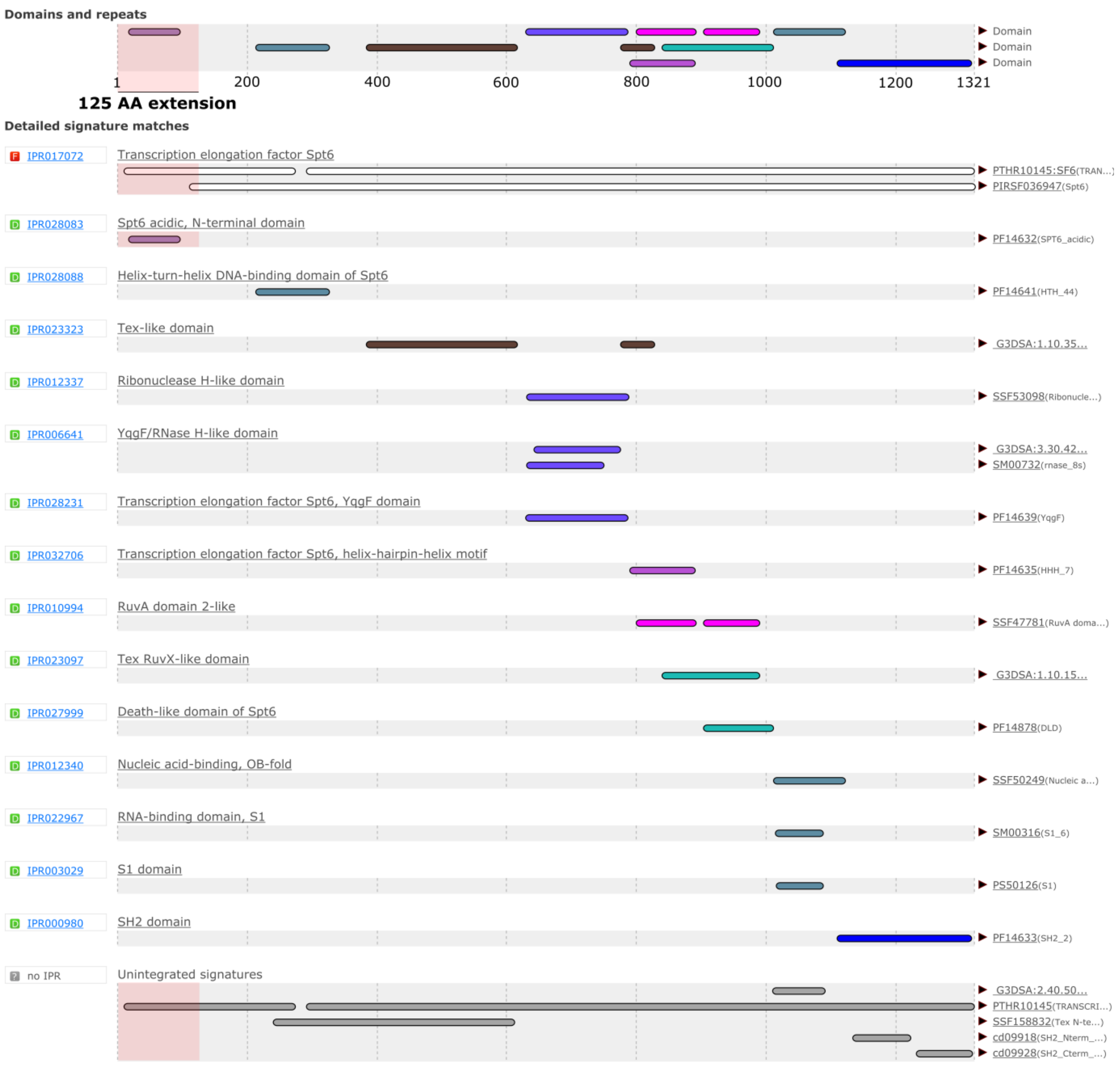

Supplemental Figure S7. InterPro sequence search (54, http://www.ebi.ac.uk/interpro/sequencesearch/) result for the 125 amino acid extended version of AT1G63210.1, starting with the novel Nt-peptide ‘MNRIDEEPQIHE’. Domains and repeats are displayed.

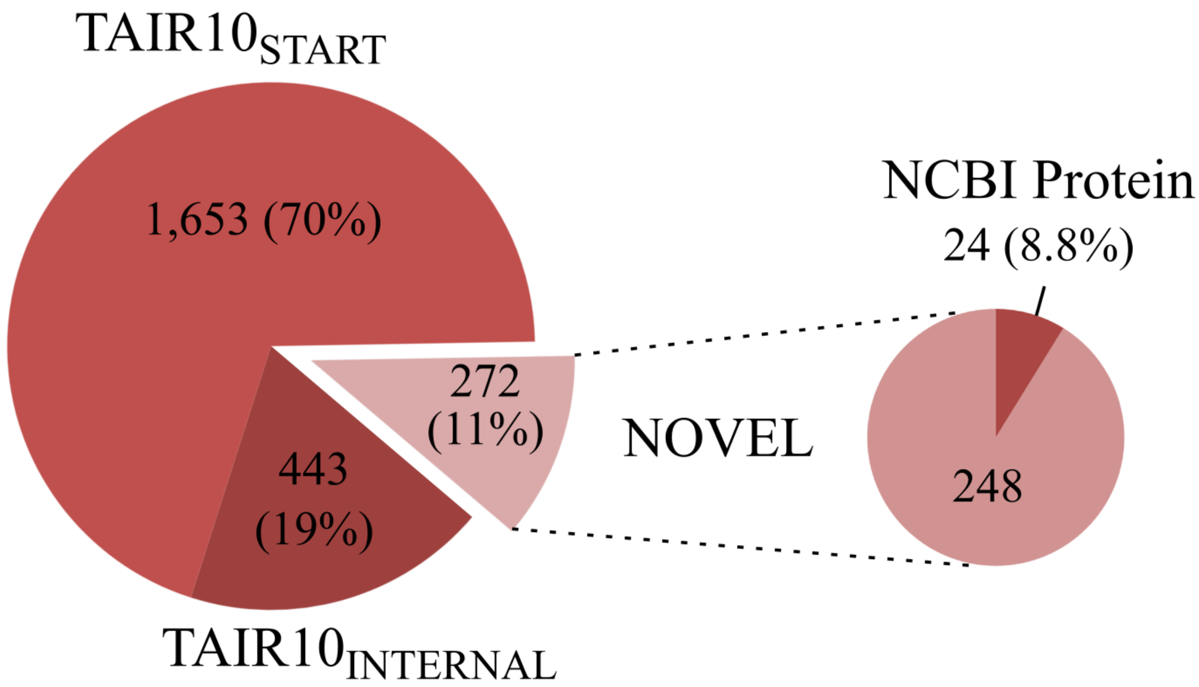

Supplemental Figure S8. Recovery of TAIR10 proteins using a Nt-peptide library derived from a genome 6-FT. MS-GF+ was used to search all trypsin-digested Nt-COFRADIC data against full-length Nt-peptides initiated from canonical start codons encoded in the Arabidopsis genome with a minimal length of 8 AA. Identified peptides were divided according their occurrence and position in TAIR10 proteins. In addition, novel peptides were compared to all Entrez Protein (https://www.ncbi.nlm.nih.gov/protein) records.

Supplemental Table S1. Identified classes of N-termini. All identified N-termini (FDR score < 0.01), for all four proteases, were classified according to their Nt-modification and protein start position. Peptides identified with different Nt-modifications indicative of Nt-proteoform expression (77) are counted as separate instances.

Peptide classes	***Identified spectra (%)***	***Identified peptides (%)***	***Identified proteins (%)***
In vivo acetylated	**19,686 (24.2%)**	**6,401 (14.5%)**	**3,478 (43.6%)**
pos 1	6,040 (7.4%)	1,974 (4.5%)	1,127 (14.1%)
pos 2	10,550 (13%)	2,877 (6.5%)	1,413 (17.7%)
pos > 2	3,096 (3.8%)	1,550 (3.5%)	1,163 (14.6%)
In vitro Ace$^{13}C_2D_3$	**28,677 (35.3%)**	**15,257 (34.6%)**	**4,970 (62.3%)**
pos 1	1,165 (1.4%)	401 (0.9%)	238 (3%)
pos 2	4,335 (5.3%)	1,252 (2.8%)	656 (8.2%)
pos > 2	23,177 (28.5%)	13,604 (30.8%)	4504 (56.5%)
Pyroglutamate	2,151 (2.6%)	1,309 (3%)	985 (12.4%)
Non-Nt-peptide	30,825 (37.9%)	21,136 (47.9%)	4,757 (59.7%)
Total	81,339	44,103	7,972

Supplemental Table S2. Novel TIS identified by different (overlapping) peptide sequences due to the different proteolytic digestions used. For the newly identified TIS the chromosomic start location is indicated with the strand orientation between parentheses (sense [+] and antisense [-]). For each novel TIS the different peptide variants were displayed along with the Nt-modification observed, number of PSMs and the respective digestive protease used.

Genomic location novel TIS / *Peptides*	*Modifications*	*PSMs*	*Protease*
Chr3:21,353,746 (-)			
AHAQTTEGASQVVESVR	Ace (Nt)	3	Trypsin
AHAQTTEGASQVVESVRF	Ace (Nt)	6	Chymotrypsin
Multiple locations (transposable elements)			
ARVKDSSGE	$Ace^{13}C_2D_3$ (Nt, K4)	1	GluC
ARVKDSSGEY	$Ace^{13}C_2D_3$ (Nt, K4)	2	Chymotrypsin
Chr5:3428175 (+)			
GDAAGGLR	Ace (Nt)	2	Trypsin
GDAAGGLRSE	Ace (Nt)	1	GluC
Multiple locations (transposable elements)			
PLSYSSPSSSEER	$Ace^{13}C_2D_3$ (Nt)	4	Trypsin
PLSYSSPSSSEERS	$Ace^{13}C_2D_3$ (Nt)	2	AspN
Chr2:16976780 (+)			
MMKRAQQSARE	Ace (Nt, K3); Oxi (M1,2)	1	GluC
MMKRAQQSARETKQEAK	Ace (Nt, K3,17) ; Oxi (M1,2)	1	AspN

Supplemental Table S3. Allocation of novel TIS to Augustus predicted or Araport11 annotated protein-coding gene models. Augustus (44, 45) gene models were predicted for the customized database generation (Figure 2) and another round of prediction was done while feeding the TIS as extrinsic information. The June 2016 Araport11 annotation was downloaded from the Arabidopsis Information Portal (https://www.araport.org/; 66).

**Novel TIS**	*Augustus*	*Augustus (+hints)*	*Araport11*
58	x	x	x
14	✓	x	x
28	x	✓	x
13	✓	x	✓
4	x	✓	✓
**117**	27	32	17
