# Supplementary material for: N-terminal Proteomics Assisted Profiling of the Unexplored Translation Initiation Landscape in Arabidopsis thaliana
Source: Mol Cell Proteomics. 2017 Apr 21;16(6):1064–80. doi: 10.1074/mcp.M116.066662 (PMC5461538; doi:10.1074/mcp.M116.066662)

Supplemental Dataset 2. Annotated MS/MS spectra matching novel Nt-peptides.

MS/MS spectra matching the 169 novel Nt-peptides were extracted and b/y ions matching the modified peptide sequence were highlighted in blue. Spectra were ordered alphabetically according the peptide sequence. The scan index number is derived from the MGF files (PRIDE accession PXD004896) on which the initial TAIR10 search was performed. Plots were made in R, using the MSnbase package (1).

1. Gatto, L. and Lilley, K.S. (2012) MSnbase-an R/Bioconductor package for isobaric tagged mass spectrometry data visualization, processing and quantitation. Bioinformatics 28, 288-289

High-confidence PSMs

TIS meta-data available (see Supplemental Dataset 3)

AND good MS2PIP correlation (see Supplemental Dataset 1)

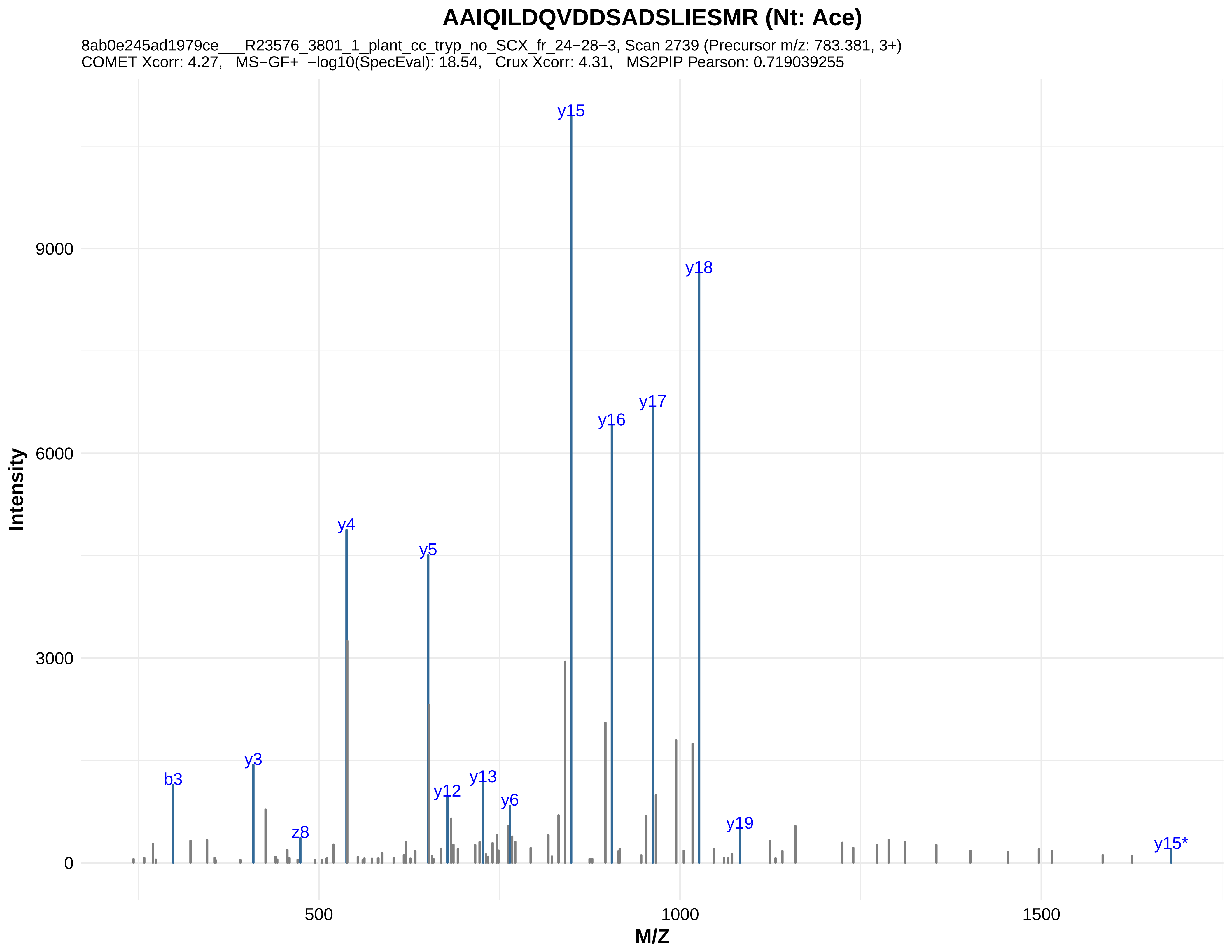
AAIQILDQVDDSADSLIESMR (Nt: Ace)
8ab0e245ad1979ce___R23576_3801_1_plant_cc_tryp_no_SCX_fr_24−28−3, Scan 2739 (Precursor m/z: 783.381, 3+)
COMET Xcorr: 4.27, MS−GF+ −log10(SpecEval): 18.54, Crux Xcorr: 4.31, MS2PIP Pearson: 0.719039255
Intensity
M/Z
0
3000
6000
9000
500
1000
1500
y15
y18
y17
y16
y4
y5
y3
b3
y13
y12
y6
z8
y19
y15*

AAIQILDQVDDSADSLIESMR (Nt: Ace)

d61db5162469cabf___L27065_2852_Petra_plant_CC_dark_32−28−9, Scan 2140 (Precursor m/z: 783.3812, 3+)
COMET Xcorr: 3.15, MS−GF+ −log10(SpecEval): 12.25, Crux Xcorr: 3.29, MS2PIP Pearson: 0.708167622

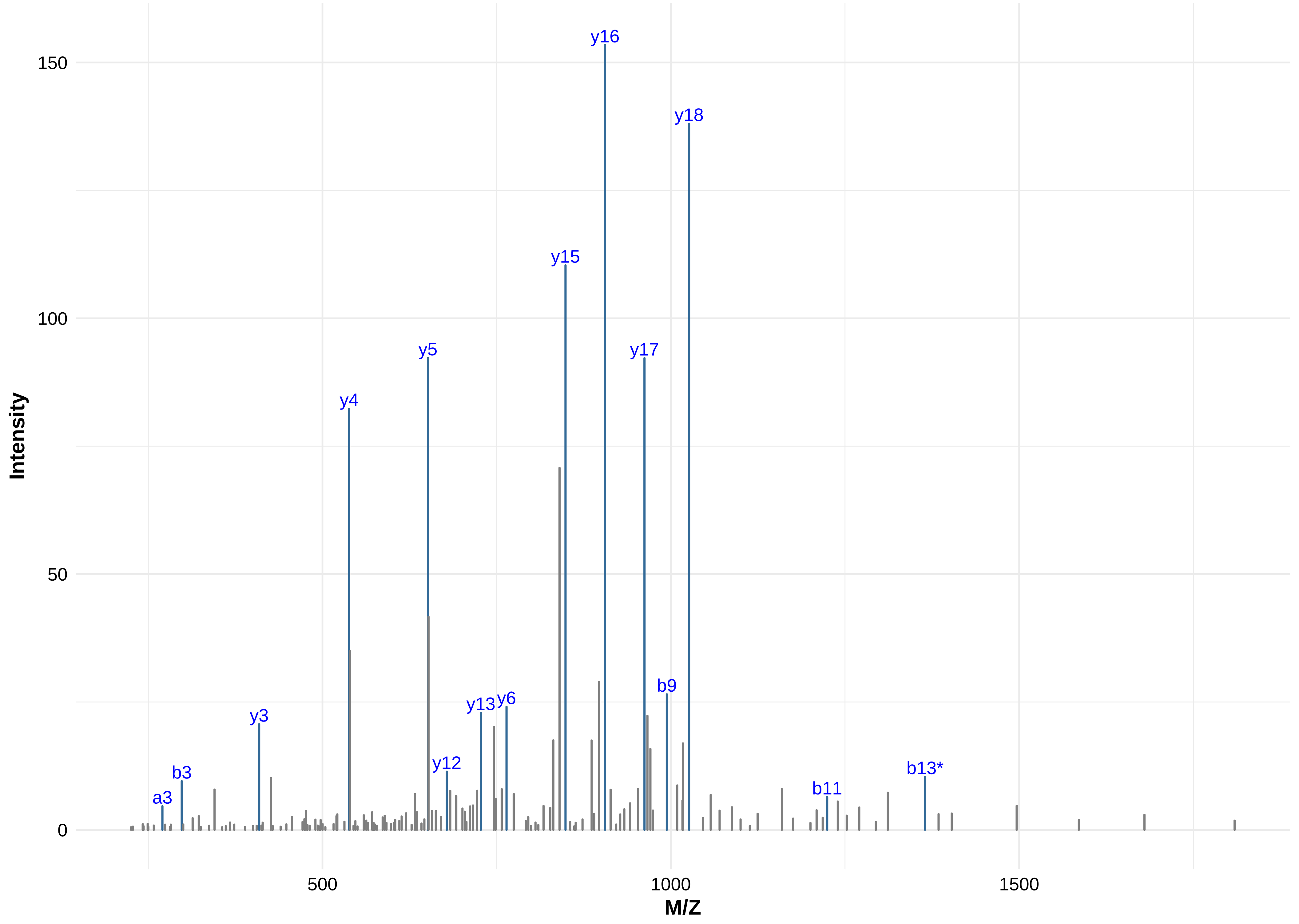

AAIQILDQVDDSADSLIESMR (Nt: Ace)

d61db5162469cabf___L27085_2852_Petra_plant_CC_dark_28−24−13, Scan 2639 (Precursor m/z: 783.3812, 3+)
COMET Xcorr: 2.92,   MS−GF+ −log10(SpecEval): 15.39,   Crux Xcorr: 2.52,   MS2PIP Pearson: 0.703432276

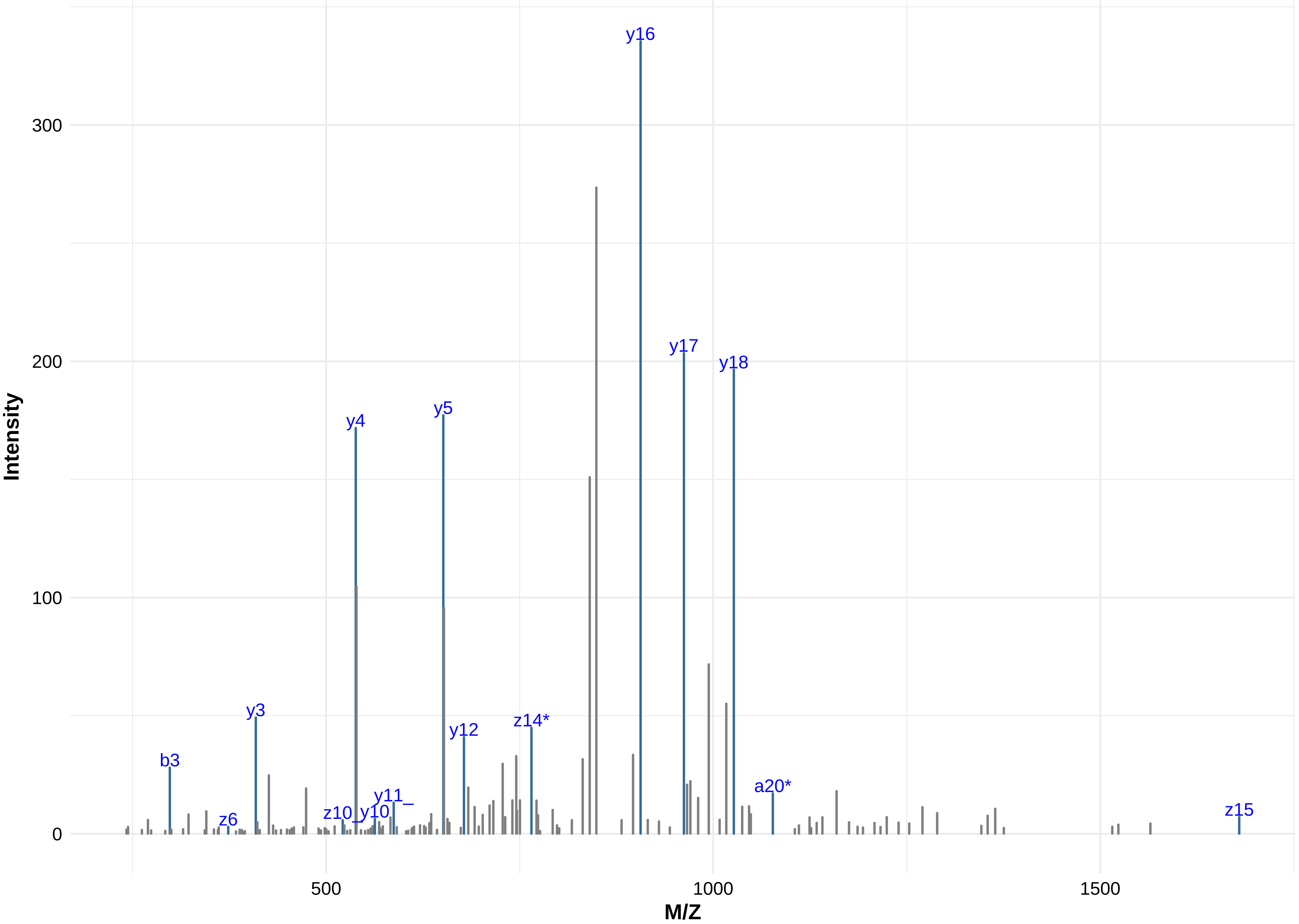

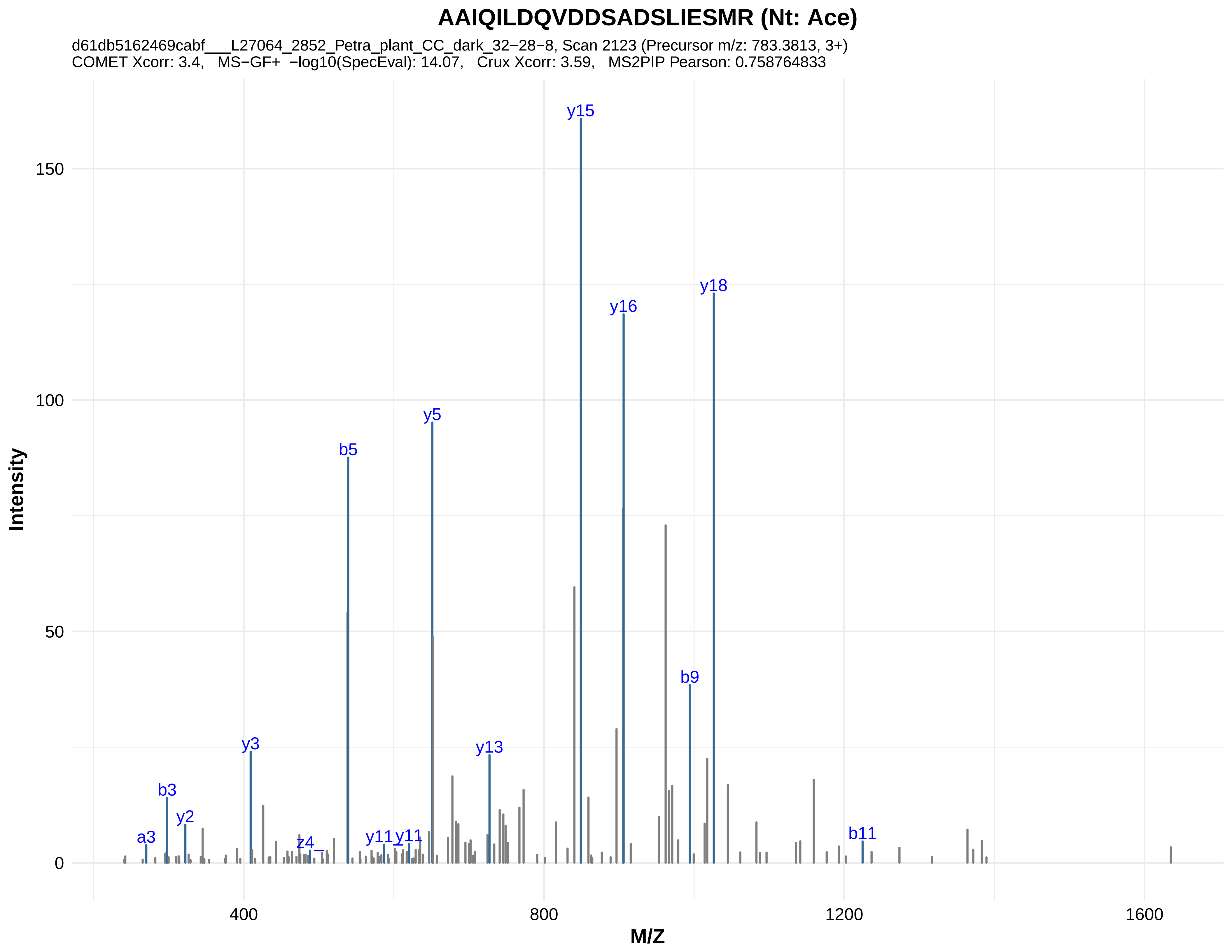

AAIQILDQVDDSADSLIESMR (Nt: Ace)
d61db5162469cabf___L27064_2852_Petra_plant_CC_dark_32−28−8, Scan 2123 (Precursor m/z: 783.3813, 3+)
COMET Xcorr: 3.4, MS−GF+ −log10(SpecEval): 14.07, Crux Xcorr: 3.59, MS2PIP Pearson: 0.758764833
Intensity
M/Z
y15
y18
y16
y5
b5
b9
y3
y13
b3
y2
a3
z4_
y11
y11
b11

AAIQILDQVDDSADSLIESMR (Nt: Ace)

8ab0e245ad1979ce___R23575_3801_1_plant_cc_tryp_no_SCX_fr_24−28−2, Scan 2628 (Precursor m/z: 783.3816, 3+)
COMET Xcorr: 4.17,   MS−GF+ −log10(SpecEval): 15.37,   Crux Xcorr: 4.13,   MS2PIP Pearson: 0.711454671

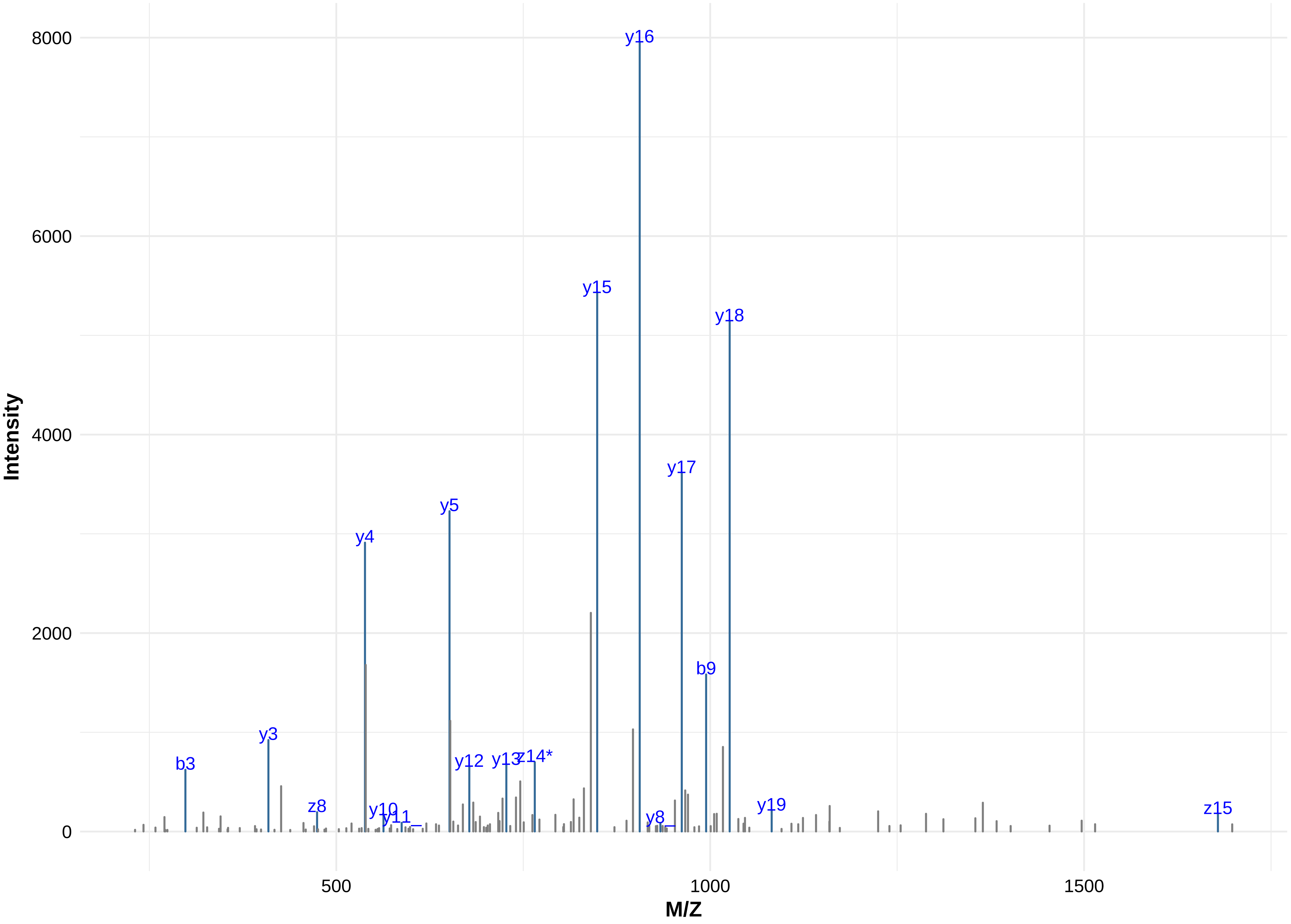

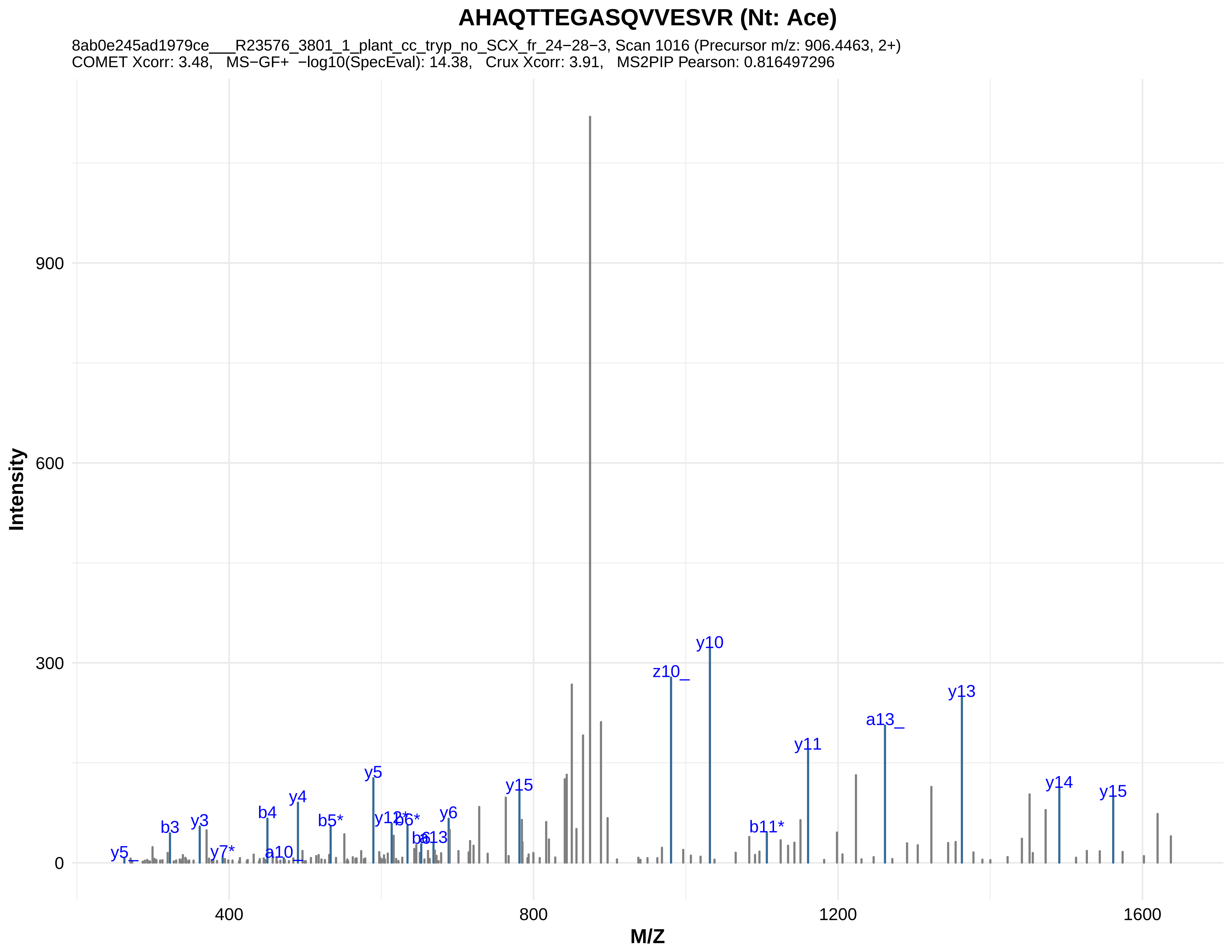

AHAQTTEGASQVVESVR (Nt: Ace)
8ab0e245ad1979ce___R23576_3801_1_plant_cc_tryp_no_SCX_fr_24−28−3, Scan 1016 (Precursor m/z: 906.4463, 2+)
COMET Xcorr: 3.48, MS−GF+ −log10(SpecEval): 14.38, Crux Xcorr: 3.91, MS2PIP Pearson: 0.816497296
Intensity
M/Z
0
300
600
900
400
800
1200
1600
y5_
b3
y3
y7*
b4
a10_
y4
b5*
y5
y12*
b6*
b6
a13
y6
y15
z10_
y10
b11*
y11
a13_
y13
y14
y15

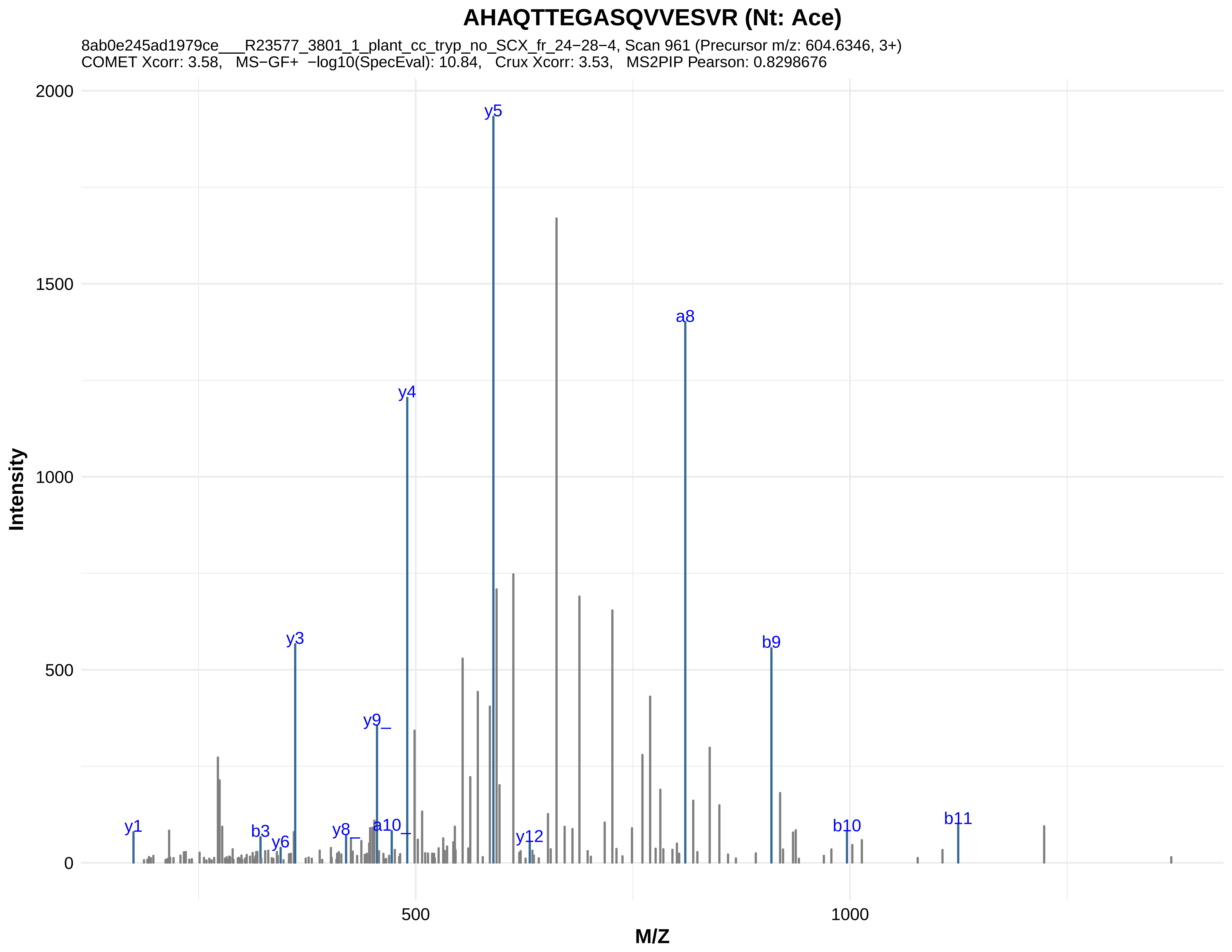
AHAQTTEGASQVVESVR (Nt: Ace)
8ab0e245ad1979ce___R23577_3801_1_plant_cc_tryp_no_SCX_fr_24−28−4, Scan 961 (Precursor m/z: 604.6346, 3+)
COMET Xcorr: 3.58, MS−GF+ −log10(SpecEval): 10.84, Crux Xcorr: 3.53, MS2PIP Pearson: 0.8298676
Intensity
M/Z
0
500
1000
1500
2000
500
1000
y5
a8
y4
y3
b9
y9_
y1
b3
y6
y8_
a10_
y12
b10
b11

AHAQTTEGASQVVESVR (Nt: Ace)

8ab0e245ad1979ce___R23577_3801_1_plant_cc_tryp_no_SCX_fr_24−28−4, Scan 962 (Precursor m/z: 906.4477, 2+)
COMET Xcorr: 3.68,   MS−GF+ −log10(SpecEval): 15.92,   Crux Xcorr: 4.34,   MS2PIP Pearson: 0.850090495

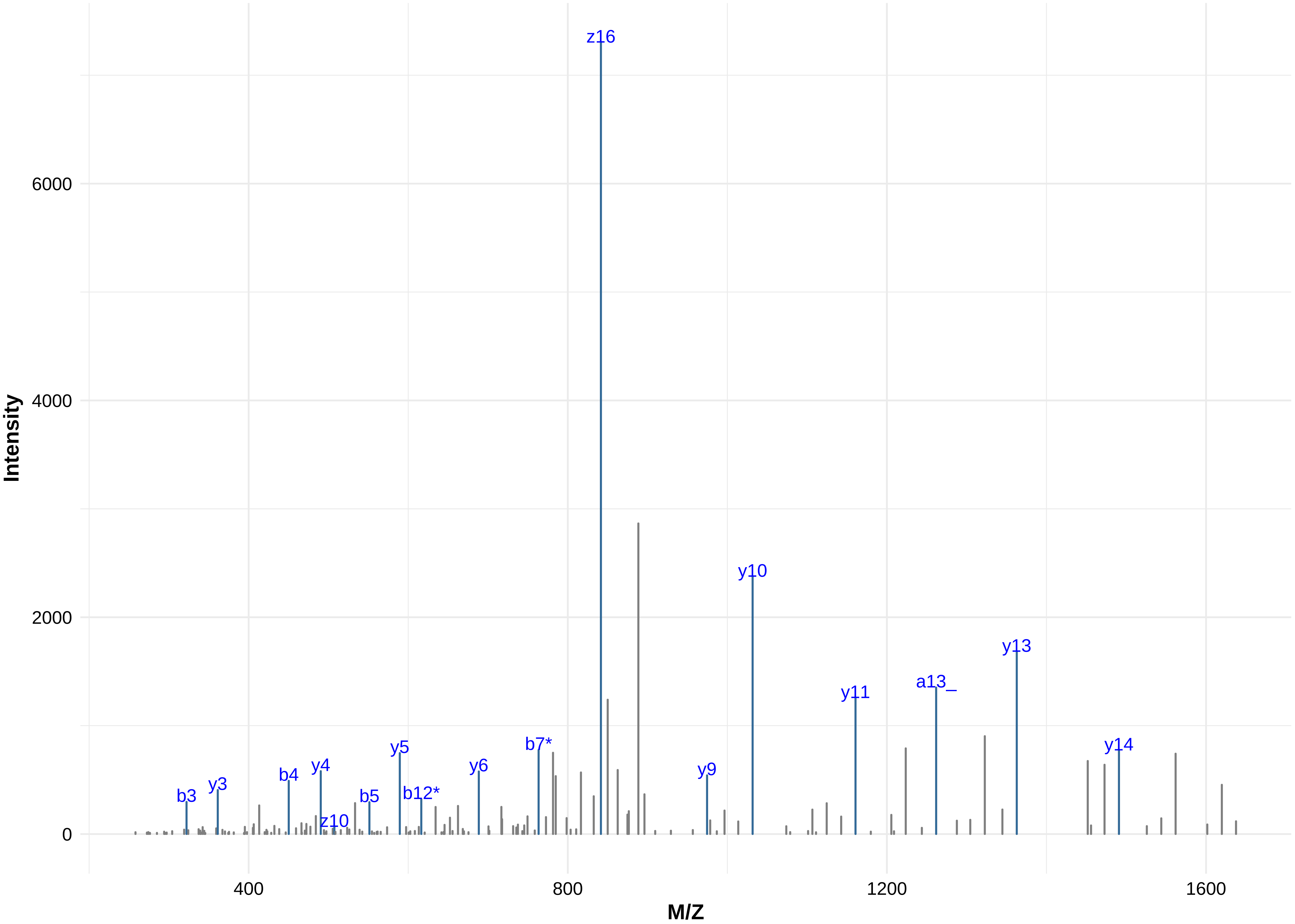

AHAQTTEGASQVVESVRF (Nt: Ace)

bccdd3e533766d9f___R23612_3802_2_plant_cc_chymo_no_SCX_fr_28−32−9, Scan 1225 (Precursor m/z: 979.983, 2+)
COMET Xcorr: 4.03, MS−GF+ −log10(SpecEval): 17.45, Crux Xcorr: 4.19, MS2PIP Pearson: 0.871972311

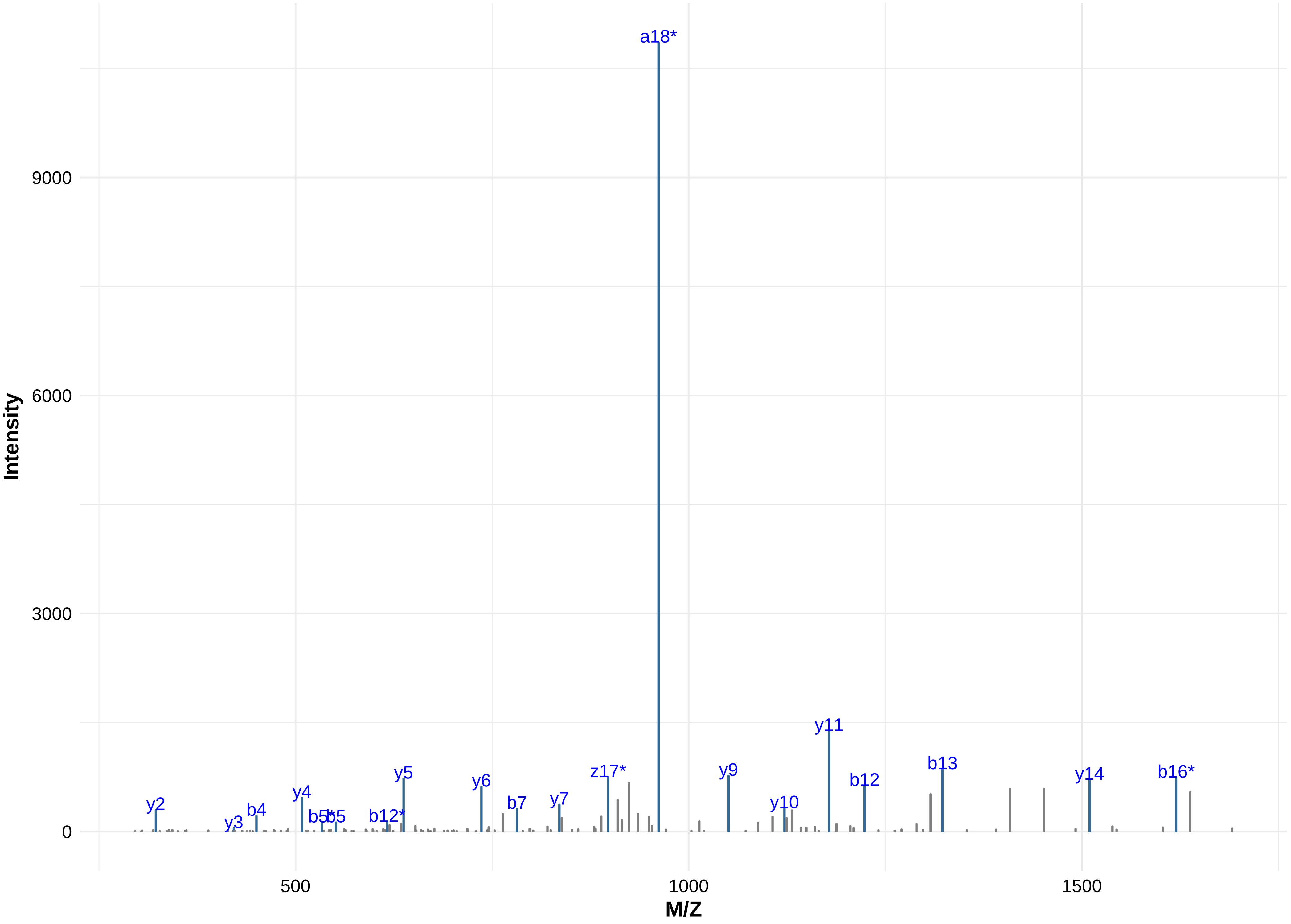

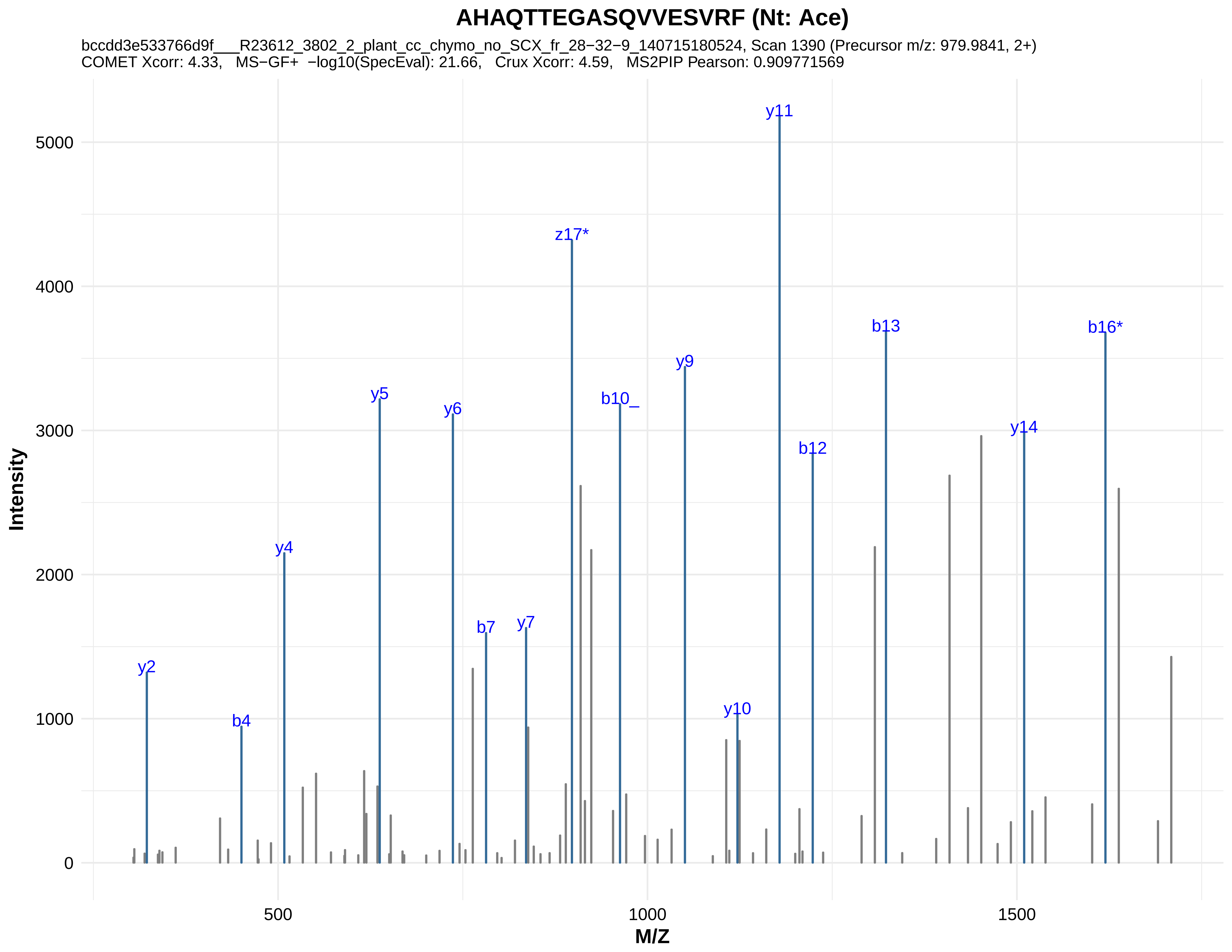
AHAQTTEGASQVVESVRF (Nt: Ace)
bccdd3e533766d9f___R23612_3802_2_plant_cc_chymo_no_SCX_fr_28−32−9_140715180524, Scan 1390 (Precursor m/z: 979.9841, 2+)
COMET Xcorr: 4.33, MS−GF+ −log10(SpecEval): 21.66, Crux Xcorr: 4.59, MS2PIP Pearson: 0.909771569
Intensity
M/Z
y11
z17*
b13
b16*
y9
y5
y6
b10_
y14
b12
y4
b7
y7
y2
y10
b4
0
1000
2000
3000
4000
5000
500
1000
1500

AHAQTTEGASQVVESVRF (Nt: Ace)

bccdd3e533766d9f___R23613_3802_2_plant_cc_chymo_no_SCX_fr_28−32−10_140715190945, Scan 1346 (Precursor m/z: 979.9837, 2+)
COMET Xcorr: 4.32, MS−GF+ −log10(SpecEval): 21.12, Crux Xcorr: 4.45, MS2PIP Pearson: 0.904010413

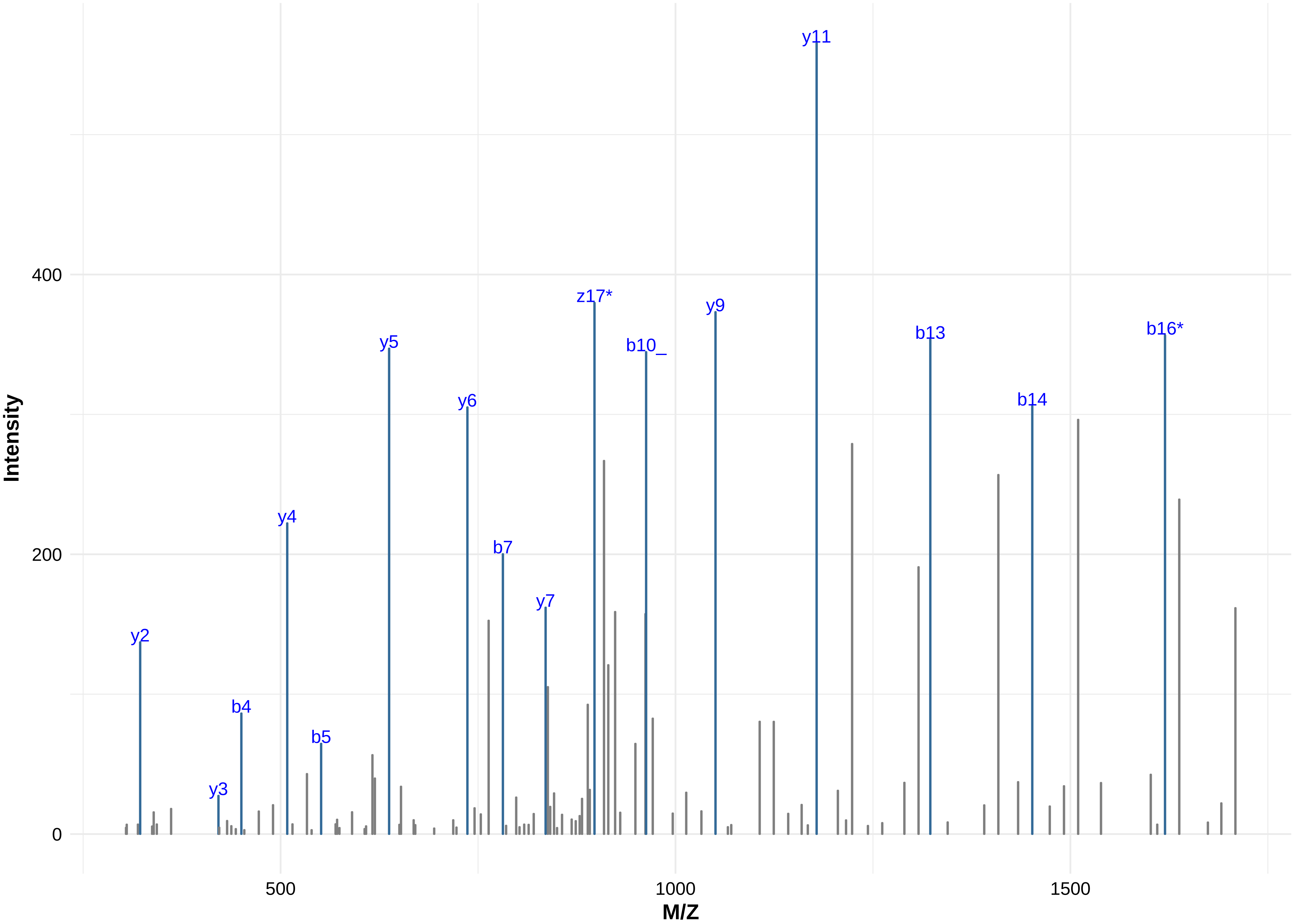

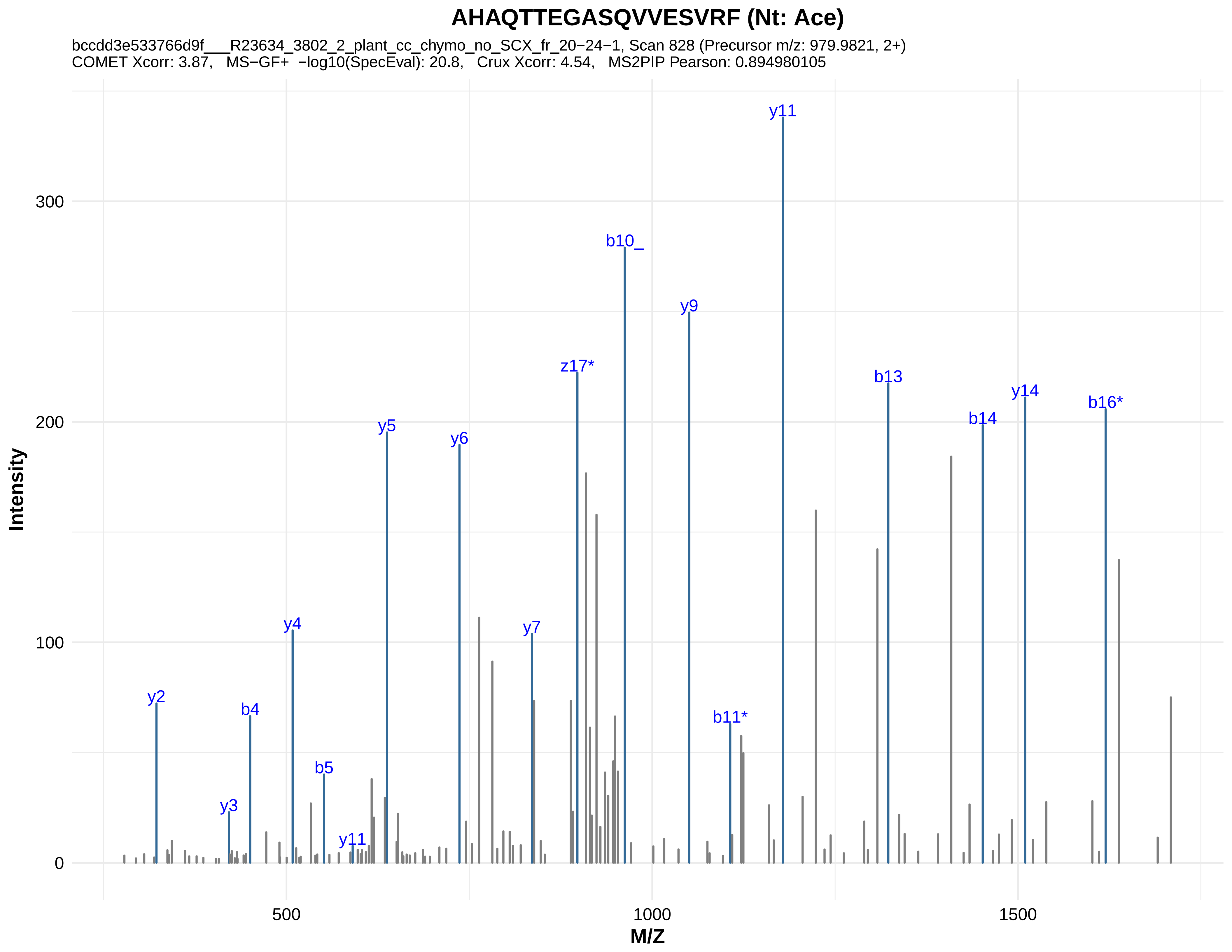
AHAQTTEGASQVVESVRF (Nt: Ace)
bccdd3e533766d9f___R23634_3802_2_plant_cc_chymo_no_SCX_fr_20−24−1, Scan 828 (Precursor m/z: 979.9821, 2+)
COMET Xcorr: 3.87, MS−GF+ −log10(SpecEval): 20.8, Crux Xcorr: 4.54, MS2PIP Pearson: 0.894980105
Intensity
M/Z
0
100
200
300
500
1000
1500
y2
y3
b4
y4
b5
y11
y5
y6
y7
z17*
b10_
y9
b11*
y11
b13
b14
y14
b16*

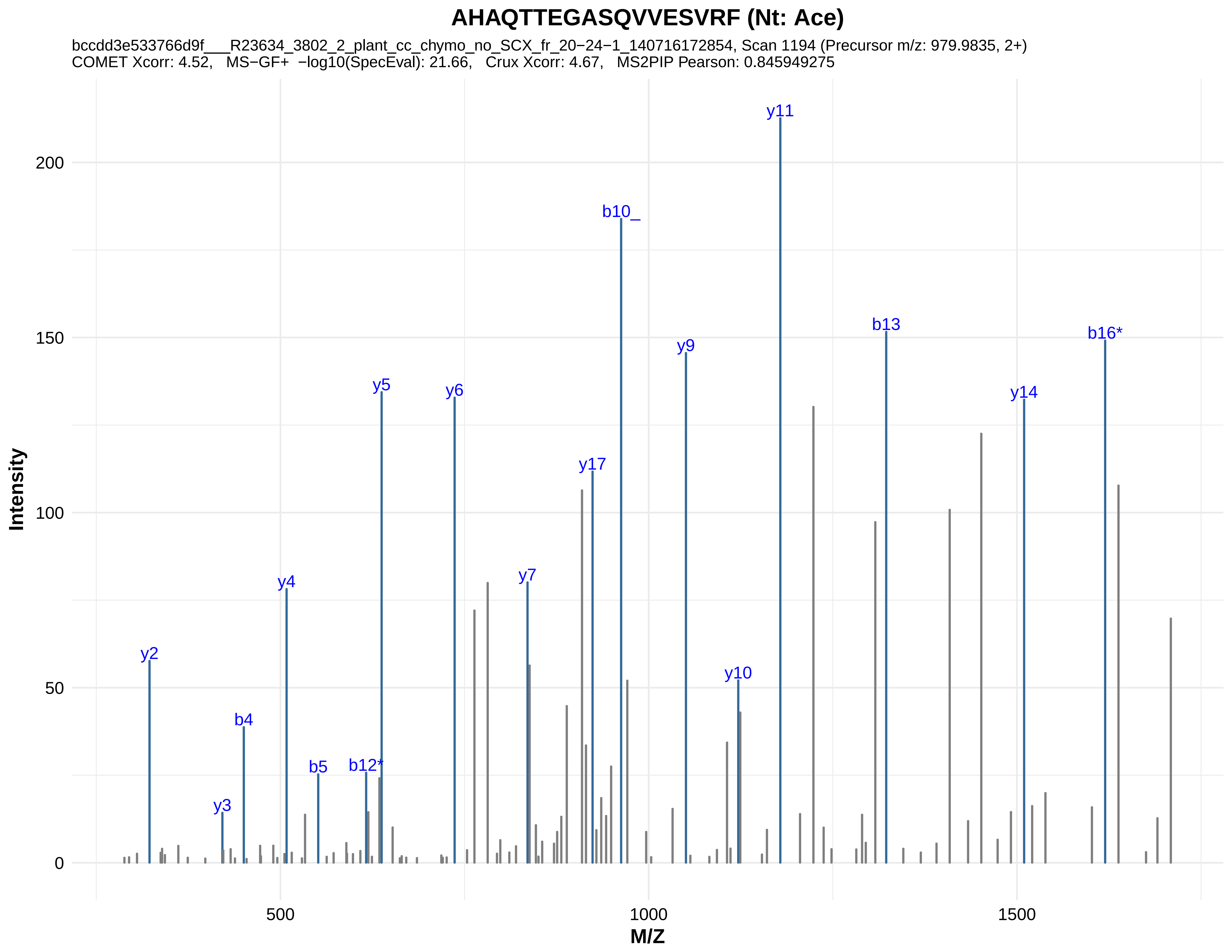

AHAQTTEGASQVVESVRF (Nt: Ace)
bccdd3e533766d9f___R23634_3802_2_plant_cc_chymo_no_SCX_fr_20−24−1_140716172854, Scan 1194 (Precursor m/z: 979.9835, 2+)
COMET Xcorr: 4.52, MS−GF+ −log10(SpecEval): 21.66, Crux Xcorr: 4.67, MS2PIP Pearson: 0.845949275
Intensity
M/Z
y2
y3
b4
y4
b5
b12*
y5
y6
y7
y17
b10_
y9
y10
y11
b13
y14
b16*

AMEDKSPTLLISEDLSR (Nt: Ace)

d61db5162469cabf___L27099_2852_Petra_plant_CC_dark_24−20−11, Scan 1712 (Precursor m/z: 1005.506, 2+)
COMET Xcorr: 3.52,   MS−GF+ −log10(SpecEval): 11.31,   Crux Xcorr: 3.36,   MS2PIP Pearson: 0.834930798

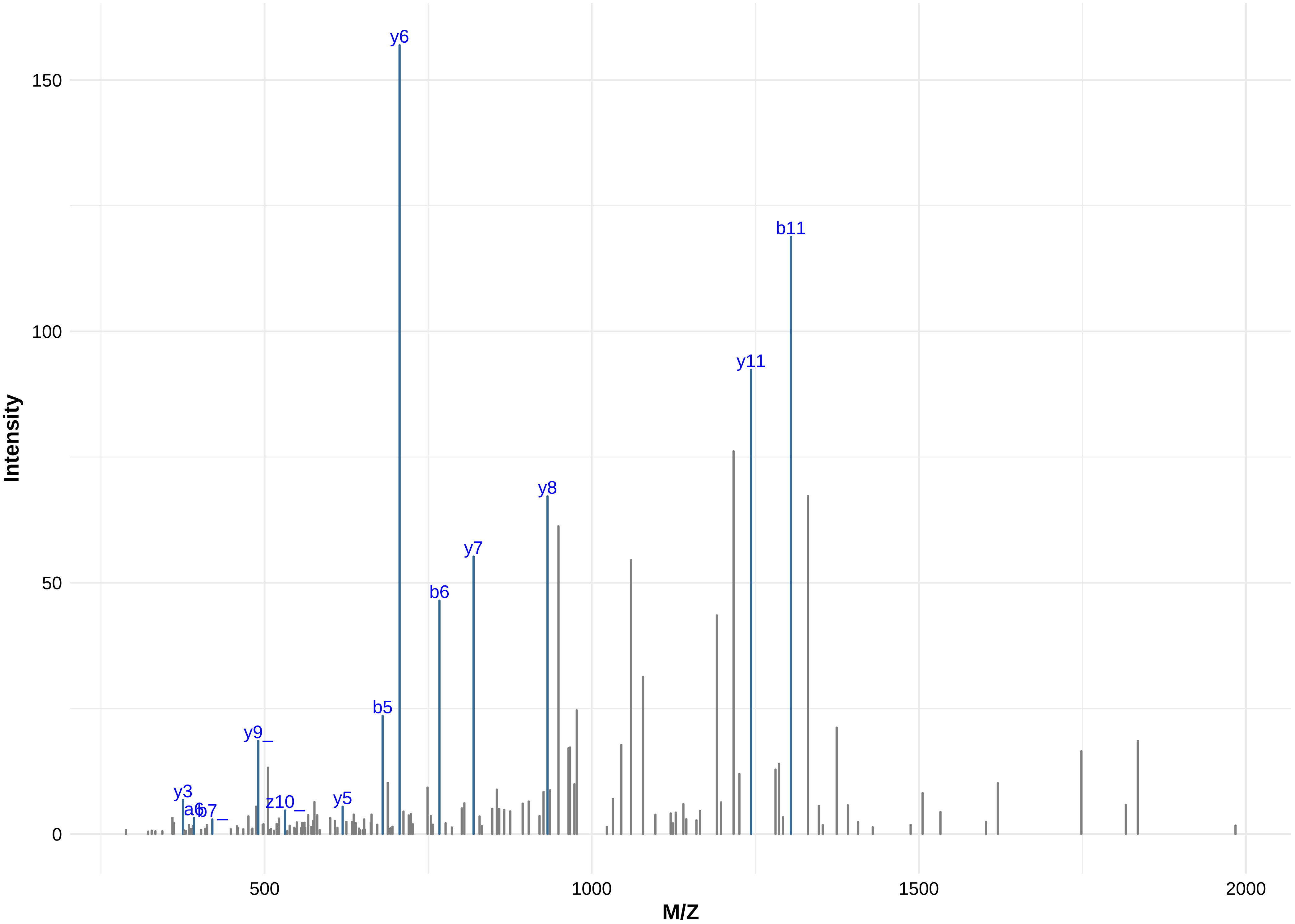

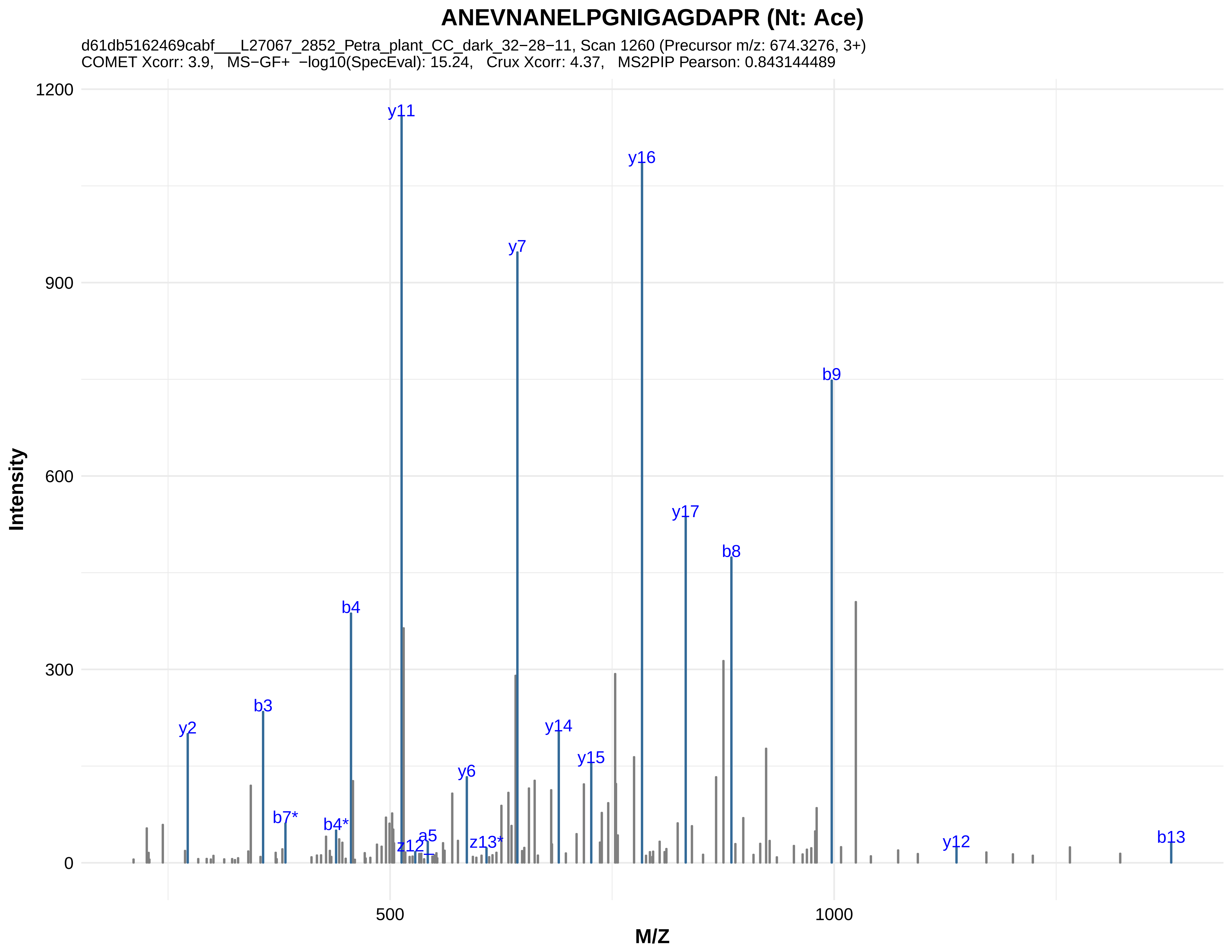
ANEVNANELPGNIGAGDAPR (Nt: Ace)
d61db5162469cabf___L27067_2852_Petra_plant_CC_dark_32−28−11, Scan 1260 (Precursor m/z: 674.3276, 3+)
COMET Xcorr: 3.9, MS−GF+ −log10(SpecEval): 15.24, Crux Xcorr: 4.37, MS2PIP Pearson: 0.843144489
Intensity
M/Z
1200
900
600
300
0
500
1000
y11
y16
y7
b9
y17
b8
b4
b3
y2
y14
y15
y6
b7*
b4*
a5
z12
z13*
y12
b13

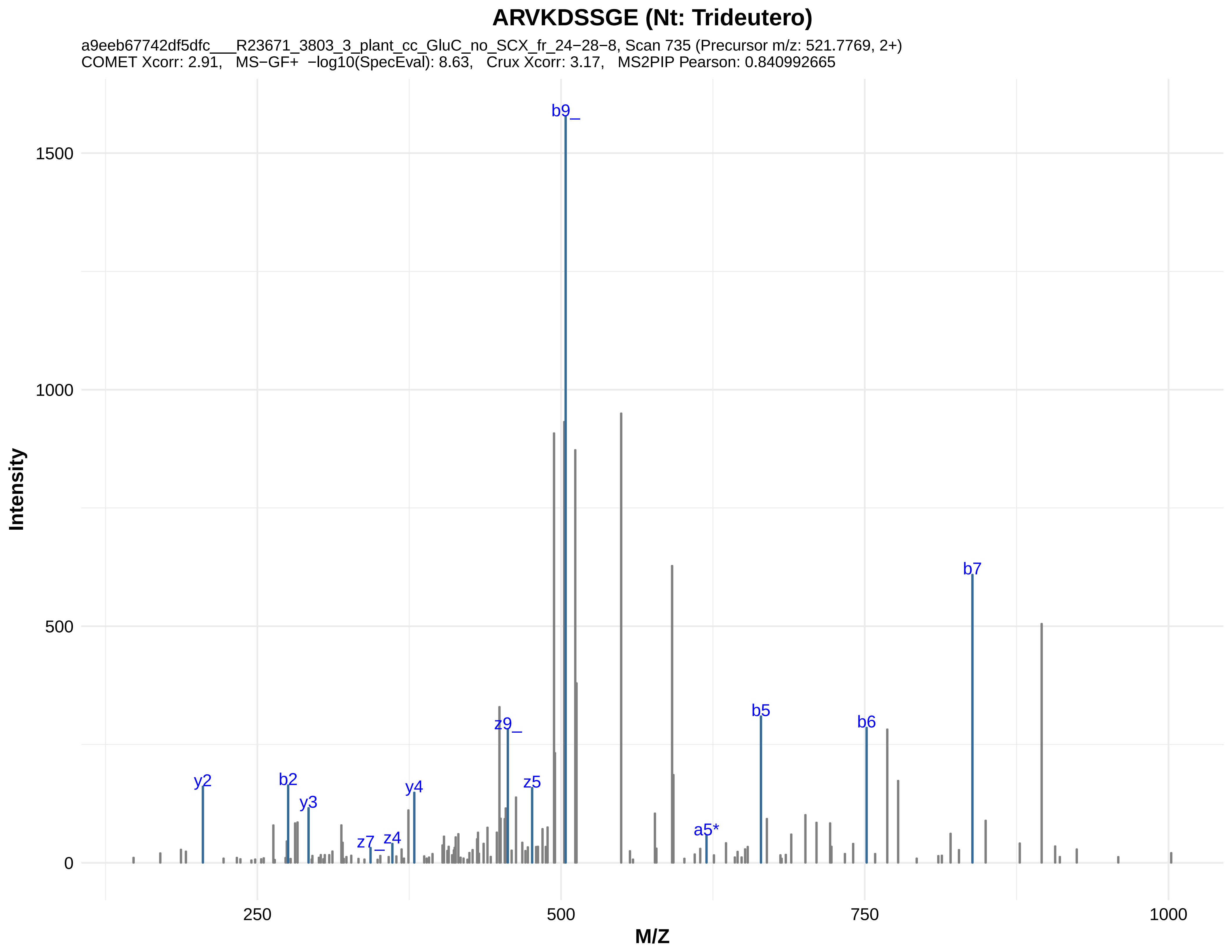
ARVKDSSGE (Nt: Trideutero)
a9eeb67742df5dfc___R23671_3803_3_plant_cc_GluC_no_SCX_fr_24−28−8, Scan 735 (Precursor m/z: 521.7769, 2+)
COMET Xcorr: 2.91, MS−GF+ −log10(SpecEval): 8.63, Crux Xcorr: 3.17, MS2PIP Pearson: 0.840992665
Intensity
M/Z
0
500
1000
1500
250
500
750
1000
b9_
b7
b5
b6
z9_
y2
b2
y3
y4
z5
z7_
z4
a5*

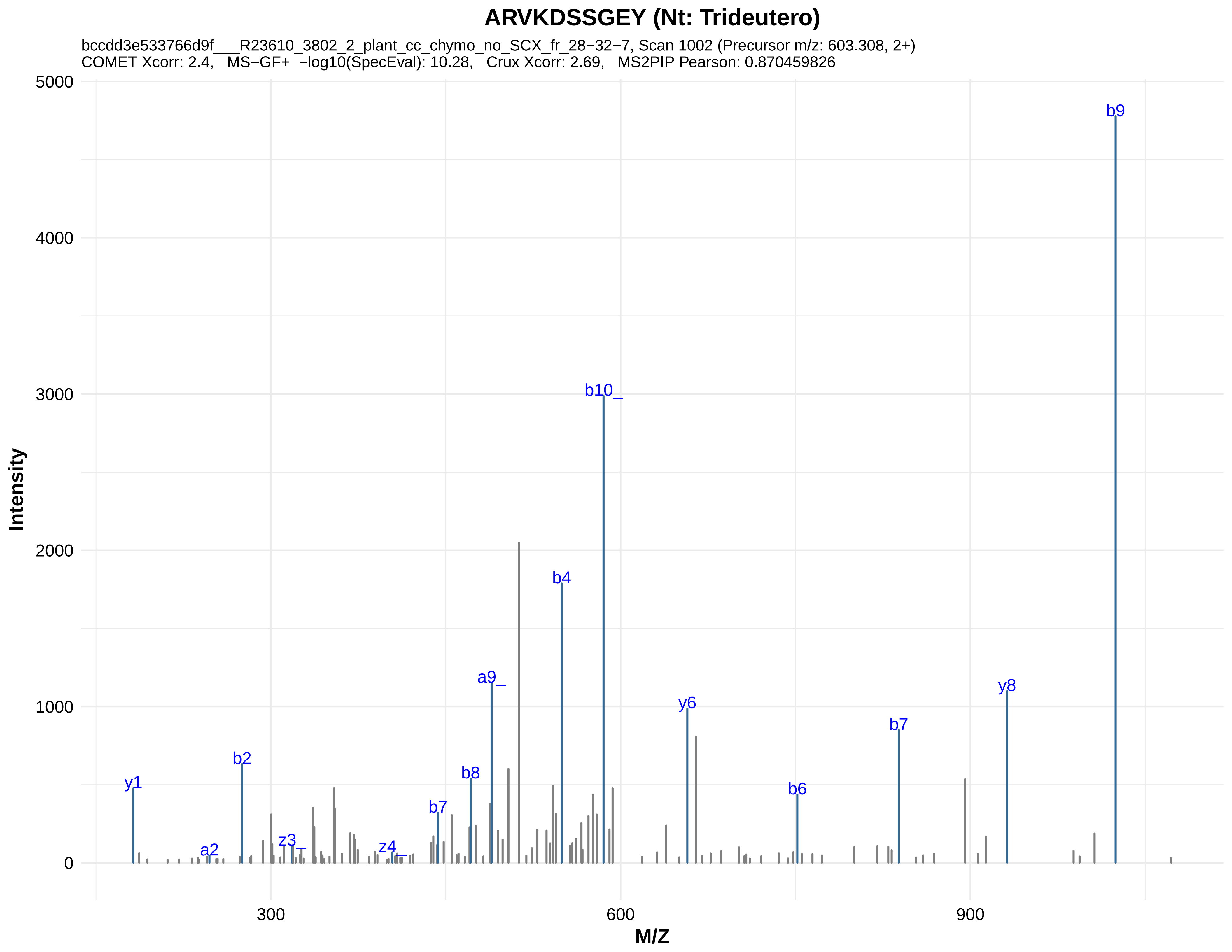

ARVKDSSGEY (Nt: Trideutero)
bccdd3e533766d9f___R23610_3802_2_plant_cc_chymo_no_SCX_fr_28−32−7, Scan 1002 (Precursor m/z: 603.308, 2+)
COMET Xcorr: 2.4, MS−GF+ −log10(SpecEval): 10.28, Crux Xcorr: 2.69, MS2PIP Pearson: 0.870459826
Intensity
M/Z
0
1000
2000
3000
4000
5000
300
600
900
y1
a2
b2
z3_
z4_
b7
b8
a9_
b4
b10_
y6
b6
b7
y8
b9

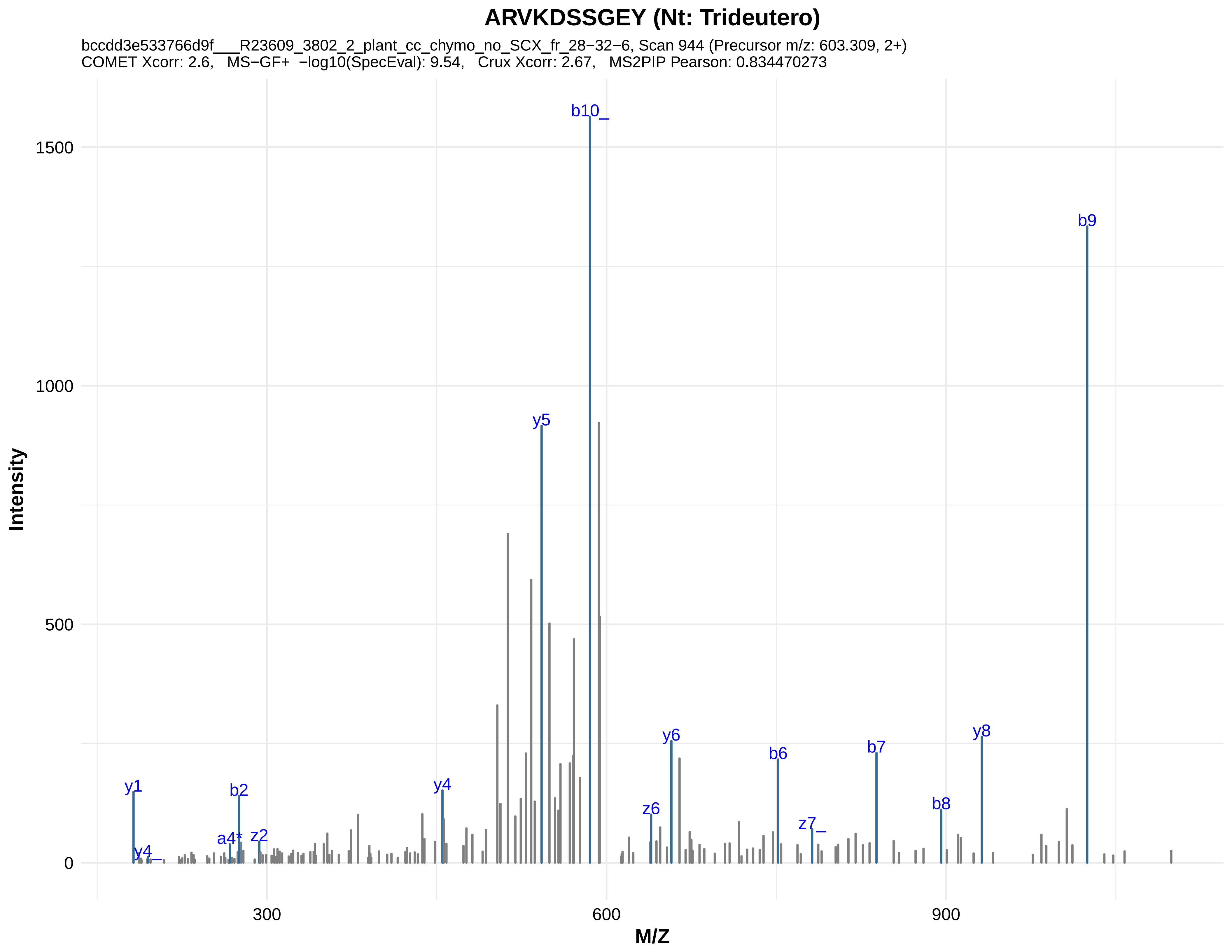

ARVKDSSGEY (Nt: Trideutero)
bccdd3e533766d9f___R23609_3802_2_plant_cc_chymo_no_SCX_fr_28−32−6, Scan 944 (Precursor m/z: 603.309, 2+)
COMET Xcorr: 2.6, MS−GF+ −log10(SpecEval): 9.54, Crux Xcorr: 2.67, MS2PIP Pearson: 0.834470273
Intensity
M/Z
0
500
1000
1500
300
600
900
b10_
b9
y5
y6
b6
b7
y8
b8
z6
z7_
y4
y1
y4_
b2
a4*
z2

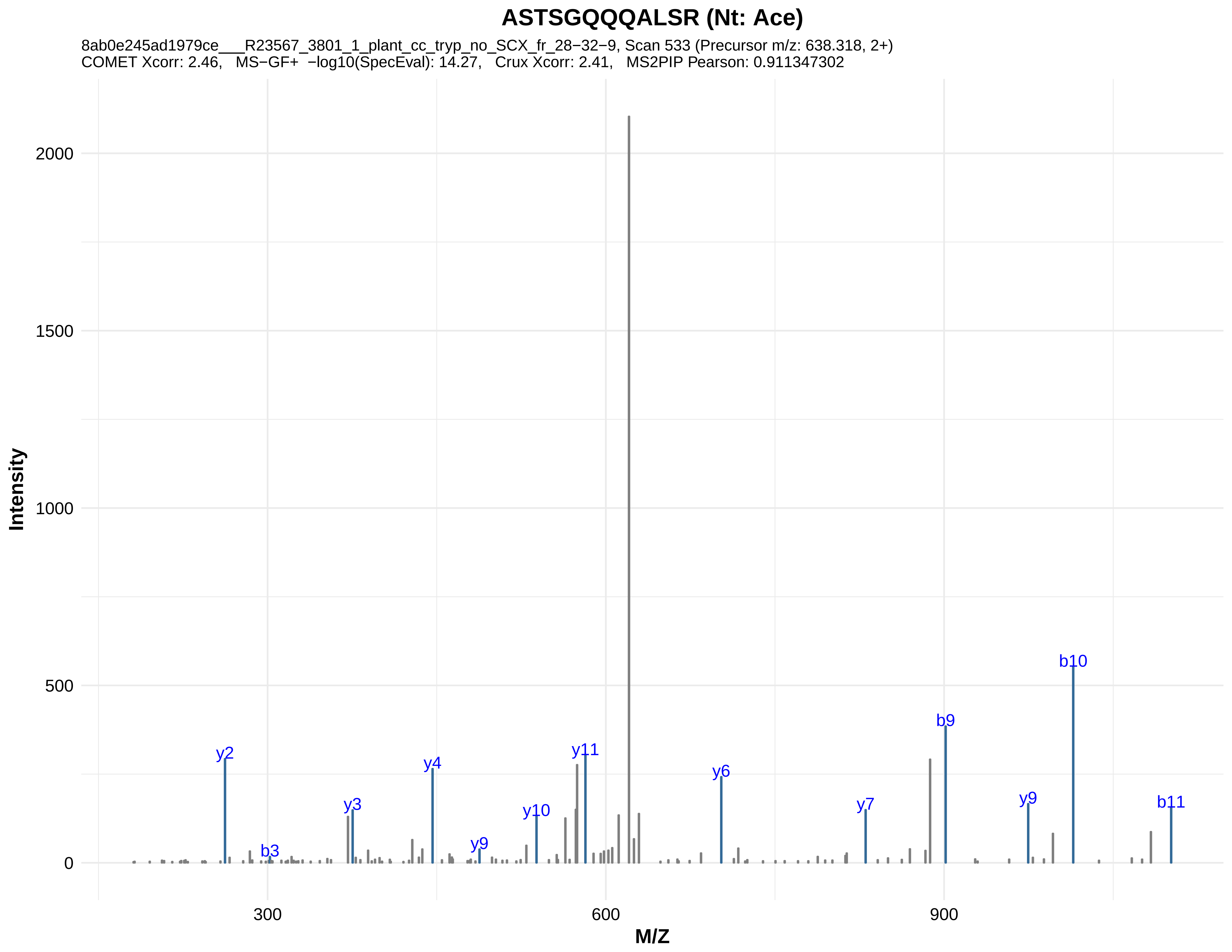

ASTSGQQQALSR (Nt: Ace)
8ab0e245ad1979ce___R23567_3801_1_plant_cc_tryp_no_SCX_fr_28−32−9, Scan 533 (Precursor m/z: 638.318, 2+)
COMET Xcorr: 2.46, MS−GF+ −log10(SpecEval): 14.27, Crux Xcorr: 2.41, MS2PIP Pearson: 0.911347302
Intensity
M/Z
0
500
1000
1500
2000
300
600
900
y2
b3
y3
y4
y9
y10
y11
y6
y7
b9
y9
b10
b11

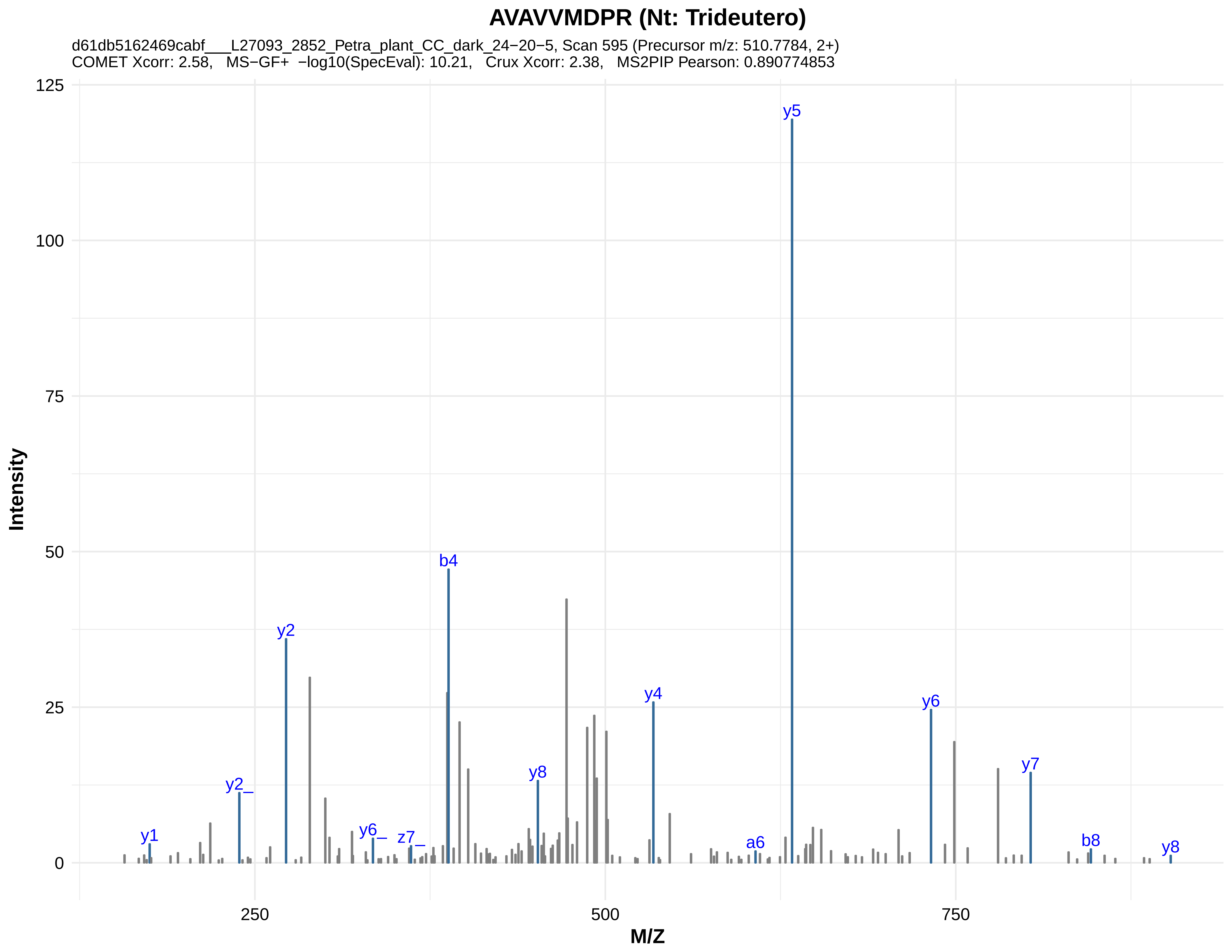
AVAVVMDPR (Nt: Trideutero)
d61db5162469cabf___L27093_2852_Petra_plant_CC_dark_24−20−5, Scan 595 (Precursor m/z: 510.7784, 2+)
COMET Xcorr: 2.58, MS−GF+ −log10(SpecEval): 10.21, Crux Xcorr: 2.38, MS2PIP Pearson: 0.890774853
Intensity
M/Z
y5
b4
y2
y4
y6
y2_
y8
y7
y1
y6_
z7_
a6
b8
y8
125
100
75
50
25
0
250
500
750

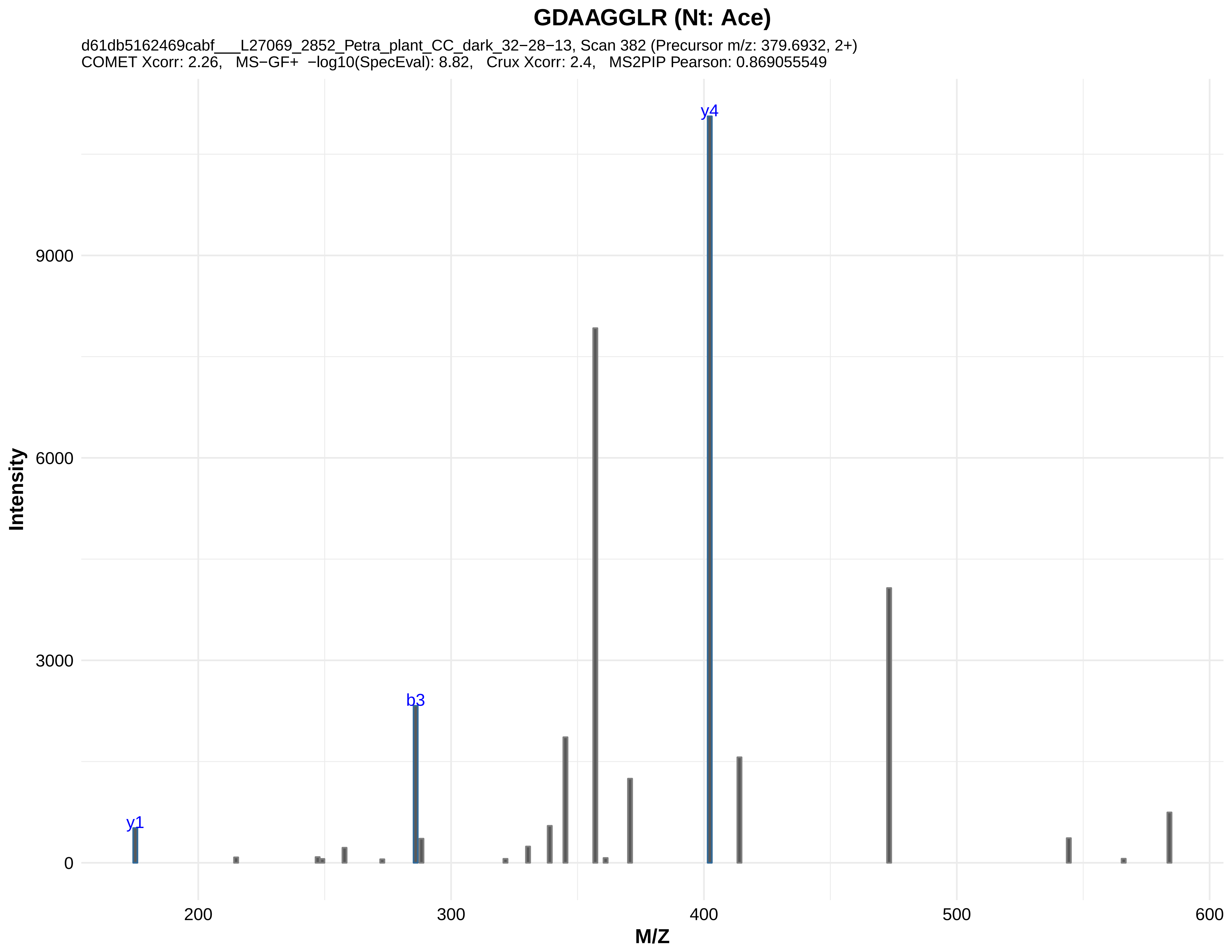
GDAAGGLR (Nt: Ace)
d61db5162469cabf___L27069_2852_Petra_plant_CC_dark_32−28−13, Scan 382 (Precursor m/z: 379.6932, 2+)
COMET Xcorr: 2.26, MS−GF+ −log10(SpecEval): 8.82, Crux Xcorr: 2.4, MS2PIP Pearson: 0.869055549
Intensity
0
3000
6000
9000
M/Z
200
300
400
500
600
y1
b3
y4

GDAAGGLR (Nt: Ace)

8ab0e245ad1979ce___R23561_3801_1_plant_cc_tryp_no_SCX_fr_28−32−3_140522121803, Scan 614 (Precursor m/z: 379.6933, 2+)
COMET Xcorr: 1.92, MS−GF+ −log10(SpecEval): 10.37, Crux Xcorr: 1.93, MS2PIP Pearson: 0.855728626

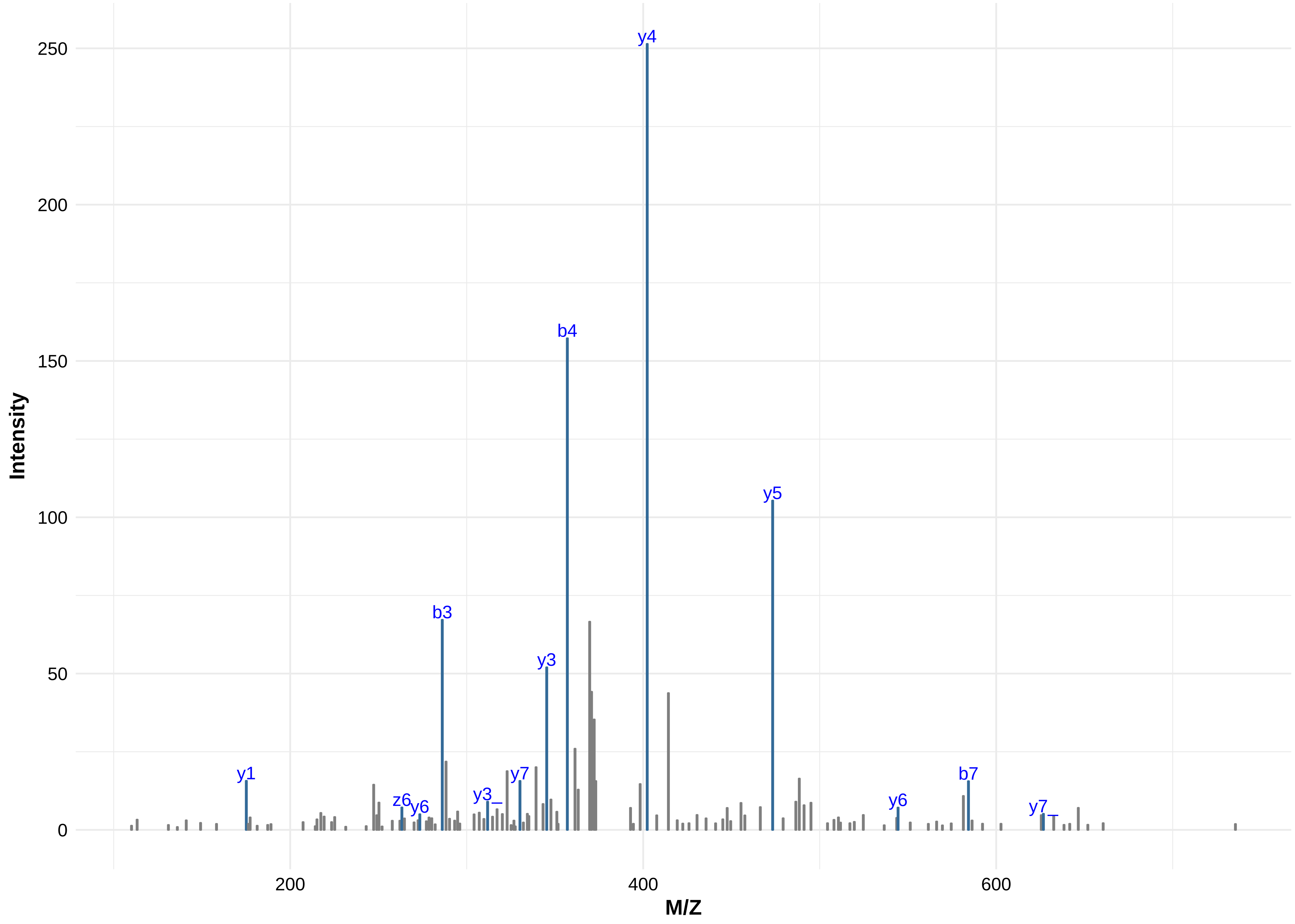

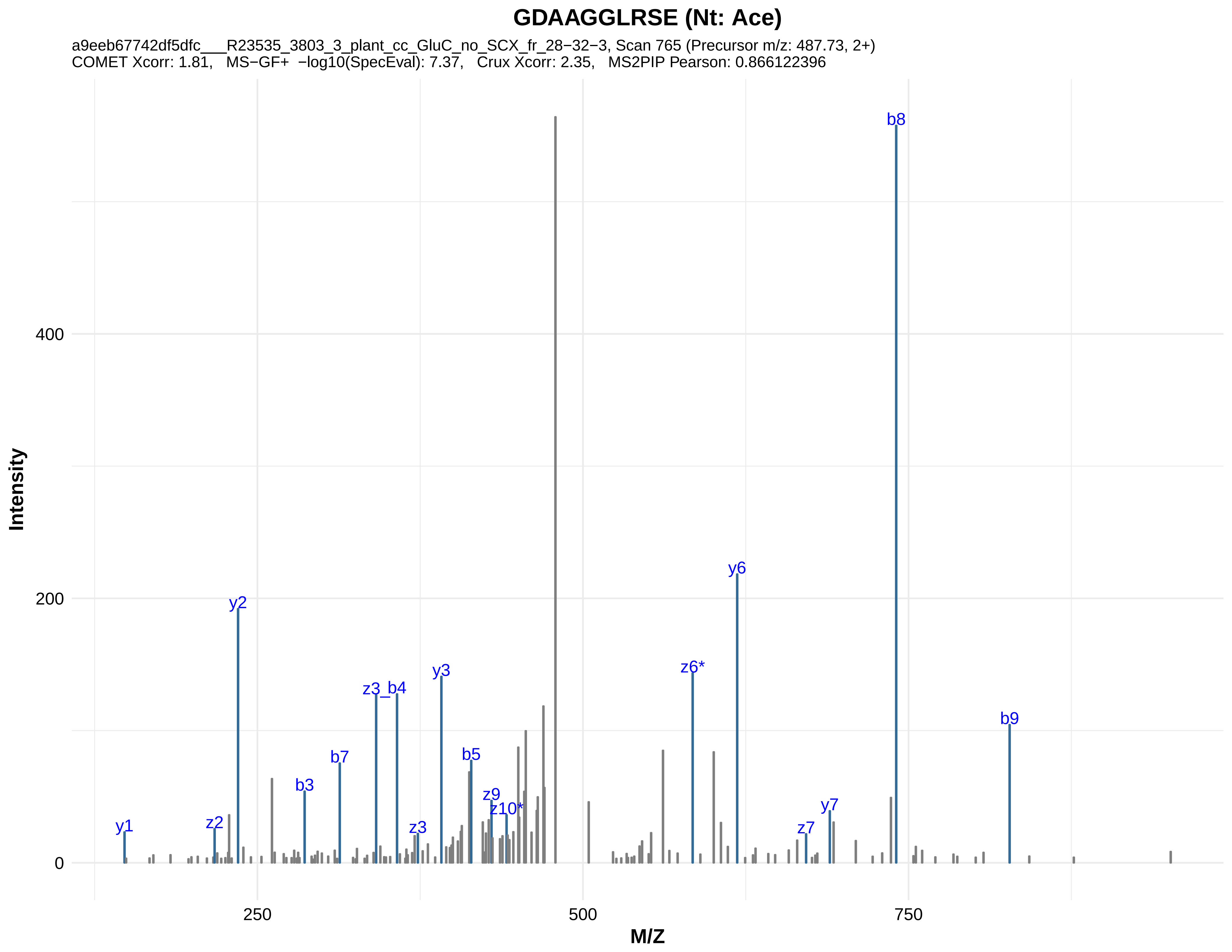
GDAAGGLRSE (Nt: Ace)
a9eeb67742df5dfc___R23535_3803_3_plant_cc_GluC_no_SCX_fr_28−32−3, Scan 765 (Precursor m/z: 487.73, 2+)
COMET Xcorr: 1.81, MS−GF+ −log10(SpecEval): 7.37, Crux Xcorr: 2.35, MS2PIP Pearson: 0.866122396
Intensity
M/Z
0
200
400
250
500
750
y1
z2
y2
b3
b7
z3_b4
z3
y3
b5
z9
z10*
z6*
y6
z7
y7
b8
b9

MDESNEIILQKSKR (Nt: Ace)

8ab0e245ad1979ce___R23561_3801_1_plant_cc_tryp_no_SCX_fr_28−32−3_140522121803, Scan 1486 (Precursor m/z: 921.9828, 2+)
COMET Xcorr: 2.7,   MS−GF+ −log10(SpecEval): 9.73,   Crux Xcorr: 3.13,   MS2PIP Pearson: 0.824169073

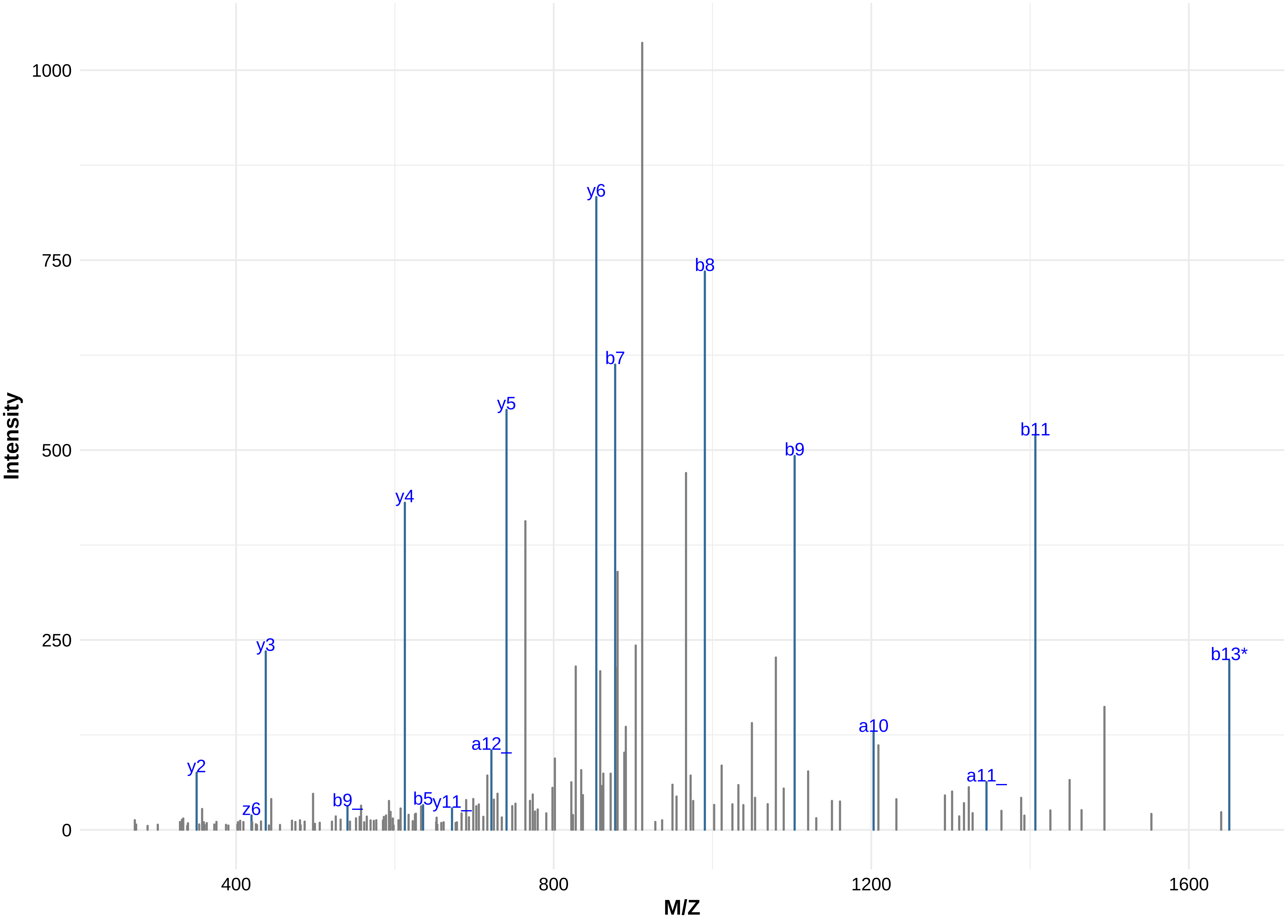

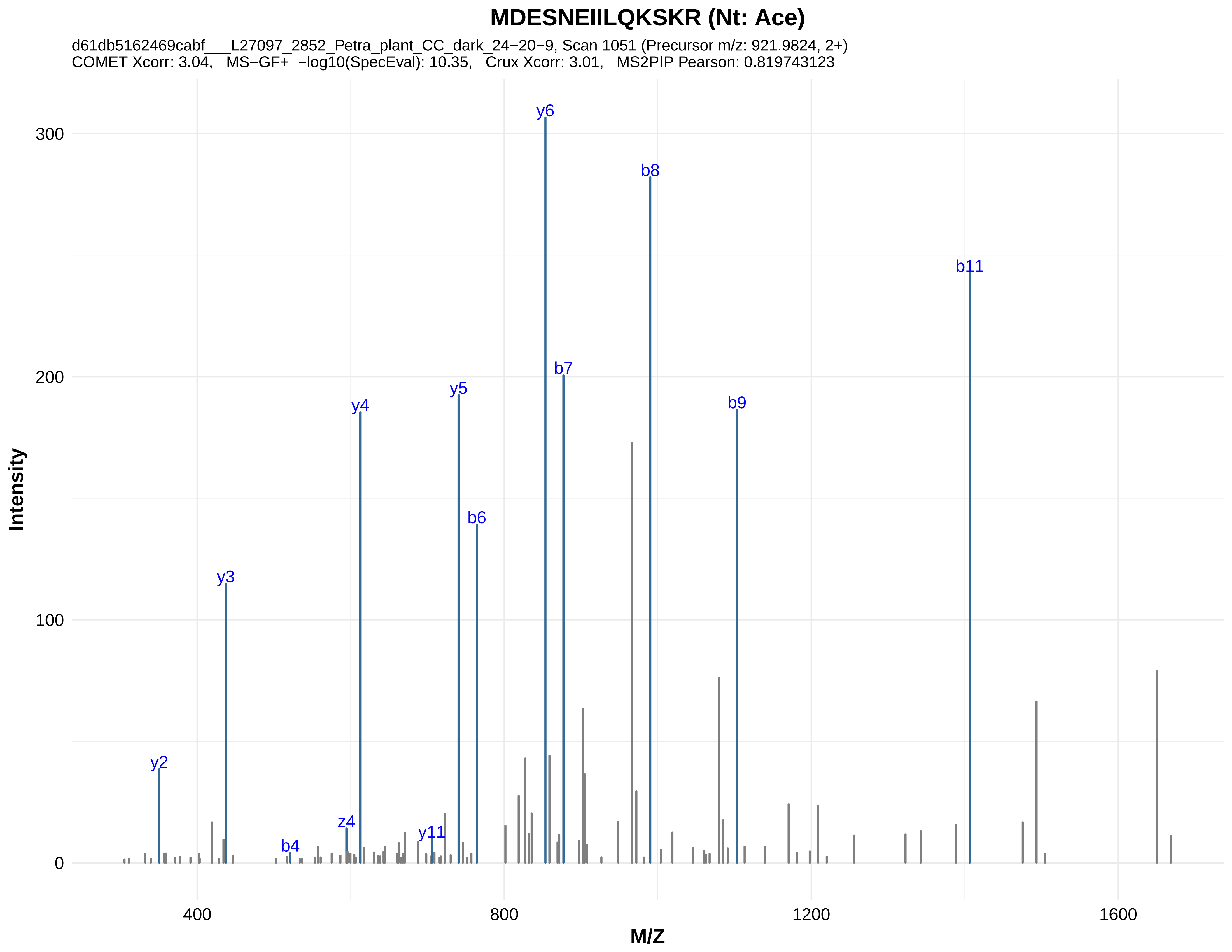
MDESNEIILQKSKR (Nt: Ace)
d61db5162469cabf___L27097_2852_Petra_plant_CC_dark_24−20−9, Scan 1051 (Precursor m/z: 921.9824, 2+)
COMET Xcorr: 3.04, MS−GF+ −log10(SpecEval): 10.35, Crux Xcorr: 3.01, MS2PIP Pearson: 0.819743123
Intensity
M/Z
y2
y3
b4
z4
y4
y11
y5
b6
y6
b7
b8
b9
b11

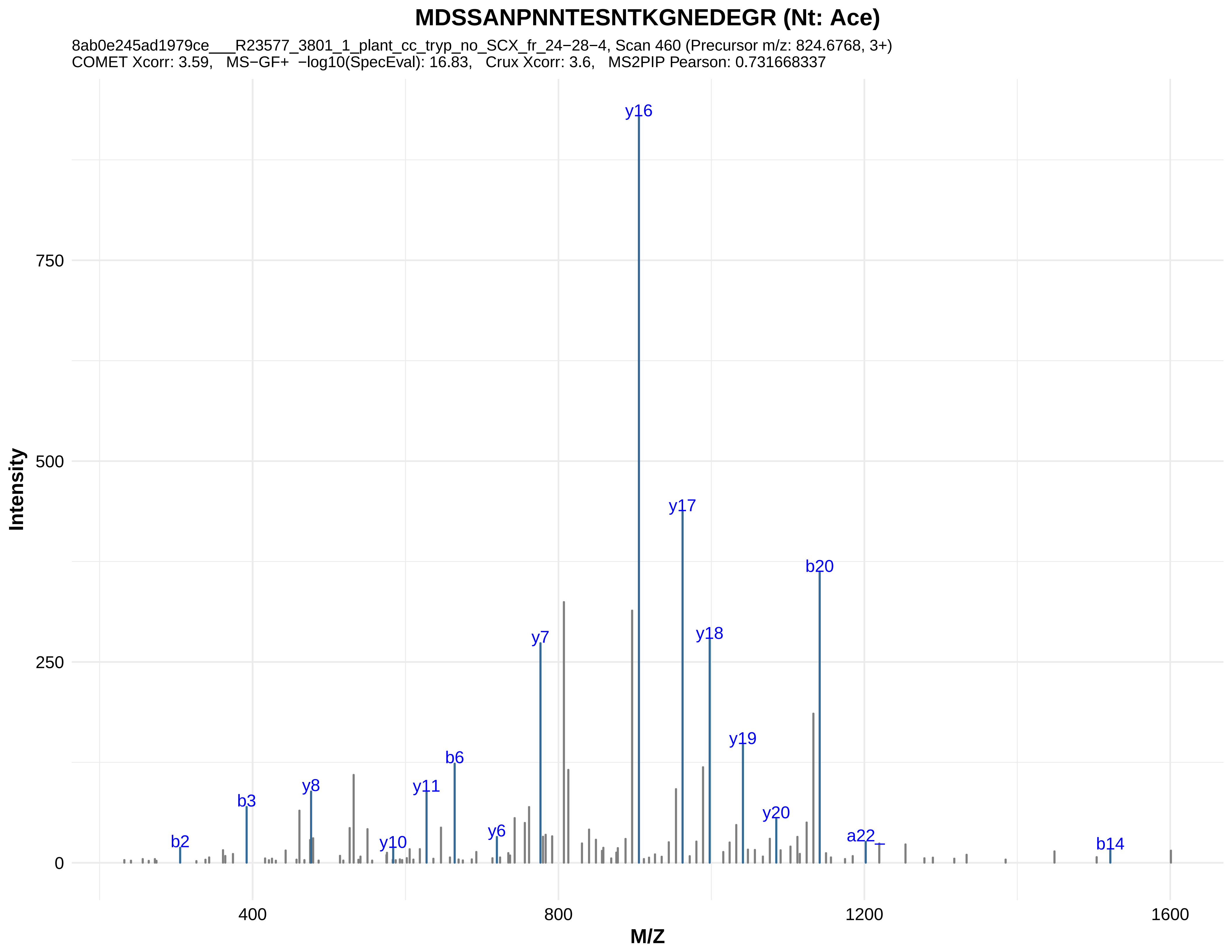

MDSSANPNNTESNTKGNEDEGR (Nt: Ace)
8ab0e245ad1979ce___R23577_3801_1_plant_cc_tryp_no_SCX_fr_24−28−4, Scan 460 (Precursor m/z: 824.6768, 3+)
COMET Xcorr: 3.59, MS−GF+ −log10(SpecEval): 16.83, Crux Xcorr: 3.6, MS2PIP Pearson: 0.731668337
Intensity
M/Z
y16
y17
b20
y7
y18
y19
b6
y8
y11
b3
y20
y6
y10
b2
a22_
b14
0
250
500
750
400
800
1200
1600

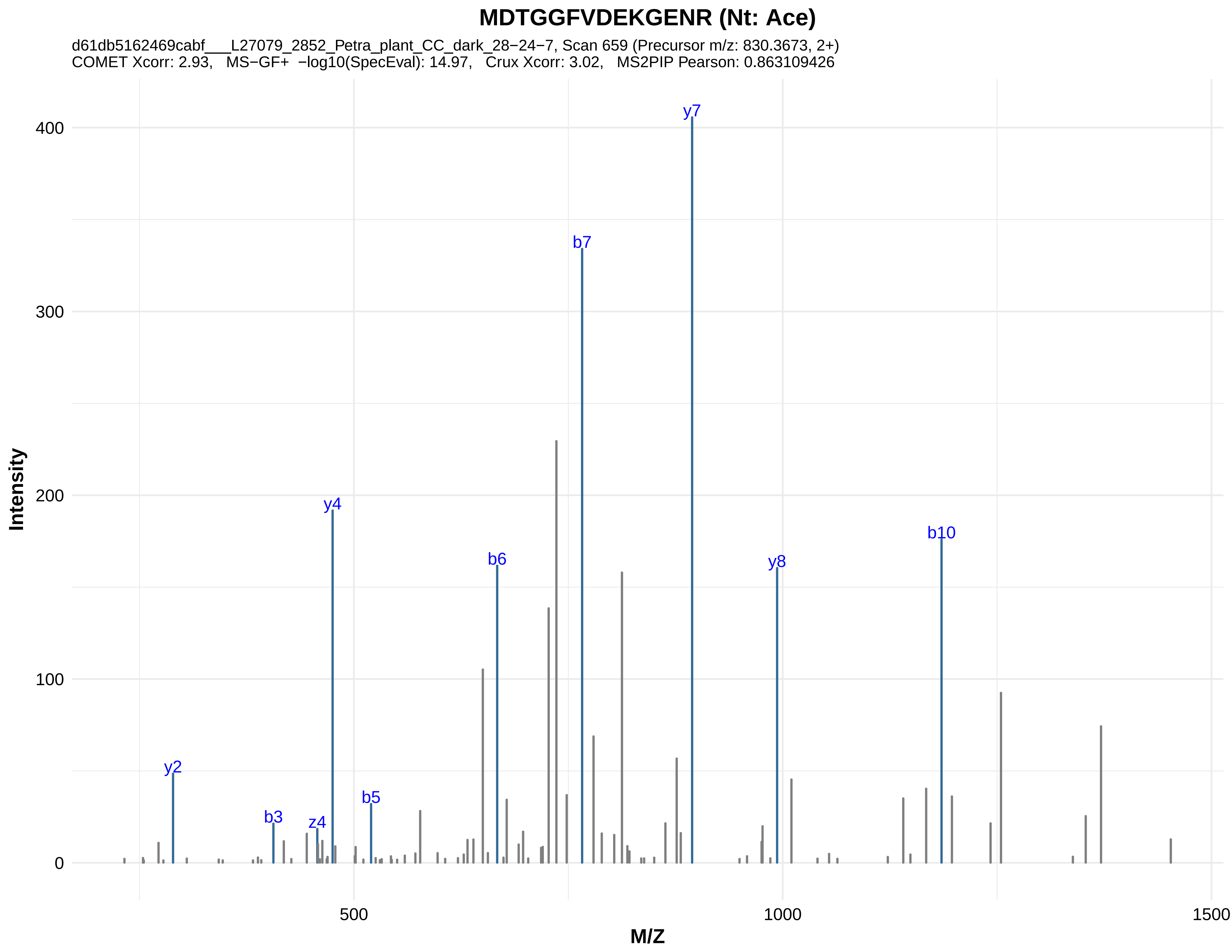

MDTGGFVDEKGENR (Nt: Ace)
d61db5162469cabf___L27079_2852_Petra_plant_CC_dark_28−24−7, Scan 659 (Precursor m/z: 830.3673, 2+)
COMET Xcorr: 2.93, MS−GF+ −log10(SpecEval): 14.97, Crux Xcorr: 3.02, MS2PIP Pearson: 0.863109426
Intensity
M/Z
y2
b3
z4
y4
b5
b6
b7
y7
y8
b10
0
100
200
300
400
500
1000
1500

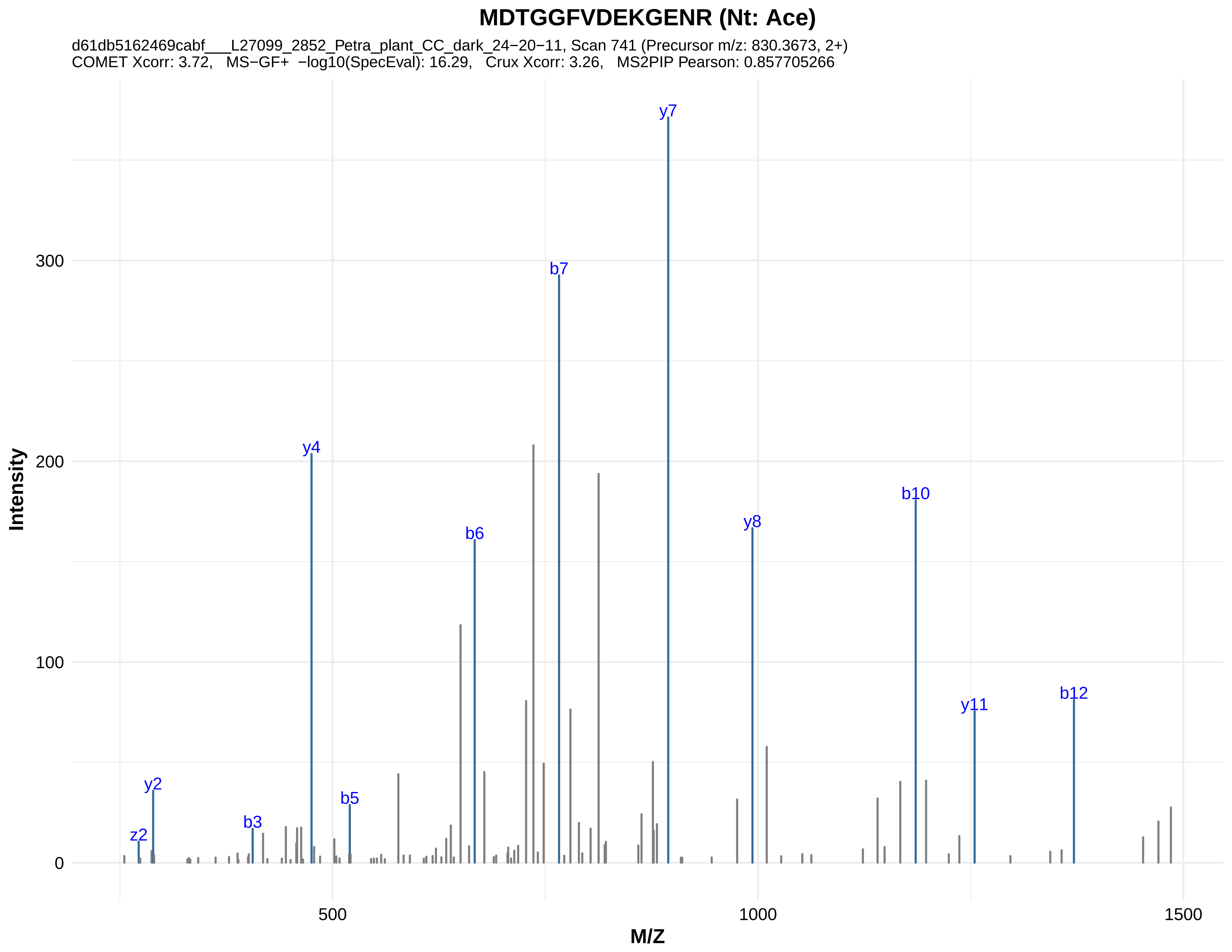
MDTGGFVDEKGENR (Nt: Ace)
d61db5162469cabf___L27099_2852_Petra_plant_CC_dark_24−20−11, Scan 741 (Precursor m/z: 830.3673, 2+)
COMET Xcorr: 3.72, MS−GF+ −log10(SpecEval): 16.29, Crux Xcorr: 3.26, MS2PIP Pearson: 0.857705266
Intensity
M/Z
0
100
200
300
500
1000
1500
y7
b7
y4
b6
y8
b10
y11
b12
y2
z2
b3
b5

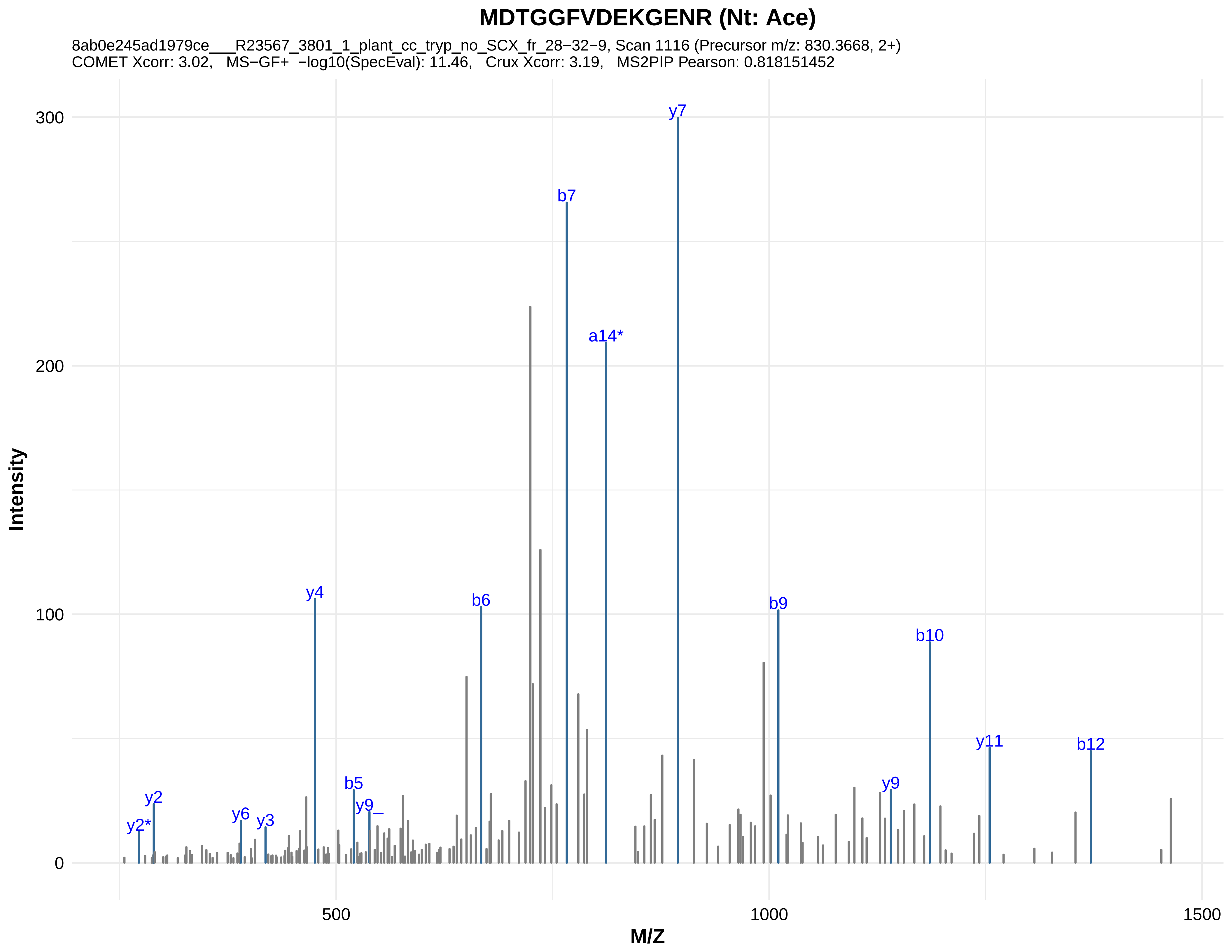
MDTGGFVDEKGENR (Nt: Ace)
8ab0e245ad1979ce___R23567_3801_1_plant_cc_tryp_no_SCX_fr_28−32−9, Scan 1116 (Precursor m/z: 830.3668, 2+)
COMET Xcorr: 3.02, MS−GF+ −log10(SpecEval): 11.46, Crux Xcorr: 3.19, MS2PIP Pearson: 0.818151452
Intensity
M/Z
y7
b7
a14*
y4
b6
b9
b10
y11
b12
y9
b5
y9_
y2
y2*
y6
y3
0
100
200
300
500
1000
1500

MDTNSLMLIDNNGSFE (Nt: Ace)

a9eeb67742df5dfc___R23657_3803_3_plant_cc_GluC_no_SCX_fr_28−32−9, Scan 1629 (Precursor m/z: 937.893, 2+)
COMET Xcorr: 3.64, MS−GF+ −log10(SpecEval): 12.71, Crux Xcorr: 3.66, MS2PIP Pearson: 0.7877696

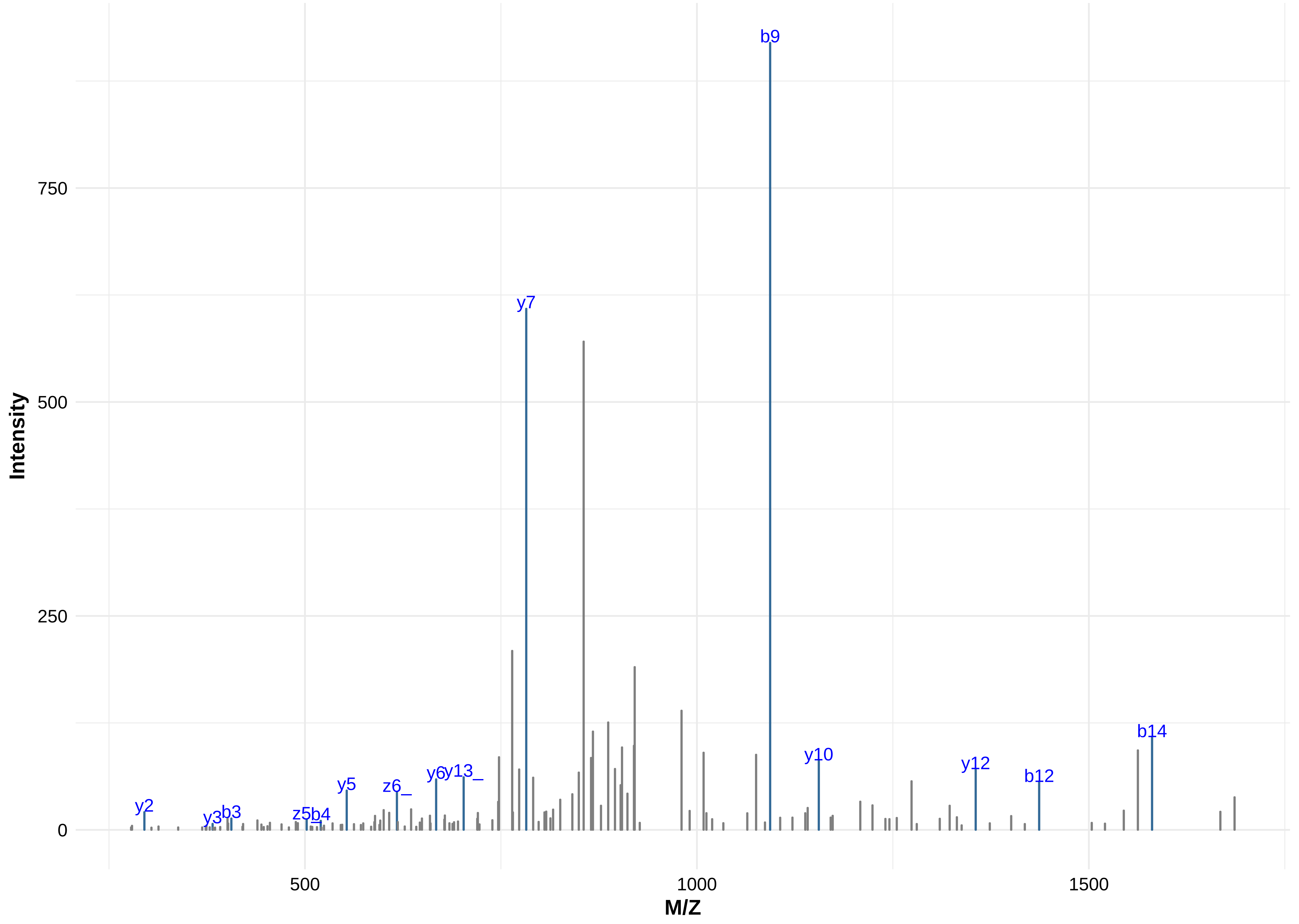

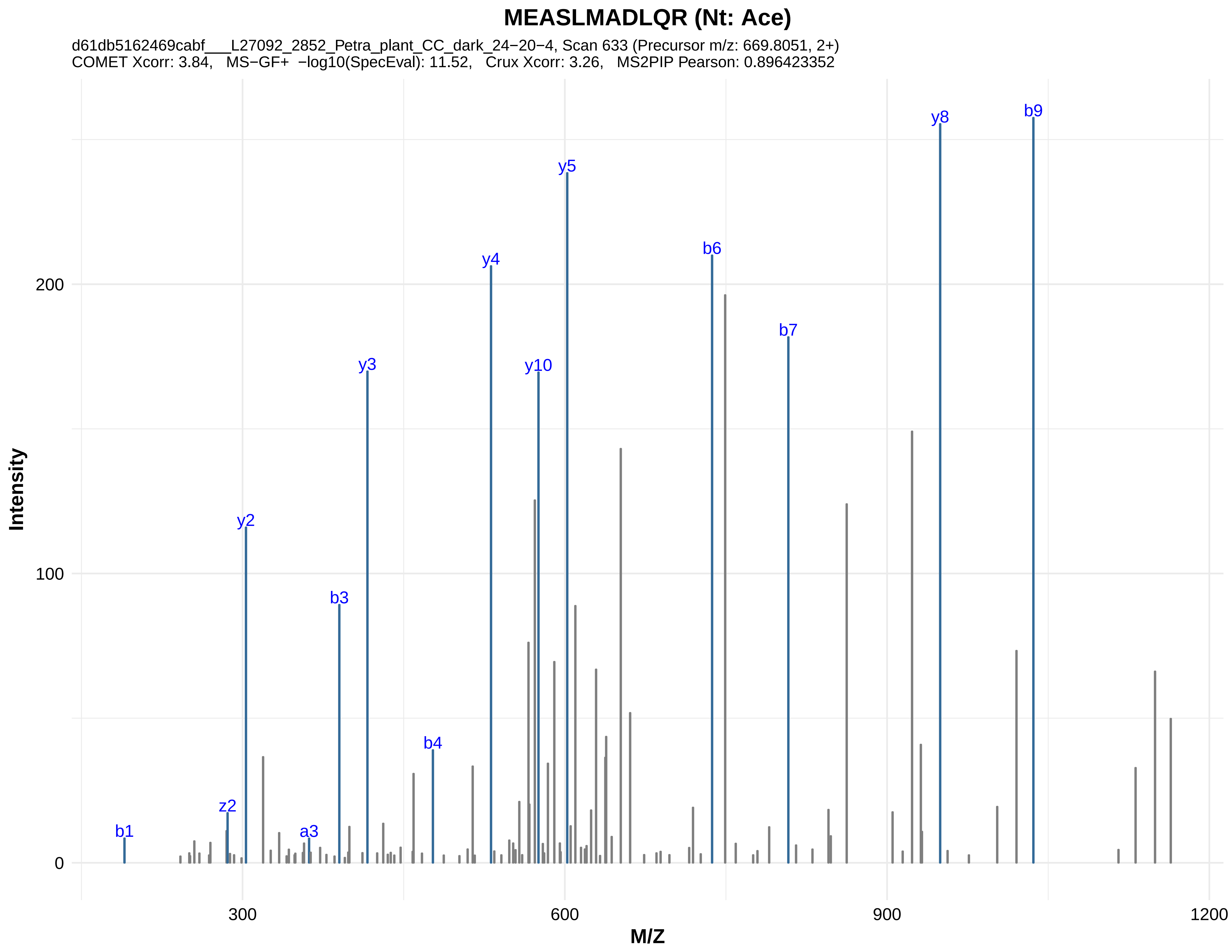

MEASLMADLQR (Nt: Ace)
d61db5162469cabf___L27092_2852_Petra_plant_CC_dark_24−20−4, Scan 633 (Precursor m/z: 669.8051, 2+)
COMET Xcorr: 3.84, MS−GF+ −log10(SpecEval): 11.52, Crux Xcorr: 3.26, MS2PIP Pearson: 0.896423352
Intensity
M/Z
0
100
200
300
600
900
1200
b1
z2
y2
a3
b3
y3
b4
y4
y10
y5
b6
b7
y8
b9

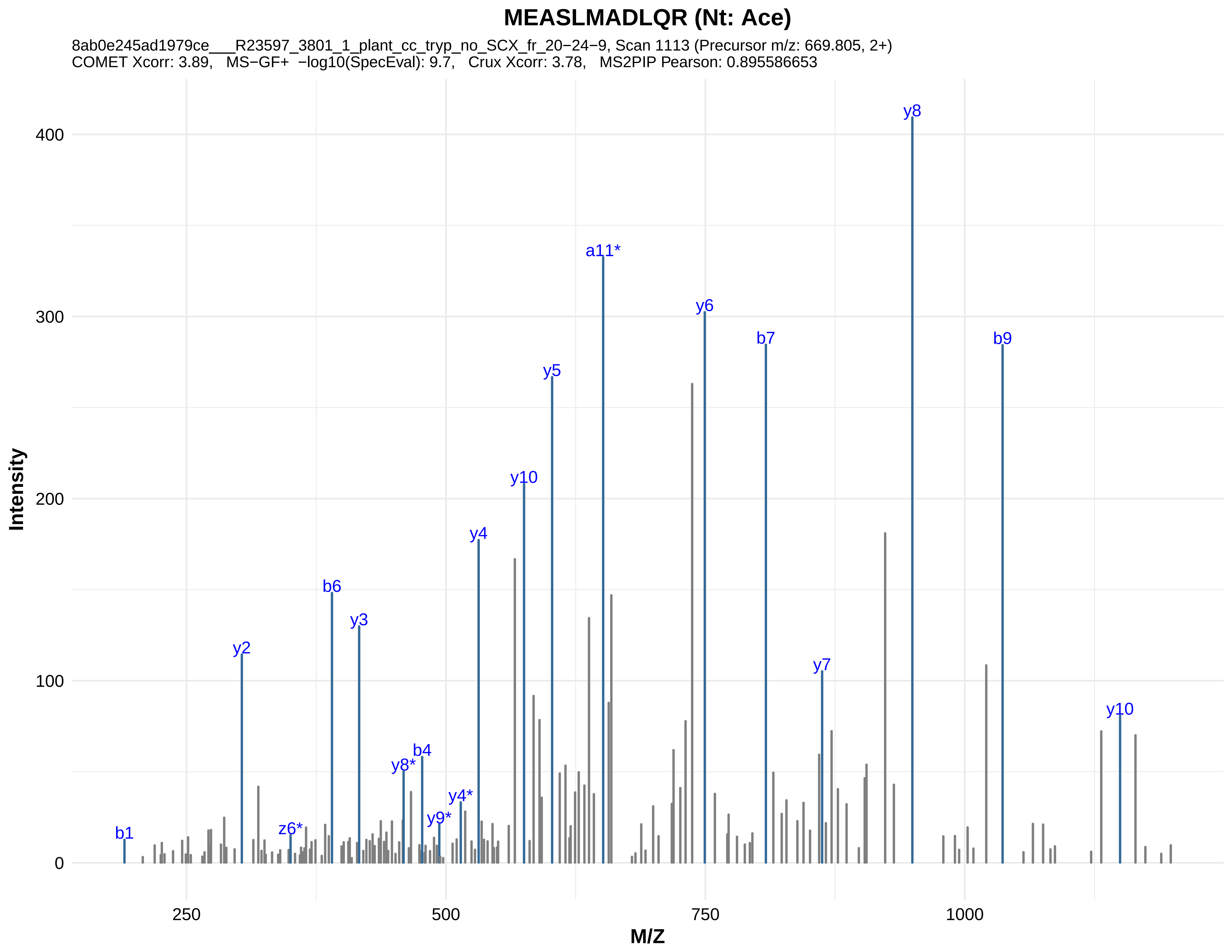
MEASLMADLQR (Nt: Ace)
8ab0e245ad1979ce___R23597_3801_1_plant_cc_tryp_no_SCX_fr_20−24−9, Scan 1113 (Precursor m/z: 669.805, 2+)
COMET Xcorr: 3.89, MS−GF+ −log10(SpecEval): 9.7, Crux Xcorr: 3.78, MS2PIP Pearson: 0.895586653
Intensity
M/Z
0
100
200
300
400
250
500
750
1000
b1
y2
z6*
b6
y3
y8*
b4
y9*
y4*
y4
y10
y5
a11*
y6
b7
y7
y8
b9
y10

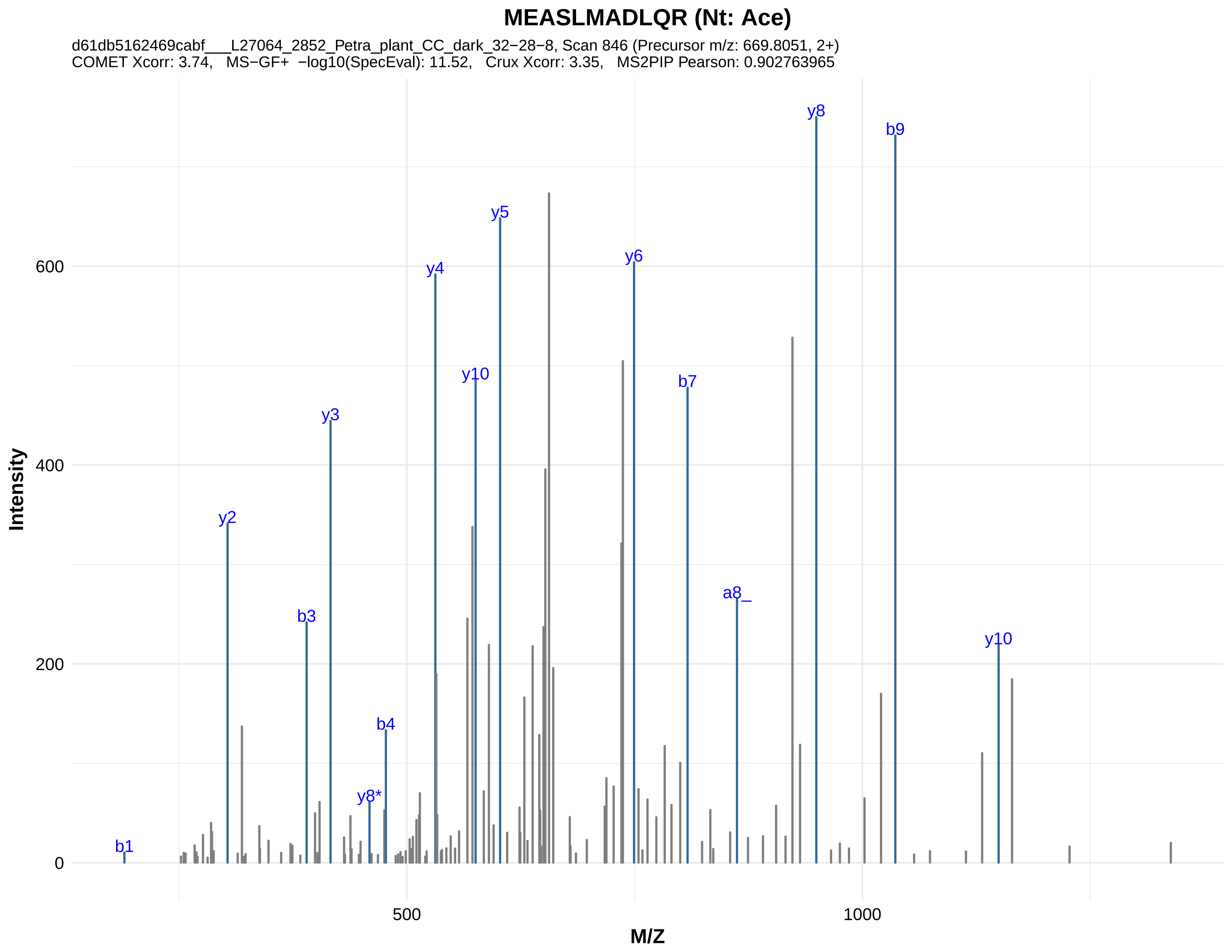
MEASLMADLQR (Nt: Ace)
d61db5162469cabf___L27064_2852_Petra_plant_CC_dark_32−28−8, Scan 846 (Precursor m/z: 669.8051, 2+)
COMET Xcorr: 3.74,   MS−GF+ −log10(SpecEval): 11.52,   Crux Xcorr: 3.35,   MS2PIP Pearson: 0.902763965
y8
b9
y5
y4
y6
y10
b7
y3
y2
b3
a8_
y10
b4
y8*
b1
Intensity
600
400
200
0
500
1000
M/Z

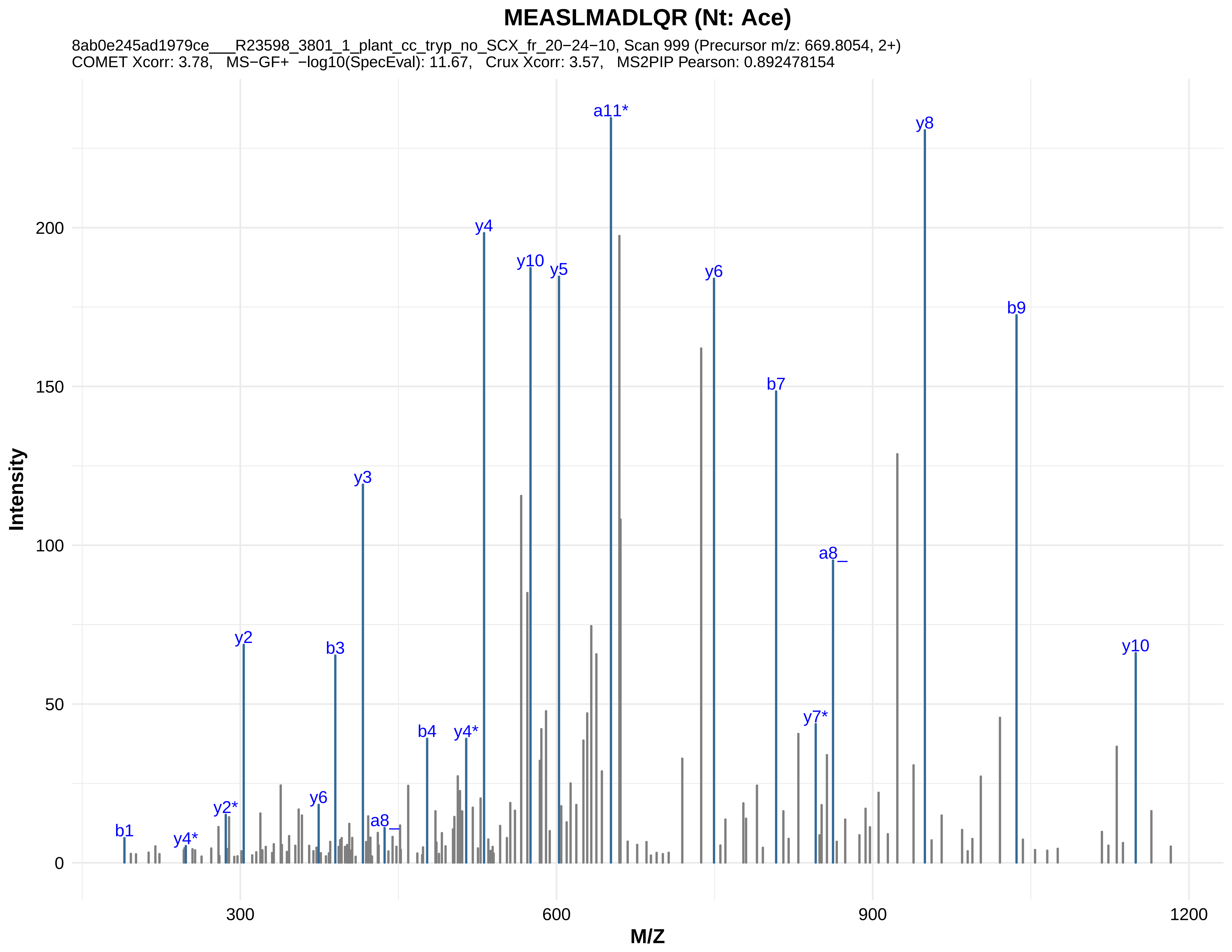

MEASLMADLQR (Nt: Ace)
8ab0e245ad1979ce___R23598_3801_1_plant_cc_tryp_no_SCX_fr_20−24−10, Scan 999 (Precursor m/z: 669.8054, 2+)
COMET Xcorr: 3.78, MS−GF+ −log10(SpecEval): 11.67, Crux Xcorr: 3.57, MS2PIP Pearson: 0.892478154
Intensity
M/Z
a11*
y8
y4
y10
y5
y6
b9
b7
y3
a8_
y2
b3
y10
y7*
b4
y4*
y6
y2*
a8_
b1
y4*

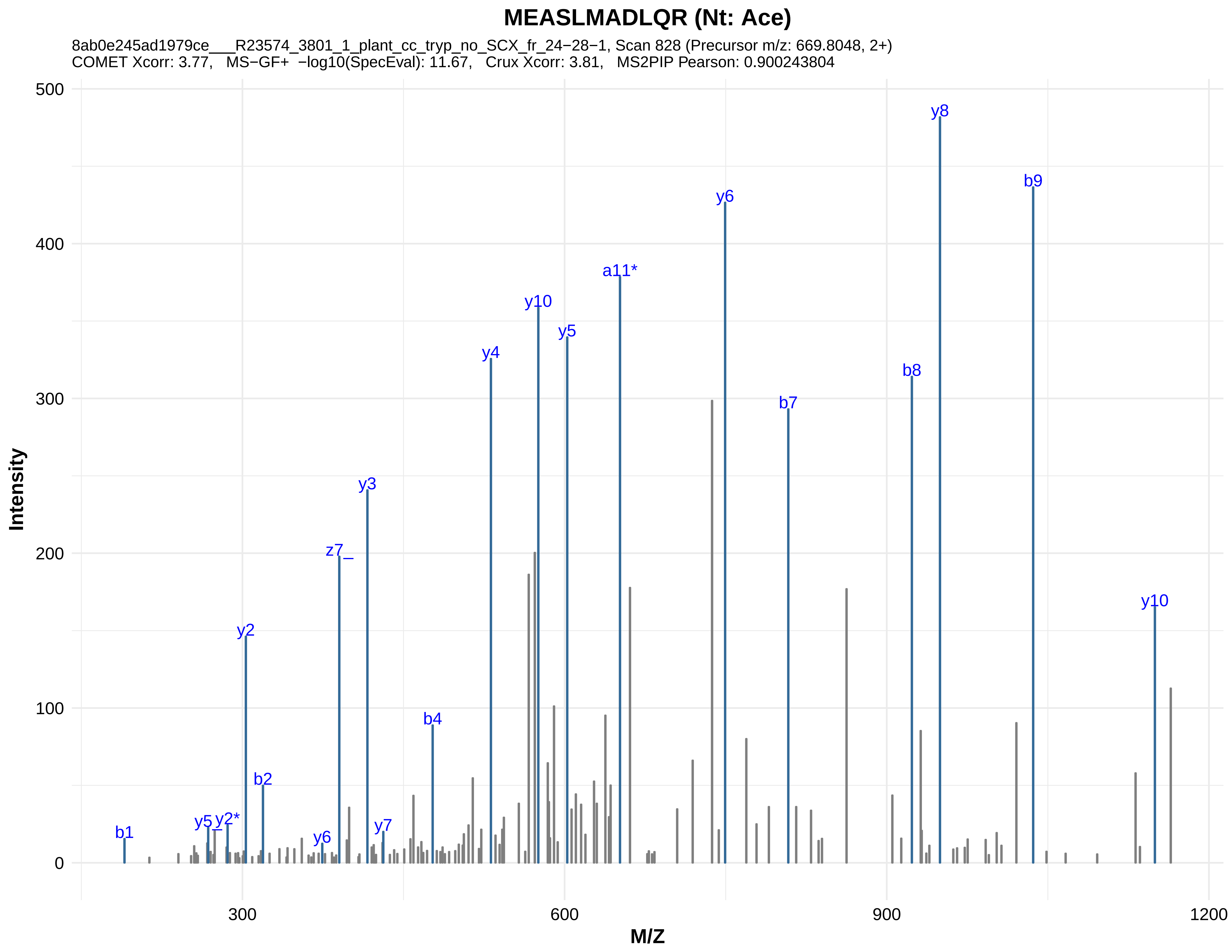

MEASLMADLQR (Nt: Ace)
8ab0e245ad1979ce___R23574_3801_1_plant_cc_tryp_no_SCX_fr_24−28−1, Scan 828 (Precursor m/z: 669.8048, 2+)
COMET Xcorr: 3.77, MS−GF+ −log10(SpecEval): 11.67, Crux Xcorr: 3.81, MS2PIP Pearson: 0.900243804
Intensity
M/Z
0
100
200
300
400
500
300
600
900
1200
b1
y5_
y2*
y2
b2
y6
z7_
y3
y7
b4
y4
y10
y5
a11*
y6
b7
b8
y8
b9
y10

MEEGTGFSLGR (Nt: Ace)

d61db5162469cabf___L27095_2852_Petra_plant_CC_dark_24−20−7_130812093233, Scan 1009 (Precursor m/z: 621.27, 2+)
COMET Xcorr: 3.48,   MS−GF+ −log10(SpecEval): 13.19,   Crux Xcorr: 2.91,   MS2PIP Pearson: 0.811084938

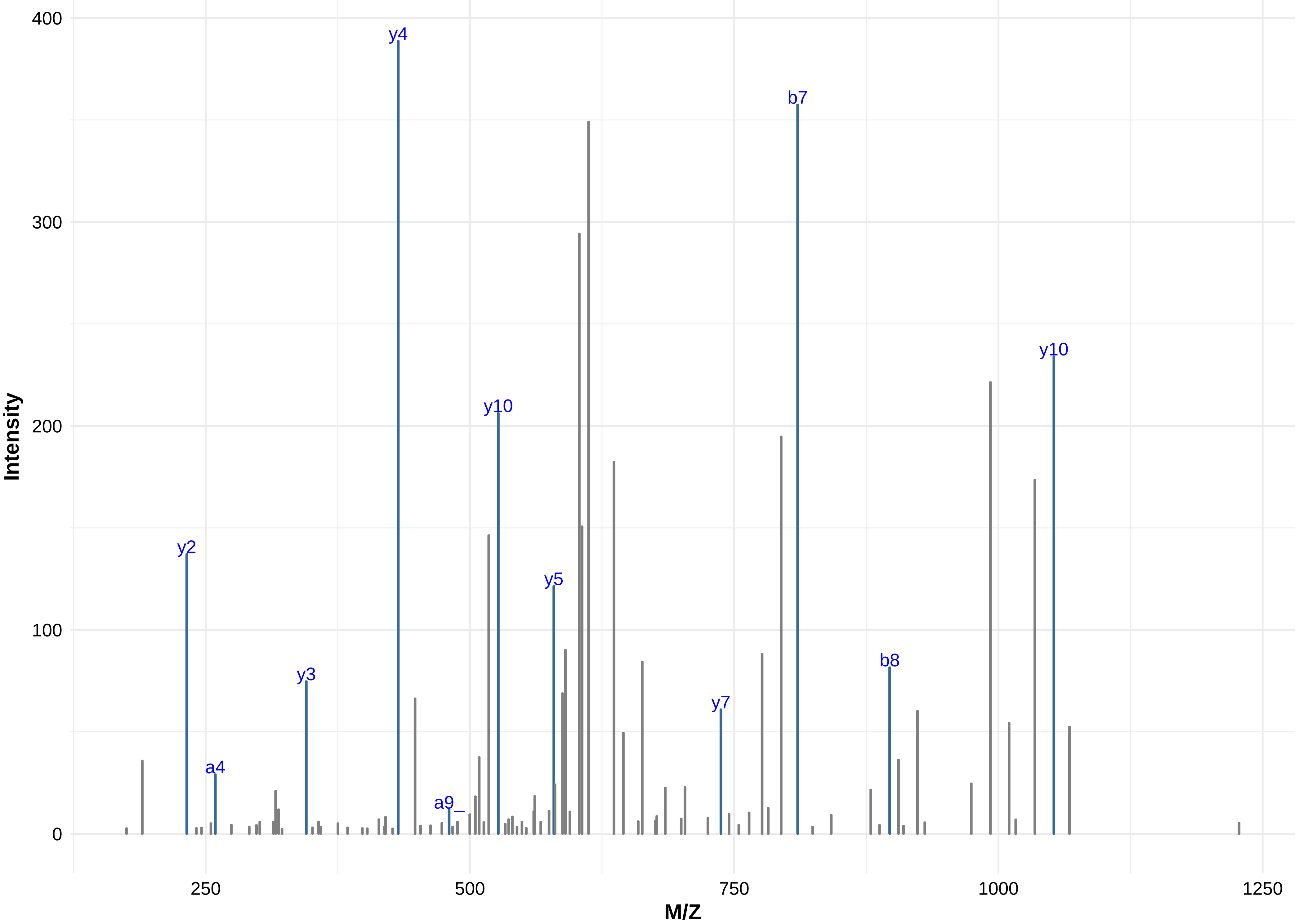

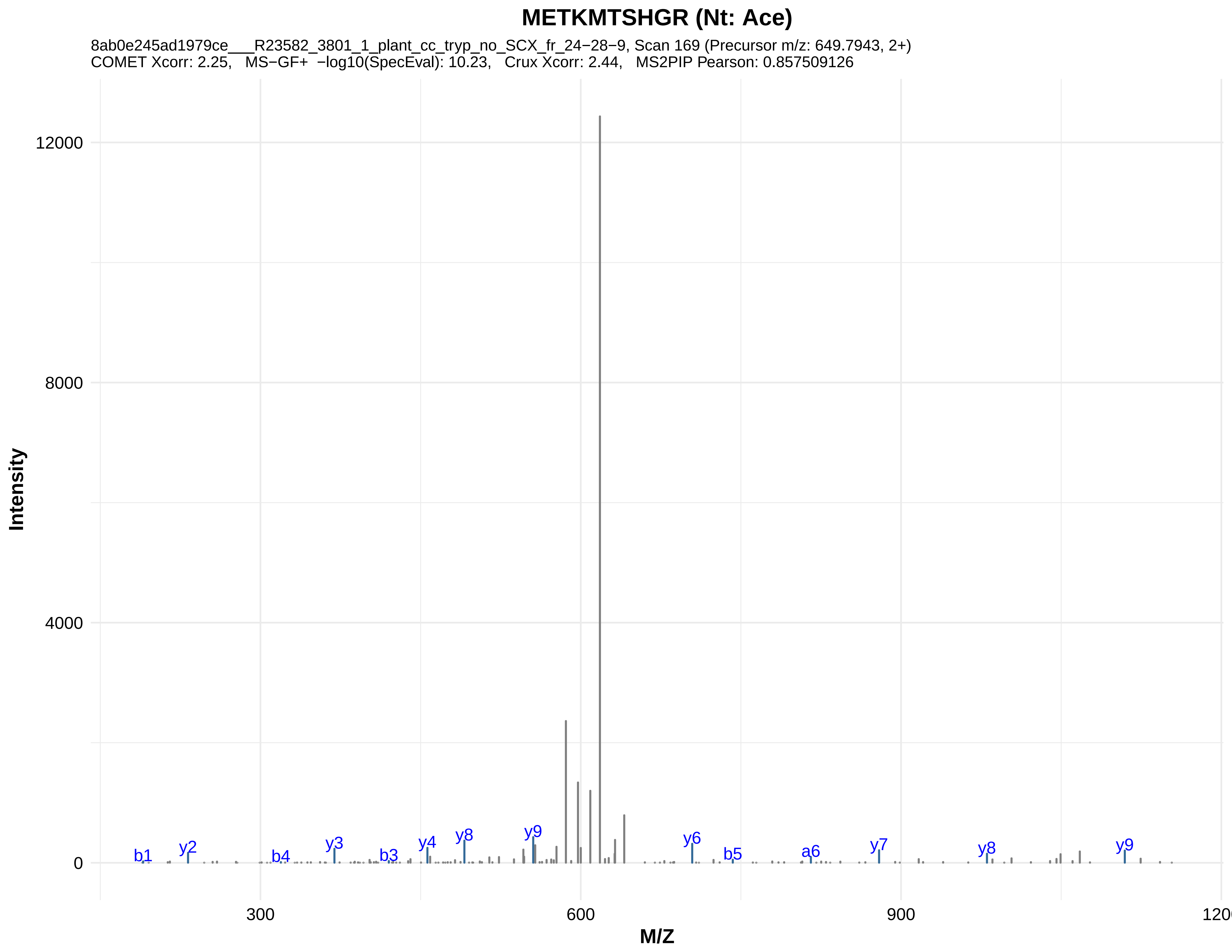

METKMTSHGR (Nt: Ace)
8ab0e245ad1979ce___R23582_3801_1_plant_cc_tryp_no_SCX_fr_24−28−9, Scan 169 (Precursor m/z: 649.7943, 2+)
COMET Xcorr: 2.25, MS−GF+ −log10(SpecEval): 10.23, Crux Xcorr: 2.44, MS2PIP Pearson: 0.857509126
Intensity
12000
8000
4000
0
b1
y2
b4
y3
b3
y4
y8
y9
y6
b5
a6
y7
y8
y9
300
600
900
M/Z

MLDLNIYR (Nt: Trideutero)

d61db5162469cabf___L27074_2852_Petra_plant_CC_dark_28−24−2, Scan 788 (Precursor m/z: 550.791, 2+)
COMET Xcorr: 2.64,   MS−GF+ −log10(SpecEval): 8.69,   Crux Xcorr: 2.59,   MS2PIP Pearson: 0.811971289

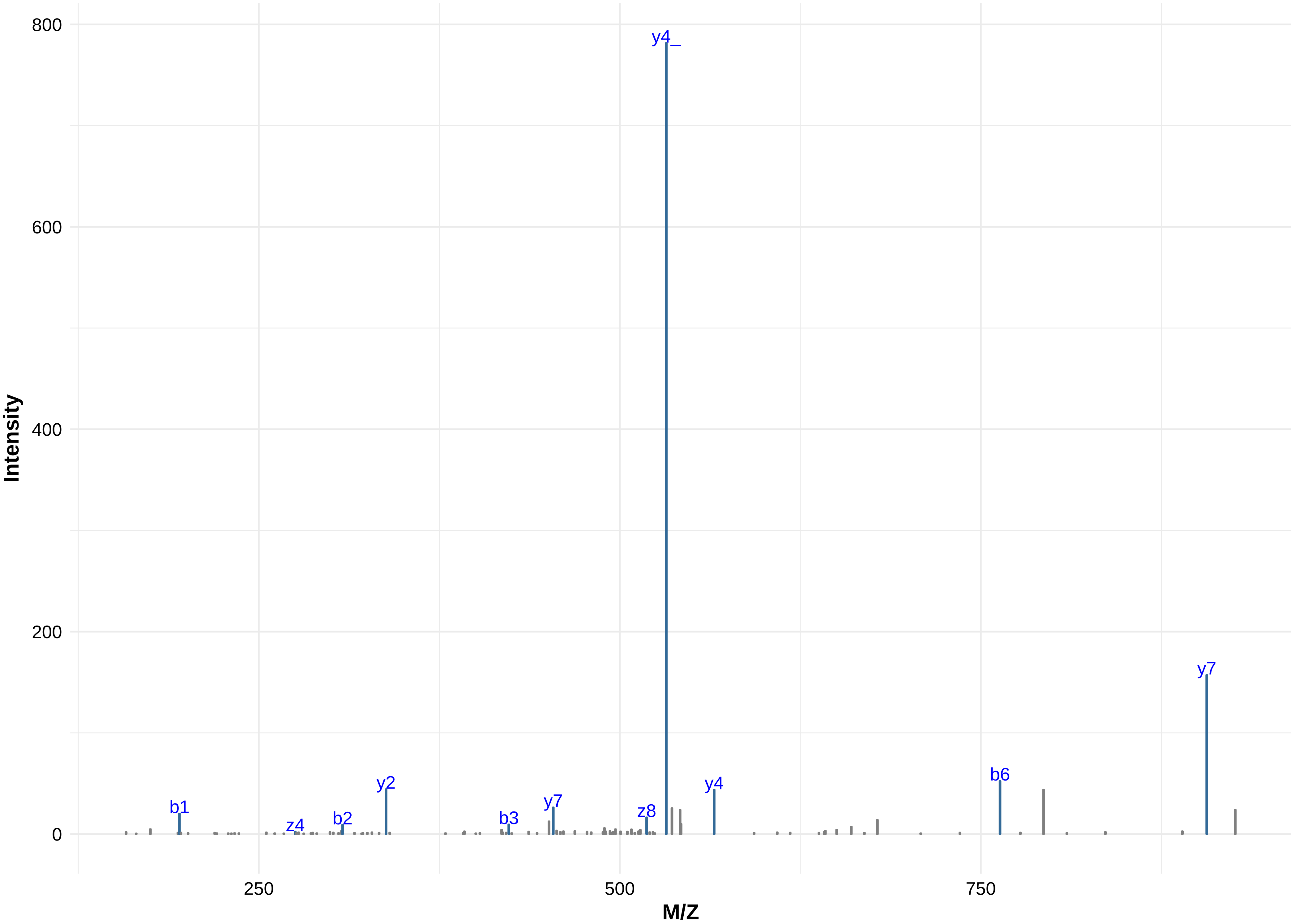

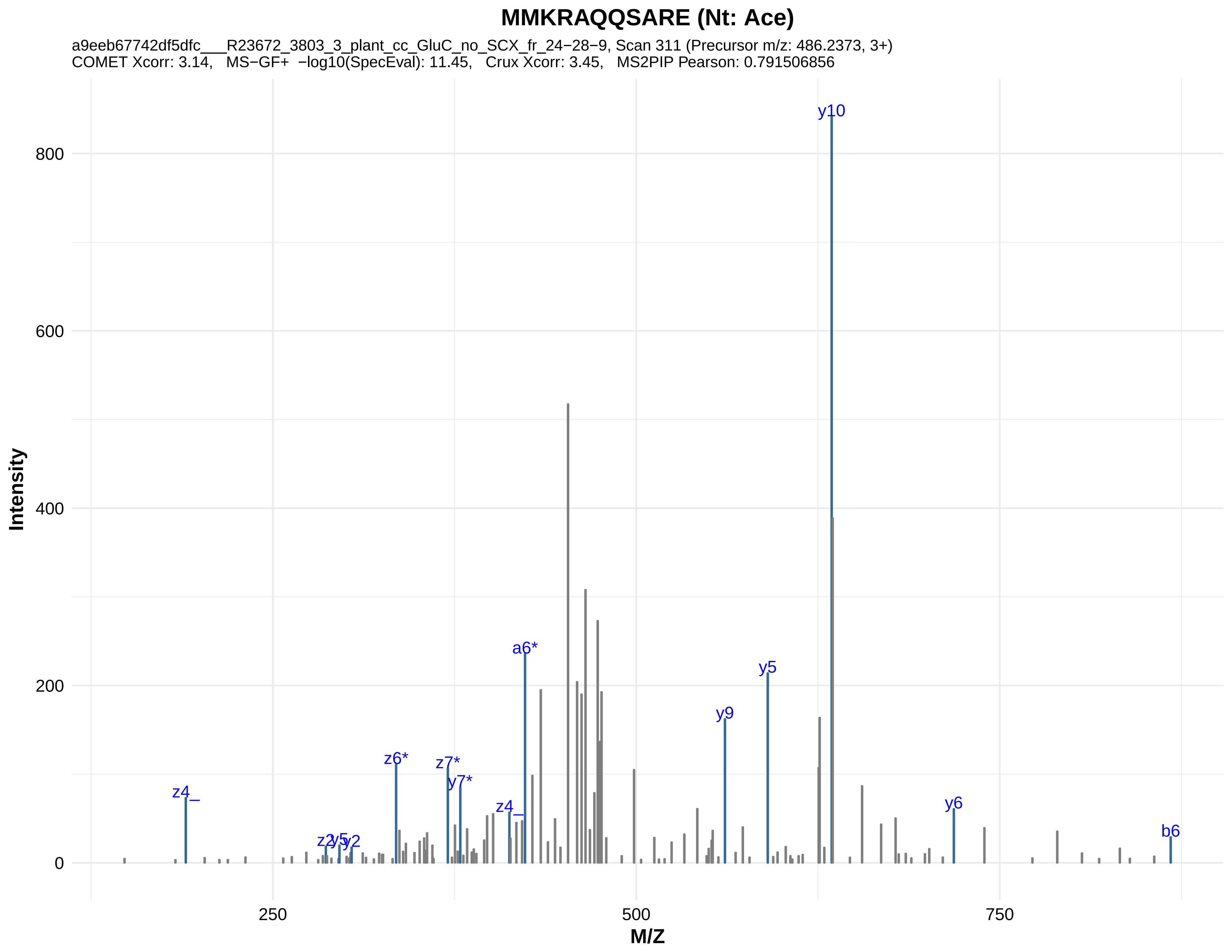
MMKRAQQSARE (Nt: Ace)
a9eeb67742df5dfc___R23672_3803_3_plant_cc_GluC_no_SCX_fr_24−28−9, Scan 311 (Precursor m/z: 486.2373, 3+)
COMET Xcorr: 3.14, MS−GF+ −log10(SpecEval): 11.45, Crux Xcorr: 3.45, MS2PIP Pearson: 0.791506856
Intensity
M/Z
y10
a6*
y5
y9
z6*
z7*
y7*
z4_
z4_
z2
y5
y2
y6
b6

MMPEKTIISSESSNSSPLNPSSTR (Nt: Ace)

d61db5162469cabf___L27081_2852_Petra_plant_CC_dark_28−24−9, Scan 948 (Precursor m/z: 901.0932, 3+)
COMET Xcorr: 3.05,   MS−GF+ −log10(SpecEval): 11.68,   Crux Xcorr: 3.5,   MS2PIP Pearson: 0.680375617

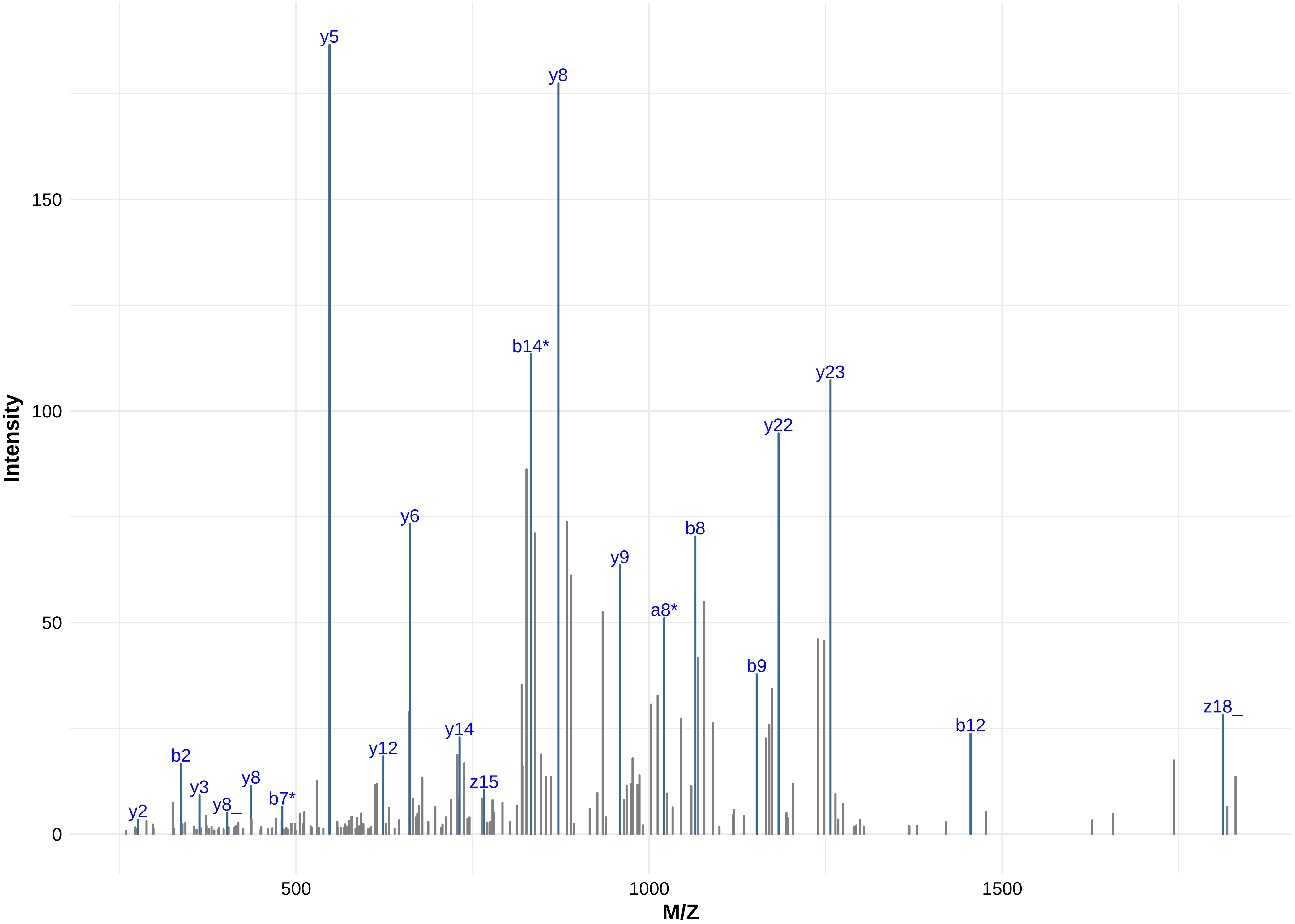

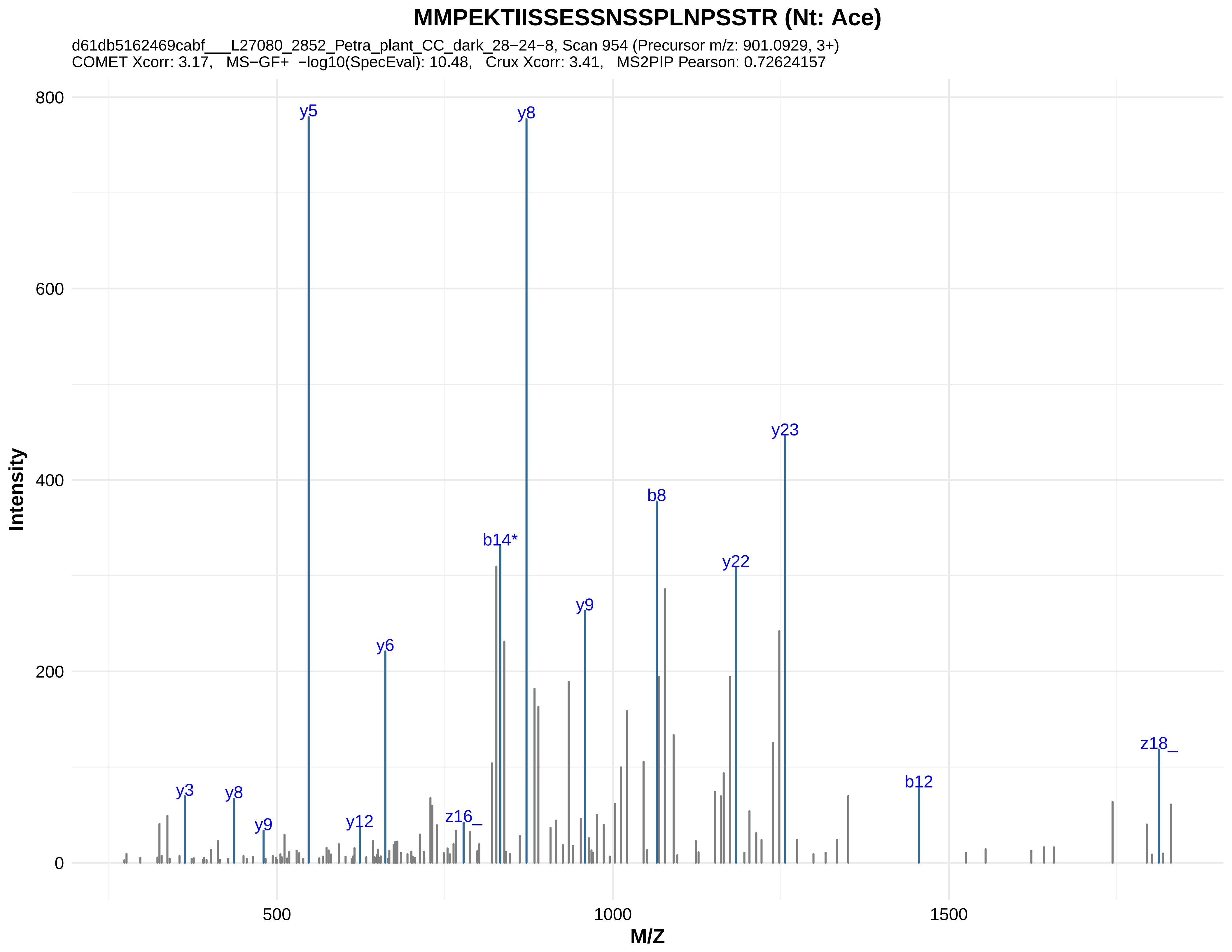

MMPEKTIISSESSNSSPLNPSSTR (Nt: Ace)
d61db5162469cabf___L27080_2852_Petra_plant_CC_dark_28−24−8, Scan 954 (Precursor m/z: 901.0929, 3+)
COMET Xcorr: 3.17, MS−GF+ −log10(SpecEval): 10.48, Crux Xcorr: 3.41, MS2PIP Pearson: 0.72624157
Intensity
M/Z
0
200
400
600
800
500
1000
1500
y5
y8
y23
b8
b14*
y22
y9
y6
z18_
b12
y3
y8
y9
y12
z16_

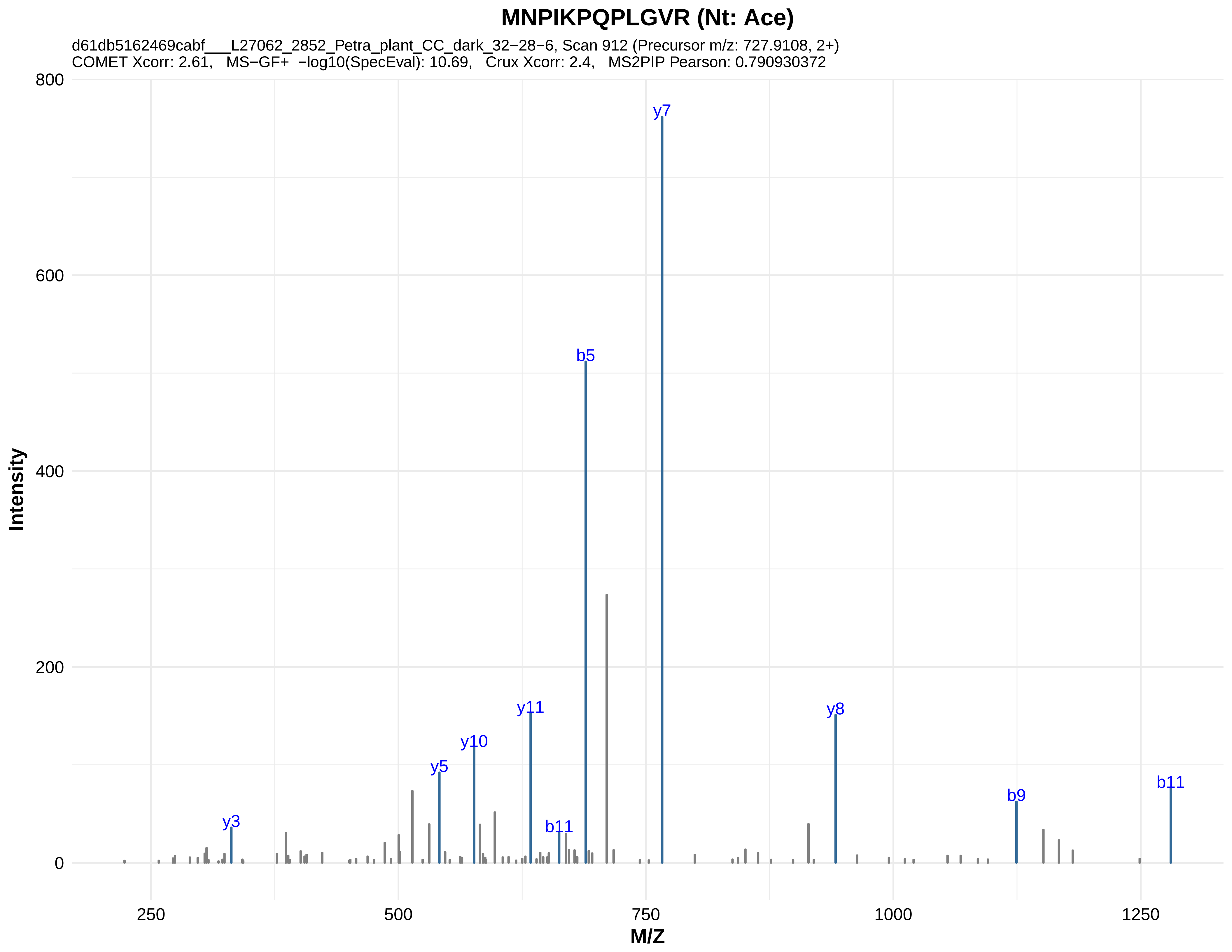

MNPIKPQPLGVR (Nt: Ace)
d61db5162469cabf___L27062_2852_Petra_plant_CC_dark_32−28−6, Scan 912 (Precursor m/z: 727.9108, 2+)
COMET Xcorr: 2.61, MS−GF+ −log10(SpecEval): 10.69, Crux Xcorr: 2.4, MS2PIP Pearson: 0.790930372
y7
b5
y11
y8
y10
y5
b11
b9
y3
b11
Intensity
M/Z
0
200
400
600
800
250
500
750
1000
1250

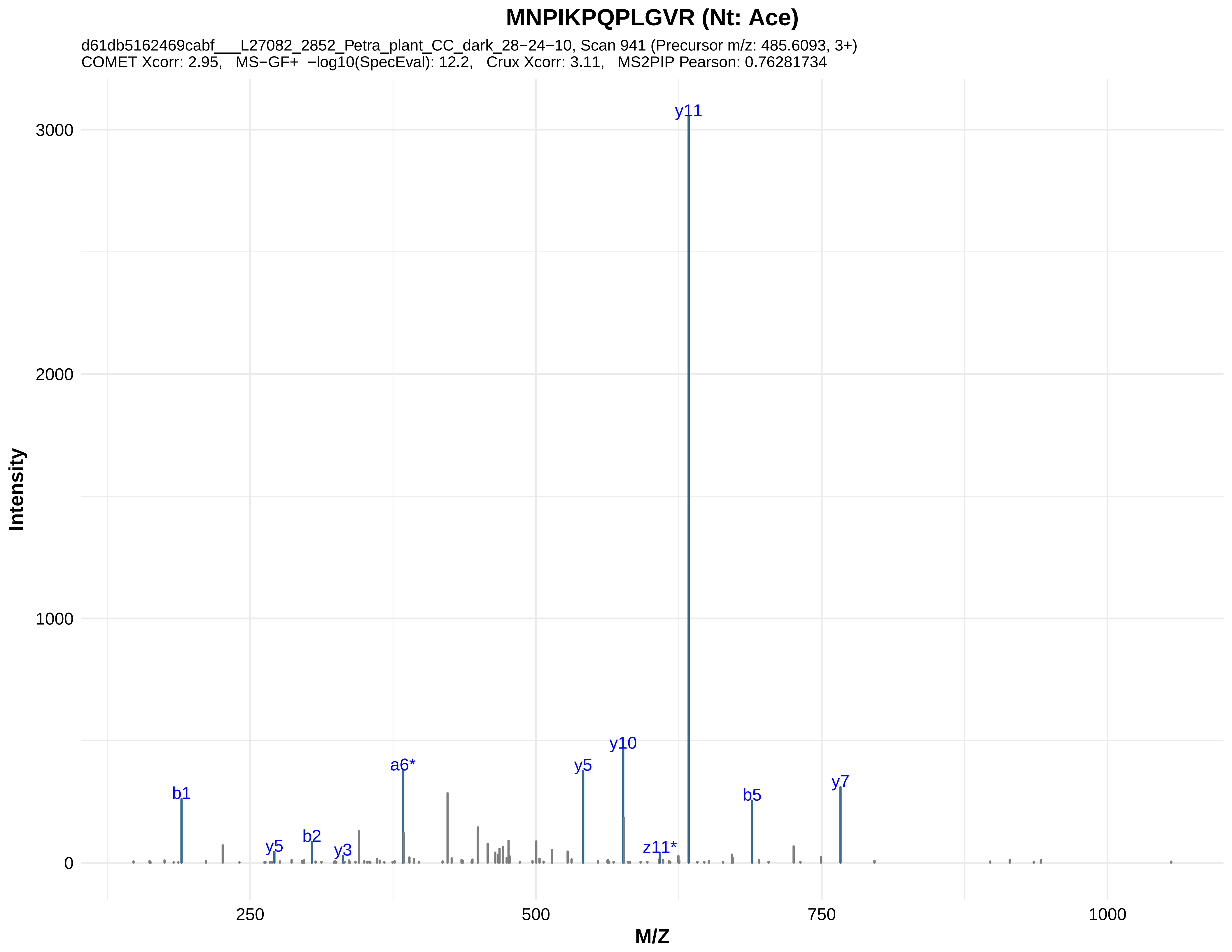
MNPIKPQPLGVR (Nt: Ace)
d61db5162469cabf___L27082_2852_Petra_plant_CC_dark_28−24−10, Scan 941 (Precursor m/z: 485.6093, 3+)
COMET Xcorr: 2.95, MS−GF+ −log10(SpecEval): 12.2, Crux Xcorr: 3.11, MS2PIP Pearson: 0.76281734
Intensity
M/Z
0
1000
2000
3000
250
500
750
1000
y11
y10
a6*
y5
y7
b5
b1
b2
y5
y3
z11*

MNQRGKSNFE (Nt: Ace)

a9eeb67742df5dfc___R23679_3803_3_plant_cc_GluC_no_SCX_fr_20−24−1, Scan 718 (Precursor m/z: 658.3059, 2+)
COMET Xcorr: 2.21, MS−GF+ −log10(SpecEval): 9.38, Crux Xcorr: 2.32, MS2PIP Pearson: 0.806129479

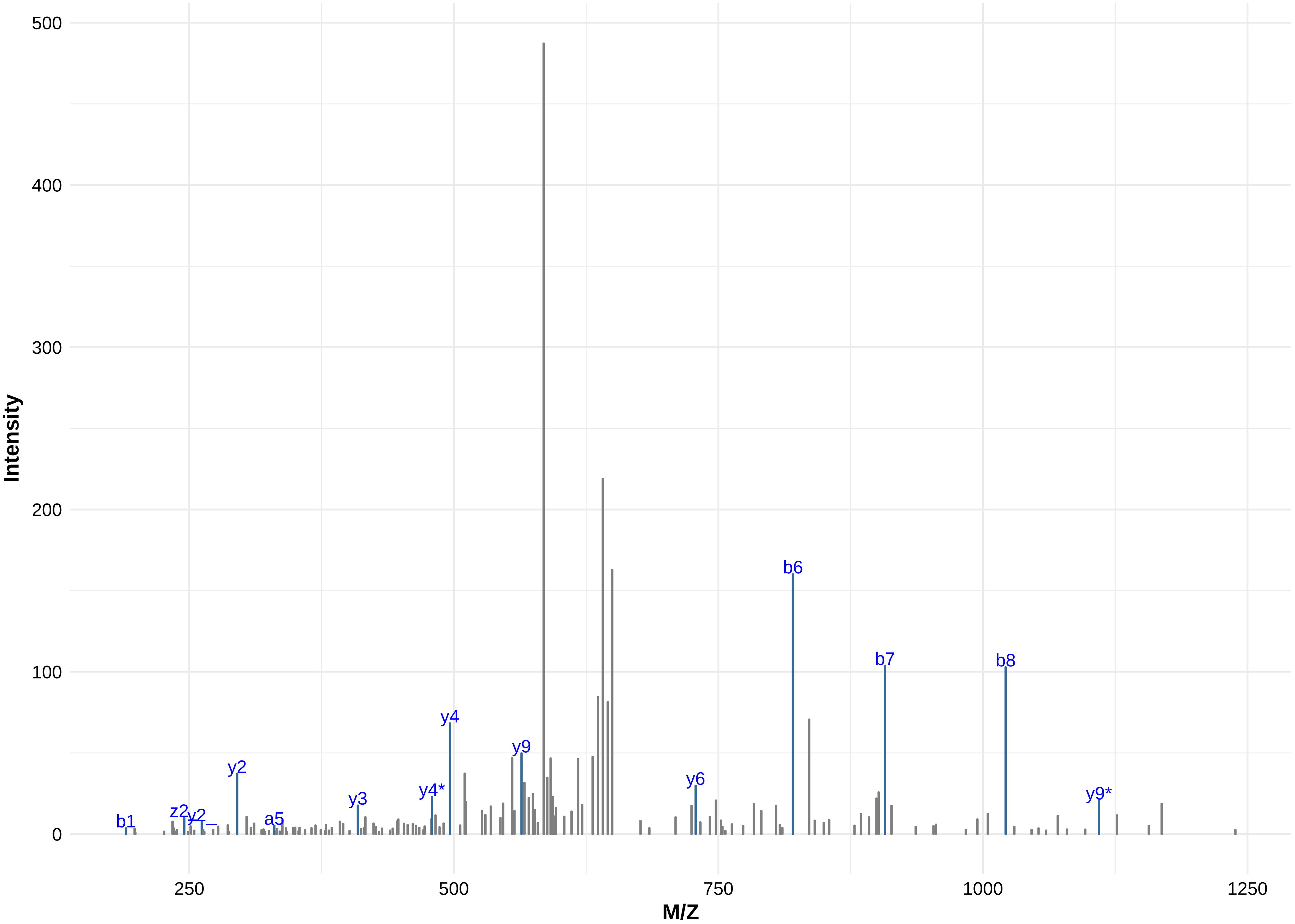

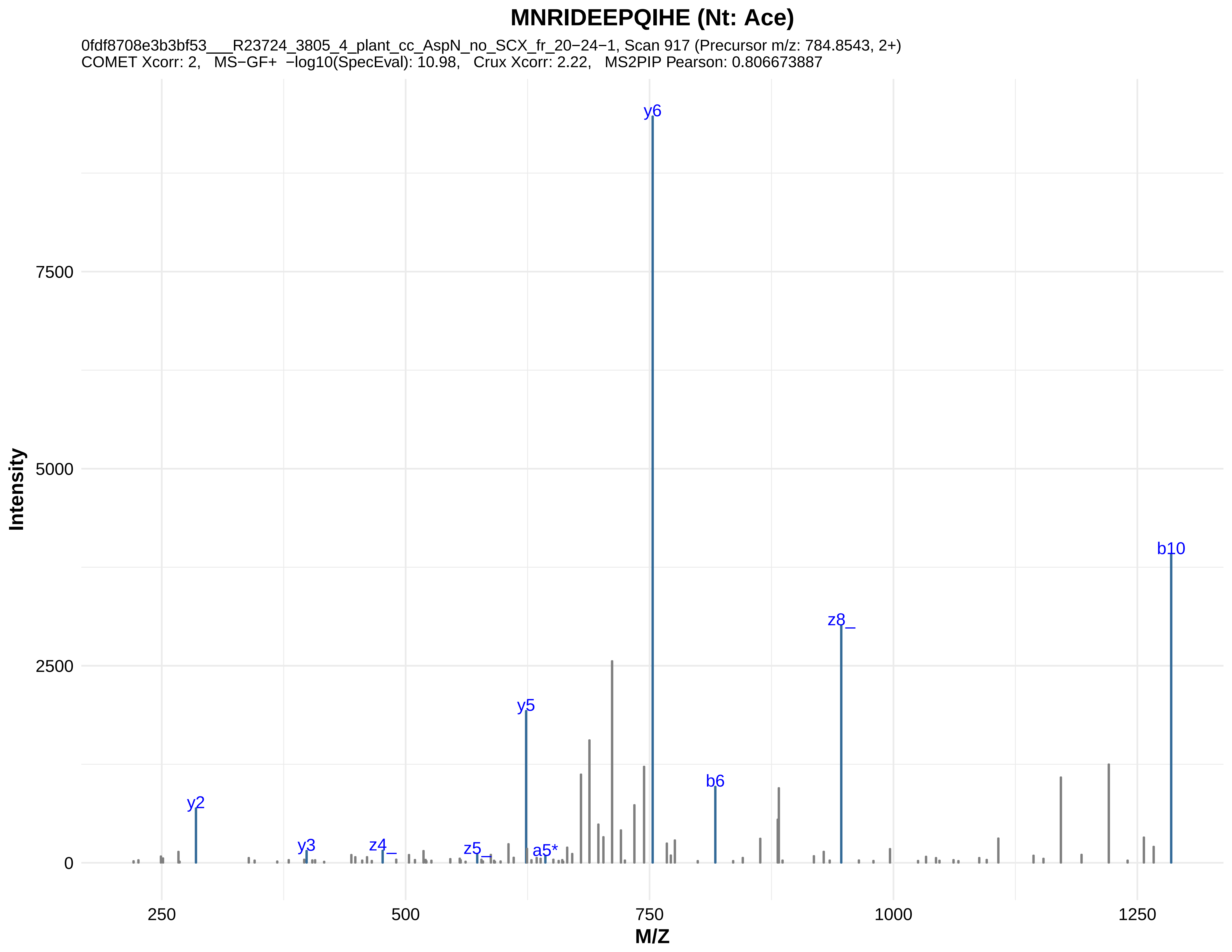
MNRIDEEPQIHE (Nt: Ace)
0fdf8708e3b3bf53___R23724_3805_4_plant_cc_AspN_no_SCX_fr_20−24−1, Scan 917 (Precursor m/z: 784.8543, 2+)
COMET Xcorr: 2, MS−GF+ −log10(SpecEval): 10.98, Crux Xcorr: 2.22, MS2PIP Pearson: 0.806673887
Intensity
M/Z
y6
b10
z8_
y5
b6
y2
y3
z4_
z5_
a5*
0
2500
5000
7500
250
500
750
1000
1250

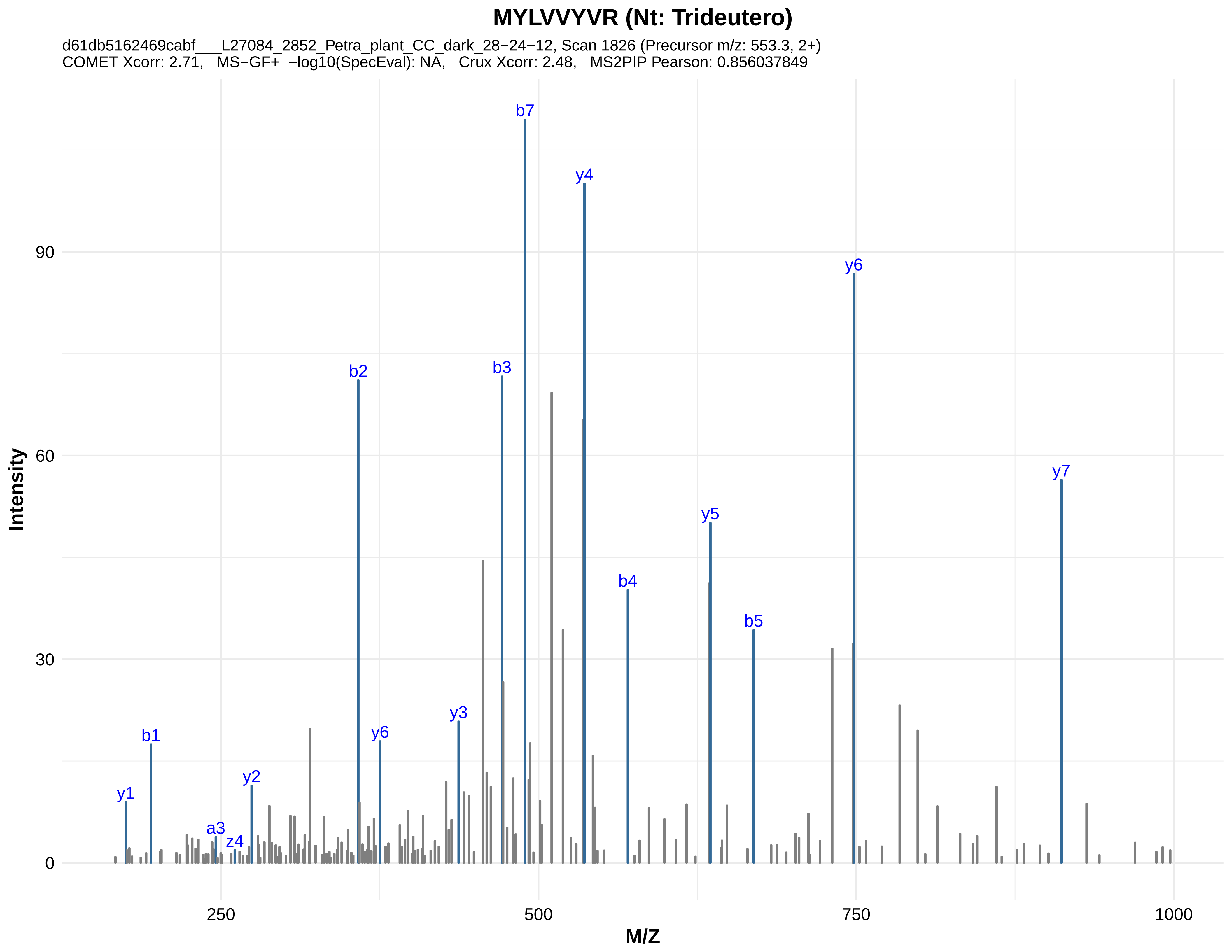

MYLVVYVR (Nt: Trideutero)
d61db5162469cabf___L27084_2852_Petra_plant_CC_dark_28−24−12, Scan 1826 (Precursor m/z: 553.3, 2+)
COMET Xcorr: 2.71, MS−GF+ −log10(SpecEval): NA, Crux Xcorr: 2.48, MS2PIP Pearson: 0.856037849
Intensity
M/Z
0
30
60
90
250
500
750
1000
y1
b1
a3
z4
y2
b2
y6
y3
b3
b7
y4
b4
y5
b5
y6
y7

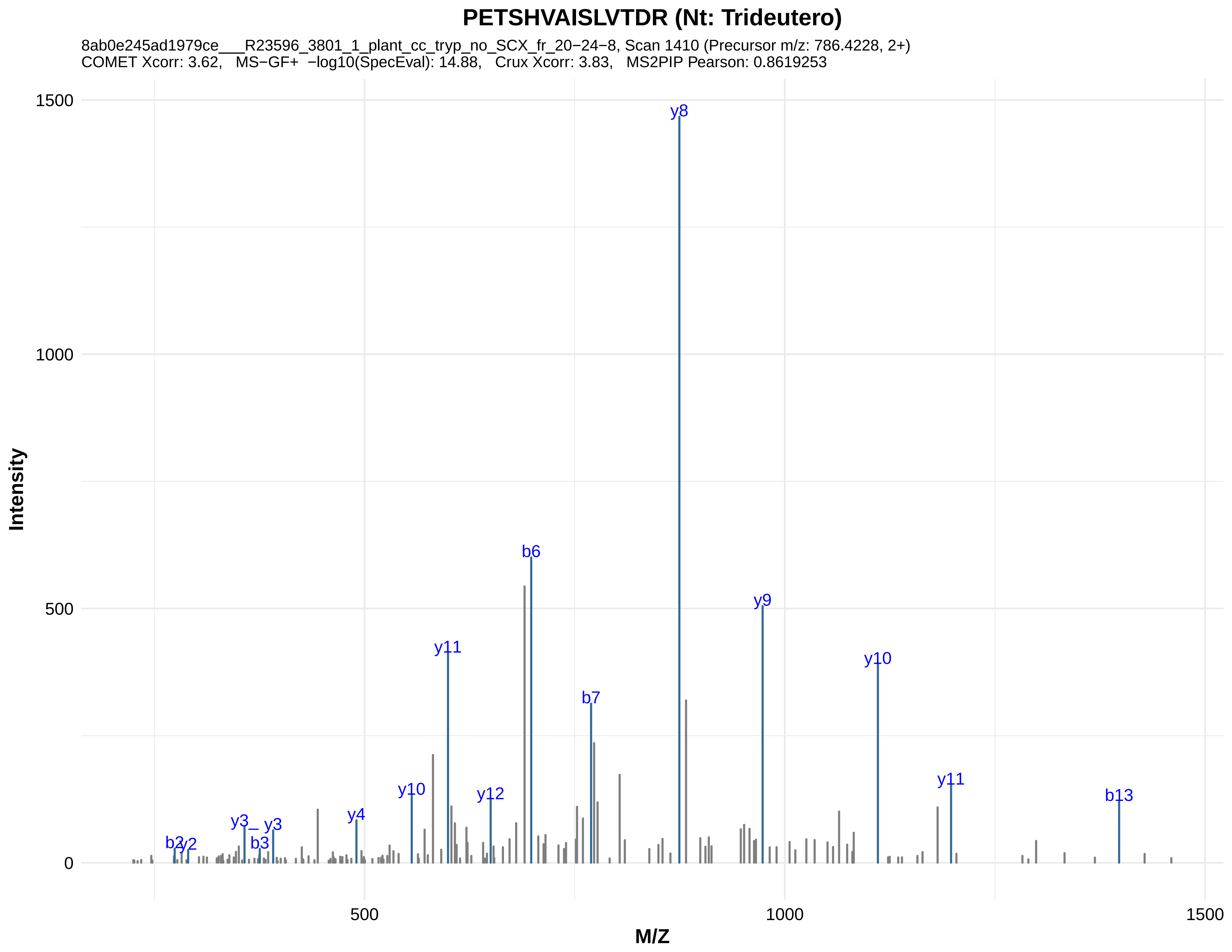
PETSHVAISLVTDR (Nt: Trideutero)
8ab0e245ad1979ce___R23596_3801_1_plant_cc_tryp_no_SCX_fr_20−24−8, Scan 1410 (Precursor m/z: 786.4228, 2+)
COMET Xcorr: 3.62, MS−GF+ −log10(SpecEval): 14.88, Crux Xcorr: 3.83, MS2PIP Pearson: 0.8619253
Intensity
M/Z
0
500
1000
1500
y8
b6
y9
y11
y10
b7
y10
y12
y11
b13
y4
y3
y3_
y3
b3
b2
y2

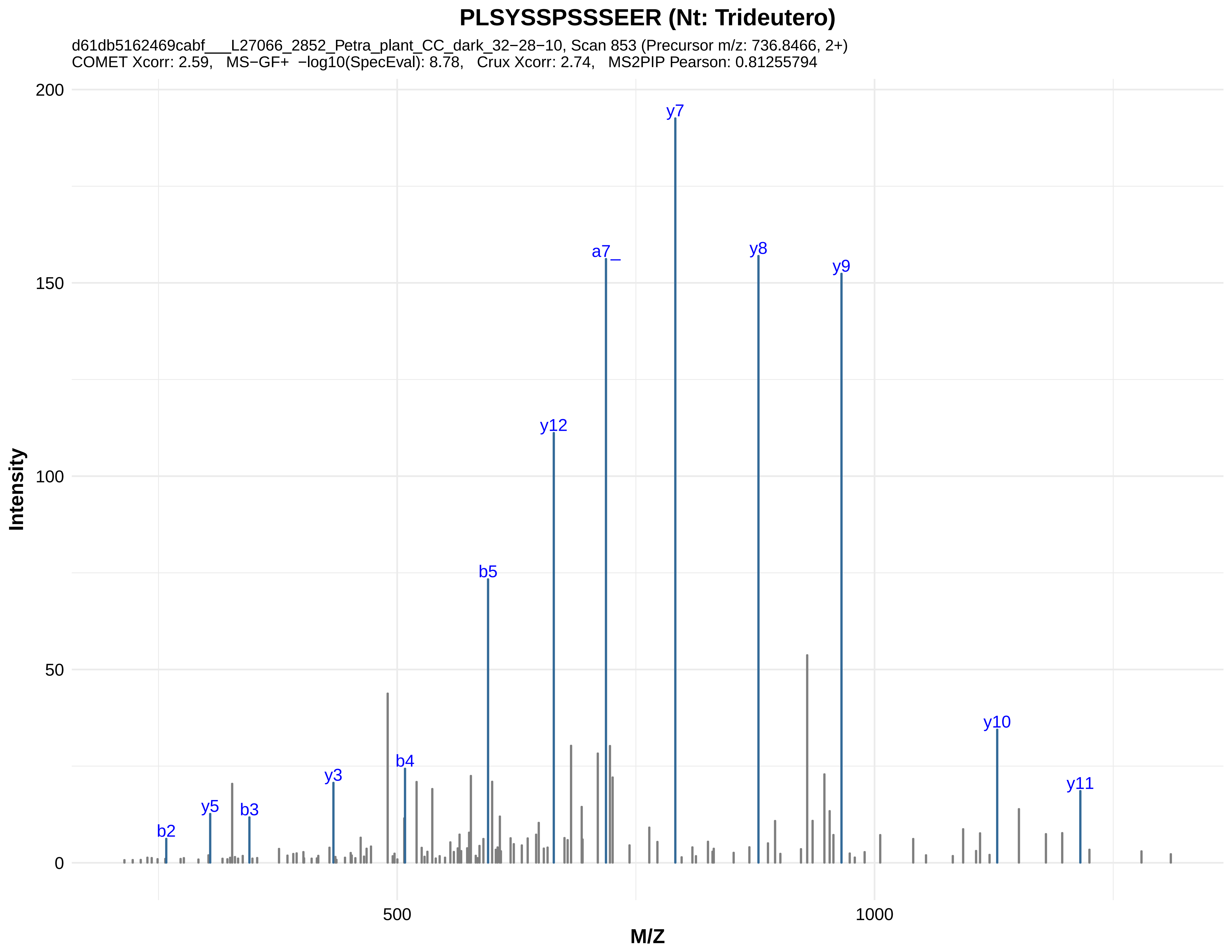
PLSYSSPSSSEER (Nt: Trideutero)
d61db5162469cabf___L27066_2852_Petra_plant_CC_dark_32−28−10, Scan 853 (Precursor m/z: 736.8466, 2+)
COMET Xcorr: 2.59, MS−GF+ −log10(SpecEval): 8.78, Crux Xcorr: 2.74, MS2PIP Pearson: 0.81255794
Intensity
M/Z
0
50
100
150
200
500
1000
y7
a7_
y8
y9
y12
b5
y10
y11
b4
y3
y5
b3
b2

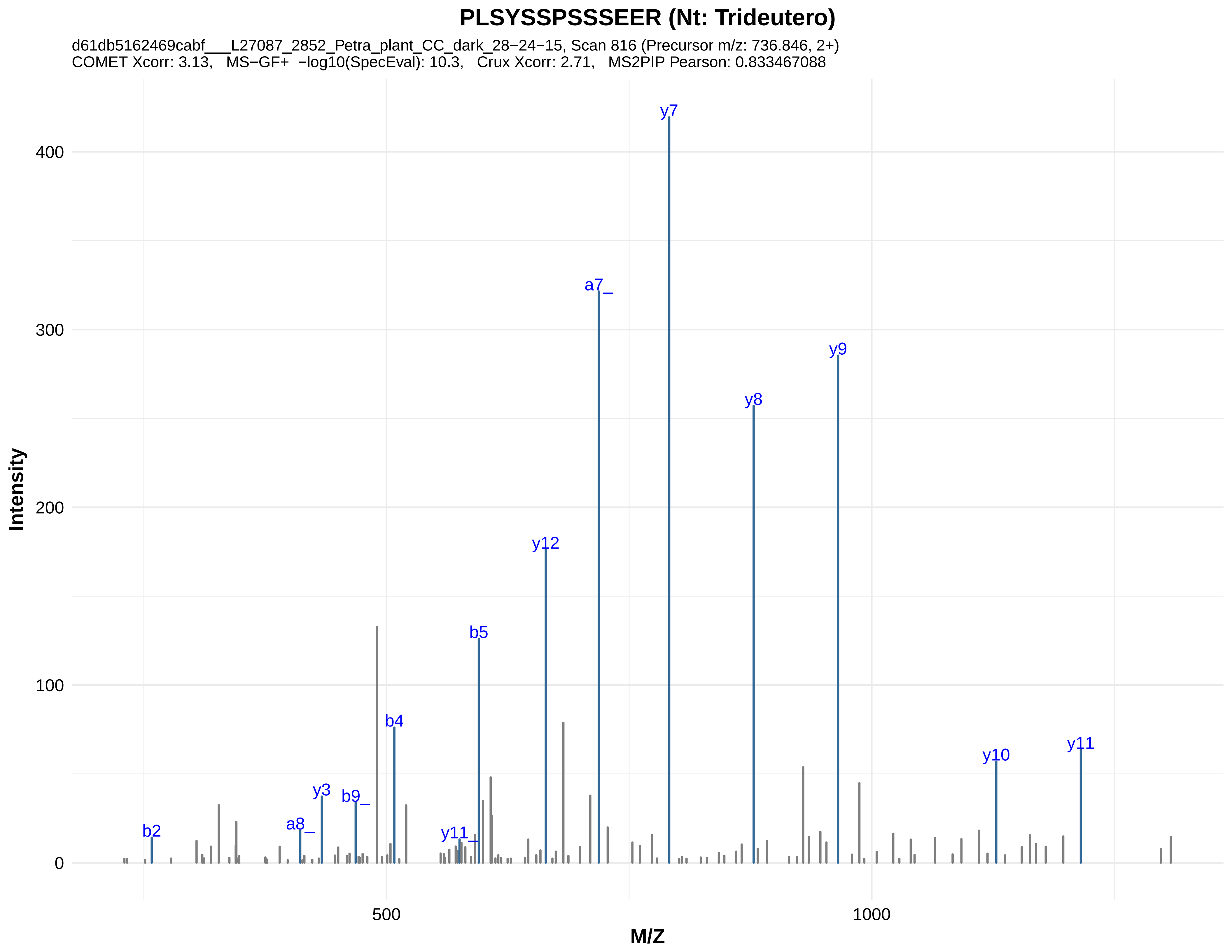

PLSYSSPSSSEER (Nt: Trideutero)
d61db5162469cabf___L27087_2852_Petra_plant_CC_dark_28−24−15, Scan 816 (Precursor m/z: 736.846, 2+)
COMET Xcorr: 3.13, MS−GF+ −log10(SpecEval): 10.3, Crux Xcorr: 2.71, MS2PIP Pearson: 0.833467088
Intensity
M/Z
0
100
200
300
400
500
1000
y7
a7_
y9
y8
y12
b5
b4
y11
y10
y3
b9_
a8_
b2
y11_

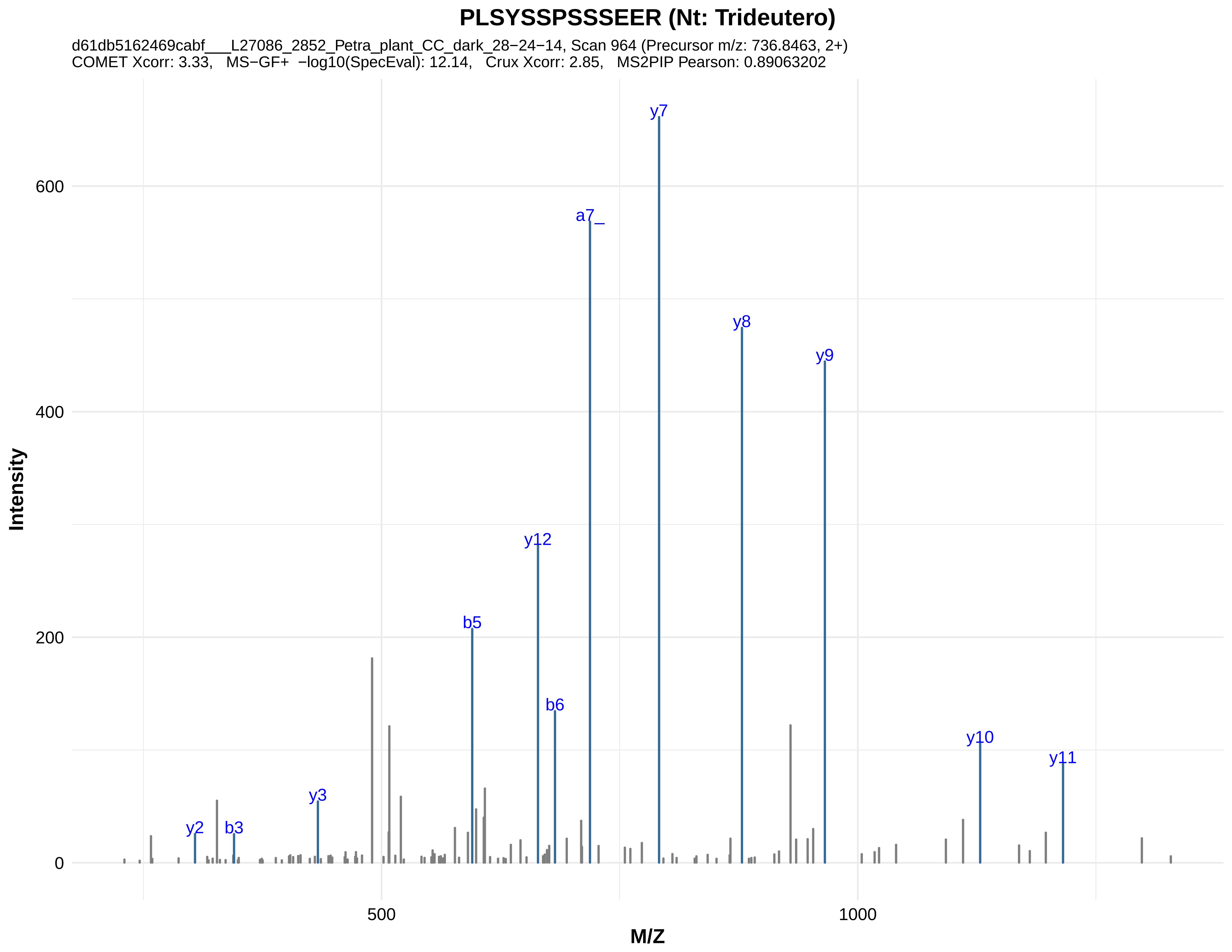
PLSYSSPSSSEER (Nt: Trideutero)
d61db5162469cabf___L27086_2852_Petra_plant_CC_dark_28−24−14, Scan 964 (Precursor m/z: 736.8463, 2+)
COMET Xcorr: 3.33, MS−GF+ −log10(SpecEval): 12.14, Crux Xcorr: 2.85, MS2PIP Pearson: 0.89063202
Intensity
0
200
400
600
M/Z
500
1000
y7
a7_
y8
y9
y12
b5
b6
y10
y11
y3
y2
b3

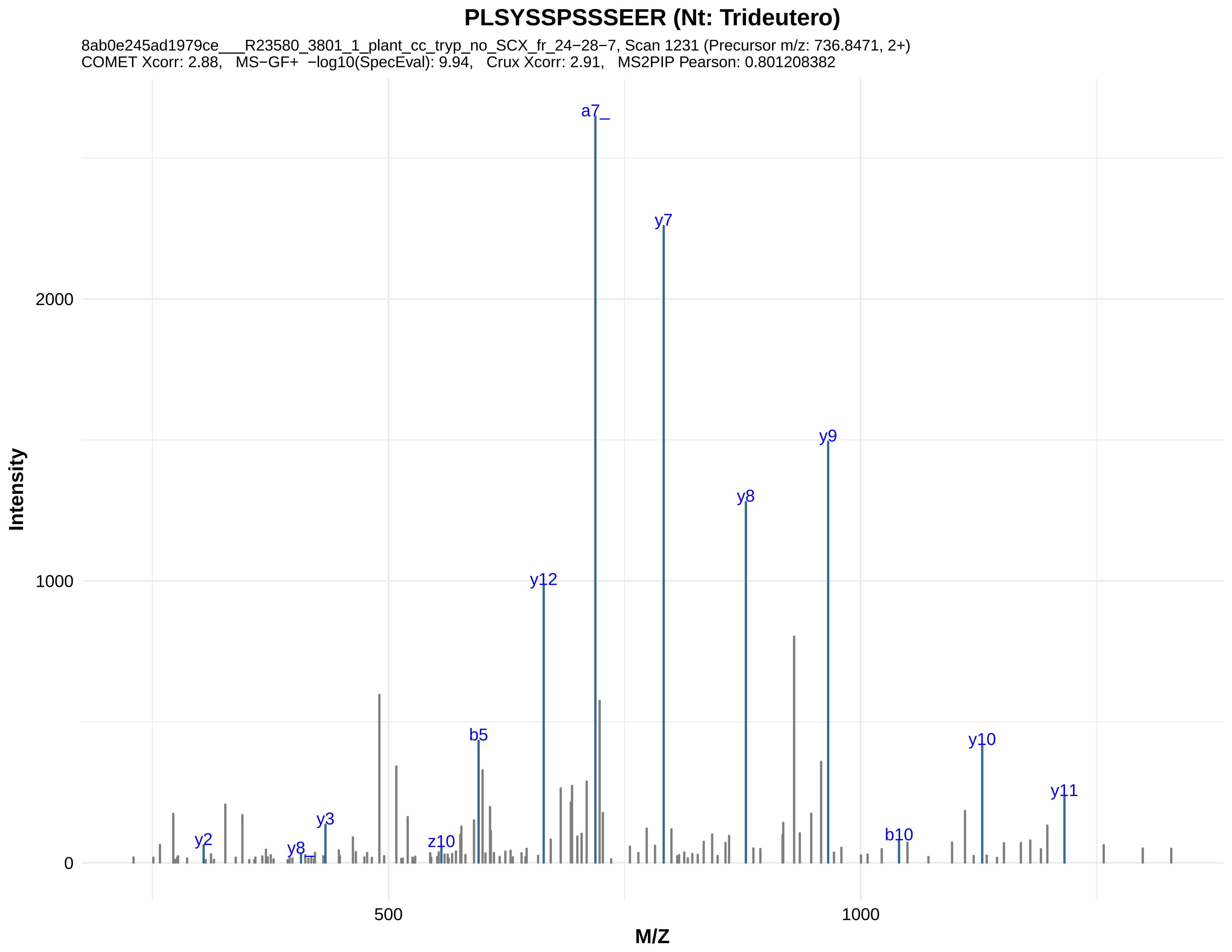
PLSYSSPSSSEER (Nt: Trideutero)
8ab0e245ad1979ce___R23580_3801_1_plant_cc_tryp_no_SCX_fr_24−28−7, Scan 1231 (Precursor m/z: 736.8471, 2+)
COMET Xcorr: 2.88, MS−GF+ −log10(SpecEval): 9.94, Crux Xcorr: 2.91, MS2PIP Pearson: 0.801208382
Intensity
M/Z
0
1000
2000
500
1000
a7_
y7
y9
y8
y12
b5
y10
y11
y3
y2
y8_
z10
b10

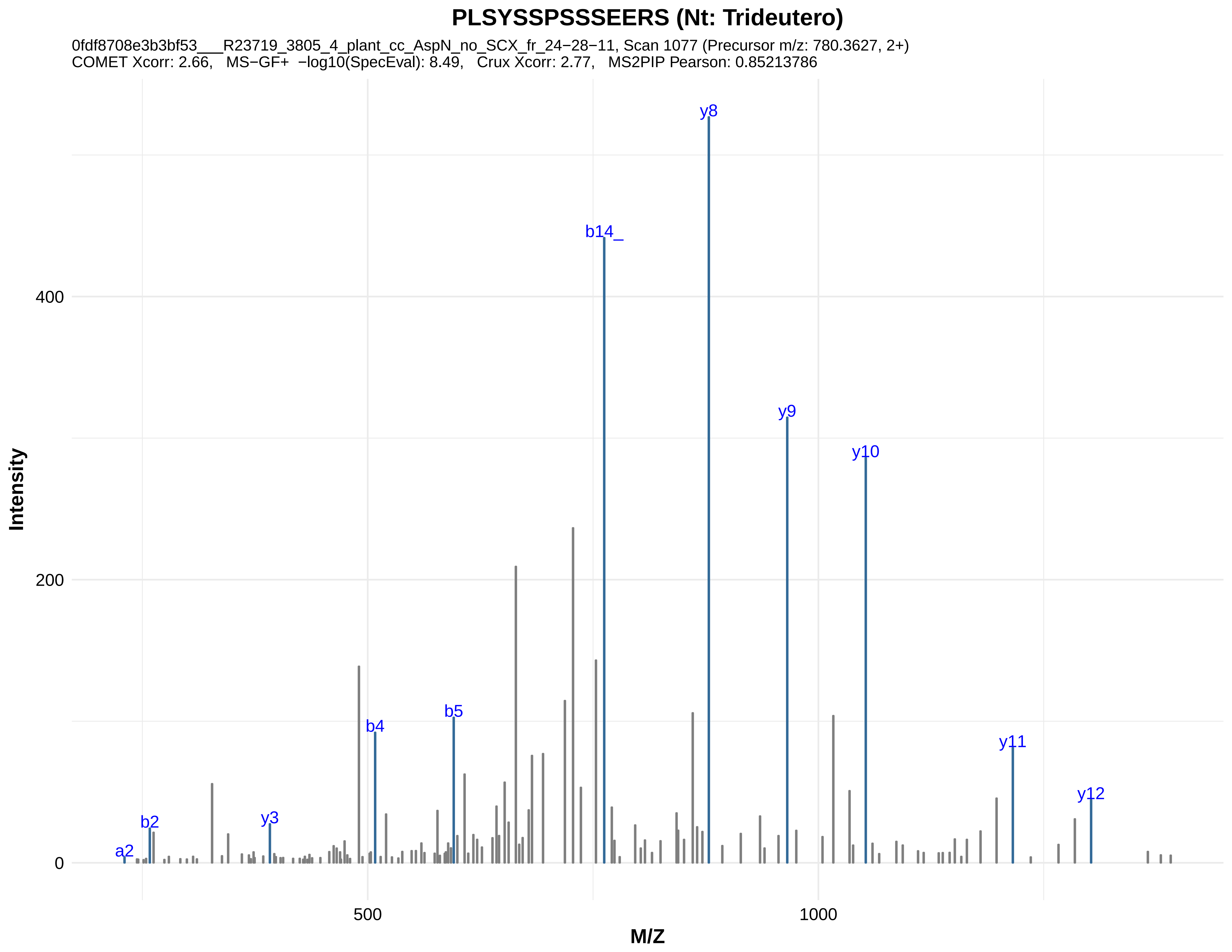
PLSYSSPSSSEERS (Nt: Trideutero)
0fdf8708e3b3bf53___R23719_3805_4_plant_cc_AspN_no_SCX_fr_24−28−11, Scan 1077 (Precursor m/z: 780.3627, 2+)
COMET Xcorr: 2.66, MS−GF+ −log10(SpecEval): 8.49, Crux Xcorr: 2.77, MS2PIP Pearson: 0.85213786
Intensity
M/Z
0
200
400
500
1000
a2
b2
y3
b4
b5
b14_
y8
y9
y10
y11
y12

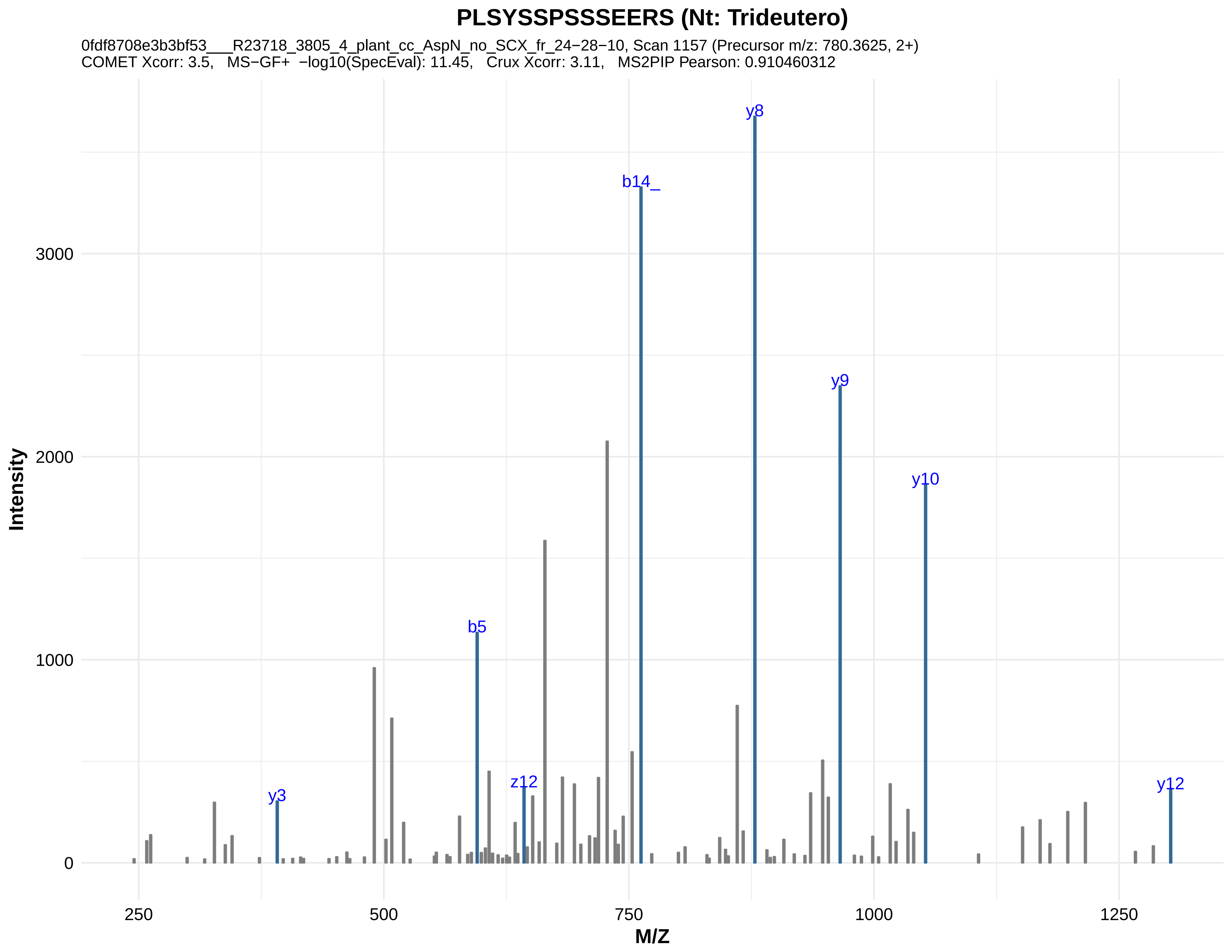

PLSYSSPSSSEERS (Nt: Trideutero)
0fdf8708e3b3bf53___R23718_3805_4_plant_cc_AspN_no_SCX_fr_24−28−10, Scan 1157 (Precursor m/z: 780.3625, 2+)
COMET Xcorr: 3.5, MS−GF+ −log10(SpecEval): 11.45, Crux Xcorr: 3.11, MS2PIP Pearson: 0.910460312
y8
b14_
y9
y10
b5
z12
y3
y12
Intensity
3000
2000
1000
0
250
500
750
1000
1250
M/Z

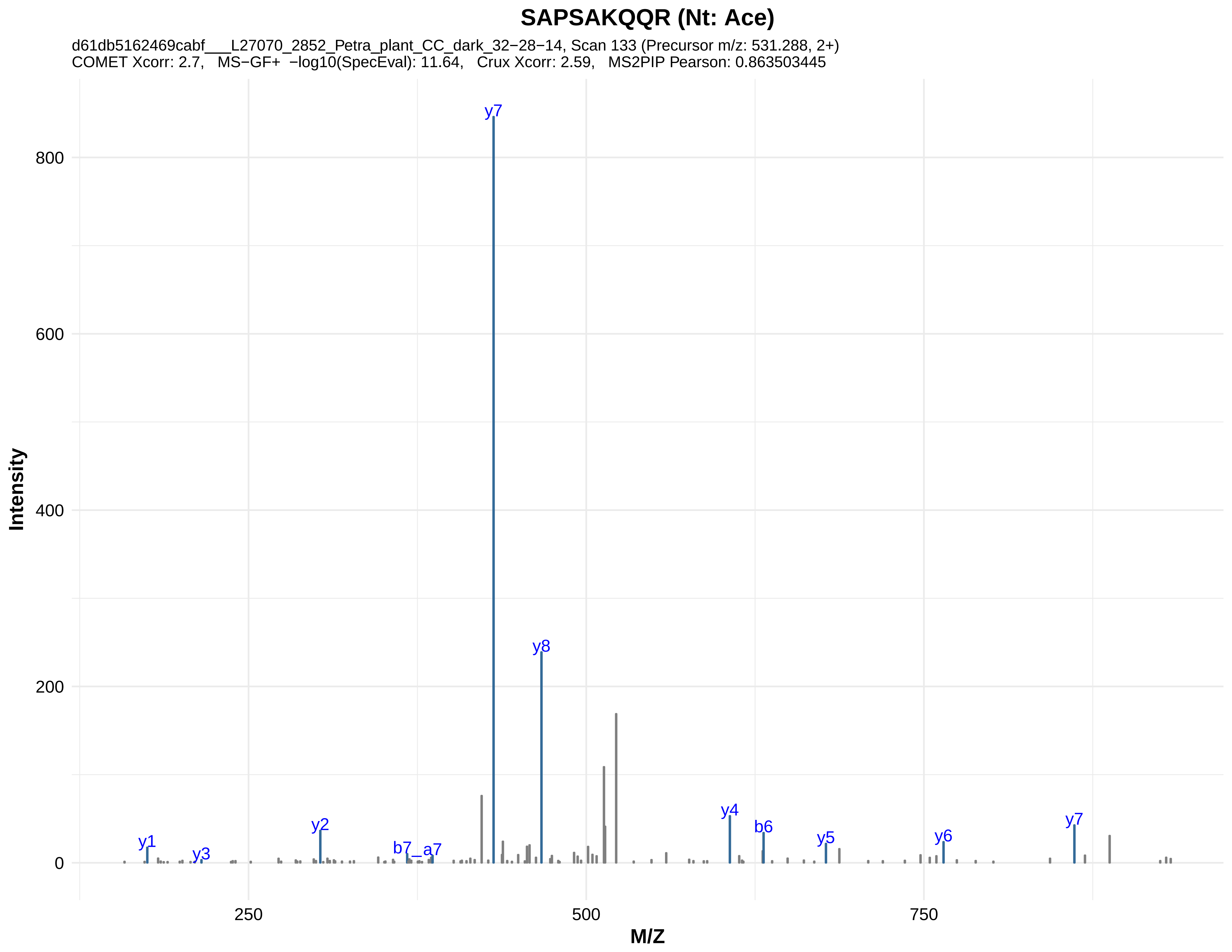
SAPSAKQQR (Nt: Ace)
d61db5162469cabf___L27070_2852_Petra_plant_CC_dark_32−28−14, Scan 133 (Precursor m/z: 531.288, 2+)
COMET Xcorr: 2.7, MS−GF+ −log10(SpecEval): 11.64, Crux Xcorr: 2.59, MS2PIP Pearson: 0.863503445
Intensity
M/Z
0
200
400
600
800
250
500
750
y7
y8
y1
y3
y2
b7_a7
y4
b6
y5
y6
y7

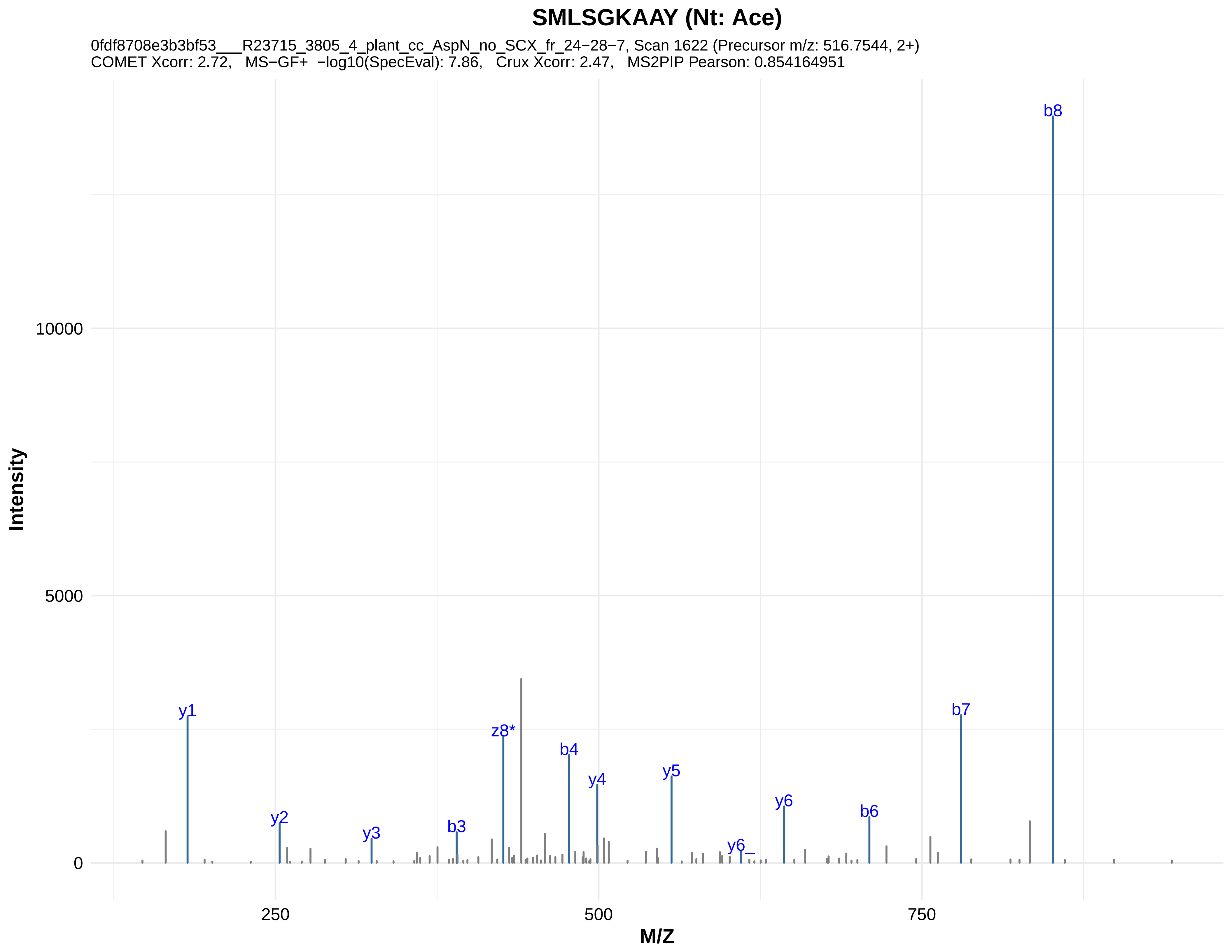
SMLSGKAAY (Nt: Ace)
0fdf8708e3b3bf53___R23715_3805_4_plant_cc_AspN_no_SCX_fr_24−28−7, Scan 1622 (Precursor m/z: 516.7544, 2+)
COMET Xcorr: 2.72, MS−GF+ −log10(SpecEval): 7.86, Crux Xcorr: 2.47, MS2PIP Pearson: 0.854164951
b8
y1
z8*
b4
y4
y5
y2
y3
b3
y6_
y6
b6
b7
Intensity
10000
5000
0
250
500
750
M/Z

SNLSTSSTGSTGPR (Nt: Ace)

8ab0e245ad1979ce___R23591_3801_1_plant_cc_tryp_no_SCX_fr_20−24−3, Scan 508 (Precursor m/z: 697.3303, 2+)
COMET Xcorr: 2.76,   MS−GF+ −log10(SpecEval): 10.49,   Crux Xcorr: 2.58,   MS2PIP Pearson: 0.836319034

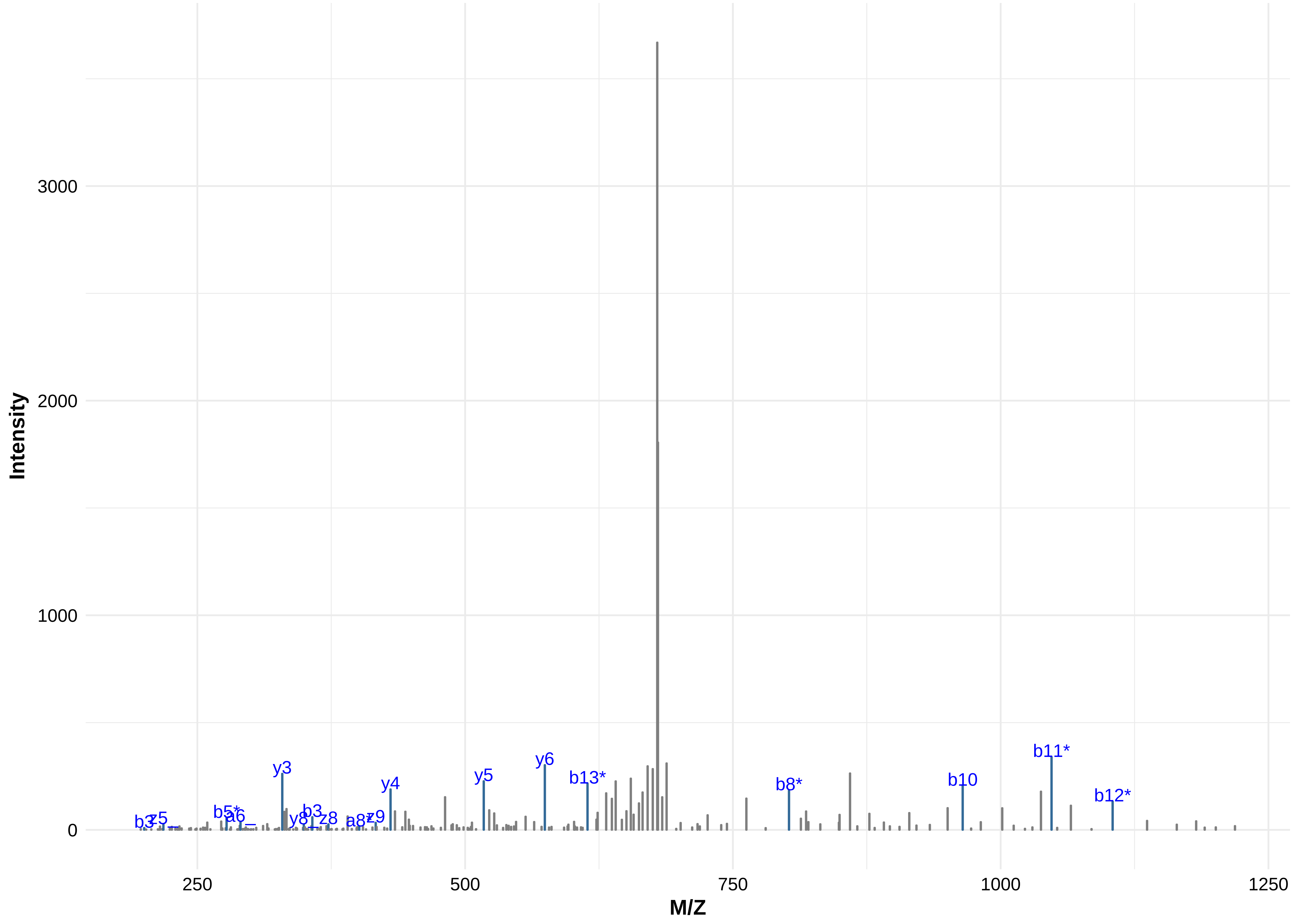

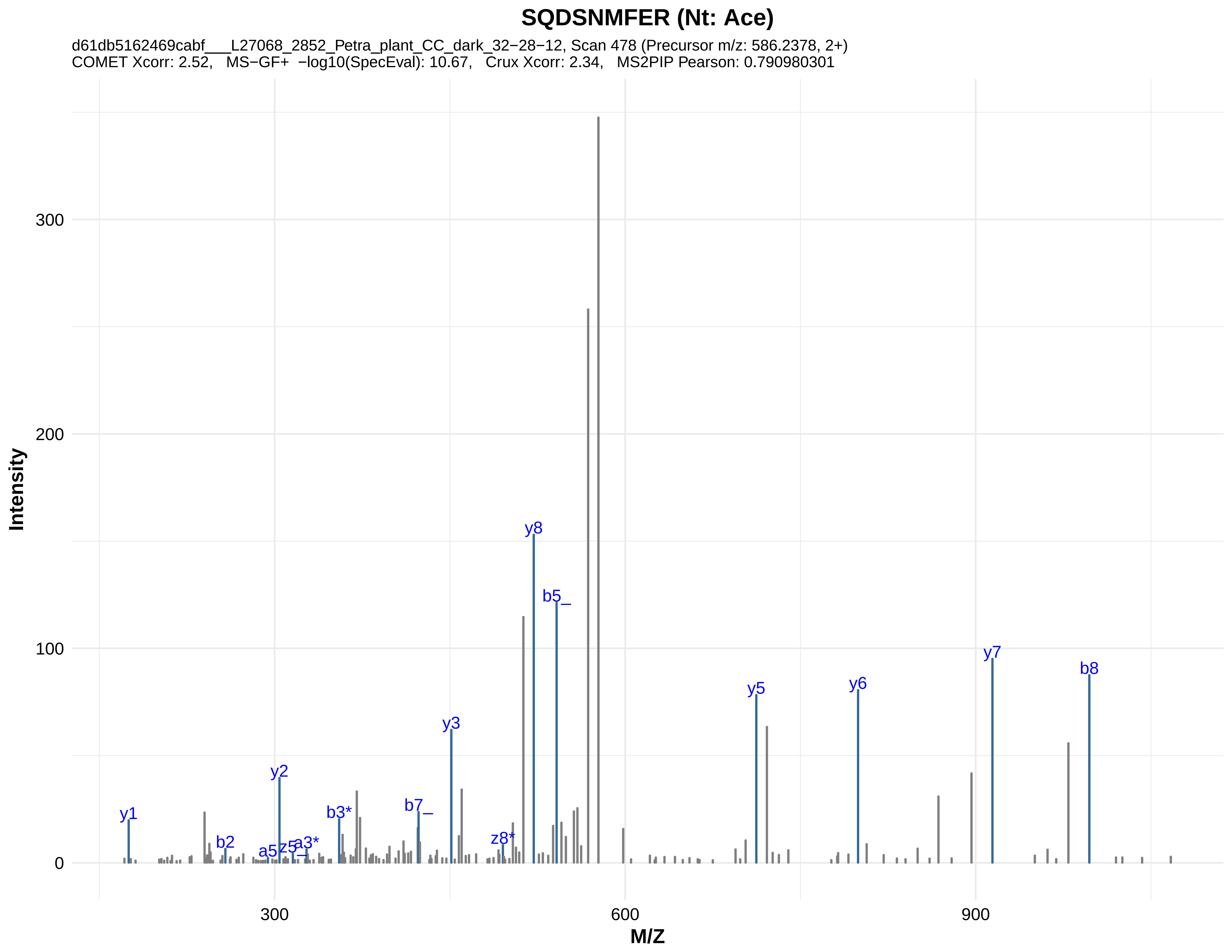
SQDSNMFER (Nt: Ace)
d61db5162469cabf___L27068_2852_Petra_plant_CC_dark_32−28−12, Scan 478 (Precursor m/z: 586.2378, 2+)
COMET Xcorr: 2.52, MS−GF+ −log10(SpecEval): 10.67, Crux Xcorr: 2.34, MS2PIP Pearson: 0.790980301
Intensity
M/Z
0
100
200
300
300
600
900
y1
b2
a5
z5_
a3*
y2
b3*
b7_
y3
z8*
y8
b5_
y5
y6
y7
b8

SQQPPAVGVPPSHAYPAEGPPK (Nt: Ace)

0fdf8708e3b3bf53___R23700_3805_4_plant_cc_AspN_no_SCX_fr_28−32−7, Scan 1527 (Precursor m/z: 767.3935, 3+)
COMET Xcorr: 2.6, MS−GF+ −log10(SpecEval): 12.24, Crux Xcorr: 2.77, MS2PIP Pearson: 0.703544495

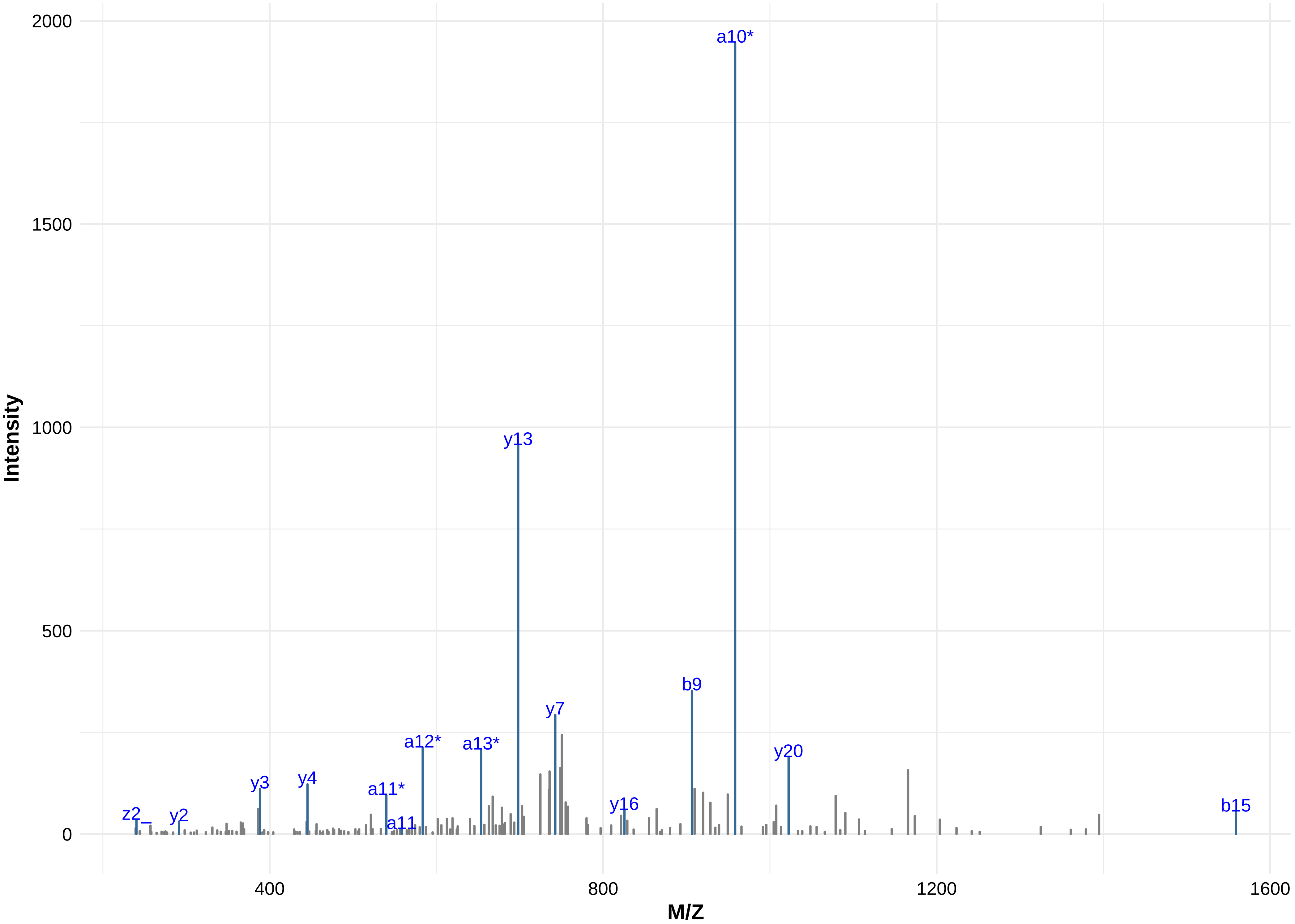

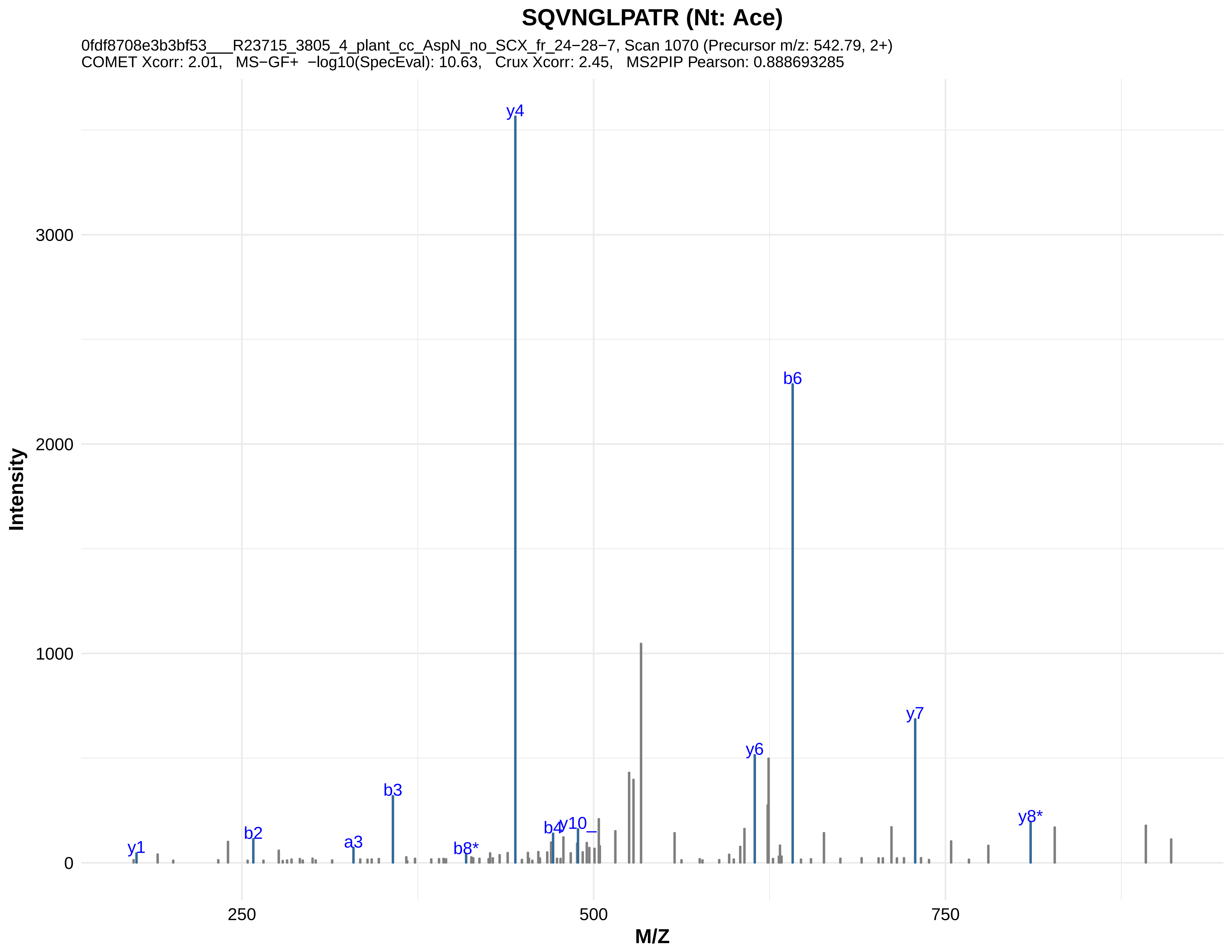

SQVNGLPATR (Nt: Ace)
0fdf8708e3b3bf53___R23715_3805_4_plant_cc_AspN_no_SCX_fr_24−28−7, Scan 1070 (Precursor m/z: 542.79, 2+)
COMET Xcorr: 2.01, MS−GF+ −log10(SpecEval): 10.63, Crux Xcorr: 2.45, MS2PIP Pearson: 0.888693285
Intensity
M/Z
0
1000
2000
3000
250
500
750
y4
b6
y7
y6
b3
b2
a3
y1
b8*
b4
y10_
y8*

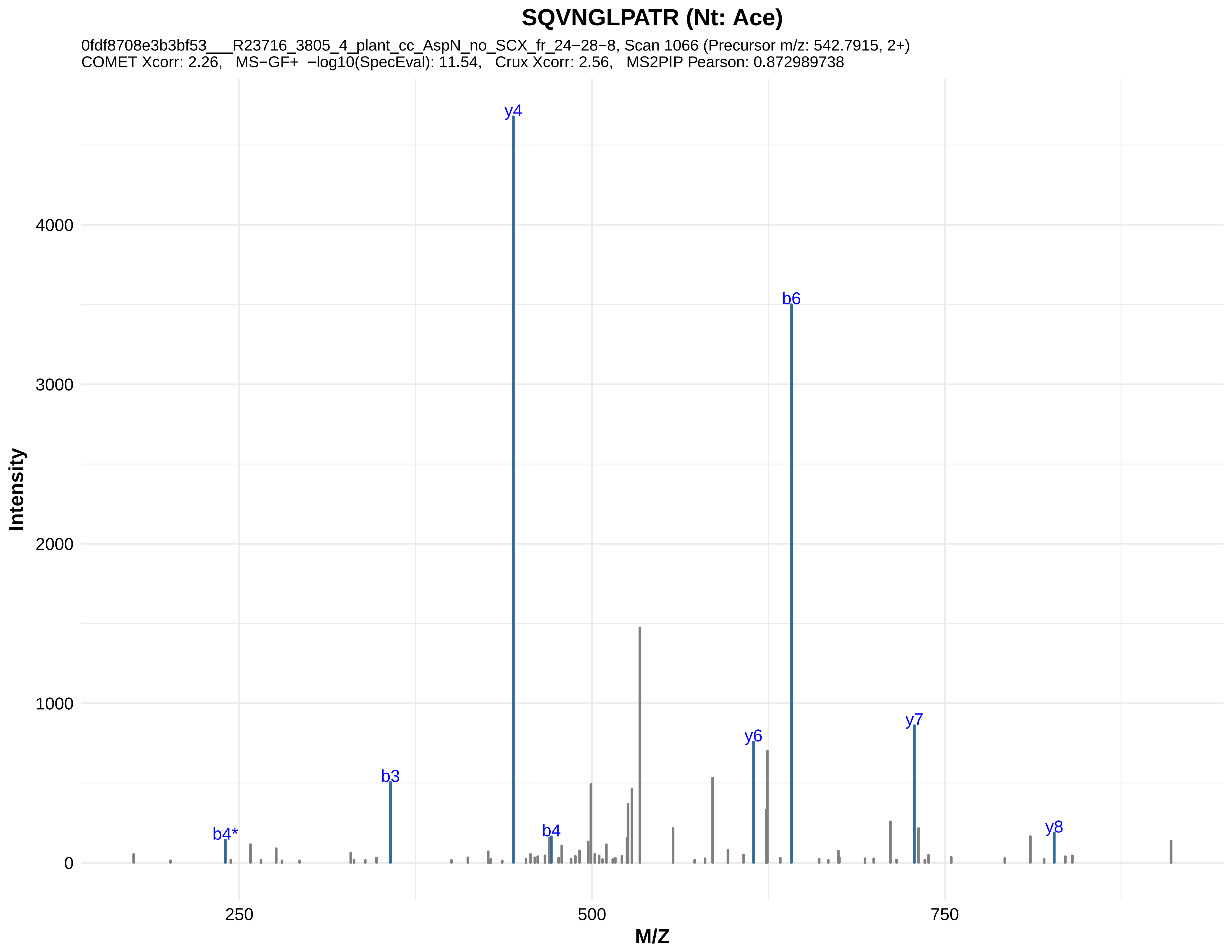
SQVNGLPATR (Nt: Ace)
0fdf8708e3b3bf53___R23716_3805_4_plant_cc_AspN_no_SCX_fr_24−28−8, Scan 1066 (Precursor m/z: 542.7915, 2+)
COMET Xcorr: 2.26, MS−GF+ −log10(SpecEval): 11.54, Crux Xcorr: 2.56, MS2PIP Pearson: 0.872989738
Intensity
M/Z
y4
b6
b3
b4*
b4
y6
y7
y8
0
1000
2000
3000
4000
250
500
750

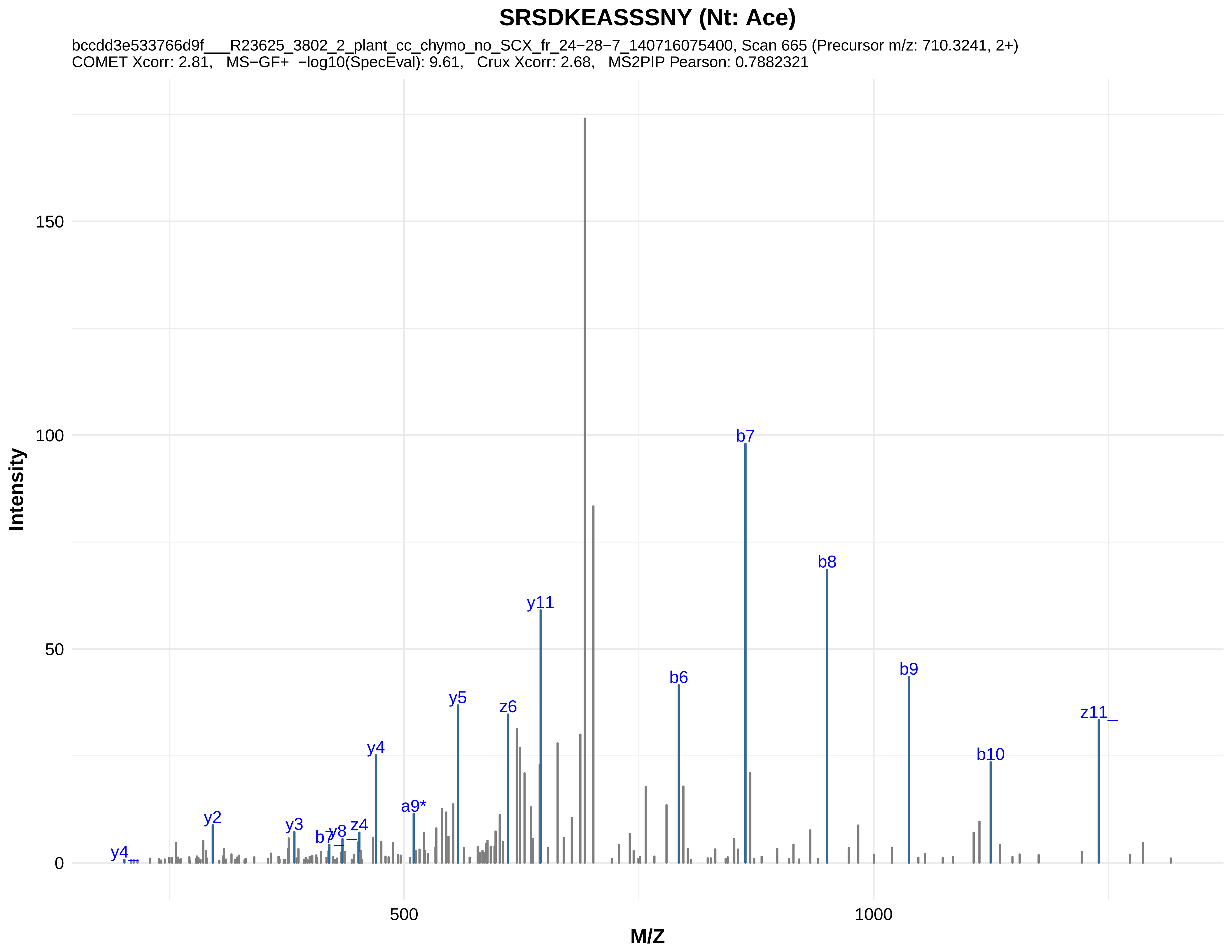
SRSDKEASSSNY (Nt: Ace)
bccdd3e533766d9f___R23625_3802_2_plant_cc_chymo_no_SCX_fr_24−28−7_140716075400, Scan 665 (Precursor m/z: 710.3241, 2+)
COMET Xcorr: 2.81, MS−GF+ −log10(SpecEval): 9.61, Crux Xcorr: 2.68, MS2PIP Pearson: 0.7882321
Intensity
M/Z
0
50
100
150
500
1000
y4_
y2
y3
b7_
y8_
z4
y4
a9*
y5
z6
y11
b6
b7
b8
b9
b10
z11_

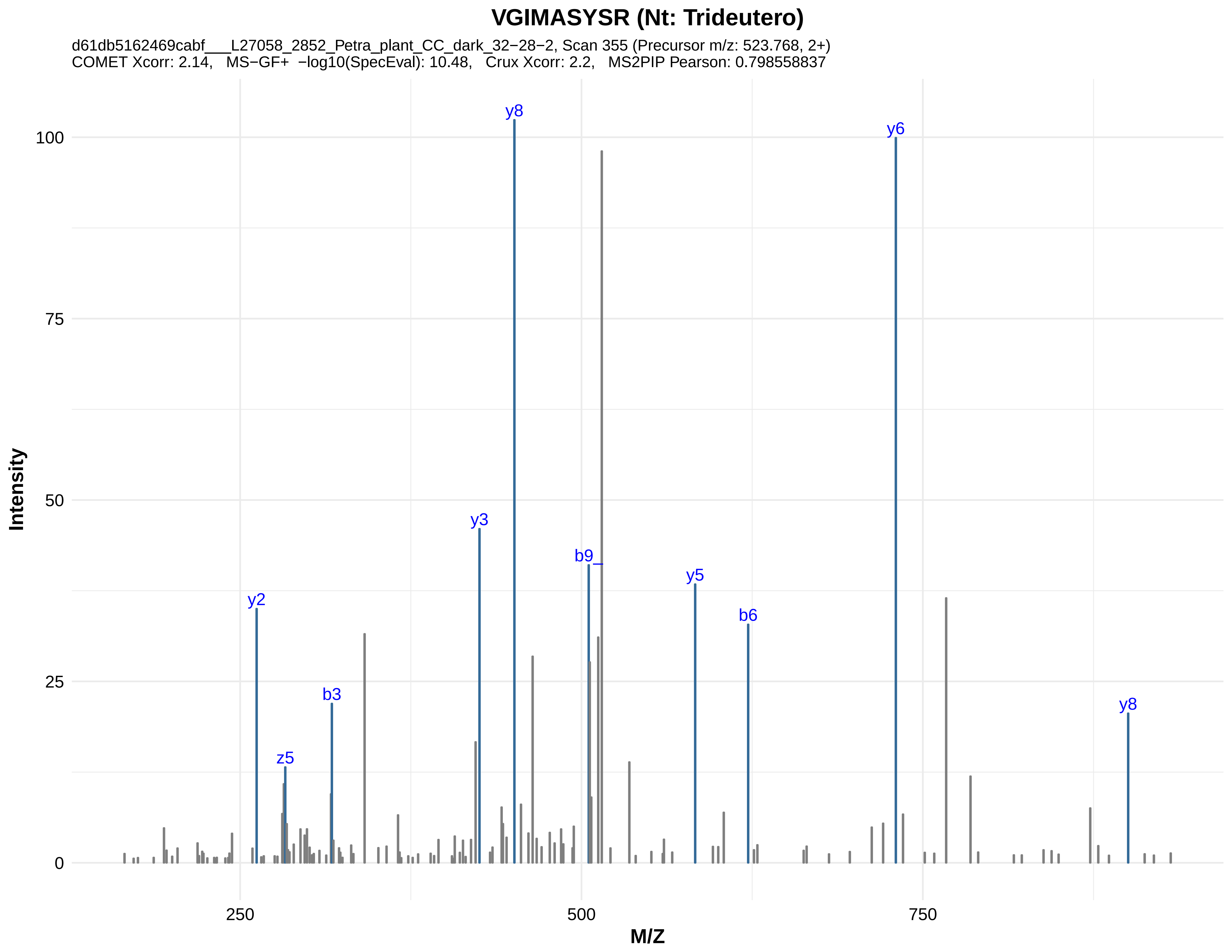
VGIMASYSR (Nt: Trideutero)
d61db5162469cabf___L27058_2852_Petra_plant_CC_dark_32−28−2, Scan 355 (Precursor m/z: 523.768, 2+)
COMET Xcorr: 2.14, MS−GF+ −log10(SpecEval): 10.48, Crux Xcorr: 2.2, MS2PIP Pearson: 0.798558837
Intensity
M/Z
y8
y6
y3
b9_
y5
y2
b6
b3
y8
z5
0
25
50
75
100
250
500
750

Medium-confidence PSMs

TIS meta-data available (see Supplemental Dataset 1)

OR good MS2PIP correlation (see Supplemental Dataset 3)

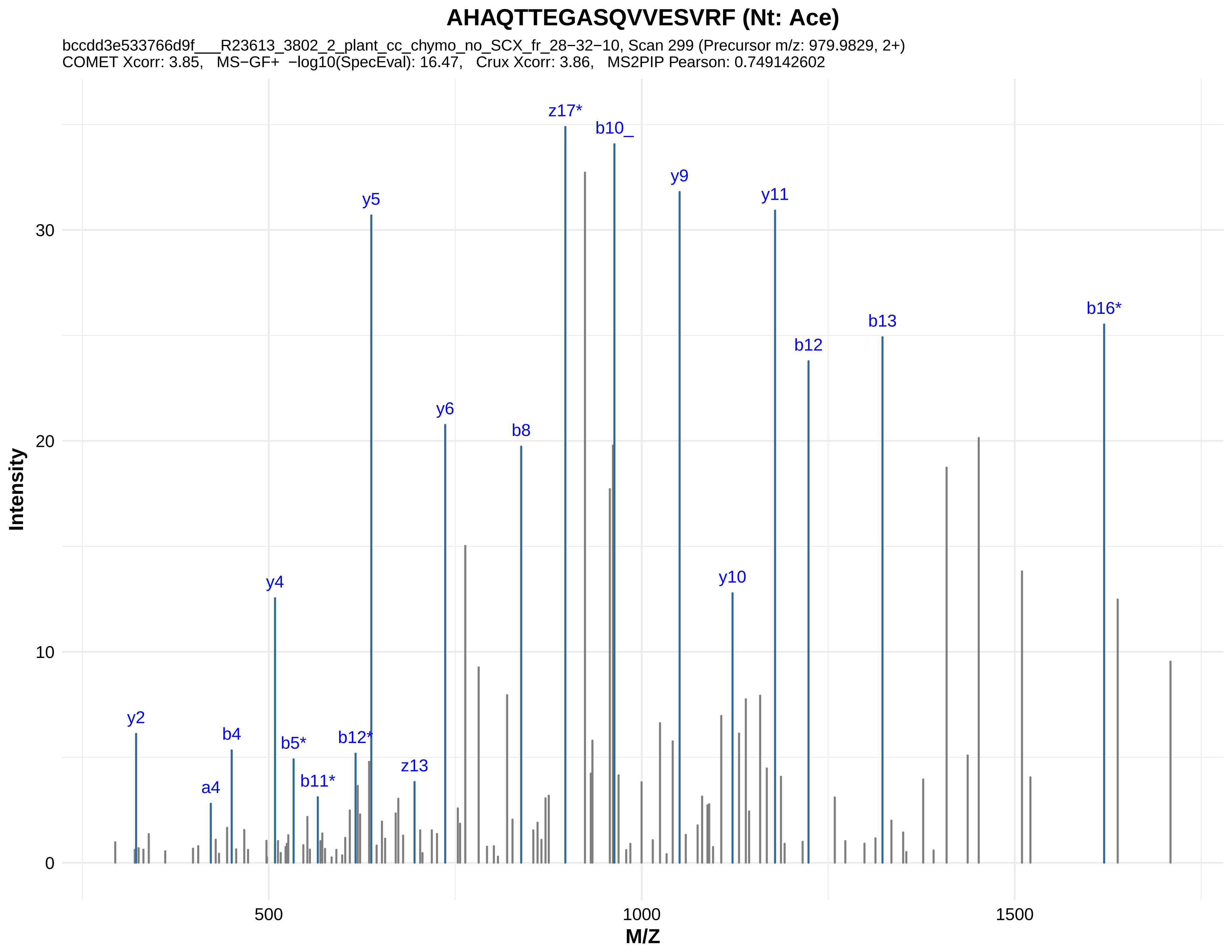
AHAQTTEGASQVVESVRF (Nt: Ace)
bccdd3e533766d9f___R23613_3802_2_plant_cc_chymo_no_SCX_fr_28−32−10, Scan 299 (Precursor m/z: 979.9829, 2+)
COMET Xcorr: 3.85, MS−GF+ −log10(SpecEval): 16.47, Crux Xcorr: 3.86, MS2PIP Pearson: 0.749142602
Intensity
M/Z
z17*
b10_
y9
y11
y5
b16*
b13
b12
y6
b8
y4
y10
y2
b4
b5*
b12*
z13
a4
b11*
0
10
20
30
500
1000
1500

ATGIWIYGVSWFR (Nt: Ace)

8ab0e245ad1979ce___R23576_3801_1_plant_cc_tryp_no_SCX_fr_24−28−3, Scan 600 (Precursor m/z: 534.956, 3+)
COMET Xcorr: 2.24,   MS−GF+ −log10(SpecEval): 5.47,   Crux Xcorr: NA,   MS2PIP Pearson: 0.479905575

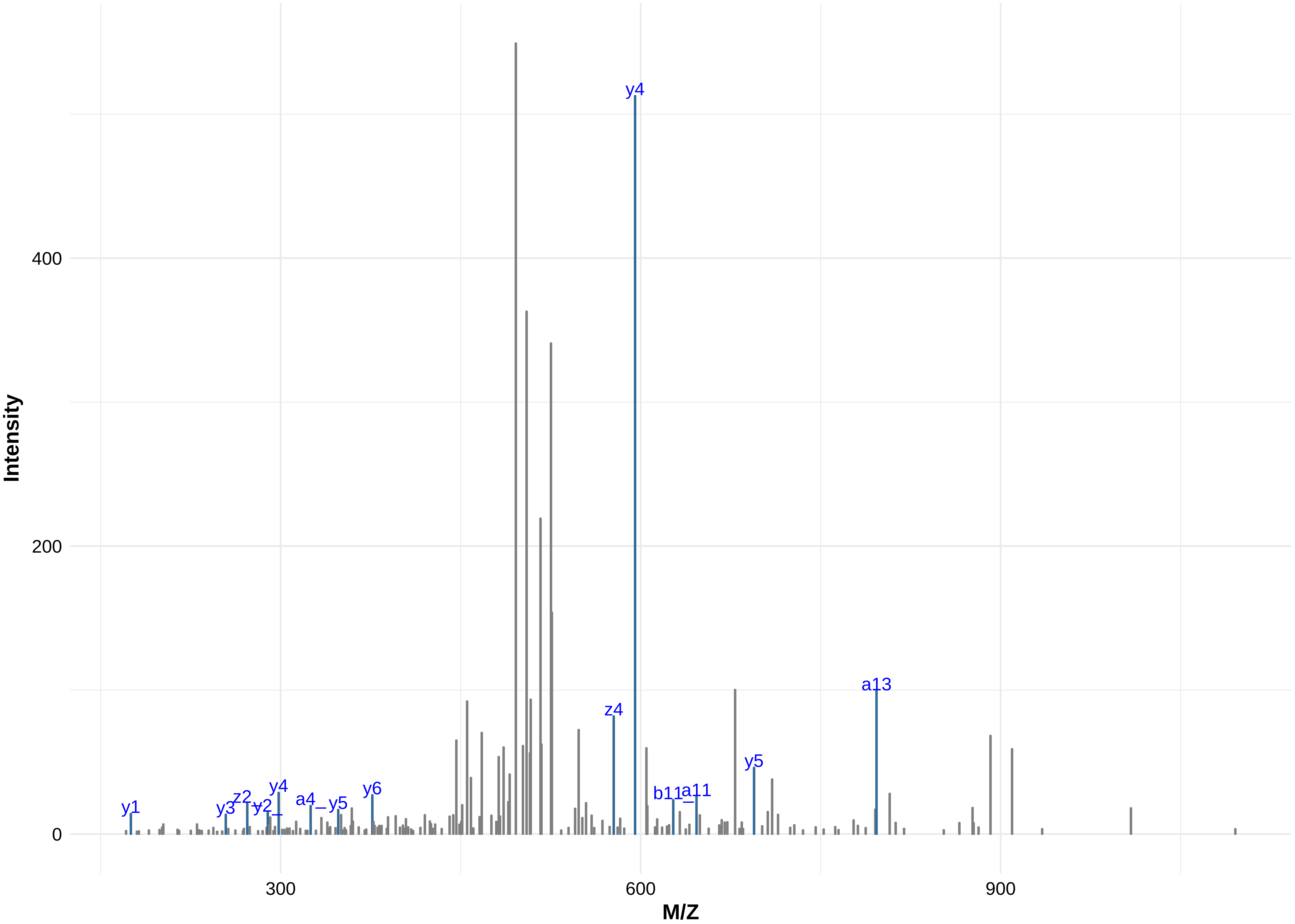

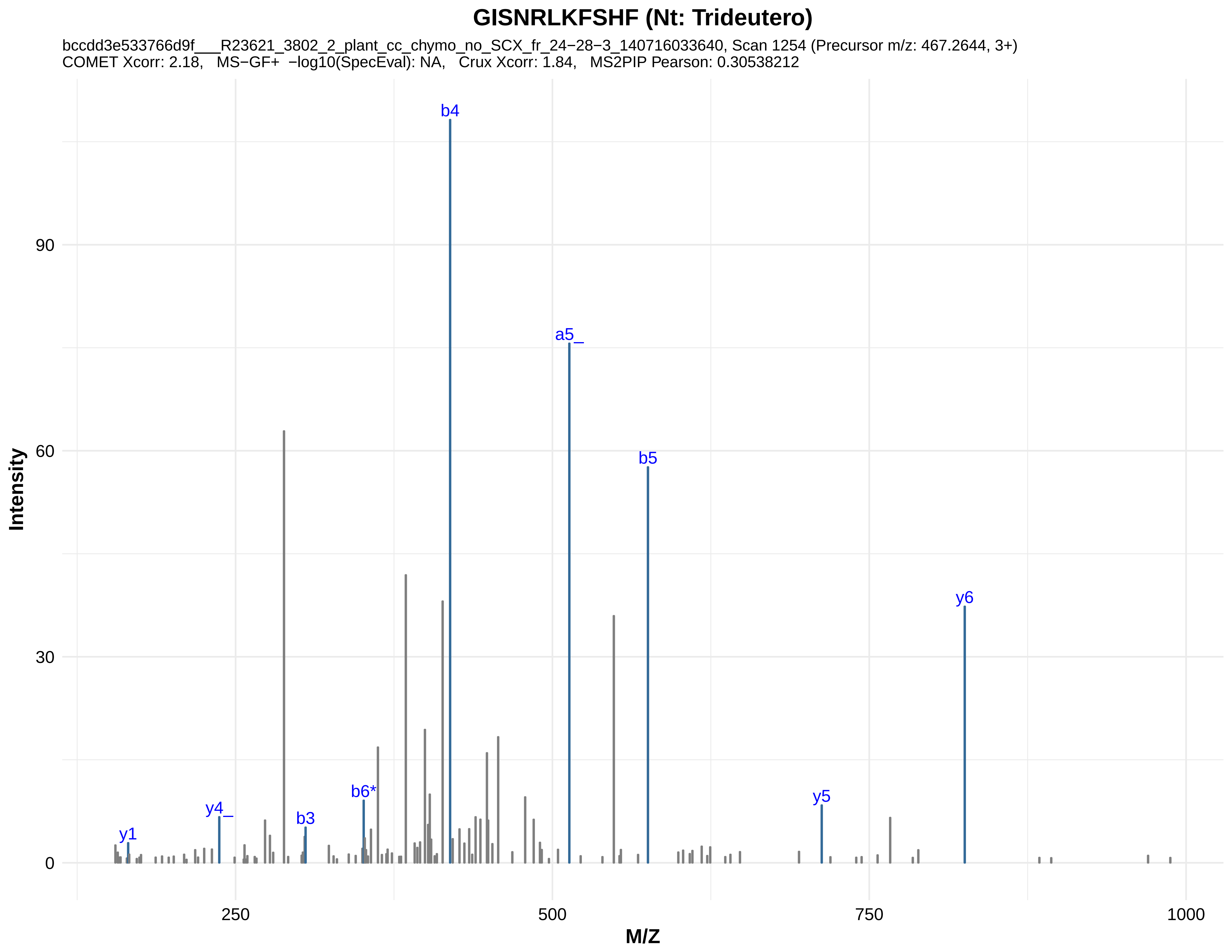
GISNRLKFSHF (Nt: Trideutero)
bccdd3e533766d9f___R23621_3802_2_plant_cc_chymo_no_SCX_fr_24−28−3_140716033640, Scan 1254 (Precursor m/z: 467.2644, 3+)
COMET Xcorr: 2.18, MS−GF+ −log10(SpecEval): NA, Crux Xcorr: 1.84, MS2PIP Pearson: 0.30538212
Intensity
M/Z
0
30
60
90
250
500
750
1000
b4
a5_
b5
y6
y5
b6*
b3
y4_
y1

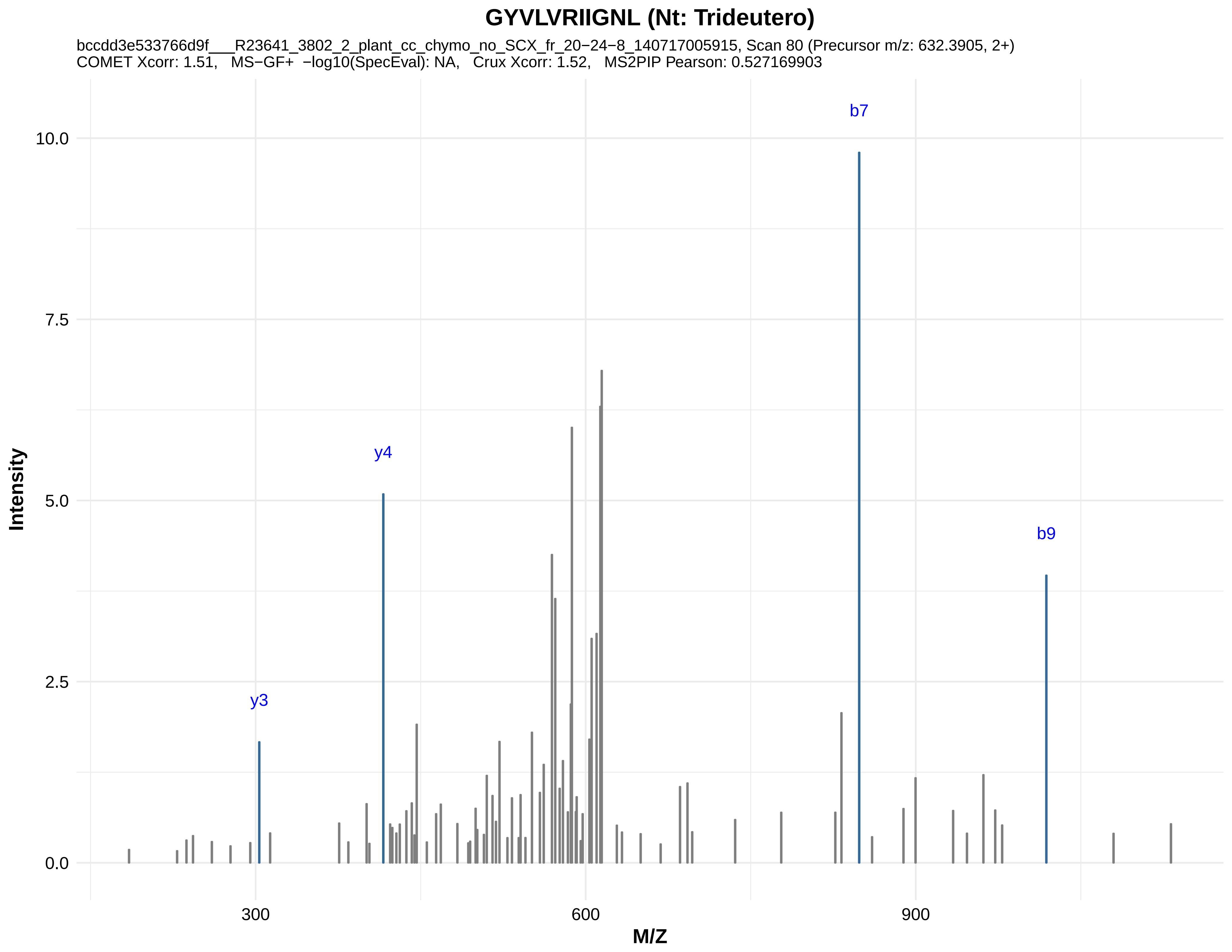

GYVLVRIIGNL (Nt: Trideutero)
bccdd3e533766d9f___R23641_3802_2_plant_cc_chymo_no_SCX_fr_20−24−8_140717005915, Scan 80 (Precursor m/z: 632.3905, 2+)
COMET Xcorr: 1.51, MS−GF+ −log10(SpecEval): NA, Crux Xcorr: 1.52, MS2PIP Pearson: 0.527169903
Intensity
M/Z
y3
y4
b7
b9
0.0
2.5
5.0
7.5
10.0
300
600
900

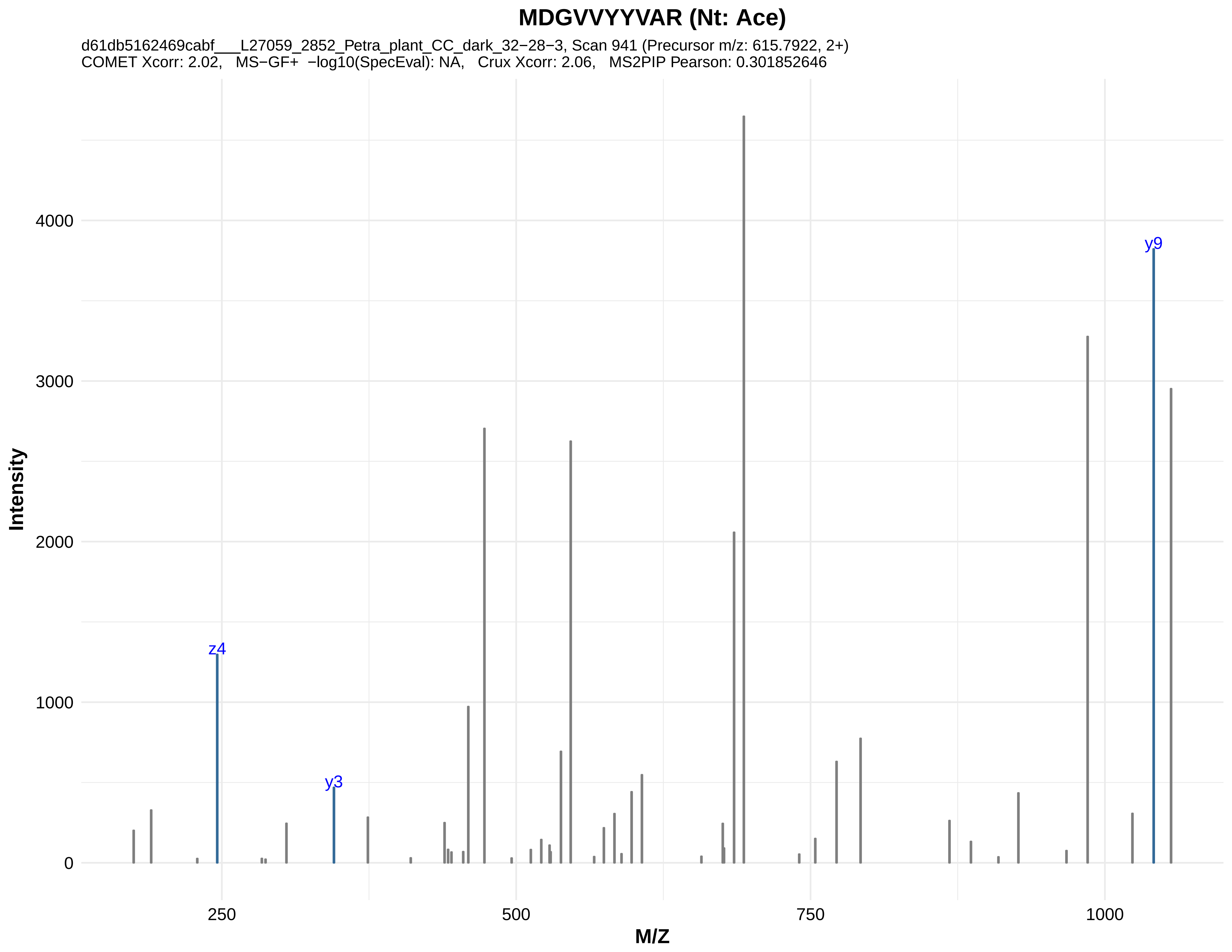
MDGVVYYVAR (Nt: Ace)
d61db5162469cabf___L27059_2852_Petra_plant_CC_dark_32−28−3, Scan 941 (Precursor m/z: 615.7922, 2+)
COMET Xcorr: 2.02, MS−GF+ −log10(SpecEval): NA, Crux Xcorr: 2.06, MS2PIP Pearson: 0.301852646
Intensity
M/Z
0
1000
2000
3000
4000
250
500
750
1000
z4
y3
y9

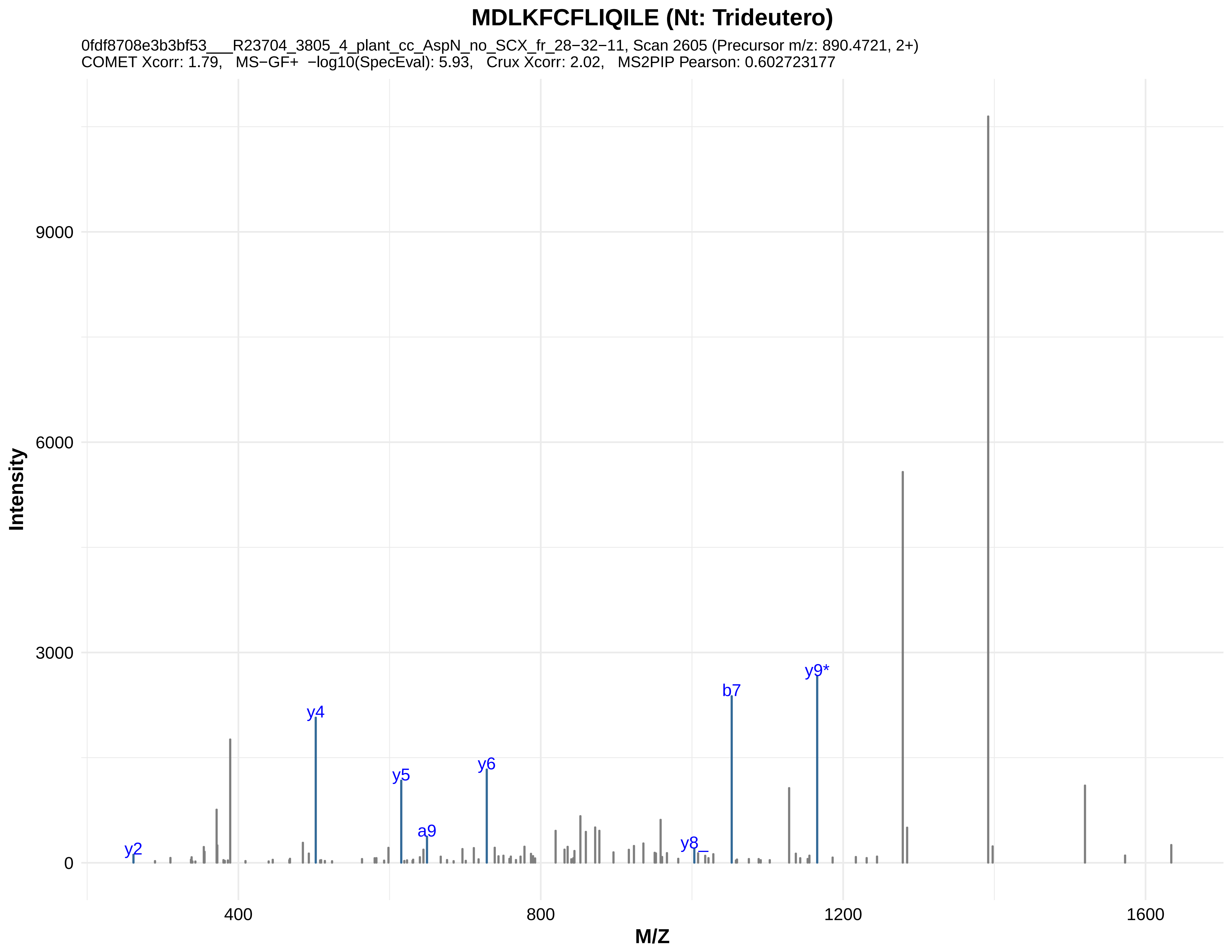

MDLKFCFLIQILE (Nt: Trideutero)
0fdf8708e3b3bf53___R23704_3805_4_plant_cc_AspN_no_SCX_fr_28−32−11, Scan 2605 (Precursor m/z: 890.4721, 2+)
COMET Xcorr: 1.79, MS−GF+ −log10(SpecEval): 5.93, Crux Xcorr: 2.02, MS2PIP Pearson: 0.602723177
Intensity
M/Z
y2
y4
y5
a9
y6
y8_
b7
y9*

MDMLKKESKKR (Nt: Ace)

d61db5162469cabf___L27073_2852_Petra_plant_CC_dark_28−24−1, Scan 311 (Precursor m/z: 828.459, 2+)
COMET Xcorr: 2.3,   MS−GF+ −log10(SpecEval): 9.29,   Crux Xcorr: 2.17,   MS2PIP Pearson: 0.711637742

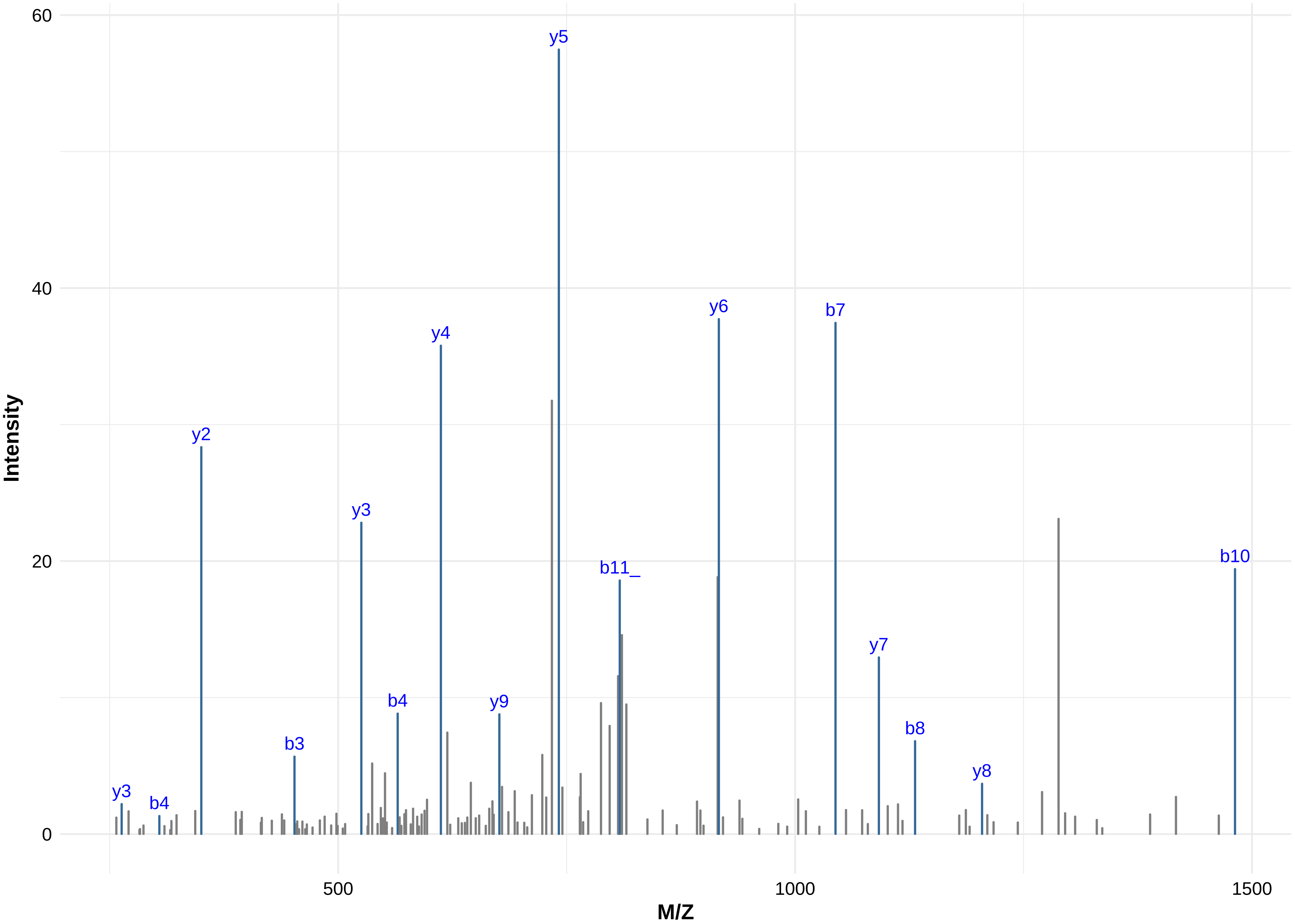

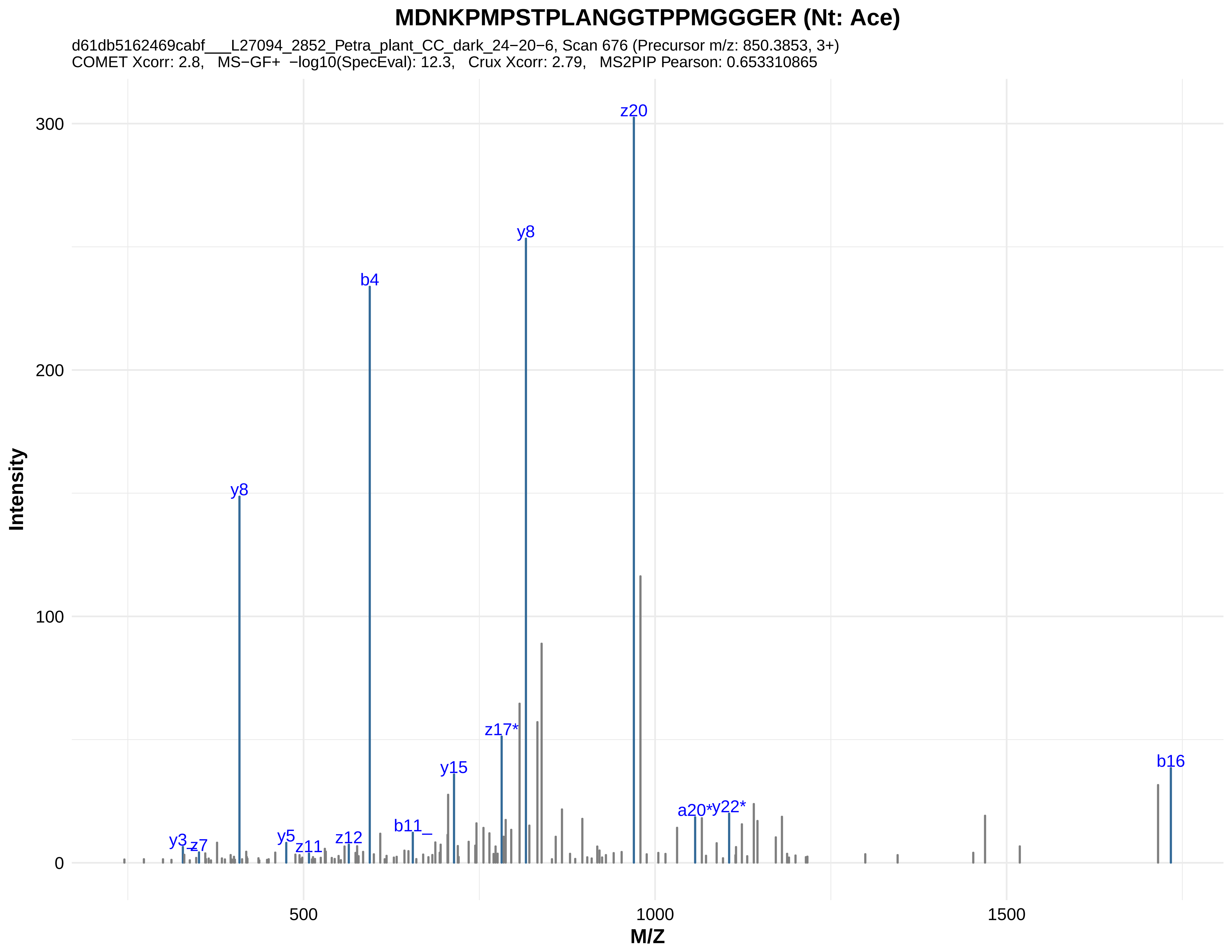

MDNKPMPSTPLANGGTPPMGGGER (Nt: Ace)
d61db5162469cabf___L27094_2852_Petra_plant_CC_dark_24−20−6, Scan 676 (Precursor m/z: 850.3853, 3+)
COMET Xcorr: 2.8, MS−GF+ −log10(SpecEval): 12.3, Crux Xcorr: 2.79, MS2PIP Pearson: 0.653310865
Intensity
M/Z
z20
y8
b4
y8
z17*
y15
b16
a20*
y22*
b11_
y3
z7
y5
z11
z12

MDSSANPNNTESNTKGNEDEGR (Nt: Ace)

d61db5162469cabf___L27065_2852_Petra_plant_CC_dark_32−28−9, Scan 236 (Precursor m/z: 824.677, 3+)
COMET Xcorr: 3.11,   MS−GF+ −log10(SpecEval): 17.14,   Crux Xcorr: 3.32,   MS2PIP Pearson: 0.656955408

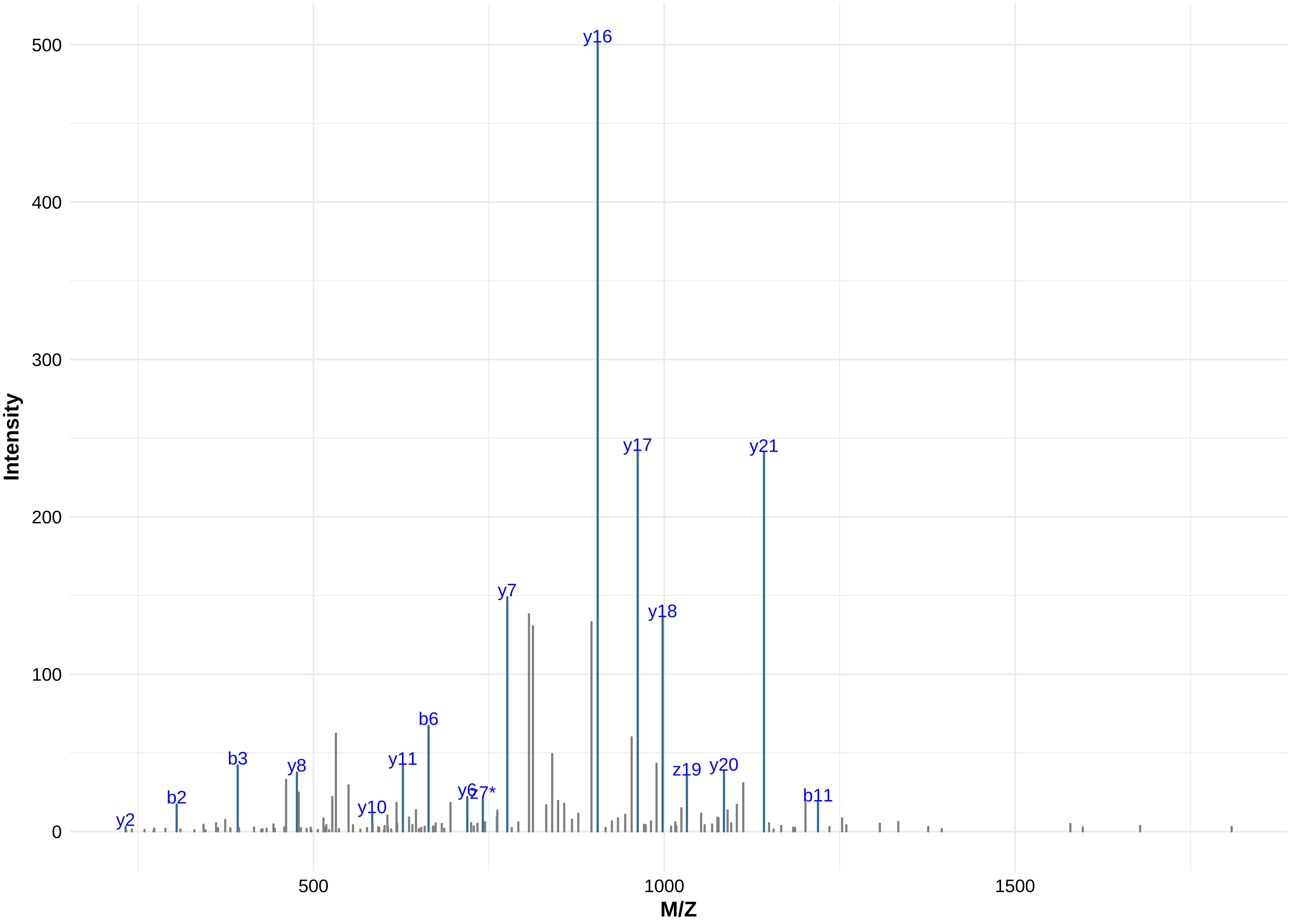

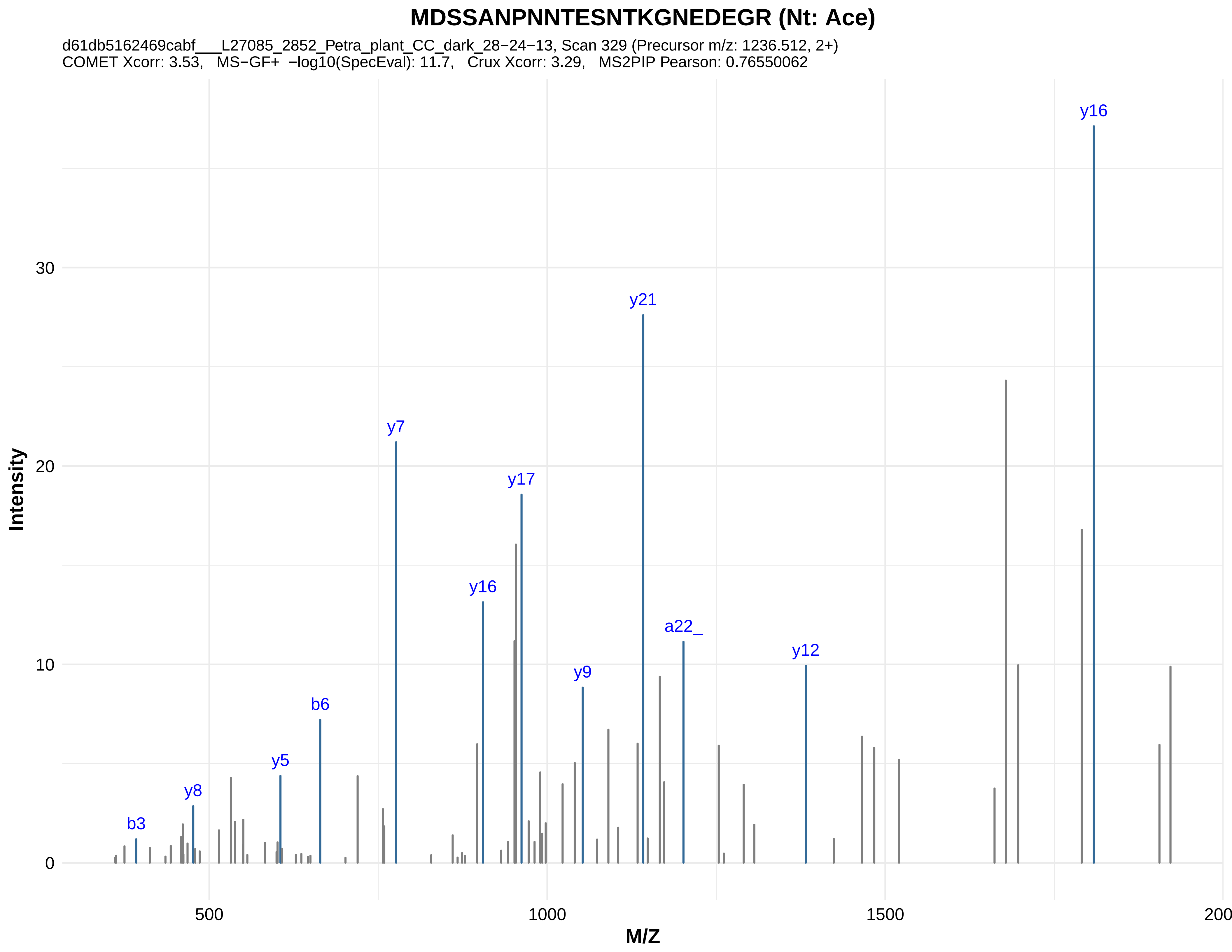
MDSSANPNNTESNTKGNEDEGR (Nt: Ace)
d61db5162469cabf___L27085_2852_Petra_plant_CC_dark_28−24−13, Scan 329 (Precursor m/z: 1236.512, 2+)
COMET Xcorr: 3.53, MS−GF+ −log10(SpecEval): 11.7, Crux Xcorr: 3.29, MS2PIP Pearson: 0.76550062
Intensity
M/Z
0
10
20
30
500
1000
1500
200
y16
y21
y7
y17
y16
a22_
y12
y9
b6
y5
y8
b3

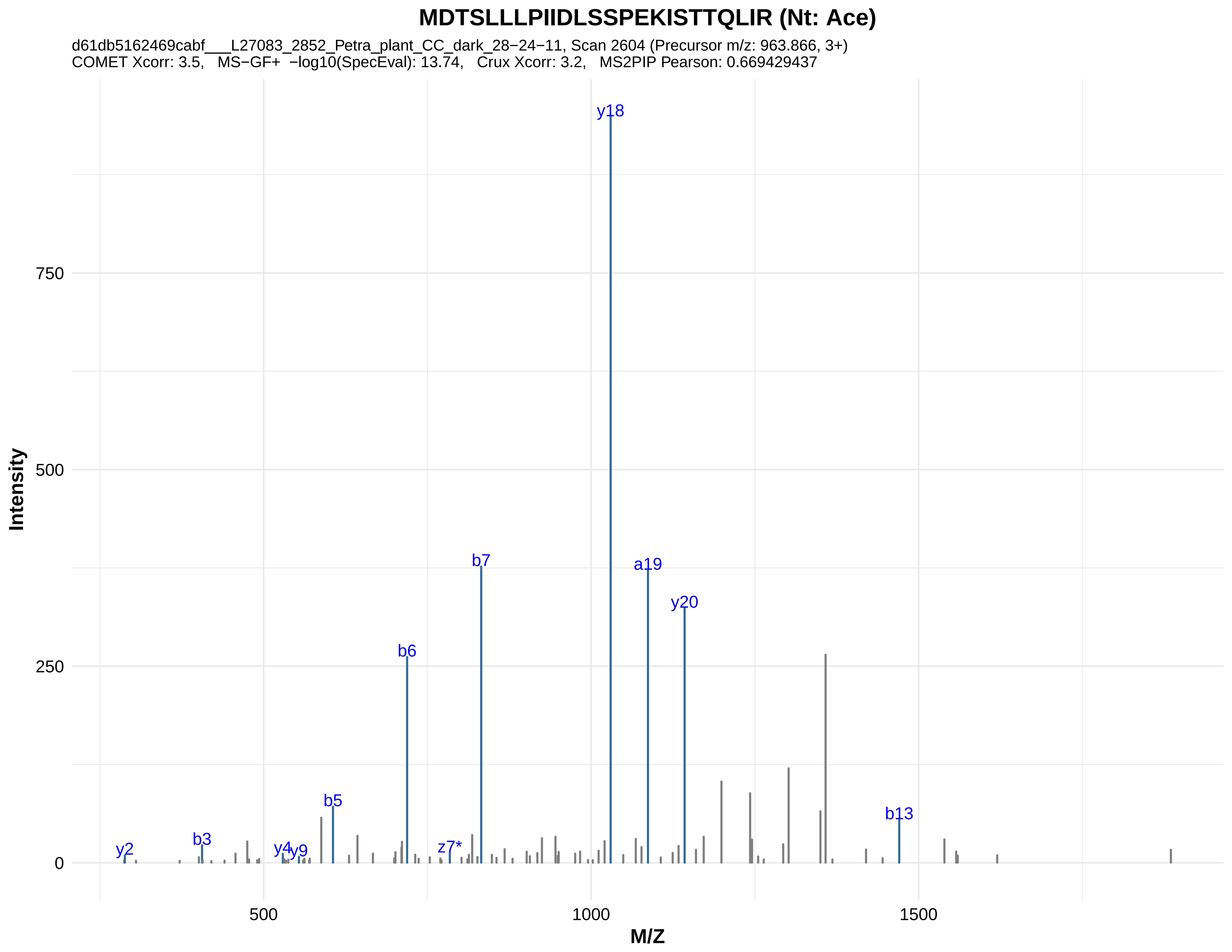

MDTSLLLPIIDLSSPEKISTTQLIR (Nt: Ace)
d61db5162469cabf___L27083_2852_Petra_plant_CC_dark_28−24−11, Scan 2604 (Precursor m/z: 963.866, 3+)
COMET Xcorr: 3.5, MS−GF+ −log10(SpecEval): 13.74, Crux Xcorr: 3.2, MS2PIP Pearson: 0.669429437
Intensity
M/Z
y18
b7
a19
y20
b6
b5
b13
b3
y2
y4
y9
z7*
0
250
500
750
500
1000
1500

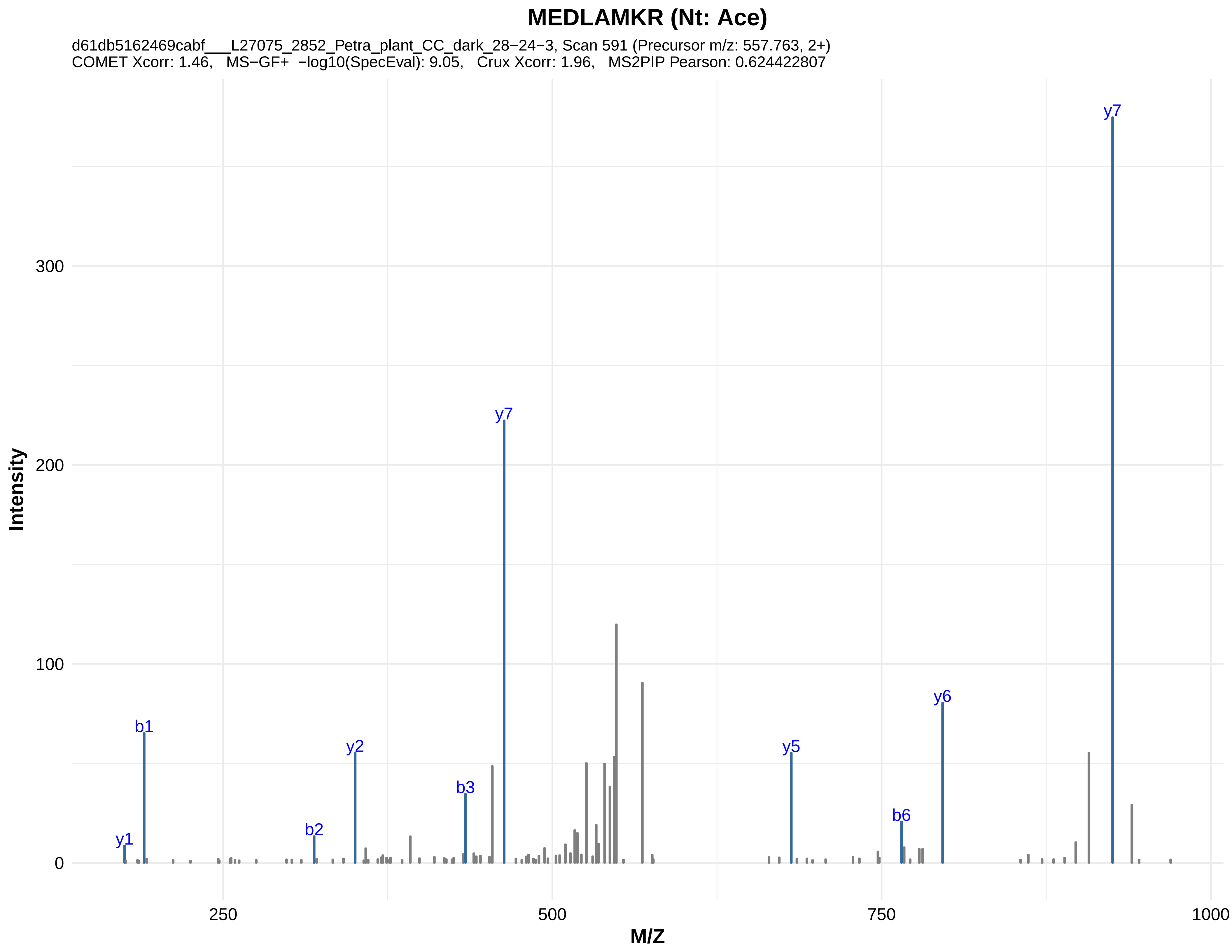

MEDLAMKR (Nt: Ace)
d61db5162469cabf___L27075_2852_Petra_plant_CC_dark_28−24−3, Scan 591 (Precursor m/z: 557.763, 2+)
COMET Xcorr: 1.46, MS−GF+ −log10(SpecEval): 9.05, Crux Xcorr: 1.96, MS2PIP Pearson: 0.624422807
Intensity
M/Z
0
100
200
300
250
500
750
1000
y1
b1
b2
y2
b3
y7
y5
b6
y6
y7

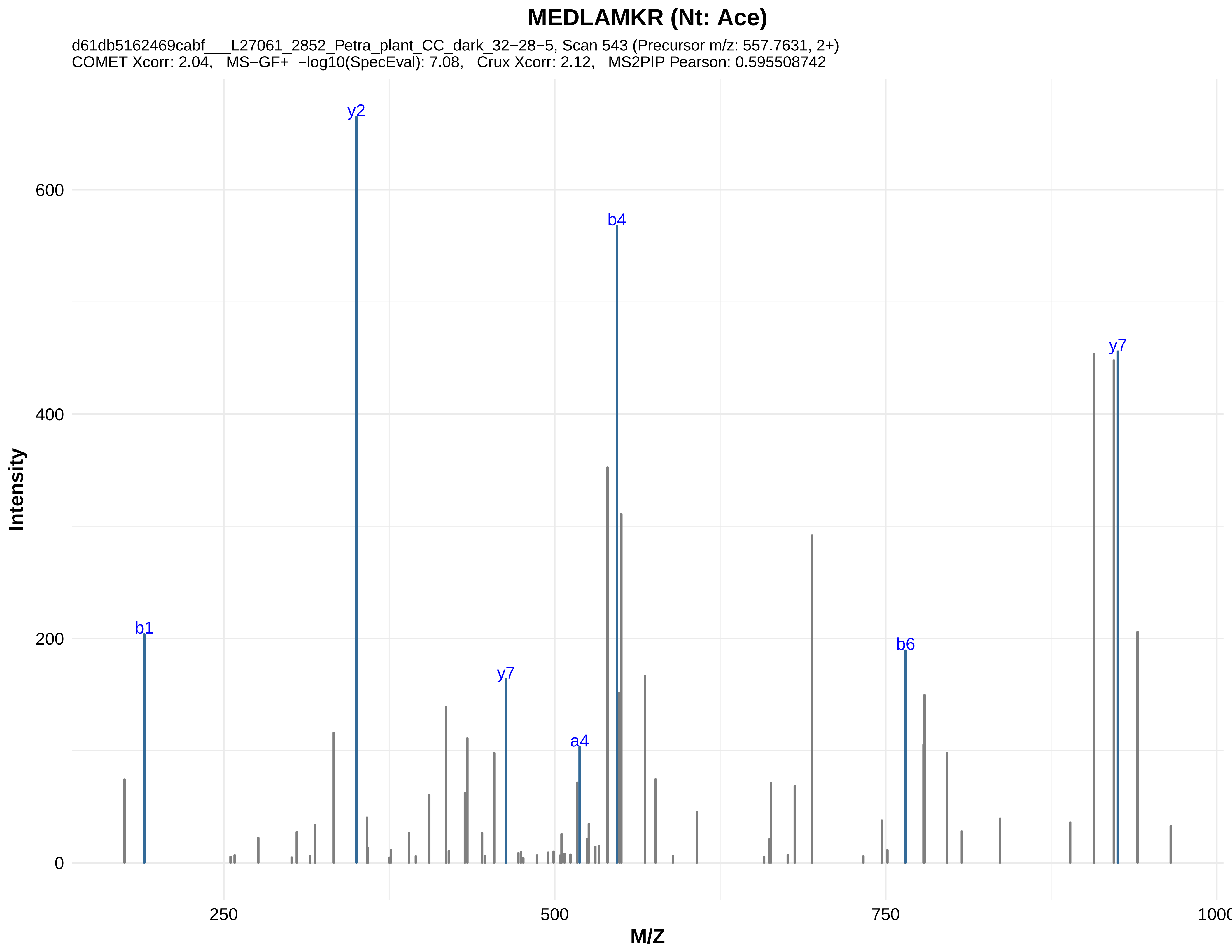
MEDLAMKR (Nt: Ace)
d61db5162469cabf___L27061_2852_Petra_plant_CC_dark_32−28−5, Scan 543 (Precursor m/z: 557.7631, 2+)
COMET Xcorr: 2.04, MS−GF+ −log10(SpecEval): 7.08, Crux Xcorr: 2.12, MS2PIP Pearson: 0.595508742
y2
b4
y7
b1
b6
y7
a4
Intensity
600
400
200
0
250
500
750
1000
M/Z

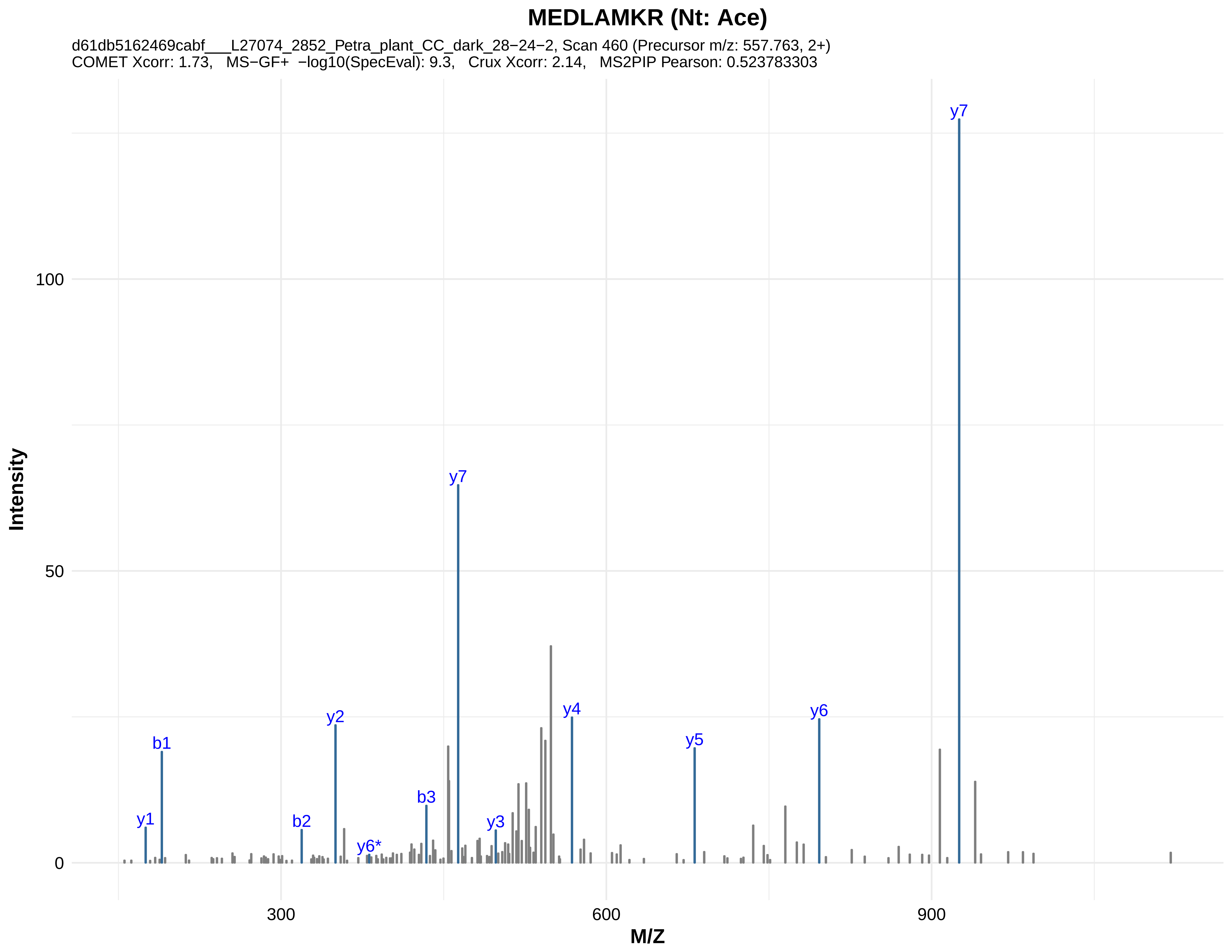

MEDLAMKR (Nt: Ace)
d61db5162469cabf___L27074_2852_Petra_plant_CC_dark_28−24−2, Scan 460 (Precursor m/z: 557.763, 2+)
COMET Xcorr: 1.73, MS−GF+ −log10(SpecEval): 9.3, Crux Xcorr: 2.14, MS2PIP Pearson: 0.523783303
Intensity
100
50
0
300
600
900
M/Z
y1
b1
b2
y2
y6*
b3
y7
y3
y4
y5
y6
y7

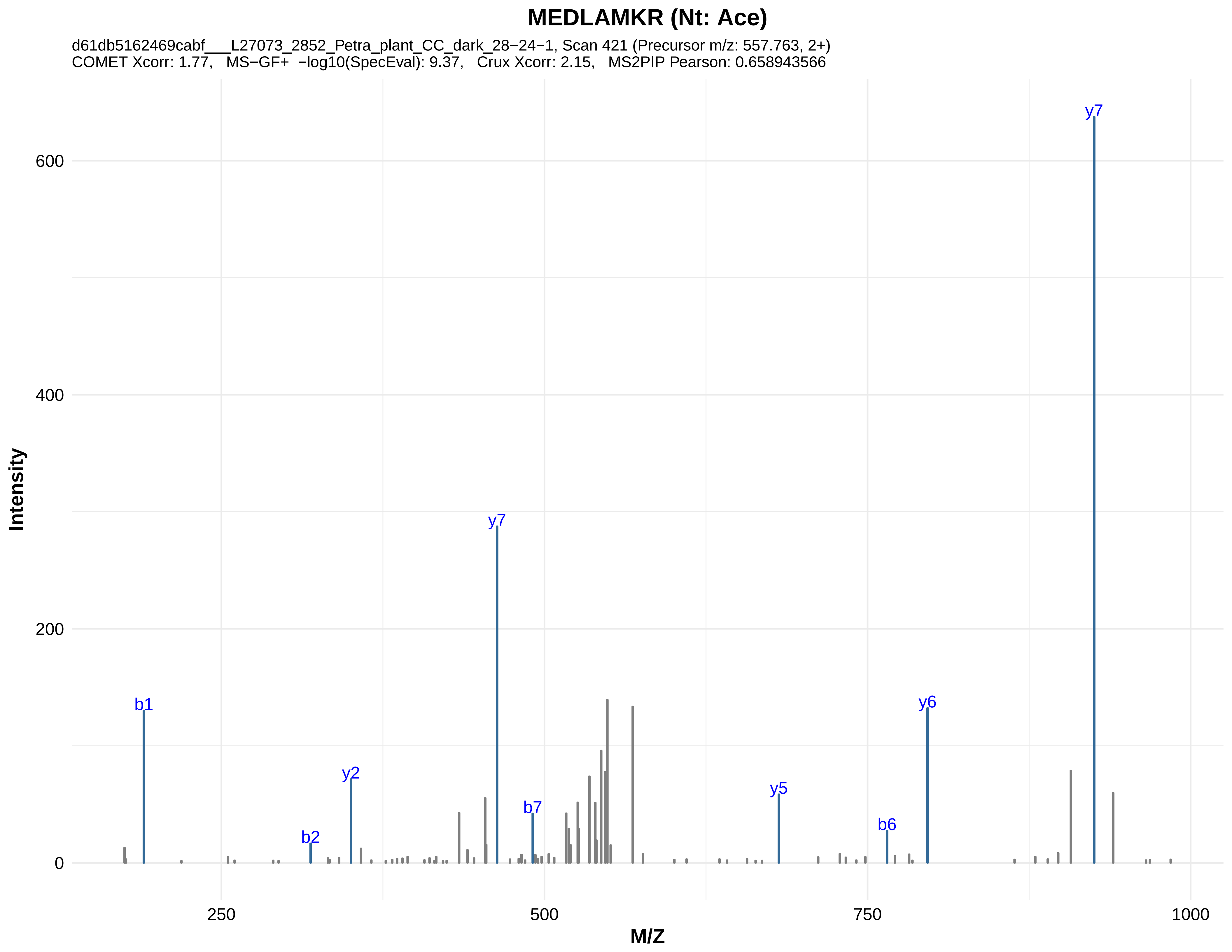

MEDLAMKR (Nt: Ace)
d61db5162469cabf___L27073_2852_Petra_plant_CC_dark_28−24−1, Scan 421 (Precursor m/z: 557.763, 2+)
COMET Xcorr: 1.77, MS−GF+ −log10(SpecEval): 9.37, Crux Xcorr: 2.15, MS2PIP Pearson: 0.658943566
Intensity
M/Z
0
200
400
600
250
500
750
1000
b1
b2
y2
y7
b7
y5
b6
y6
y7

MEEMIIDEAIDETIVEENVGELGMR (Nt: Trideutero)

8ab0e245ad1979ce___R23584_3801_1_plant_cc_tryp_no_SCX_fr_24−28−11, Scan 1394 (Precursor m/z: 987.4557, 3+)
COMET Xcorr: 1.52,   MS−GF+ −log10(SpecEval): 4.17,   Crux Xcorr: 2.14,   MS2PIP Pearson: −0.02626742

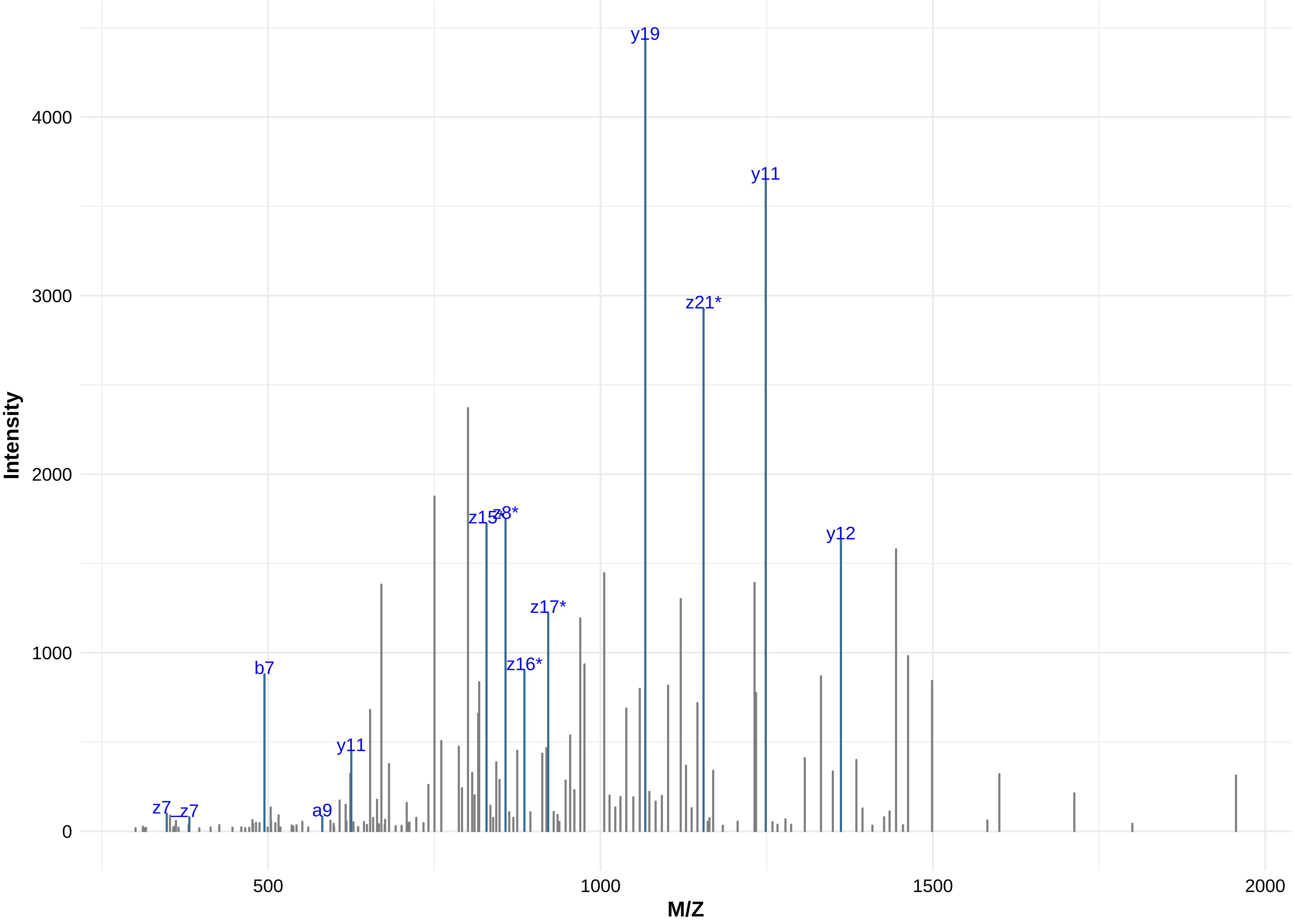

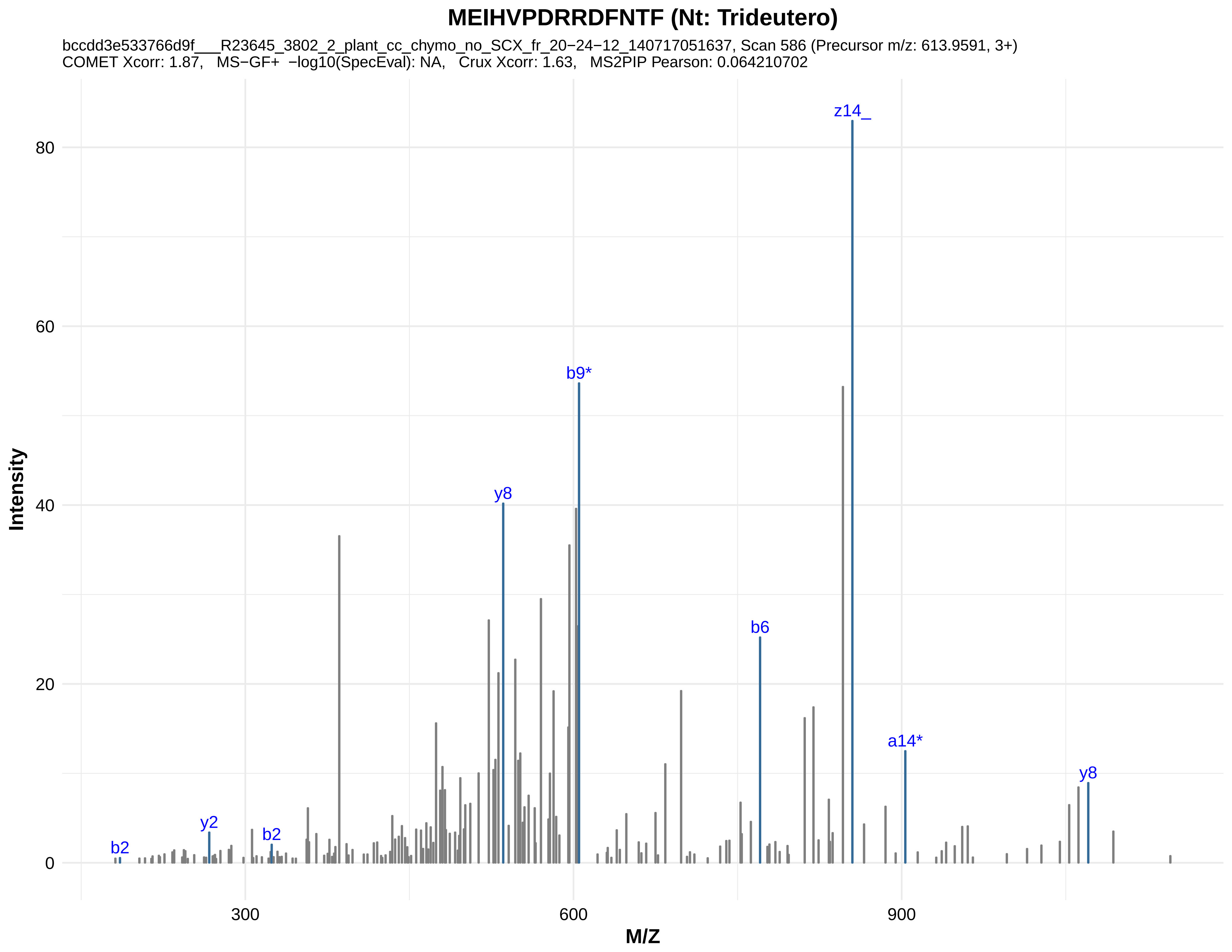

MEIHVPDRRDFNTF (Nt: Trideutero)
bccdd3e533766d9f___R23645_3802_2_plant_cc_chymo_no_SCX_fr_20−24−12_140717051637, Scan 586 (Precursor m/z: 613.9591, 3+)
COMET Xcorr: 1.87, MS−GF+ −log10(SpecEval): NA, Crux Xcorr: 1.63, MS2PIP Pearson: 0.064210702
Intensity
M/Z
z14_
b9*
y8
b6
a14*
y8
b2
y2
b2
0
20
40
60
80
300
600
900

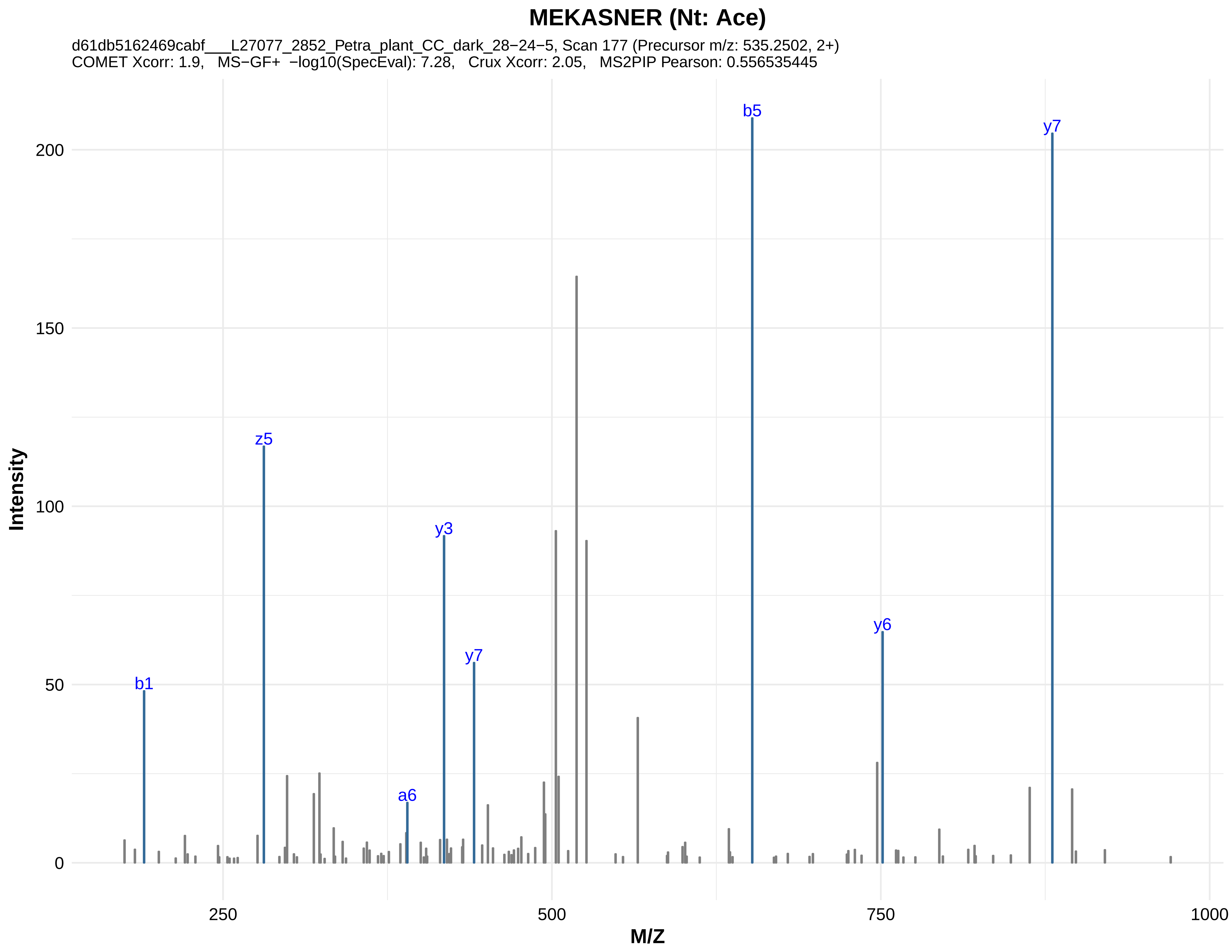
MEKASNER (Nt: Ace)
d61db5162469cabf___L27077_2852_Petra_plant_CC_dark_28−24−5, Scan 177 (Precursor m/z: 535.2502, 2+)
COMET Xcorr: 1.9, MS−GF+ −log10(SpecEval): 7.28, Crux Xcorr: 2.05, MS2PIP Pearson: 0.556535445
Intensity
M/Z
b1
z5
a6
y3
y7
b5
y6
y7
0
50
100
150
200
250
500
750
1000

MEMPAATCL (Nt: Ace)

0fdf8708e3b3bf53___R23711_3805_4_plant_cc_AspN_no_SCX_fr_24−28−3, Scan 973 (Precursor m/z: 549.2185, 2+)
COMET Xcorr: 1.96,   MS−GF+ −log10(SpecEval): NA,   Crux Xcorr: 1.78,   MS2PIP Pearson: 0.404038638

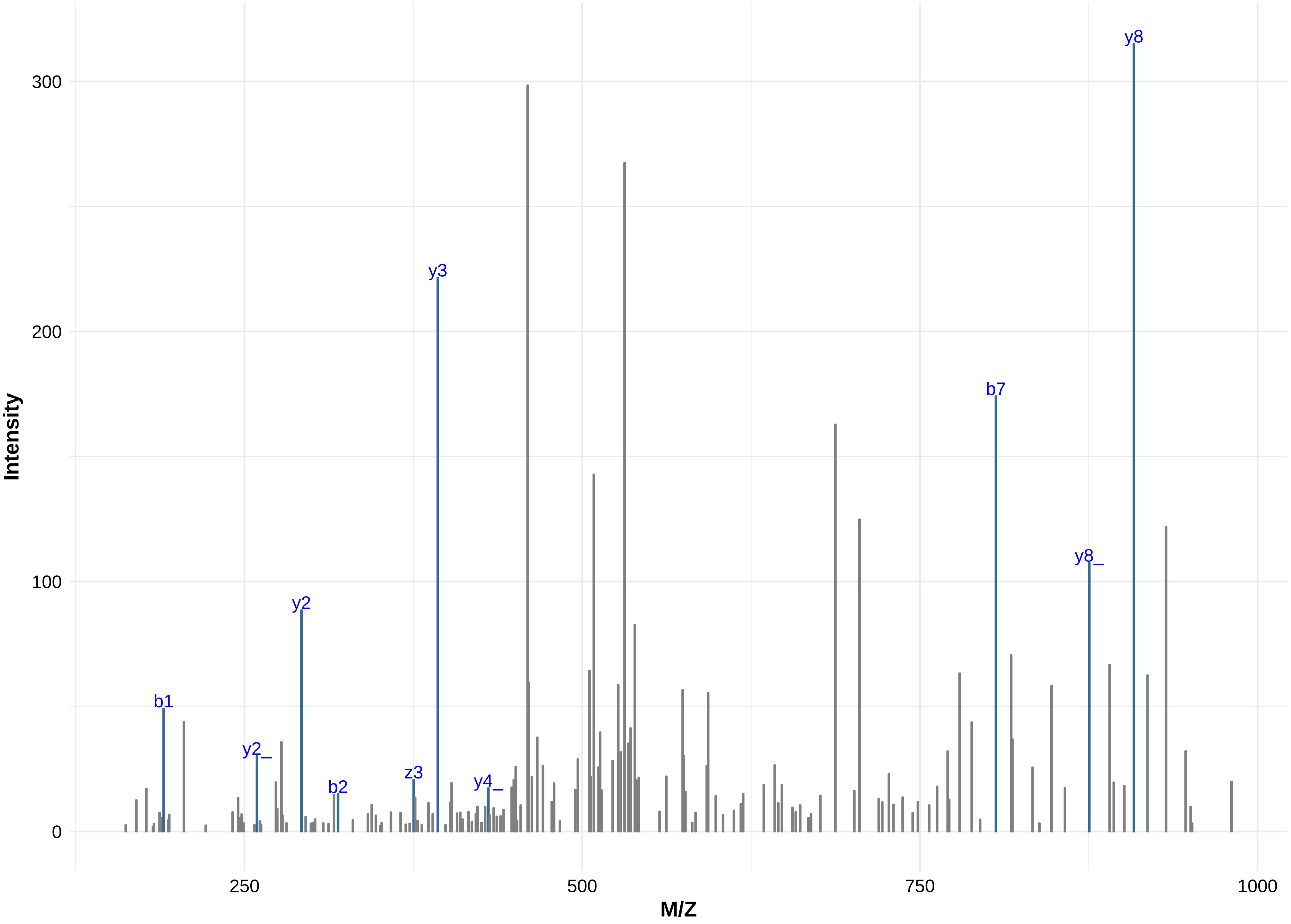

MESANLNIGM (Nt: Ace)

bccdd3e533766d9f___R23611_3802_2_plant_cc_chymo_no_SCX_fr_28−32−8_140715170103, Scan 588 (Precursor m/z: 577.2467, 2+)
COMET Xcorr: 1.62, MS−GF+ −log10(SpecEval): NA, Crux Xcorr: 1.92, MS2PIP Pearson: 0.394338042

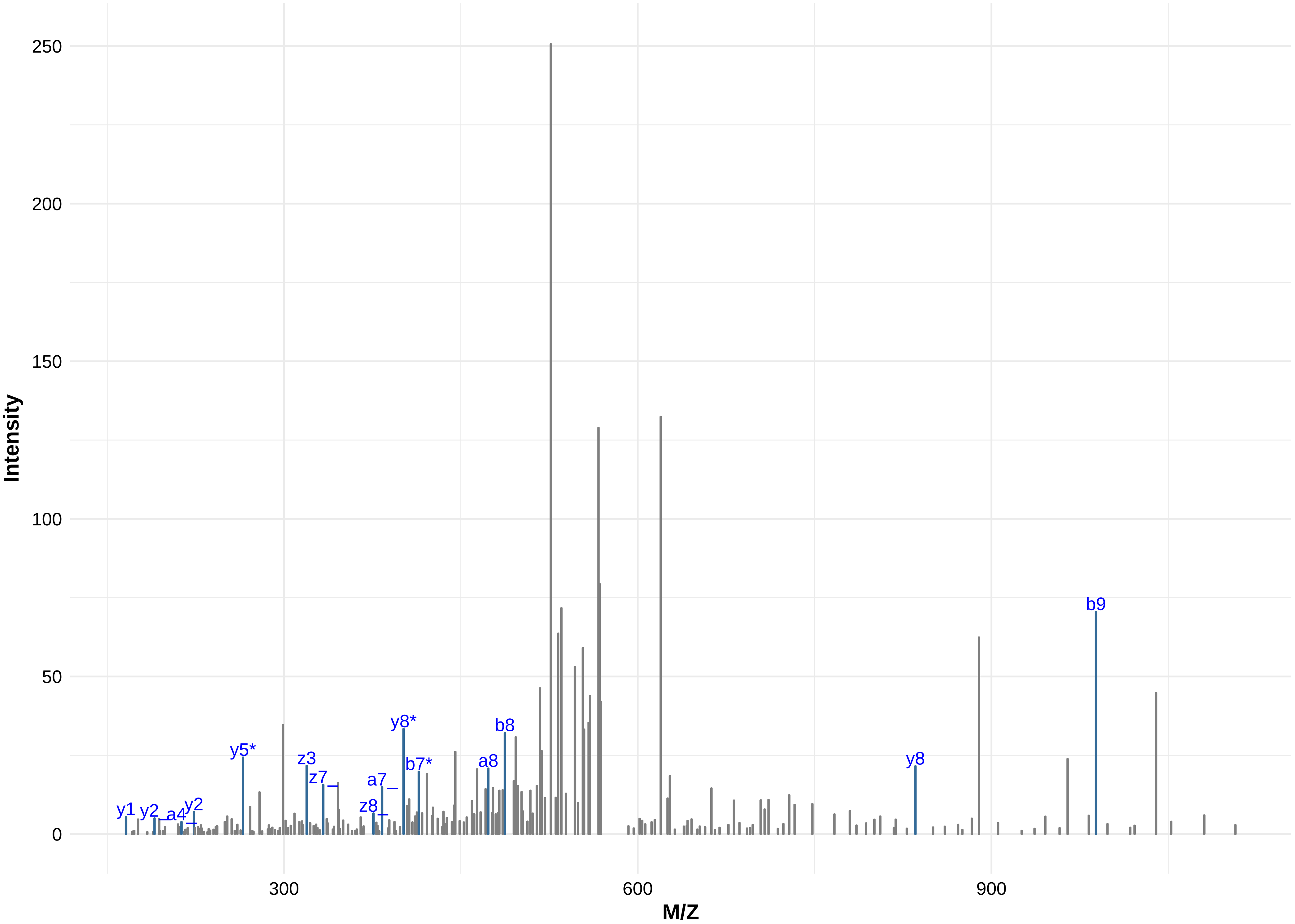

METKMTSHGR (Nt: Ace)

8ab0e245ad1979ce___R23592_3801_1_plant_cc_tryp_no_SCX_fr_20−24−4, Scan 53 (Precursor m/z: 649.7942, 2+)
COMET Xcorr: 2.36,   MS−GF+ −log10(SpecEval): 11.82,   Crux Xcorr: 2.33,   MS2PIP Pearson: 0.74978014

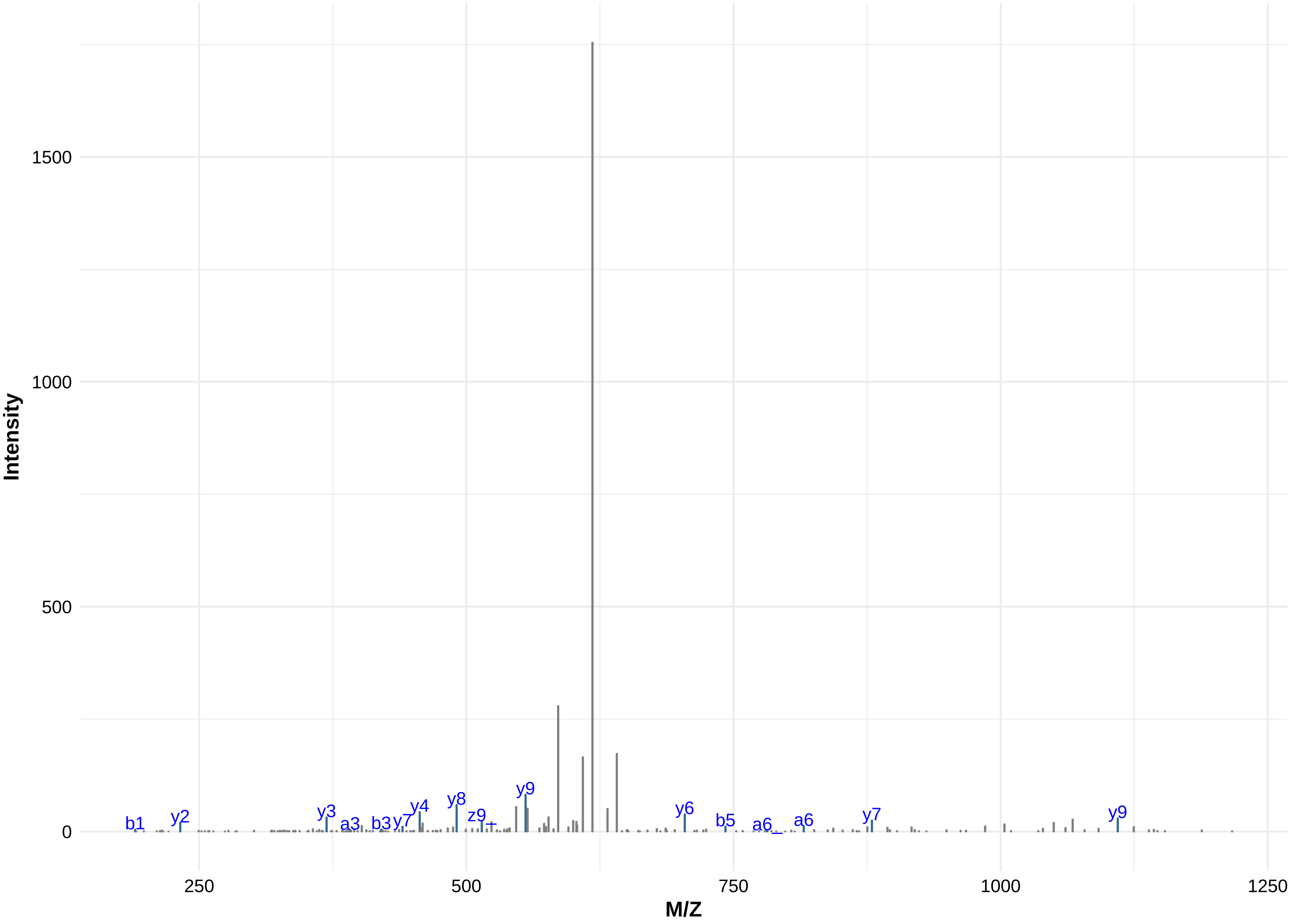

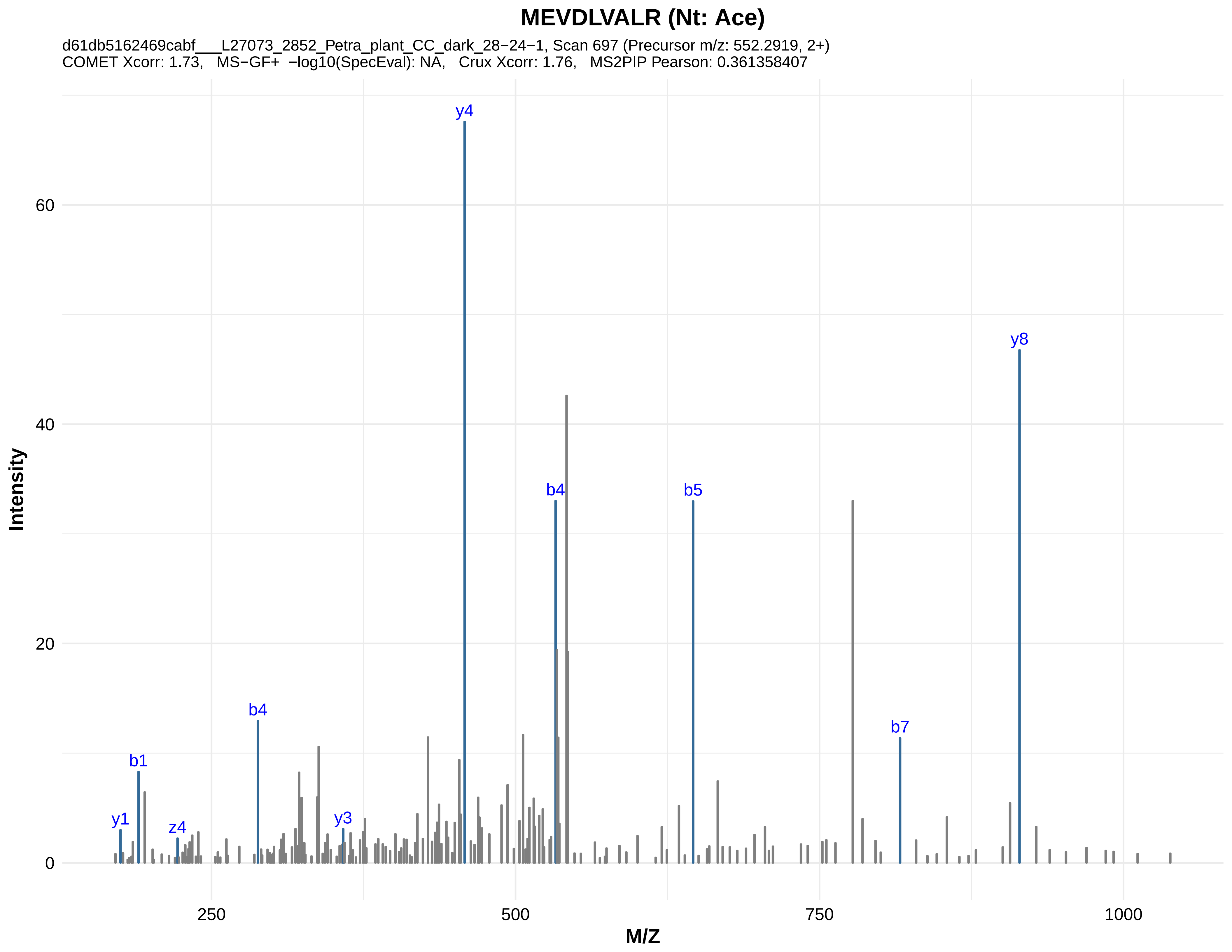
MEVDLVALR (Nt: Ace)
d61db5162469cabf___L27073_2852_Petra_plant_CC_dark_28−24−1, Scan 697 (Precursor m/z: 552.2919, 2+)
COMET Xcorr: 1.73, MS−GF+ −log10(SpecEval): NA, Crux Xcorr: 1.76, MS2PIP Pearson: 0.361358407
Intensity
M/Z
0
20
40
60
250
500
750
1000
y1
b1
z4
b4
y3
y4
b4
b5
b7
y8

MFGGNDYTR (Nt: Ace)

d61db5162469cabf___L27093_2852_Petra_plant_CC_dark_24−20−5, Scan 489 (Precursor m/z: 559.7322, 2+)
COMET Xcorr: 2.31, MS−GF+ −log10(SpecEval): 8.74, Crux Xcorr: 2.03, MS2PIP Pearson: 0.733754187

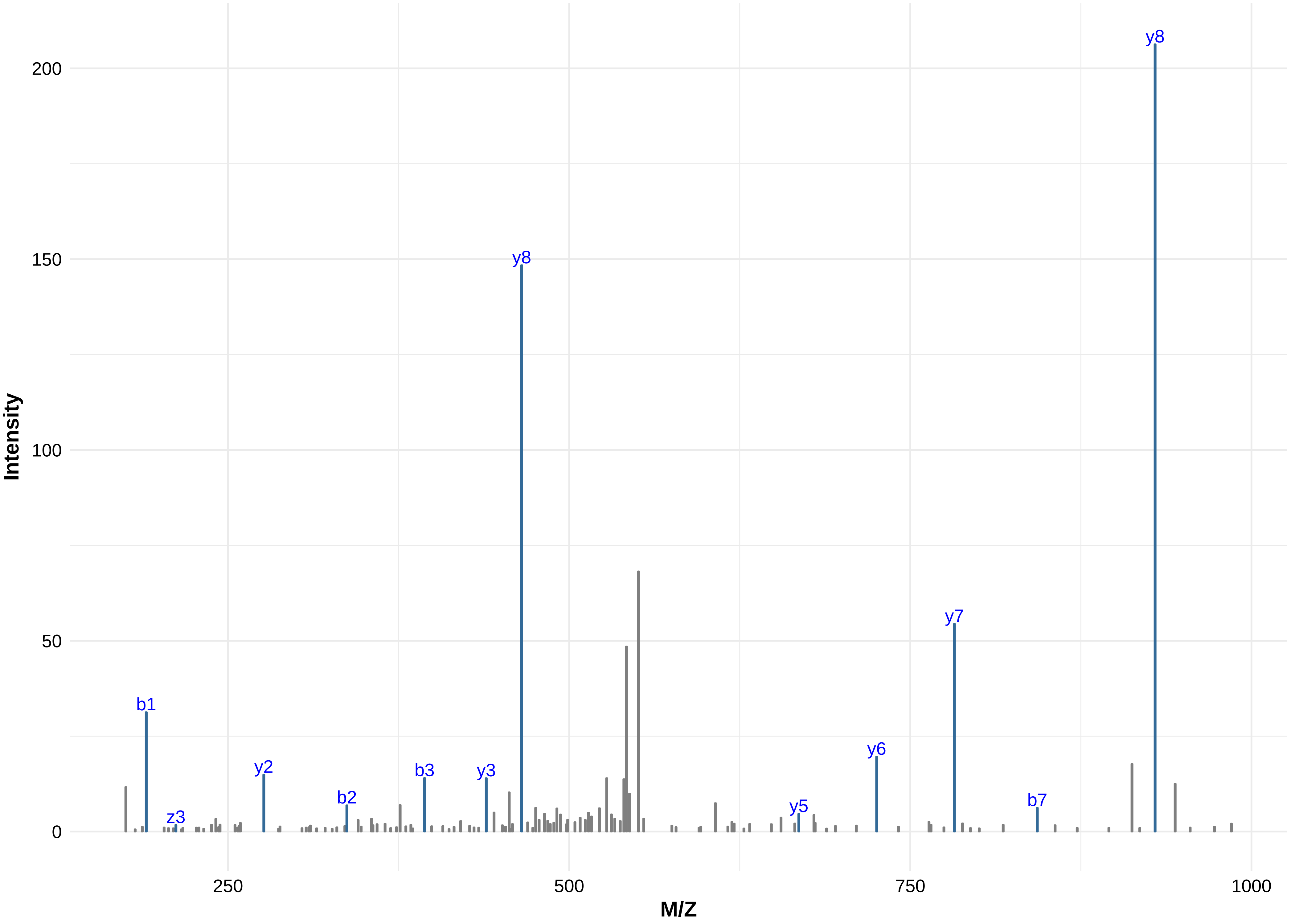

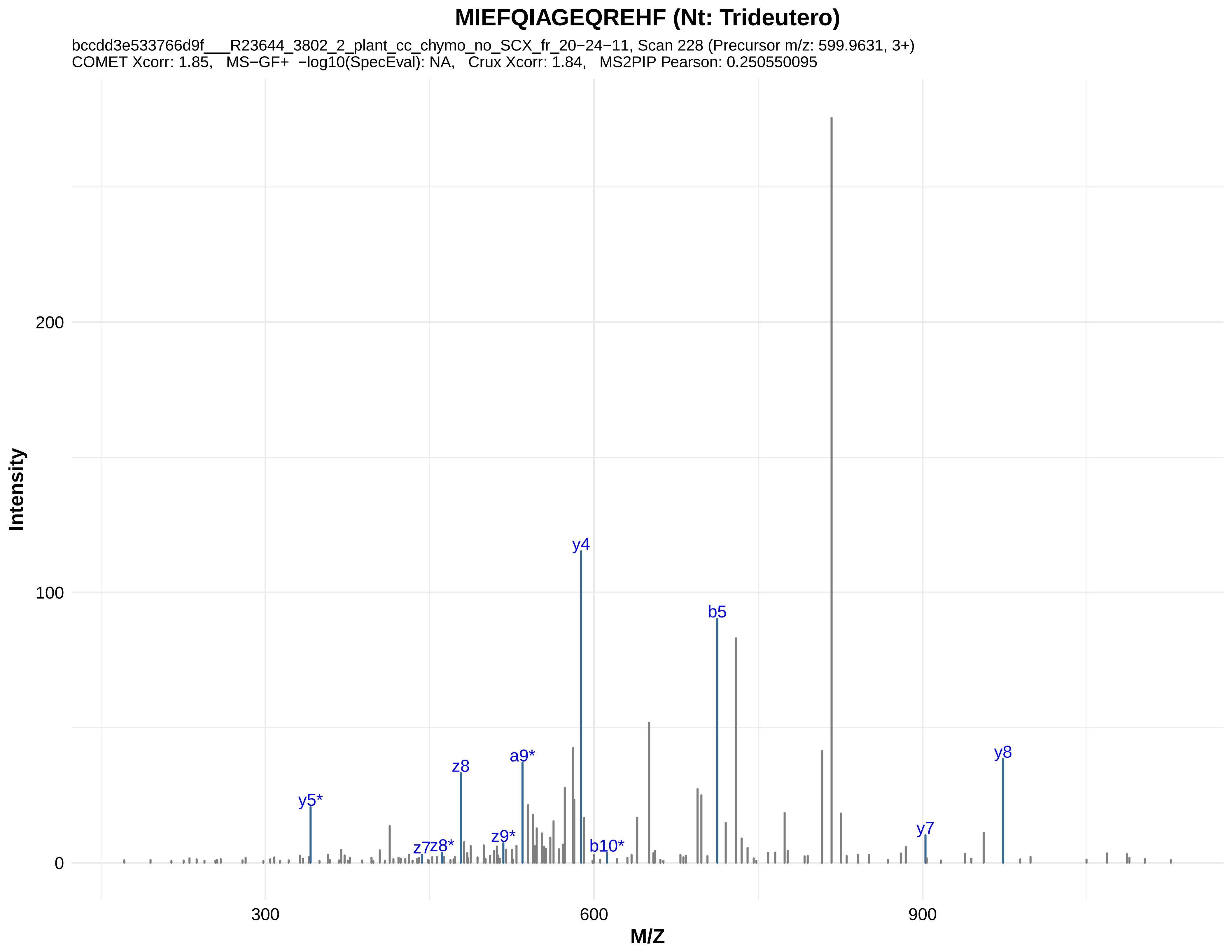

MIEFQIAGEQREHF (Nt: Trideutero)
bccdd3e533766d9f___R23644_3802_2_plant_cc_chymo_no_SCX_fr_20−24−11, Scan 228 (Precursor m/z: 599.9631, 3+)
COMET Xcorr: 1.85, MS−GF+ −log10(SpecEval): NA, Crux Xcorr: 1.84, MS2PIP Pearson: 0.250550095
Intensity
M/Z
0
100
200
300
600
900
y5*
z7
z8*
z8
z9*
a9*
y4
b10*
b5
y7
y8

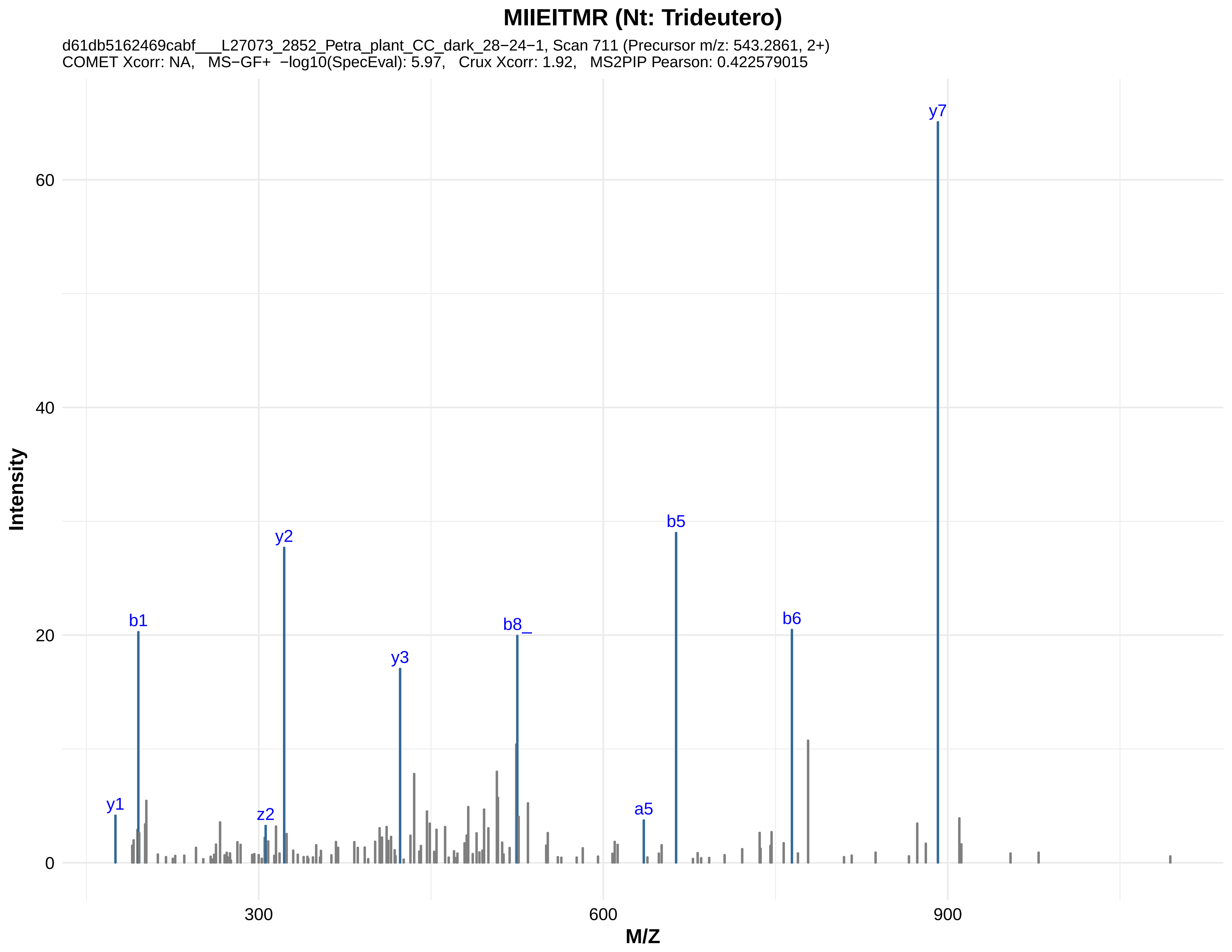

MIIEITMR (Nt: Trideutero)
d61db5162469cabf___L27073_2852_Petra_plant_CC_dark_28−24−1, Scan 711 (Precursor m/z: 543.2861, 2+)
COMET Xcorr: NA, MS−GF+ −log10(SpecEval): 5.97, Crux Xcorr: 1.92, MS2PIP Pearson: 0.422579015
Intensity
M/Z
y1
b1
z2
y2
y3
b8_
a5
b5
b6
y7

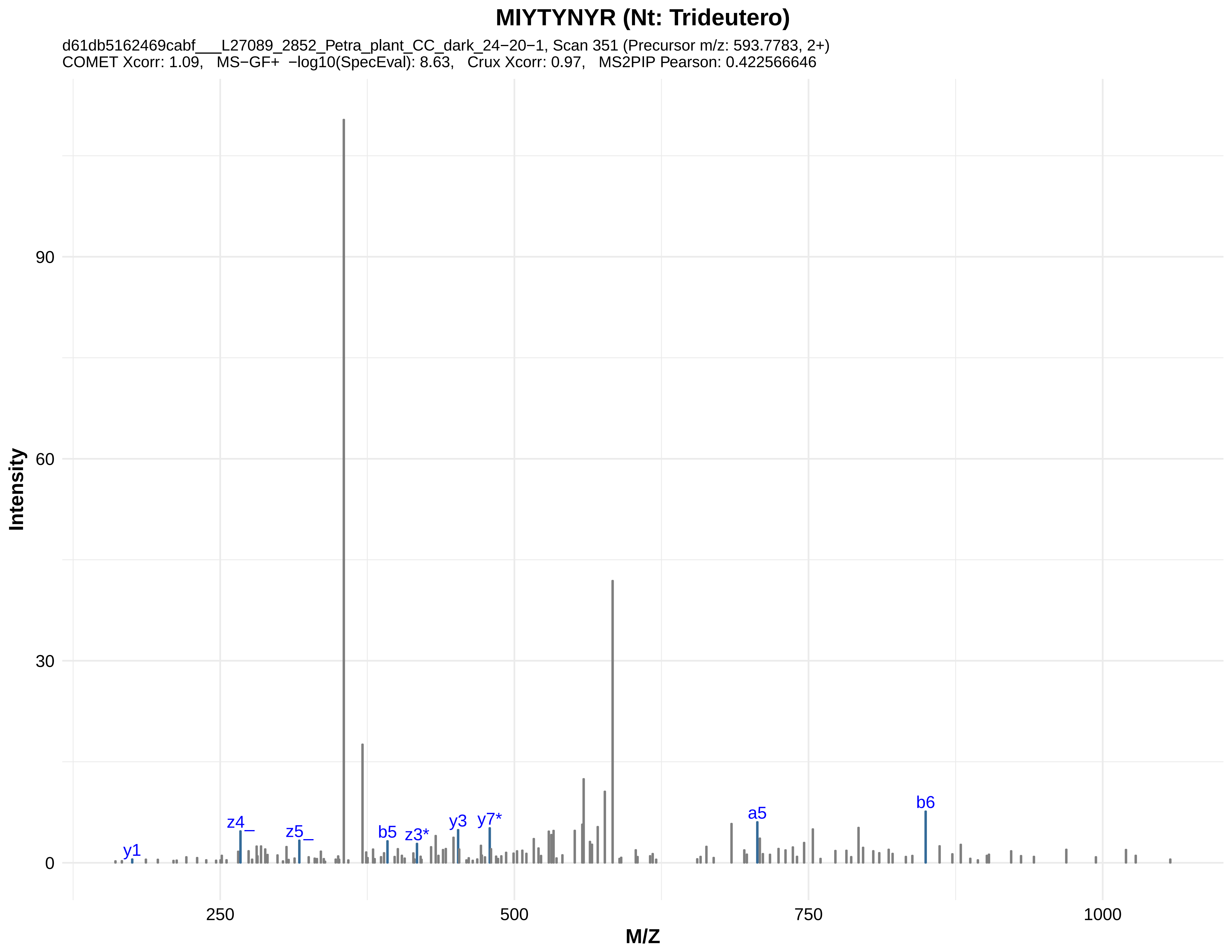

MIYTYNYR (Nt: Trideutero)
d61db5162469cabf___L27089_2852_Petra_plant_CC_dark_24−20−1, Scan 351 (Precursor m/z: 593.7783, 2+)
COMET Xcorr: 1.09, MS−GF+ −log10(SpecEval): 8.63, Crux Xcorr: 0.97, MS2PIP Pearson: 0.422566646
Intensity
M/Z
0
30
60
90
250
500
750
1000
y1
z4_
z5_
b5
z3*
y3
y7*
a5
b6

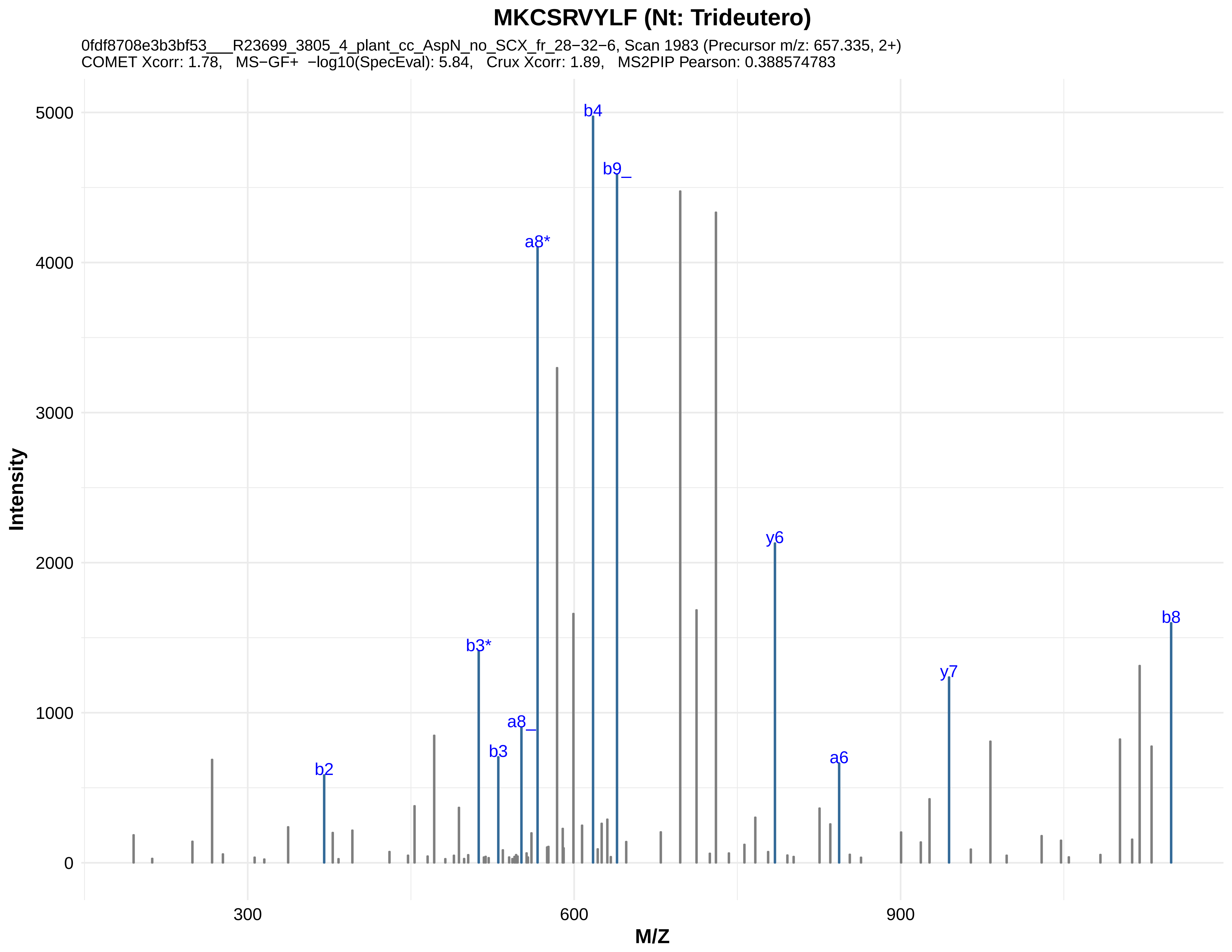

MKCSRVYLF (Nt: Trideutero)
0fdf8708e3b3bf53___R23699_3805_4_plant_cc_AspN_no_SCX_fr_28−32−6, Scan 1983 (Precursor m/z: 657.335, 2+)
COMET Xcorr: 1.78, MS−GF+ −log10(SpecEval): 5.84, Crux Xcorr: 1.89, MS2PIP Pearson: 0.388574783
Intensity
M/Z
b4
b9_
a8*
y6
b8
b3*
y7
a8_
b3
a6
b2

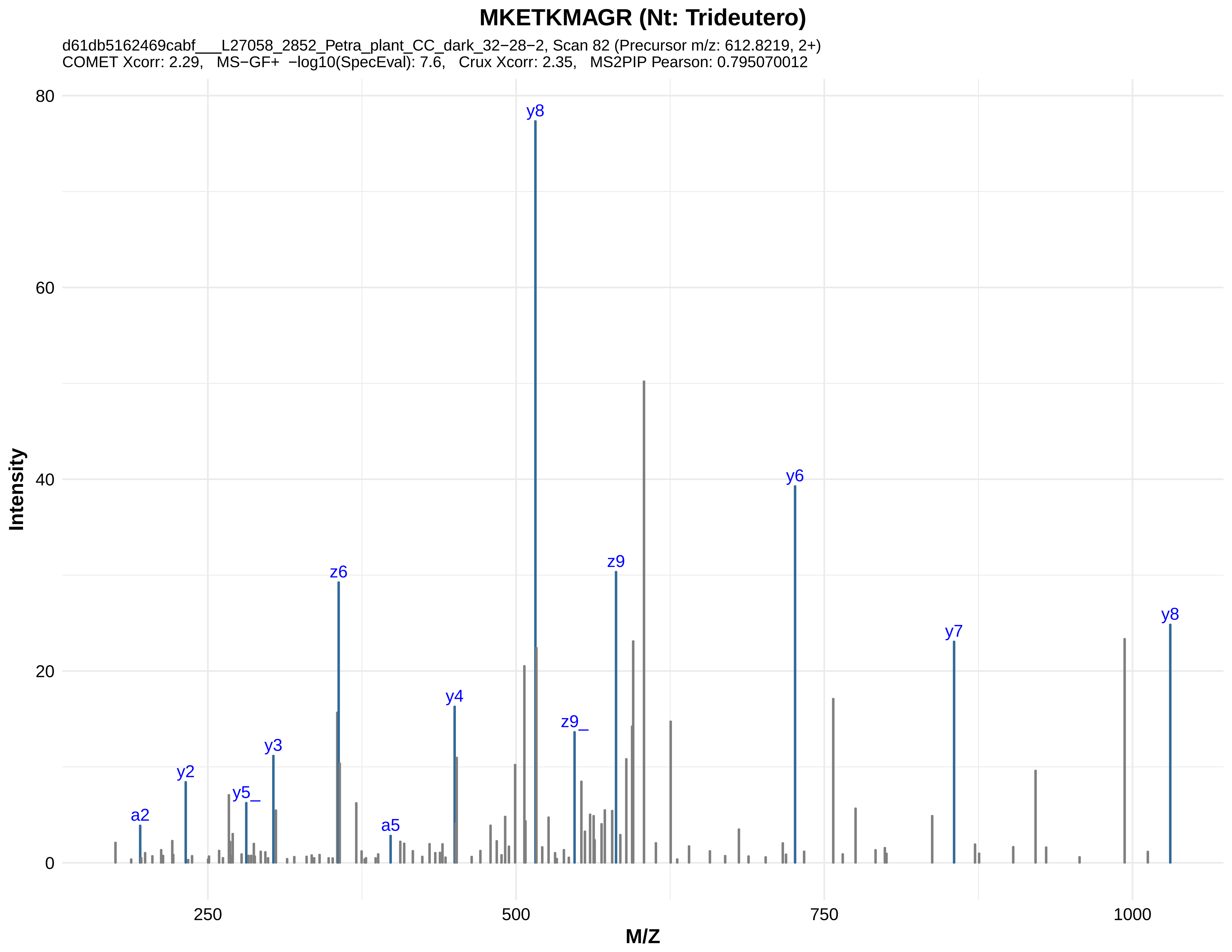
MKETKMAGR (Nt: Trideutero)
d61db5162469cabf___L27058_2852_Petra_plant_CC_dark_32−28−2, Scan 82 (Precursor m/z: 612.8219, 2+)
COMET Xcorr: 2.29, MS−GF+ −log10(SpecEval): 7.6, Crux Xcorr: 2.35, MS2PIP Pearson: 0.795070012
Intensity
M/Z
0
20
40
60
80
250
500
750
1000
y8
y6
z9
z6
y8
y7
y4
z9_
y3
y2
y5_
a2
a5

MLDYSDVGIFR (Nt: Trideutero)

d61db5162469cabf___L27090_2852_Petra_plant_CC_dark_24−20−2, Scan 768 (Precursor m/z: 689.8373, 2+)
COMET Xcorr: 2.24,   MS−GF+ −log10(SpecEval): 6.2,   Crux Xcorr: 2.17,   MS2PIP Pearson: 0.647773252

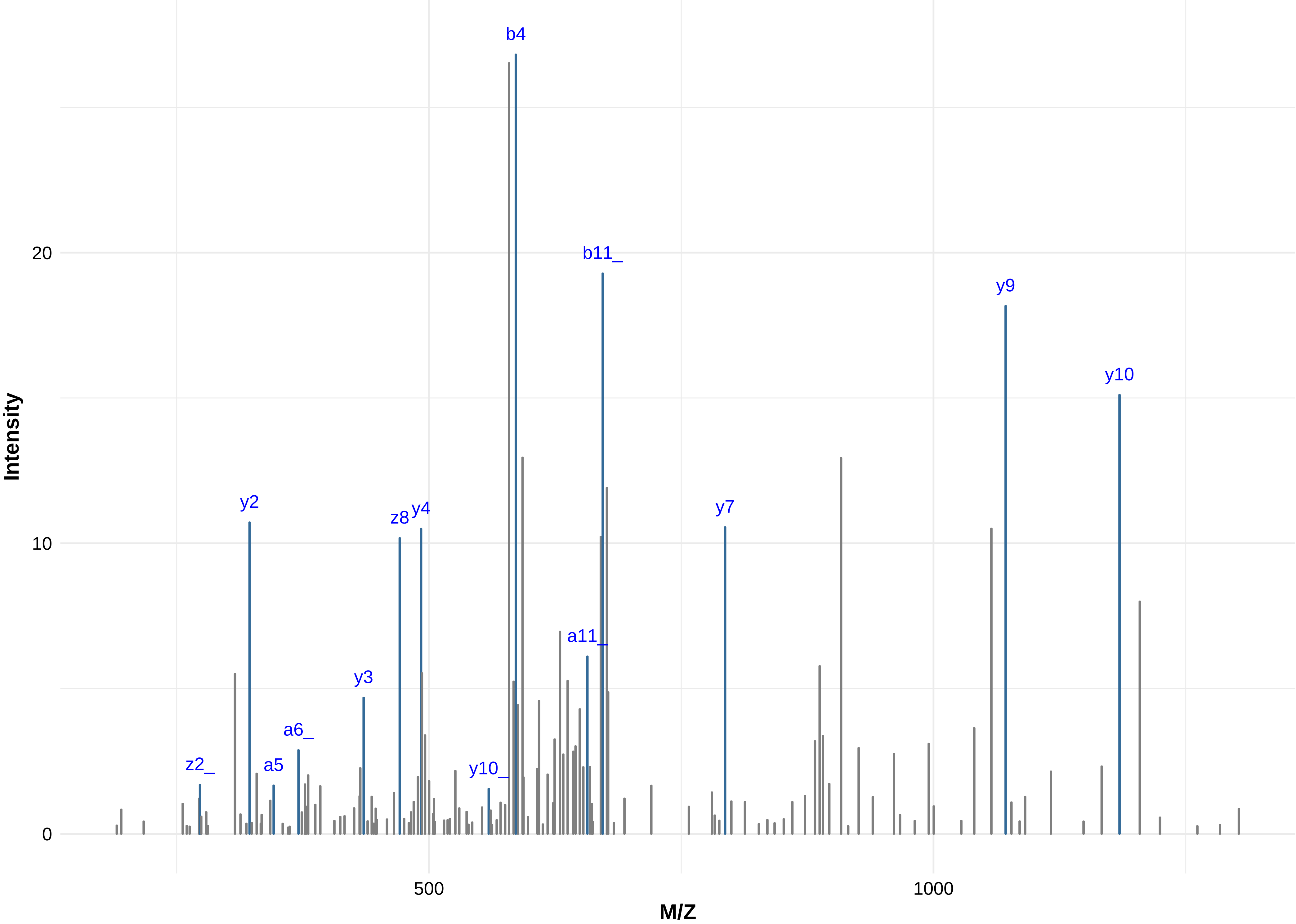

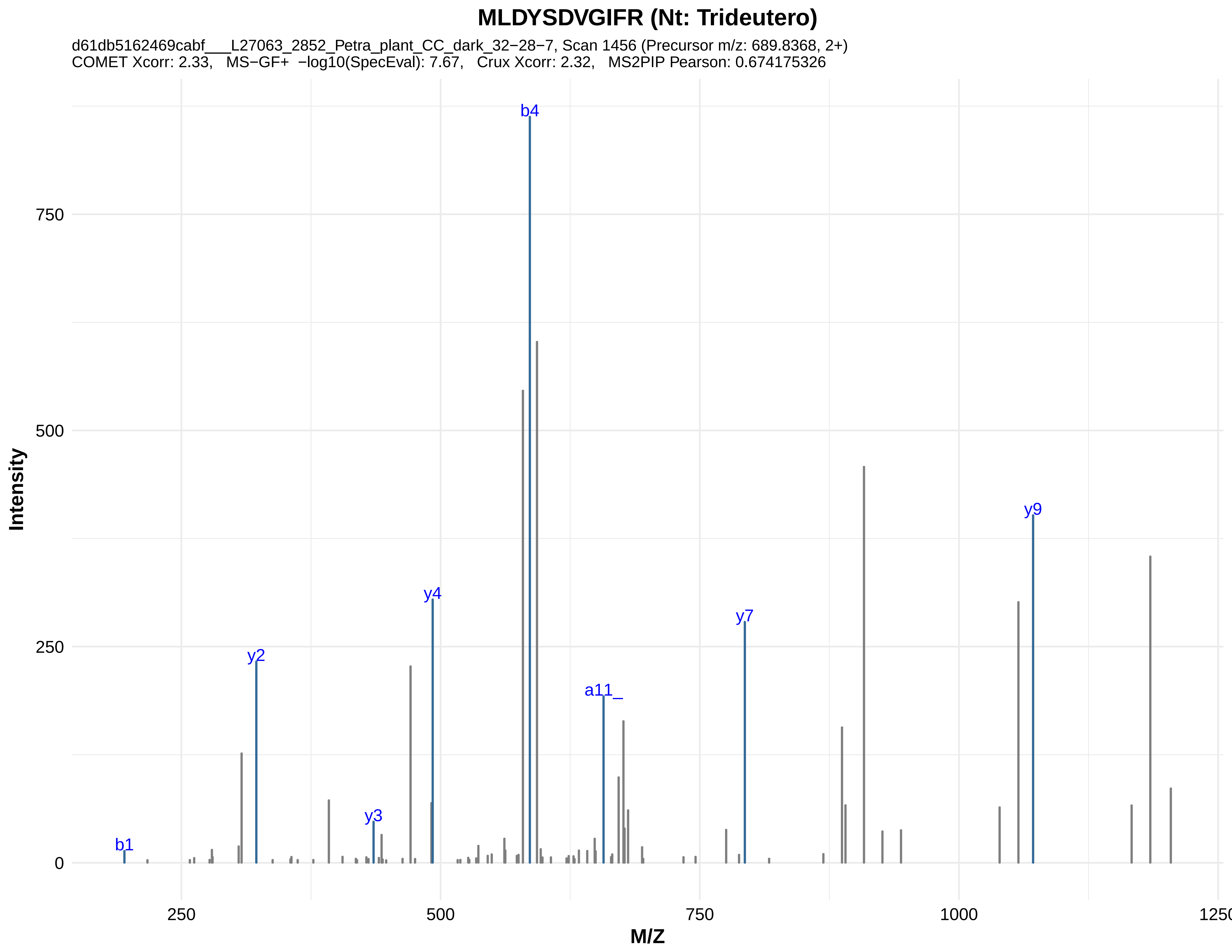
MLDYSDVGIFR (Nt: Trideutero)
d61db5162469cabf___L27063_2852_Petra_plant_CC_dark_32−28−7, Scan 1456 (Precursor m/z: 689.8368, 2+)
COMET Xcorr: 2.33, MS−GF+ −log10(SpecEval): 7.67, Crux Xcorr: 2.32, MS2PIP Pearson: 0.674175326
b4
y9
y4
y7
y2
a11_
y3
b1
Intensity
M/Z
0
250
500
750
250
500
750
1000
1250

MLDYSDVGIFR (Nt: Trideutero)

d61db5162469cabf___L27103_2852_Petra_plant_CC_dark_24−20−15, Scan 1300 (Precursor m/z: 689.8371, 2+)
COMET Xcorr: 2.54,   MS−GF+ −log10(SpecEval): 7.44,   Crux Xcorr: 2.15,   MS2PIP Pearson: 0.683985103

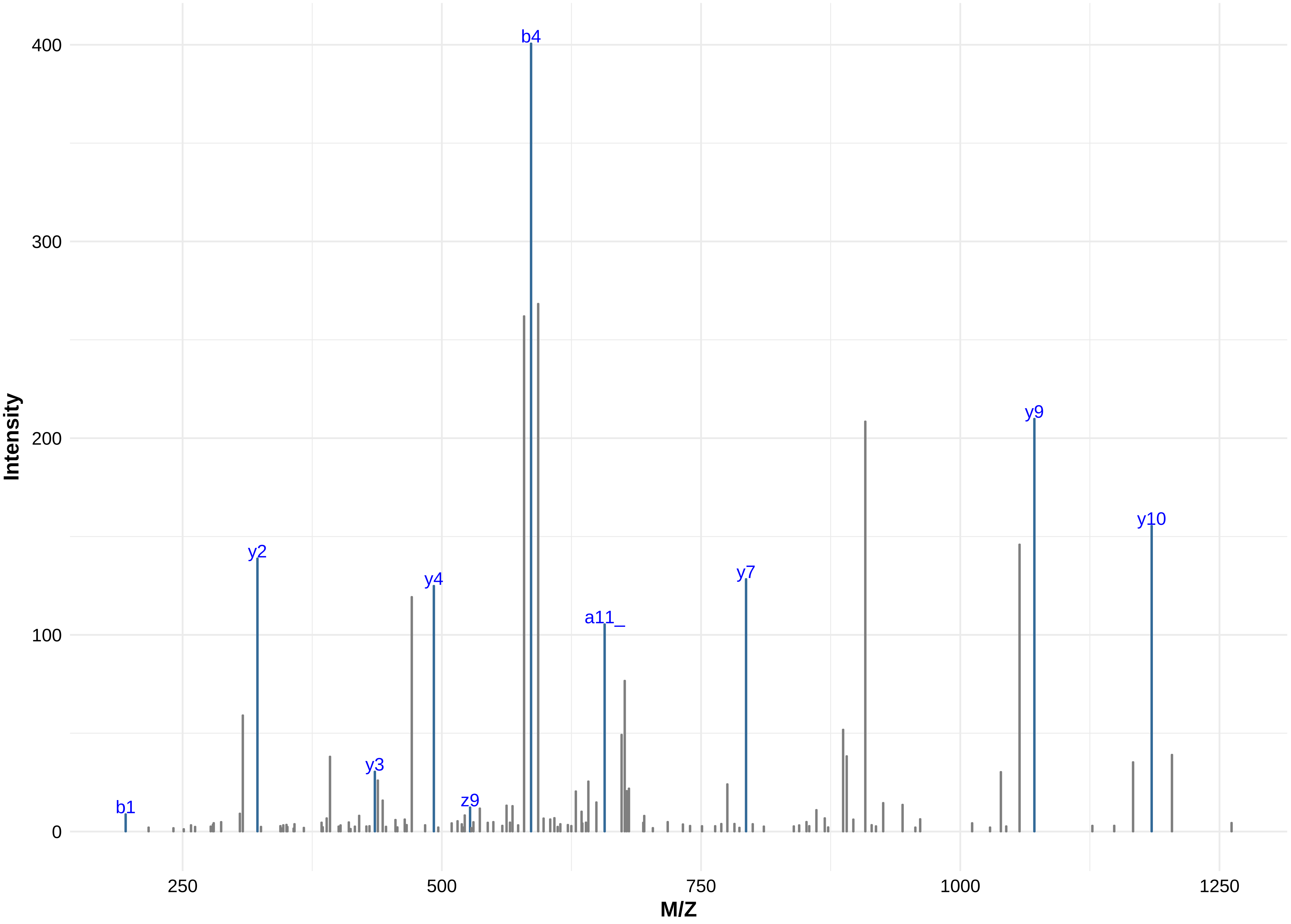

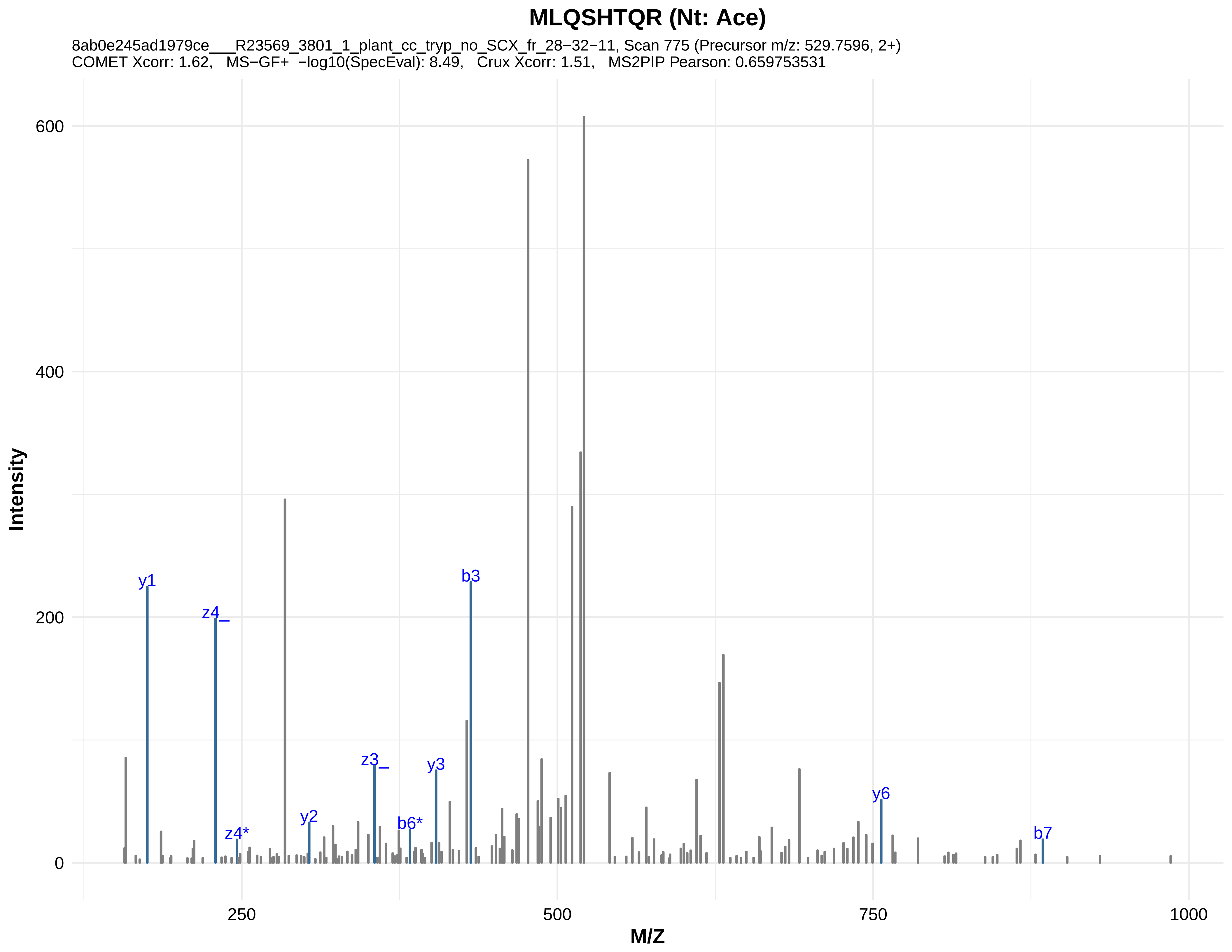
MLQSHTQR (Nt: Ace)
8ab0e245ad1979ce___R23569_3801_1_plant_cc_tryp_no_SCX_fr_28−32−11, Scan 775 (Precursor m/z: 529.7596, 2+)
COMET Xcorr: 1.62, MS−GF+ −log10(SpecEval): 8.49, Crux Xcorr: 1.51, MS2PIP Pearson: 0.659753531
Intensity
M/Z
y1
z4_
z4*
y2
z3_
b6*
y3
b3
y6
b7
0
200
400
600
250
500
750
1000

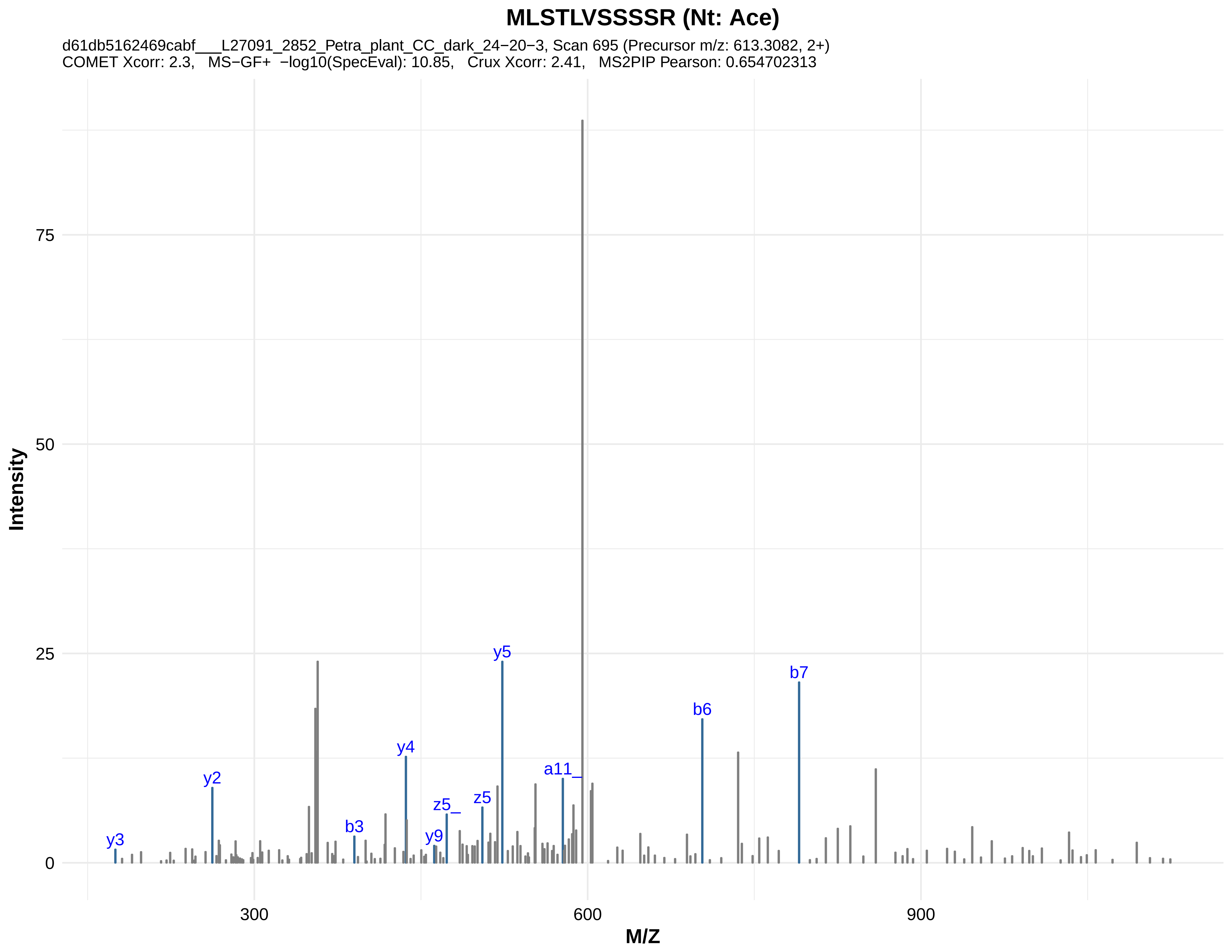
MLSTLVSSSSR (Nt: Ace)
d61db5162469cabf___L27091_2852_Petra_plant_CC_dark_24−20−3, Scan 695 (Precursor m/z: 613.3082, 2+)
COMET Xcorr: 2.3,   MS−GF+ −log10(SpecEval): 10.85,   Crux Xcorr: 2.41,   MS2PIP Pearson: 0.654702313
Intensity
75
50
25
0
300
600
900
M/Z
y3
y2
b3
y4
y9
z5_
z5
y5
a11_
b6
b7

MMKRAQQSARETKQEAK (Nt: Ace)

0fdf8708e3b3bf53___R23725_3805_4_plant_cc_AspN_no_SCX_fr_20−24−2, Scan 598 (Precursor m/z: 746.053, 3+)
COMET Xcorr: 2.3, MS−GF+ −log10(SpecEval): 10.4, Crux Xcorr: 2.02, MS2PIP Pearson: 0.460227129

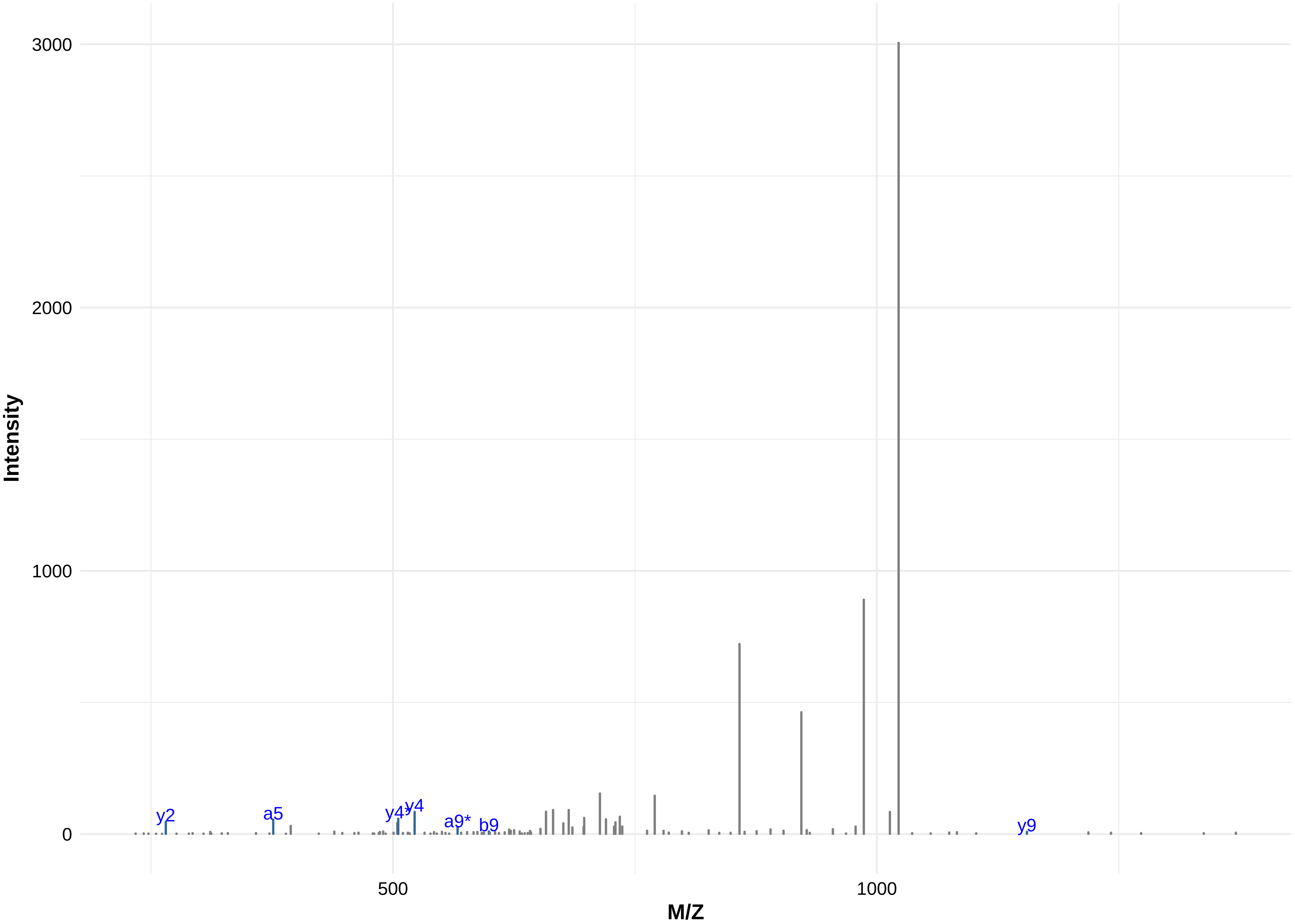

MMPEKTIISSESSNSSPLNPSSTR (Nt: Ace)

8ab0e245ad1979ce___R23589_3801_1_plant_cc_tryp_no_SCX_fr_20−24−1, Scan 1055 (Precursor m/z: 901.09, 3+)
COMET Xcorr: 1.94, MS−GF+ −log10(SpecEval): 6.07, Crux Xcorr: 2.61, MS2PIP Pearson: 0.458076528

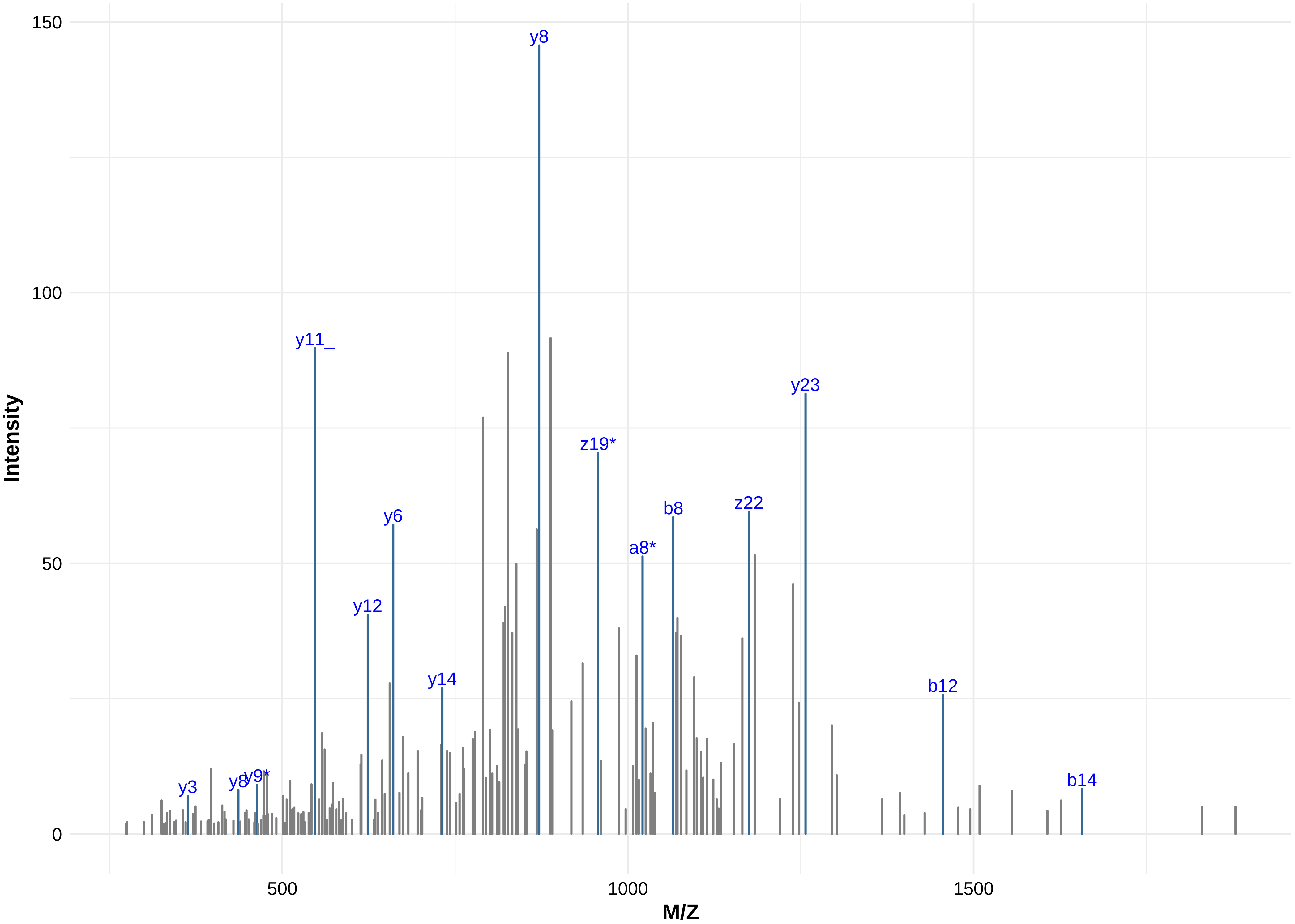

MMSQSSGSDANSISR (Nt: Ace)

d61db5162469cabf___L27096_2852_Petra_plant_CC_dark_24−20−8, Scan 220 (Precursor m/z: 816.3354, 2+)
COMET Xcorr: 3.23, MS−GF+ −log10(SpecEval): 14.46, Crux Xcorr: 2.95, MS2PIP Pearson: 0.83054017

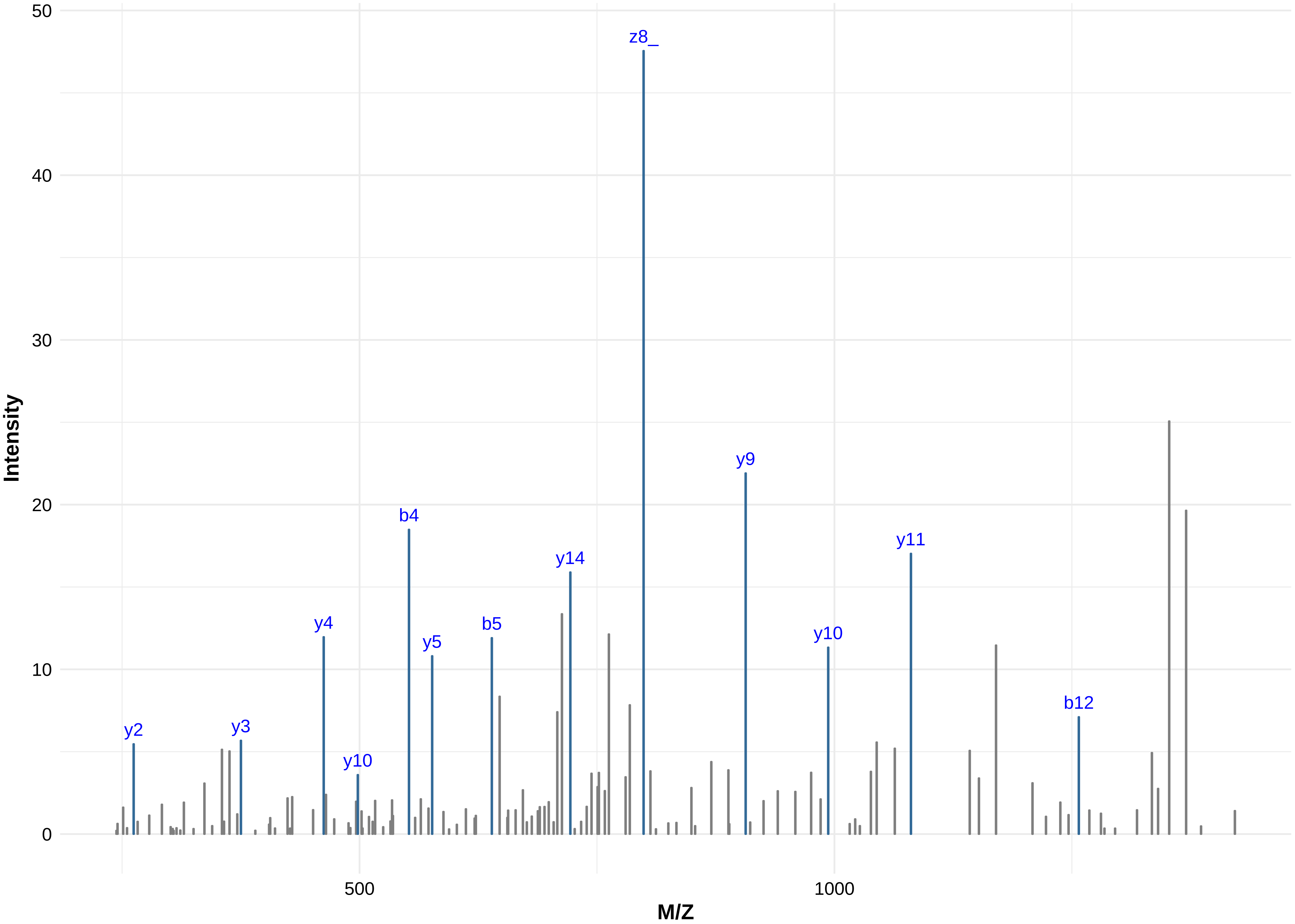

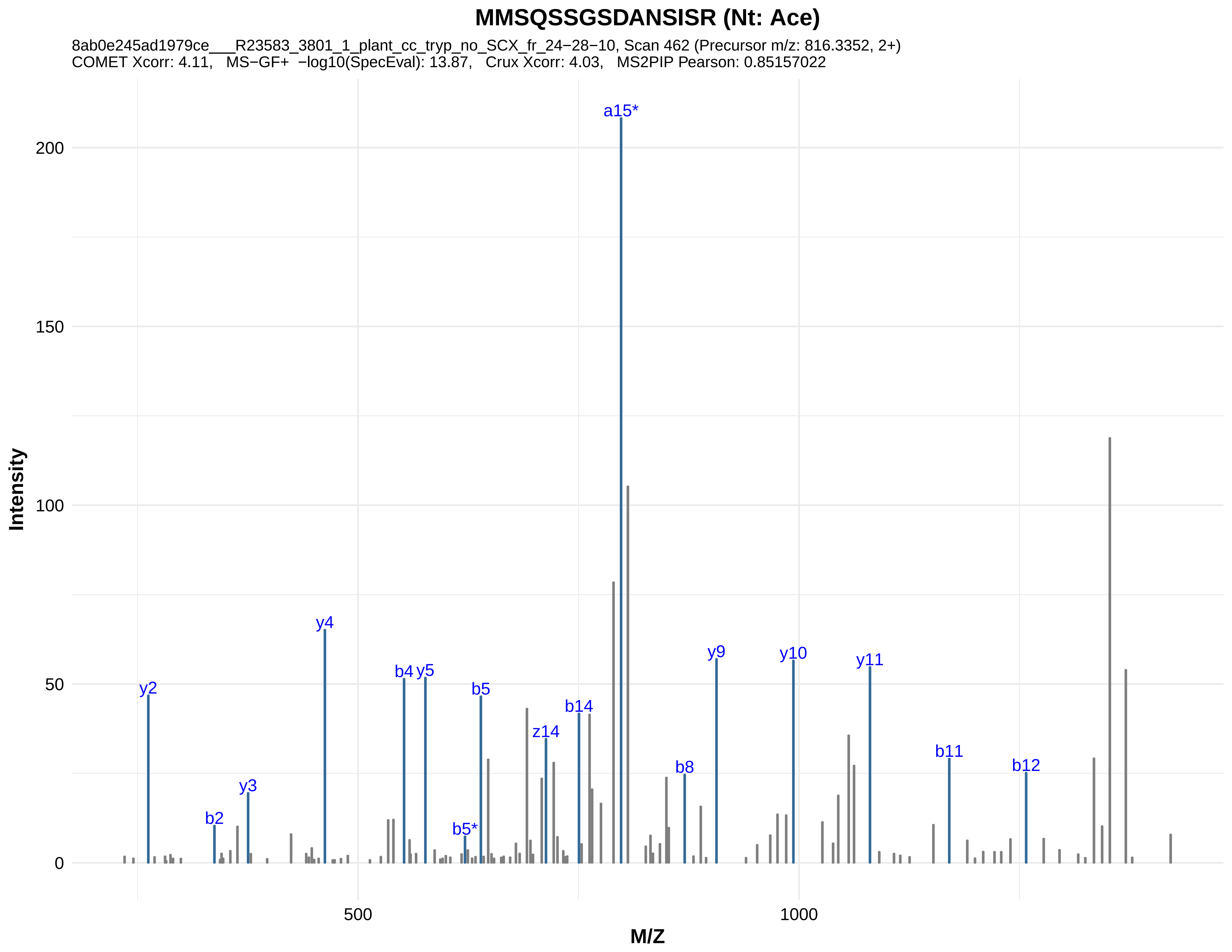
MMSQSSGSDANSISR (Nt: Ace)
8ab0e245ad1979ce___R23583_3801_1_plant_cc_tryp_no_SCX_fr_24−28−10, Scan 462 (Precursor m/z: 816.3352, 2+)
COMET Xcorr: 4.11, MS−GF+ −log10(SpecEval): 13.87, Crux Xcorr: 4.03, MS2PIP Pearson: 0.85157022
a15*
y2
b2
y3
y4
b4
y5
b5*
b5
z14
b14
b8
y9
y10
y11
b11
b12
Intensity
0
50
100
150
200
M/Z
500
1000

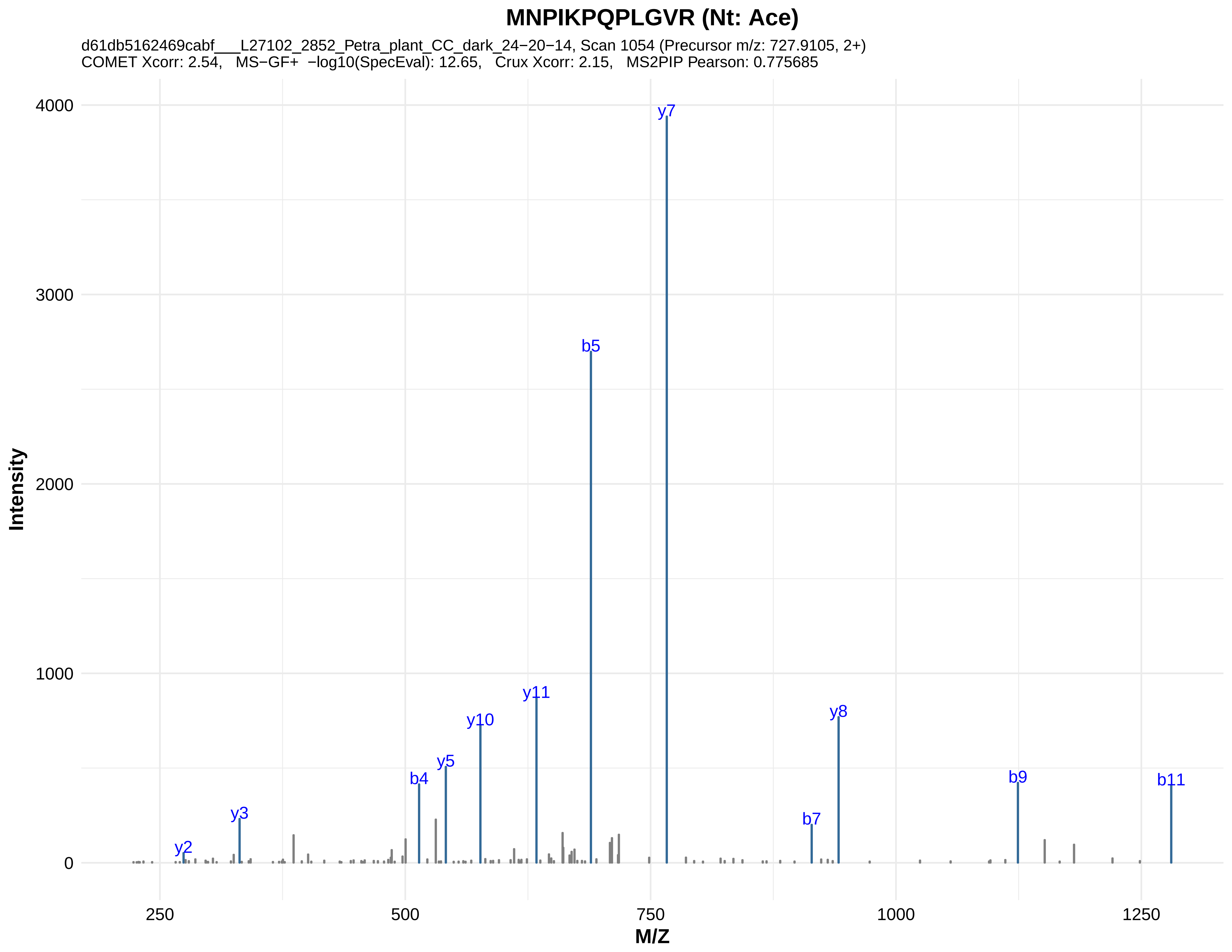
MNPIKPQPLGVR (Nt: Ace)
d61db5162469cabf___L27102_2852_Petra_plant_CC_dark_24−20−14, Scan 1054 (Precursor m/z: 727.9105, 2+)
COMET Xcorr: 2.54, MS−GF+ −log10(SpecEval): 12.65, Crux Xcorr: 2.15, MS2PIP Pearson: 0.775685
Intensity
M/Z
y7
b5
y11
y10
y8
y5
b4
b9
b11
y3
b7
y2
0
1000
2000
3000
4000
250
500
750
1000
1250

MNSISSSSMR (Nt: Ace)

d61db5162469cabf___L27058_2852_Petra_plant_CC_dark_32−28−2, Scan 56 (Precursor m/z: 587.2477, 2+)
COMET Xcorr: 2.4, MS−GF+ −log10(SpecEval): 8.56, Crux Xcorr: 2.22, MS2PIP Pearson: 0.71607864

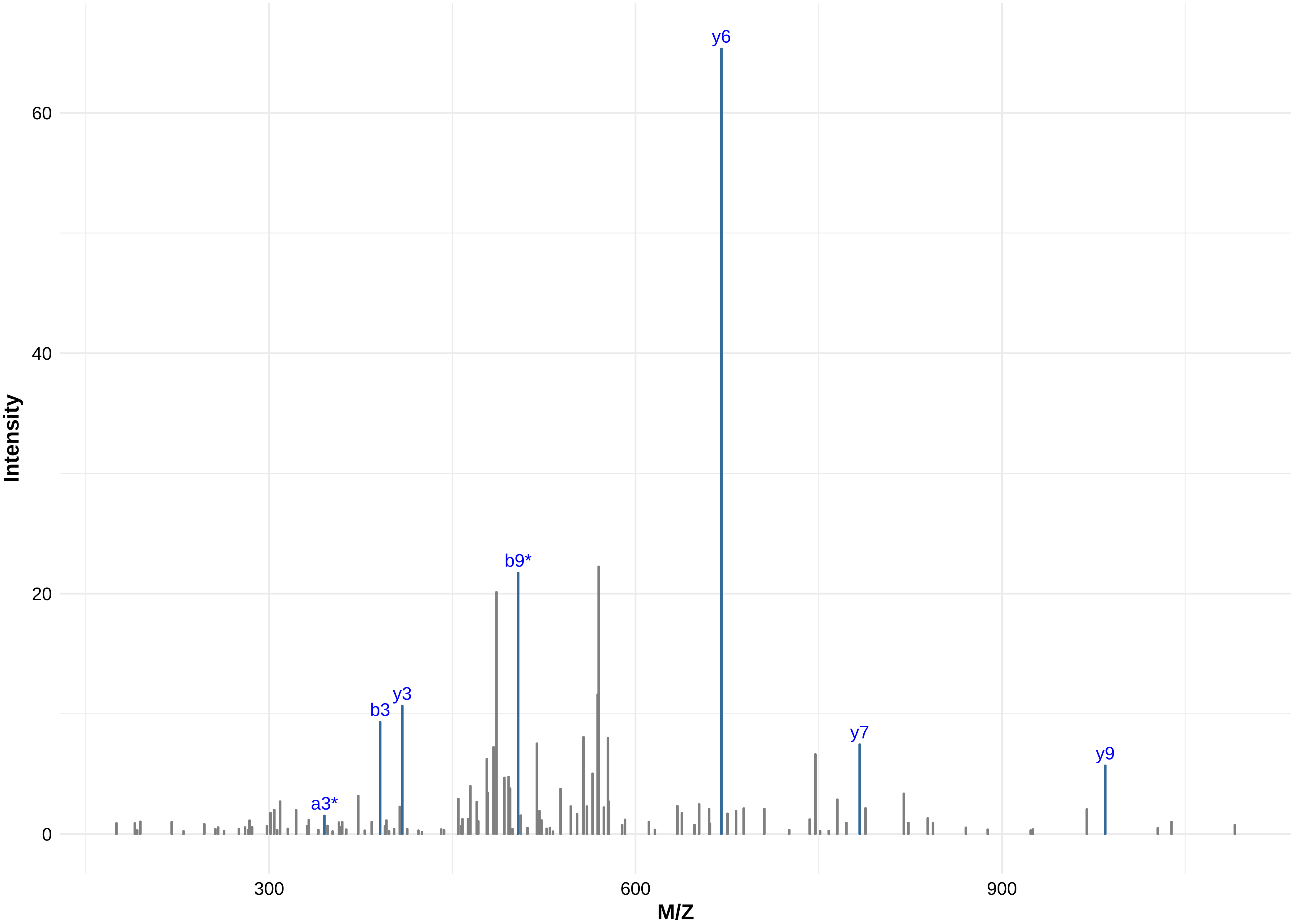

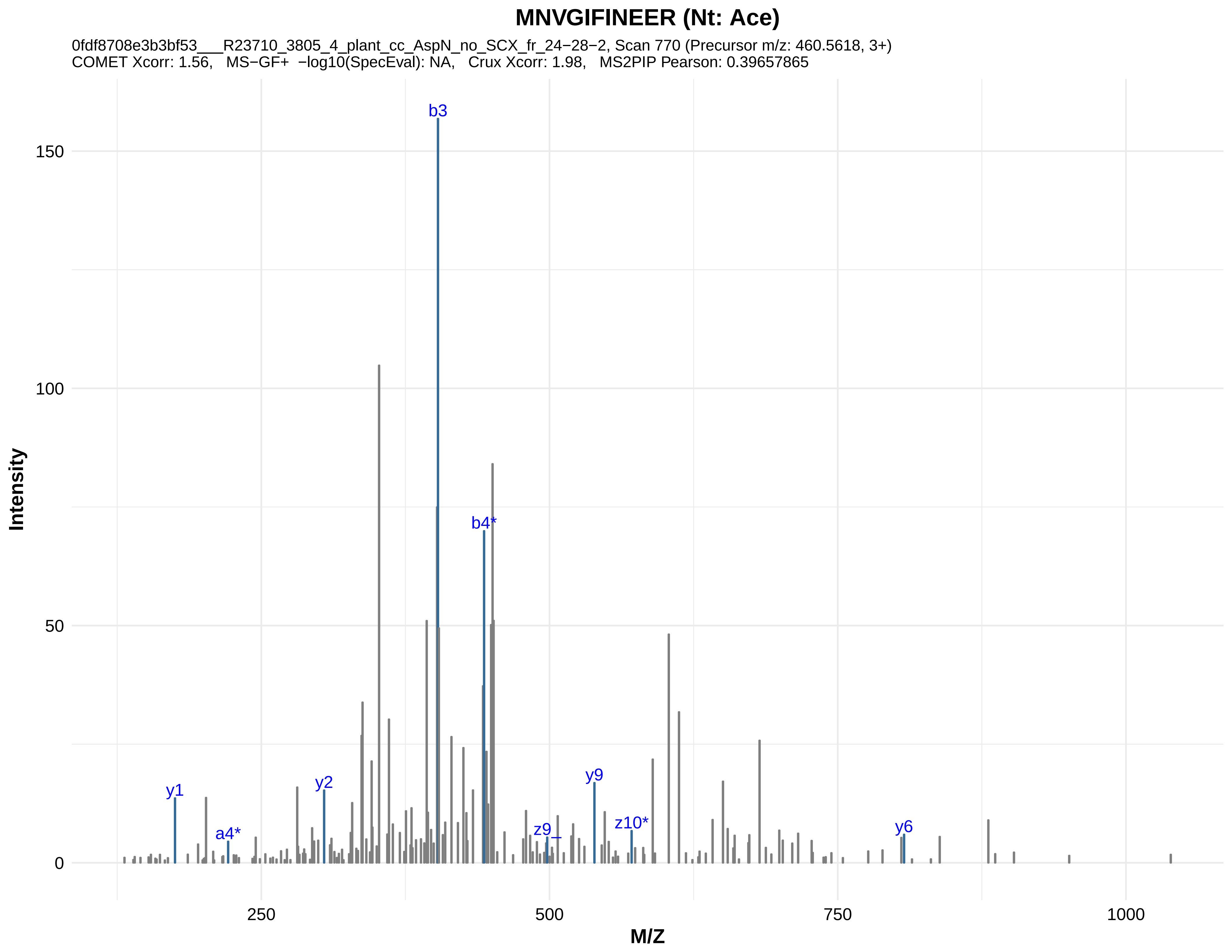
MNVGIFINEEER (Nt: Ace)
0fdf8708e3b3bf53___R23710_3805_4_plant_cc_AspN_no_SCX_fr_24−28−2, Scan 770 (Precursor m/z: 460.5618, 3+)
COMET Xcorr: 1.56,   MS−GF+ −log10(SpecEval): NA,   Crux Xcorr: 1.98,   MS2PIP Pearson: 0.39657865
b3
b4*
y1
a4*
y2
z9_
y9
z10*
y6
Intensity
150
100
50
0
250
500
750
1000
M/Z

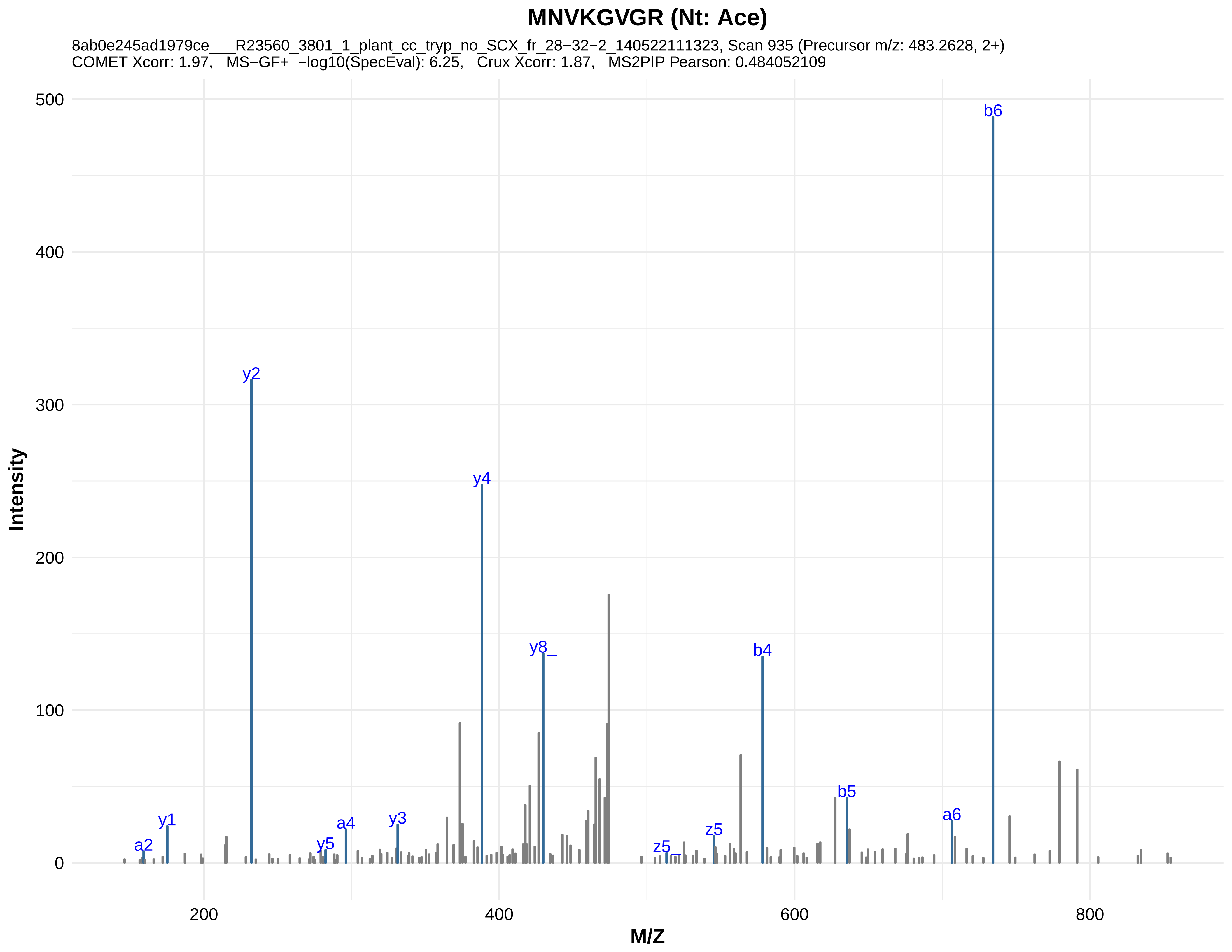
MNVKGVGR (Nt: Ace)
8ab0e245ad1979ce___R23560_3801_1_plant_cc_tryp_no_SCX_fr_28−32−2_140522111323, Scan 935 (Precursor m/z: 483.2628, 2+)
COMET Xcorr: 1.97, MS−GF+ −log10(SpecEval): 6.25, Crux Xcorr: 1.87, MS2PIP Pearson: 0.484052109
Intensity
M/Z
0
100
200
300
400
500
200
400
600
800
b6
y2
y4
y8_
b4
b5
a6
z5
z5_
y1
a2
y5
a4
y3

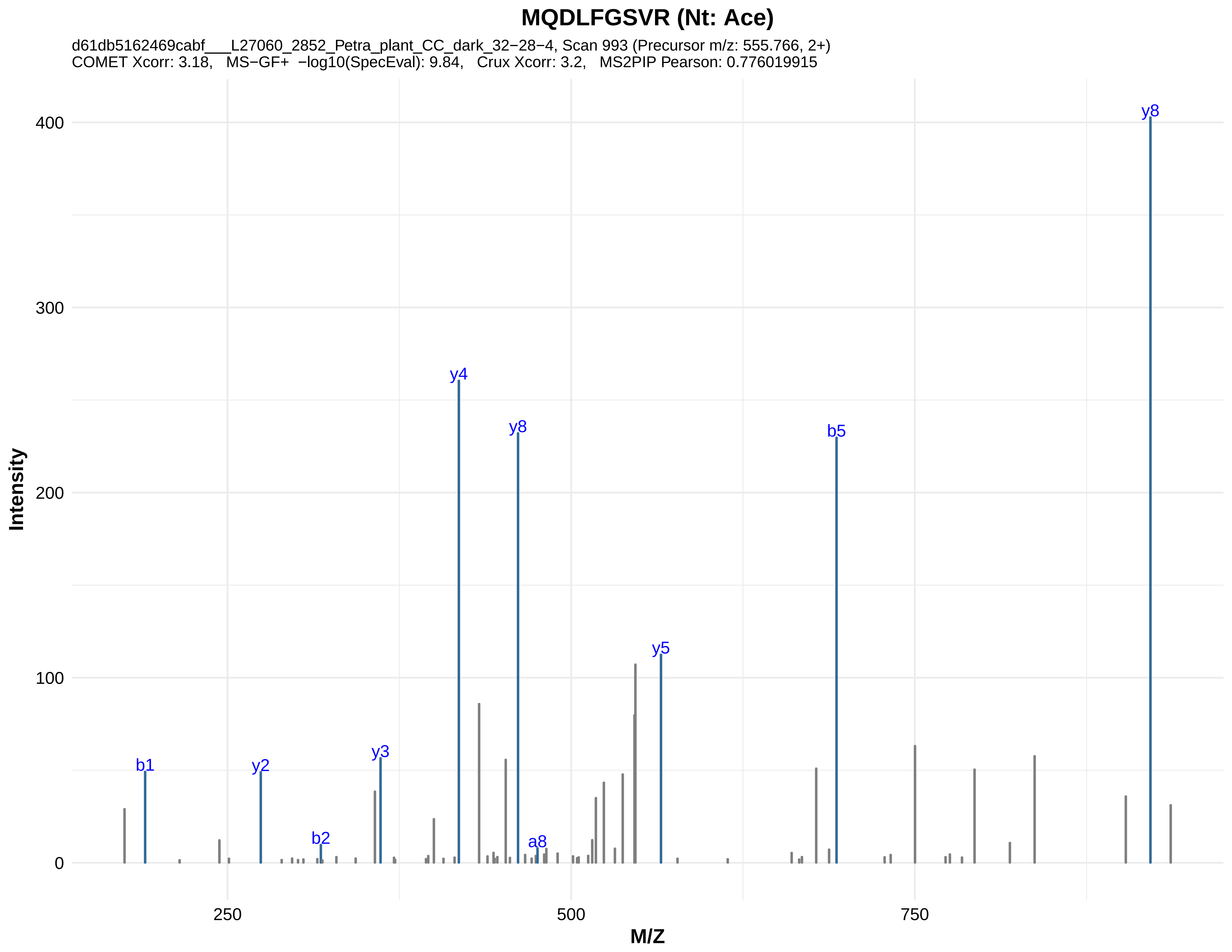
MQDLFGSVR (Nt: Ace)
d61db5162469cabf___L27060_2852_Petra_plant_CC_dark_32−28−4, Scan 993 (Precursor m/z: 555.766, 2+)
COMET Xcorr: 3.18,   MS−GF+ −log10(SpecEval): 9.84,   Crux Xcorr: 3.2,   MS2PIP Pearson: 0.776019915
Intensity
M/Z
0
100
200
300
400
250
500
750
b1
y2
b2
y3
y4
y8
a8
y5
b5
y8

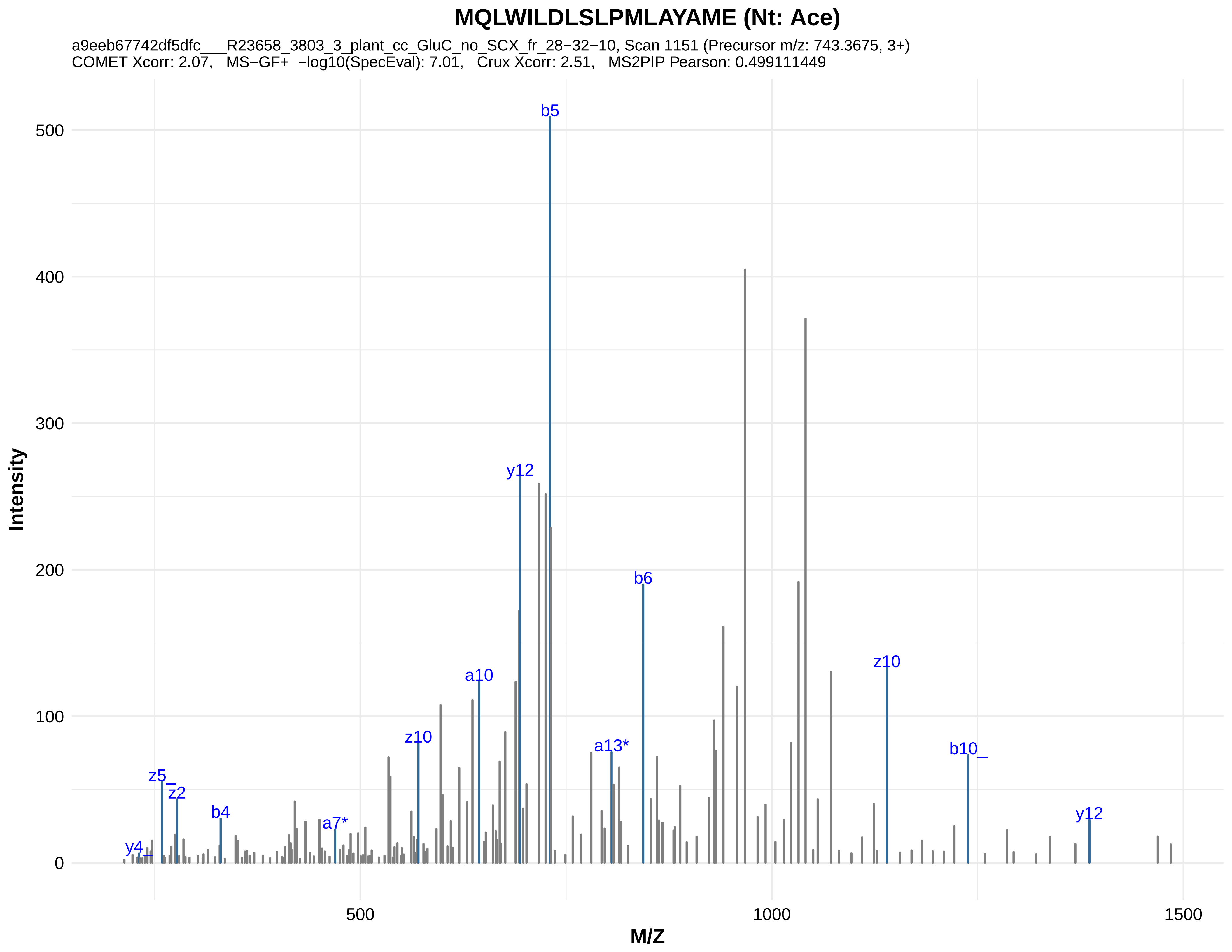
MQLWILDLSLPMLAYAME (Nt: Ace)
a9eeb67742df5dfc___R23658_3803_3_plant_cc_GluC_no_SCX_fr_28−32−10, Scan 1151 (Precursor m/z: 743.3675, 3+)
COMET Xcorr: 2.07, MS−GF+ −log10(SpecEval): 7.01, Crux Xcorr: 2.51, MS2PIP Pearson: 0.499111449
Intensity
M/Z
b5
y12
b6
a10
z10
z10
a13*
b10_
z5_
z2
b4
a7*
y4_
y12

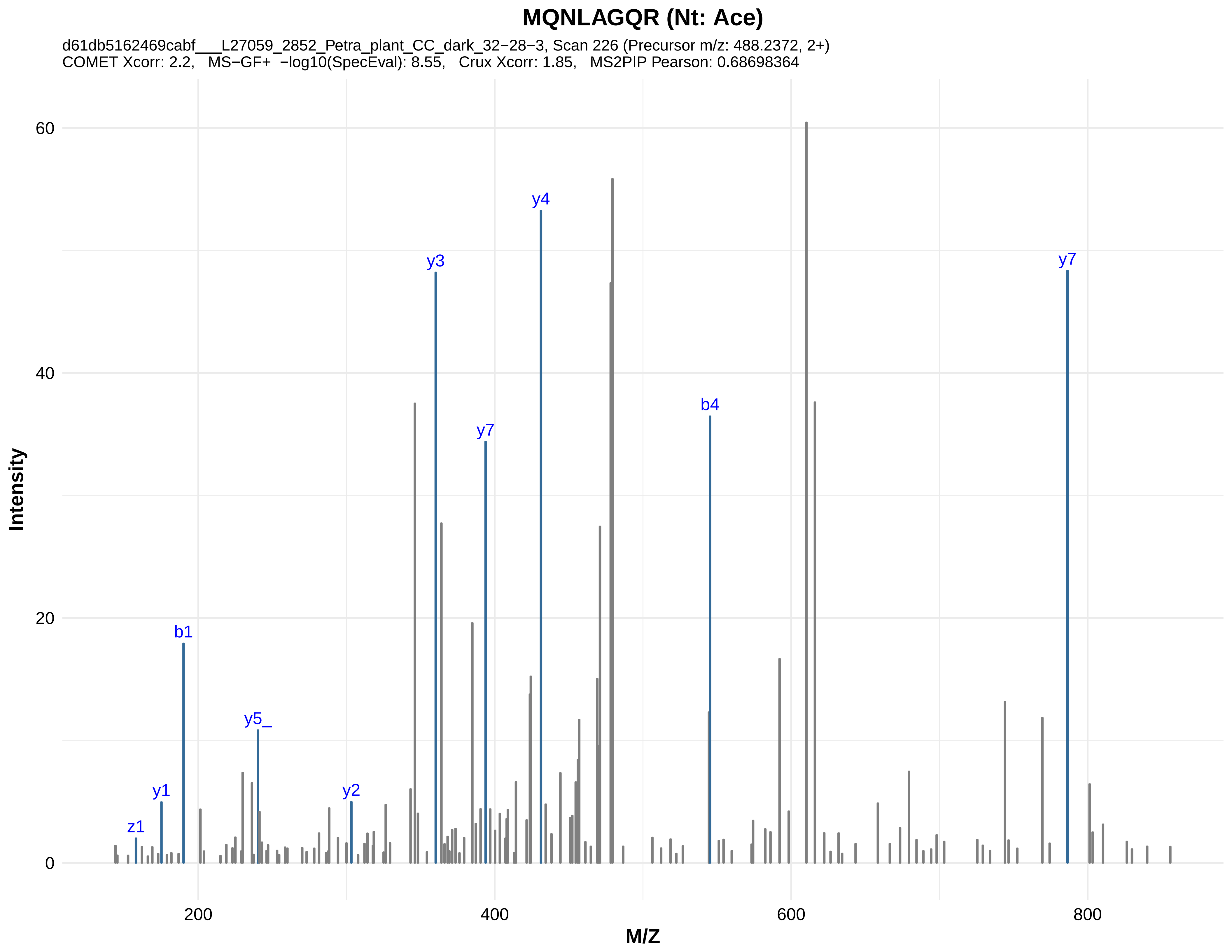
MQNLAGQR (Nt: Ace)
d61db5162469cabf___L27059_2852_Petra_plant_CC_dark_32−28−3, Scan 226 (Precursor m/z: 488.2372, 2+)
COMET Xcorr: 2.2,   MS−GF+ −log10(SpecEval): 8.55,   Crux Xcorr: 1.85,   MS2PIP Pearson: 0.68698364
Intensity
M/Z
z1
y1
b1
y5_
y2
y3
y7
y4
b4
y7

MQWRLNPLEL (Nt: Ace)

0fdf8708e3b3bf53___R23728_3805_4_plant_cc_AspN_no_SCX_fr_20−24−5, Scan 688 (Precursor m/z: 453.2341, 3+)
COMET Xcorr: 1.43, MS−GF+ −log10(SpecEval): NA, Crux Xcorr: 1.79, MS2PIP Pearson: 0.301725178

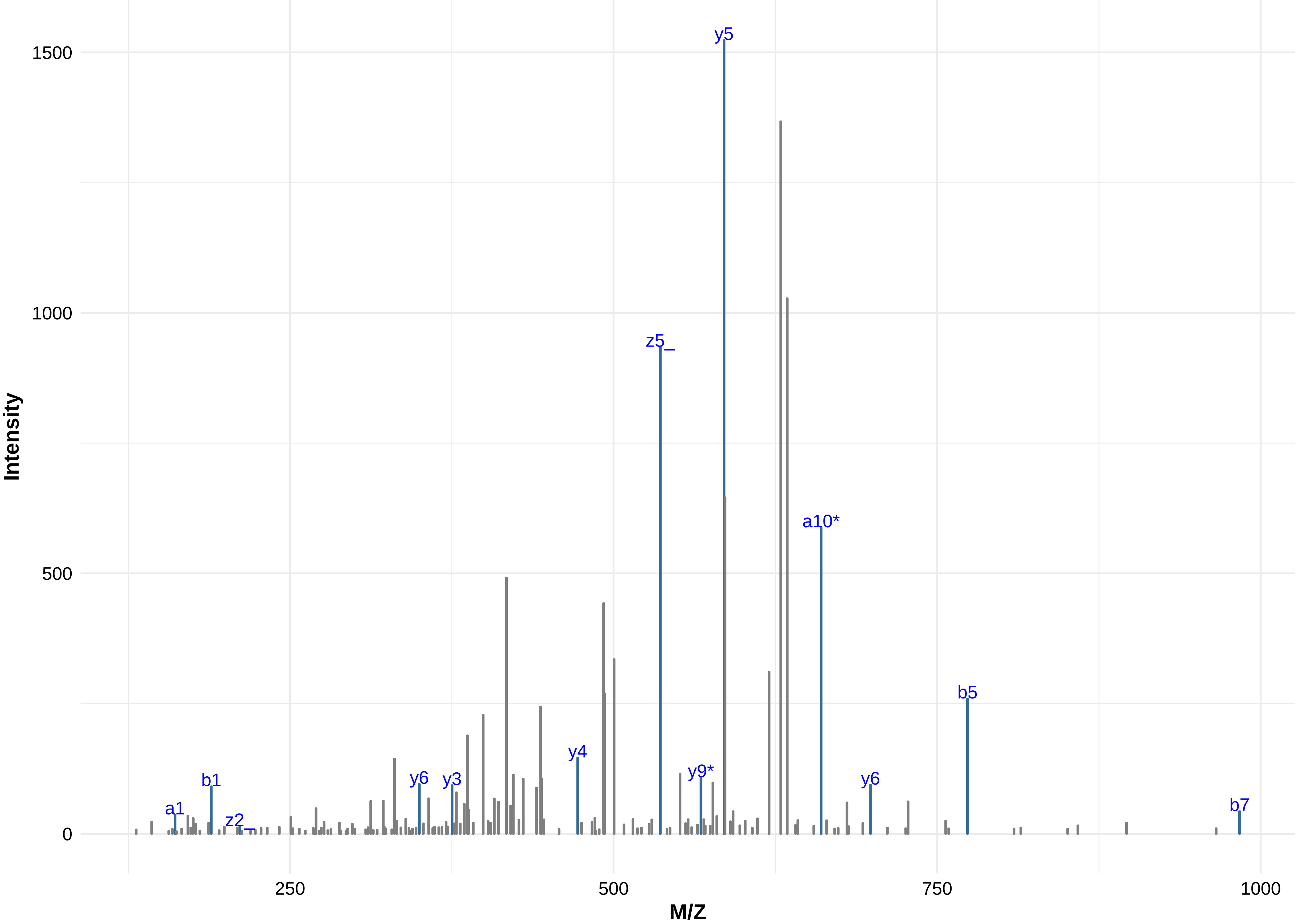

MYCEDSIWLS (Nt: Trideutero)

0fdf8708e3b3bf53___R23704_3805_4_plant_cc_AspN_no_SCX_fr_28−32−11, Scan 1464 (Precursor m/z: 683.7883, 2+)
COMET Xcorr: NA, MS−GF+ −log10(SpecEval): 8.07, Crux Xcorr: 0.95, MS2PIP Pearson: 0.415029053

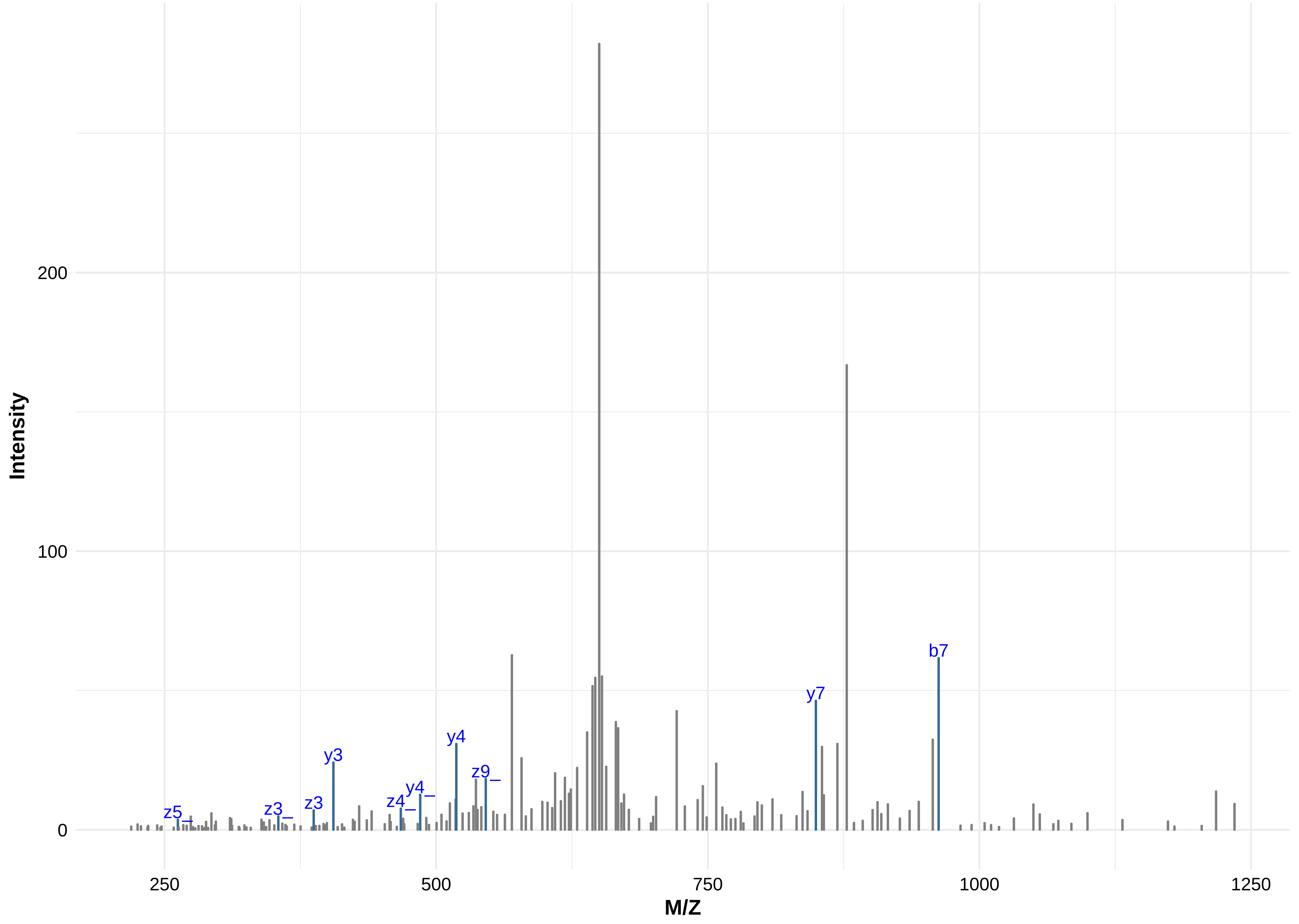

MYGENGKR (Nt: Ace)

d61db5162469cabf___L27061_2852_Petra_plant_CC_dark_32−28−5, Scan 341 (Precursor m/z: 530.2474, 2+)
COMET Xcorr: 2.09, MS−GF+ −log10(SpecEval): NA, Crux Xcorr: 1.69, MS2PIP Pearson: 0.364831935

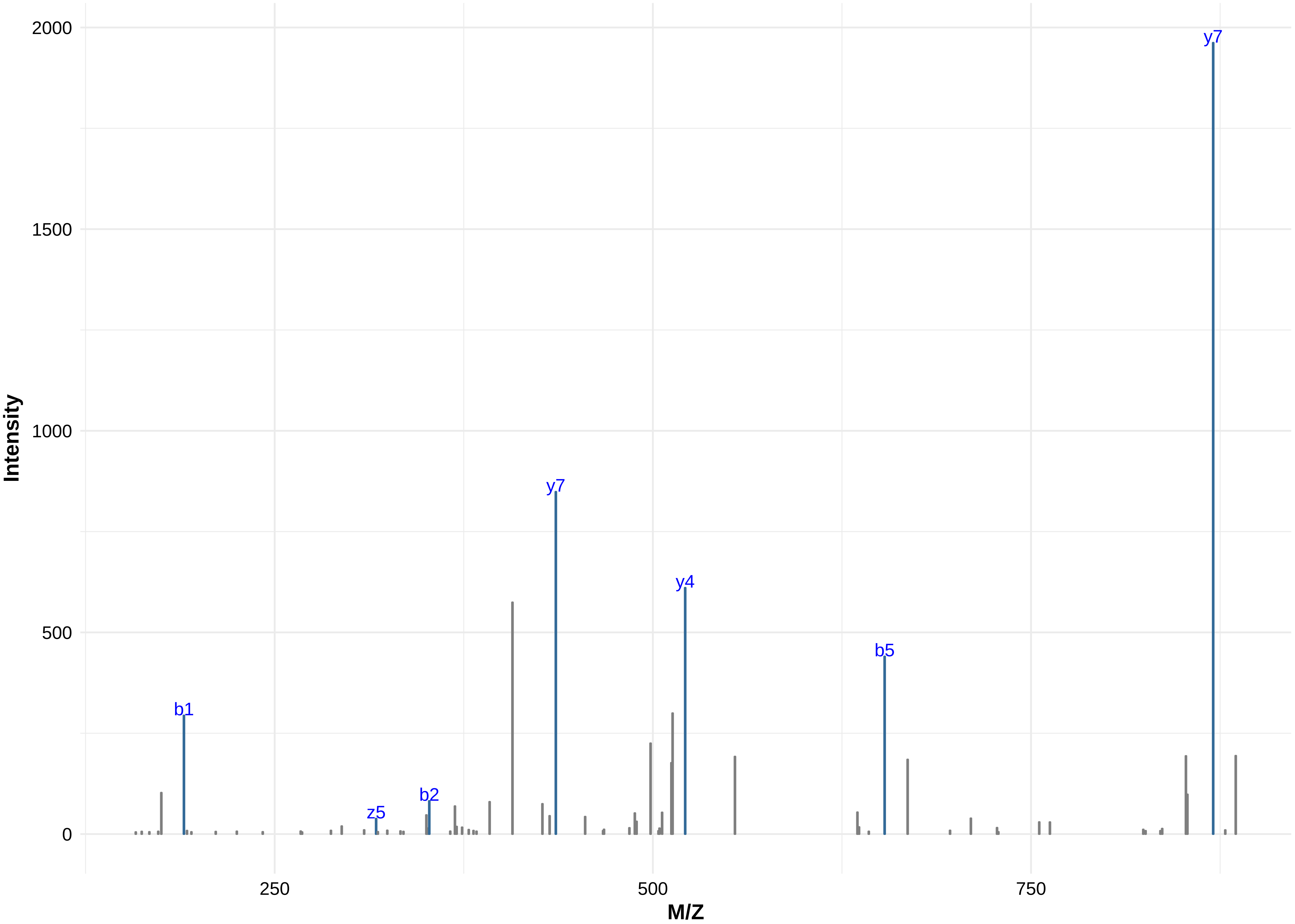

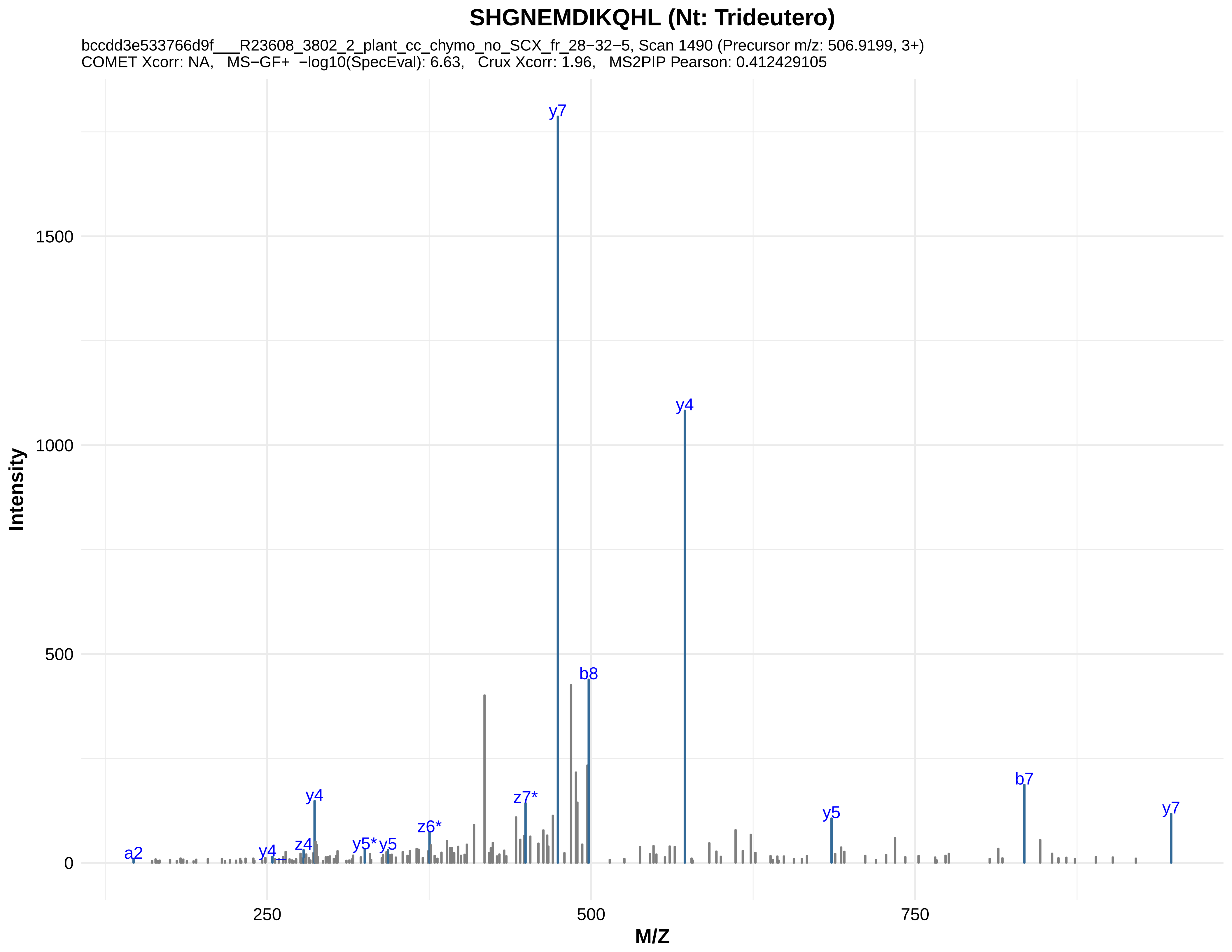

SHGNEMDIKQHL (Nt: Trideutero)
bccdd3e533766d9f___R23608_3802_2_plant_cc_chymo_no_SCX_fr_28−32−5, Scan 1490 (Precursor m/z: 506.9199, 3+)
COMET Xcorr: NA, MS−GF+ −log10(SpecEval): 6.63, Crux Xcorr: 1.96, MS2PIP Pearson: 0.412429105
Intensity
M/Z
0
500
1000
1500
250
500
750
y7
y4
b8
b7
y7
y5
z7*
z6*
y4
z4
y5*
y5
y4
a2

SMLSGKAAY (Nt: Ace)

bccdd3e533766d9f___R23621_3802_2_plant_cc_chymo_no_SCX_fr_24−28−3, Scan 1101 (Precursor m/z: 516.7547, 2+)
COMET Xcorr: 2.26, MS−GF+ −log10(SpecEval): NA, Crux Xcorr: 2.2, MS2PIP Pearson: 0.697217639

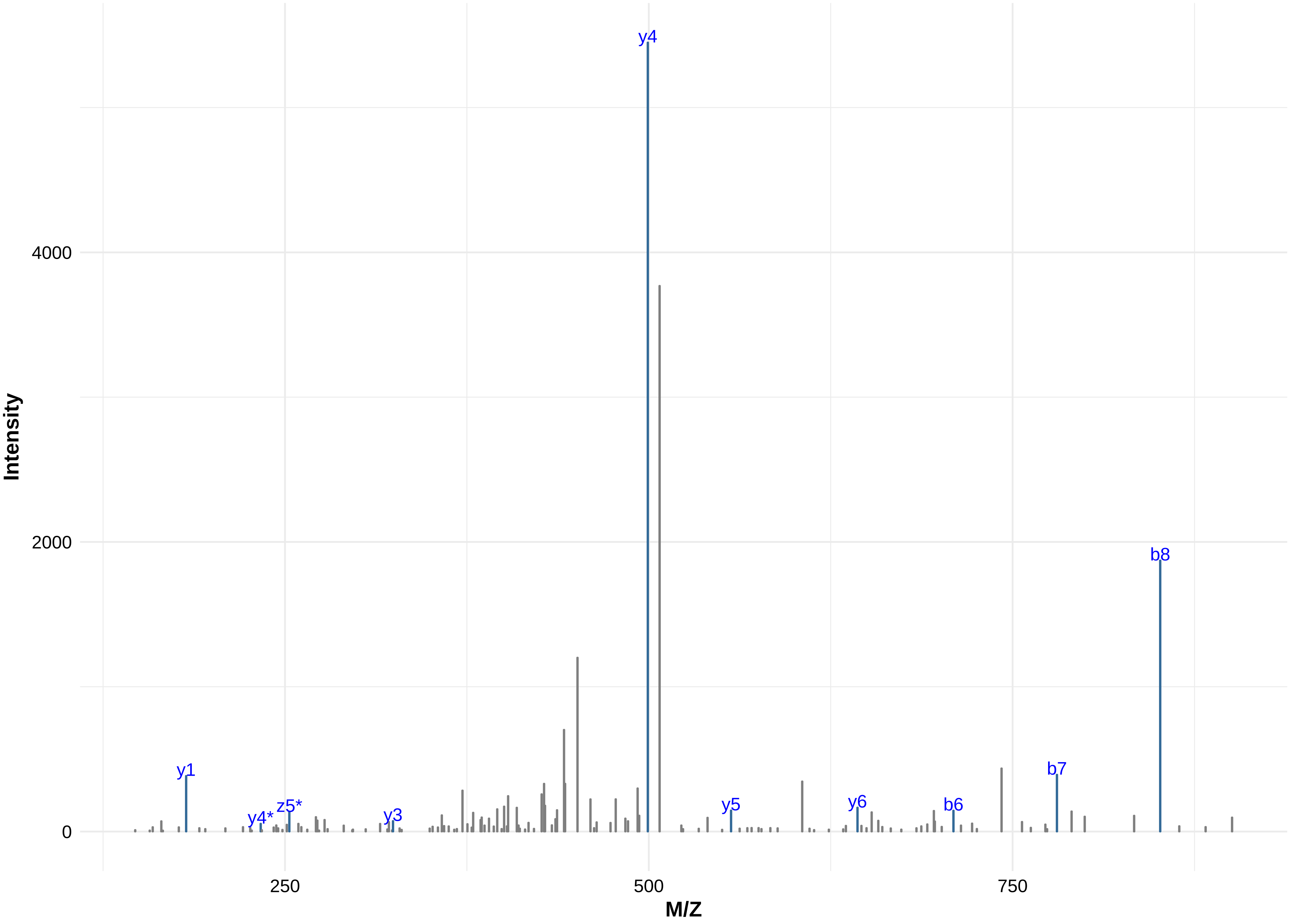

SNLSSSSSGSTGPR (Nt: Ace)

8ab0e245ad1979ce___R23566_3801_1_plant_cc_tryp_no_SCX_fr_28−32−8, Scan 505 (Precursor m/z: 683.3157, 2+)
COMET Xcorr: 2.58,   MS−GF+ −log10(SpecEval): 7.87,   Crux Xcorr: 2.62,   MS2PIP Pearson: 0.761614954

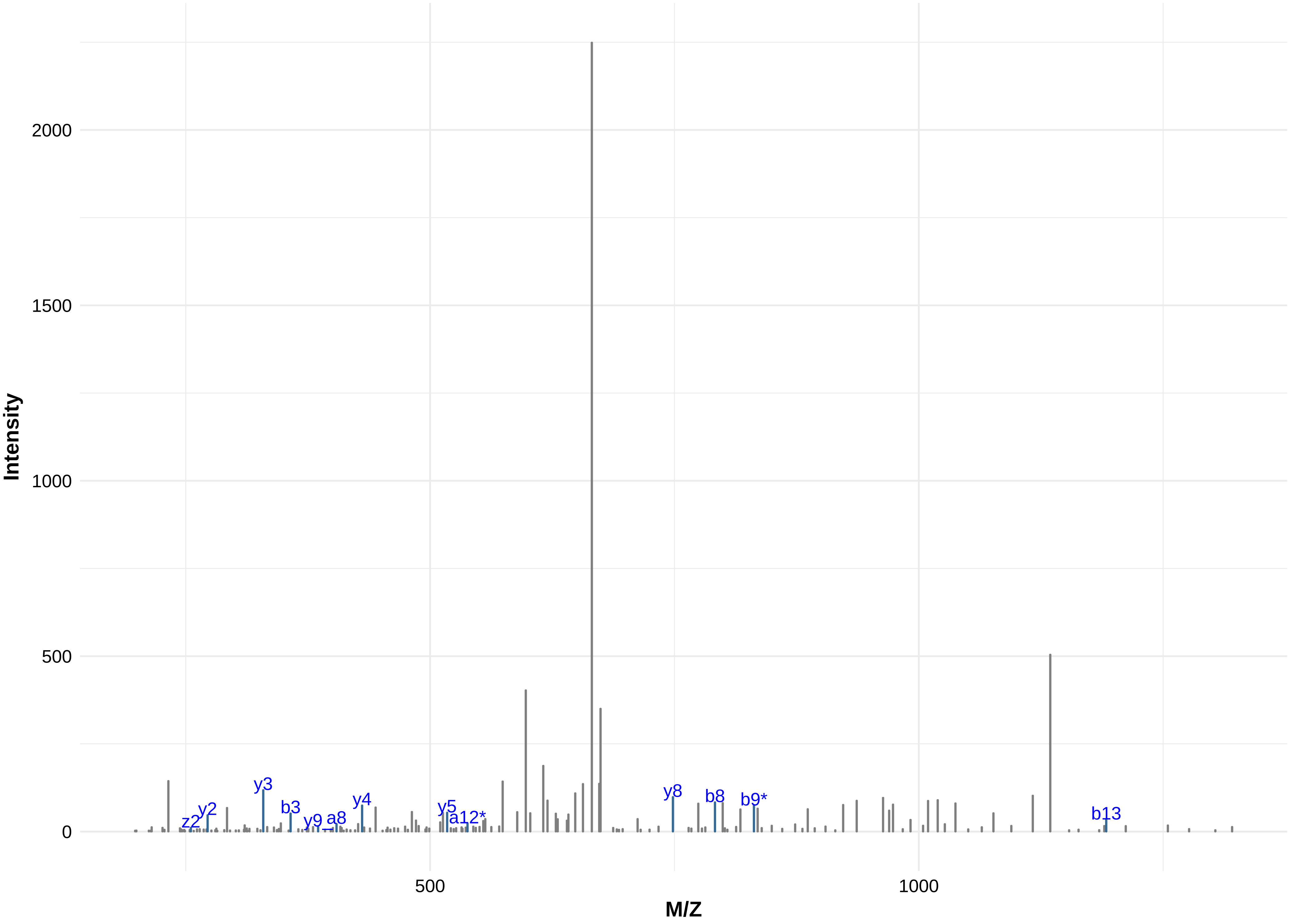

SRDDIPFTKSPGITTATSD (Nt: Trideutero)

a9eeb67742df5dfc___R23660_3803_3_plant_cc_GluC_no_SCX_fr_28−32−12, Scan 307 (Precursor m/z: 701.6908, 3+)
COMET Xcorr: 1.5, MS−GF+ −log10(SpecEval): NA, Crux Xcorr: 2.4, MS2PIP Pearson: 0.245612263

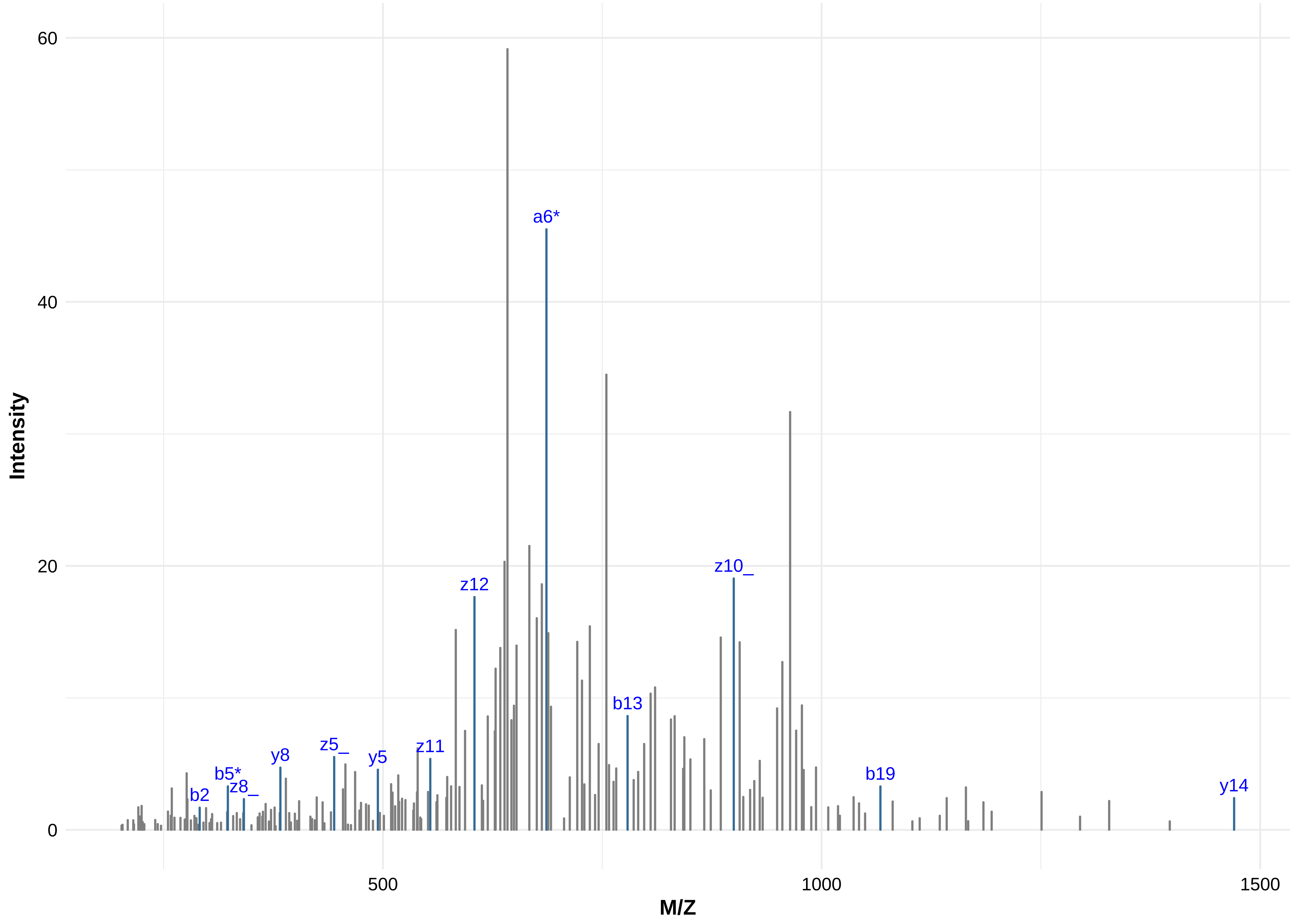

SSENDYTR (Nt: Ace)

d61db5162469cabf___L27084_2852_Petra_plant_CC_dark_28−24−12, Scan 284 (Precursor m/z: 507.2119, 2+)
COMET Xcorr: 1.61,   MS−GF+ −log10(SpecEval): 9.91,   Crux Xcorr: 1.78,   MS2PIP Pearson: 0.693909069

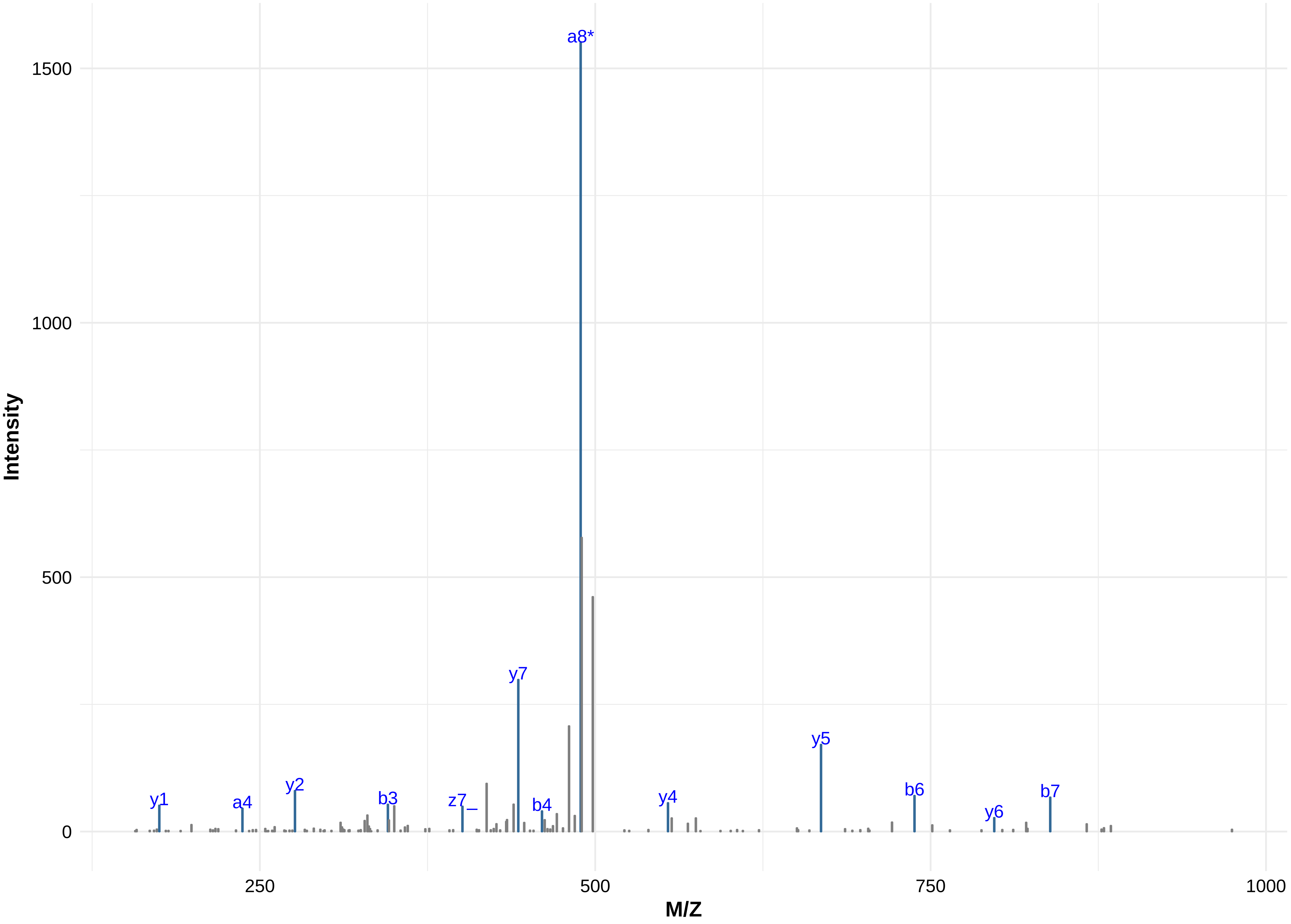

SSVPRNAPDANVVM (Nt: Ace)

bccdd3e533766d9f___R23642_3802_2_plant_cc_chymo_no_SCX_fr_20−24−9_140717020336, Scan 559 (Precursor m/z: 505.581, 3+)
COMET Xcorr: 1.99, MS−GF+ −log10(SpecEval): NA, Crux Xcorr: 2.17, MS2PIP Pearson: 0.514785486

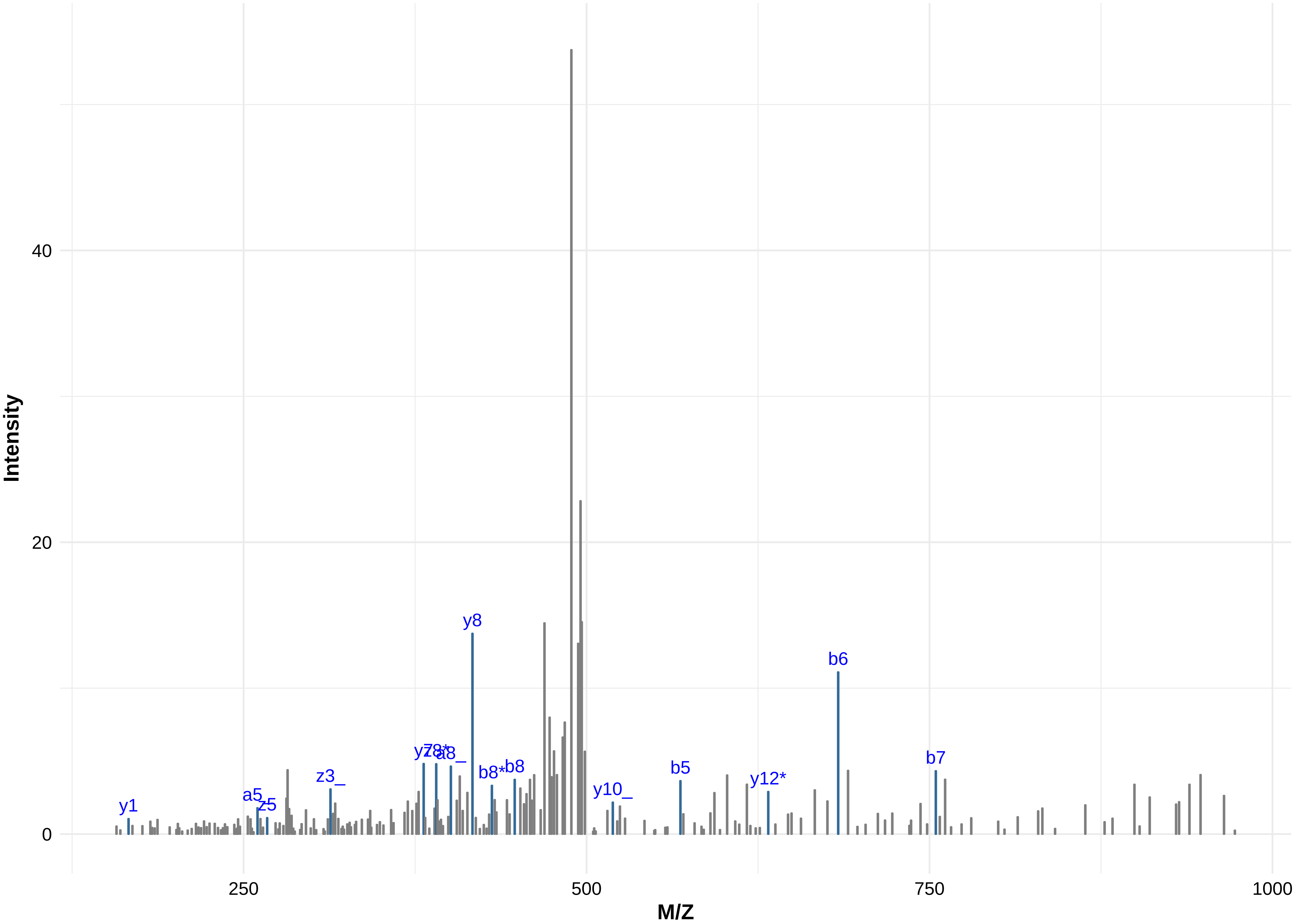

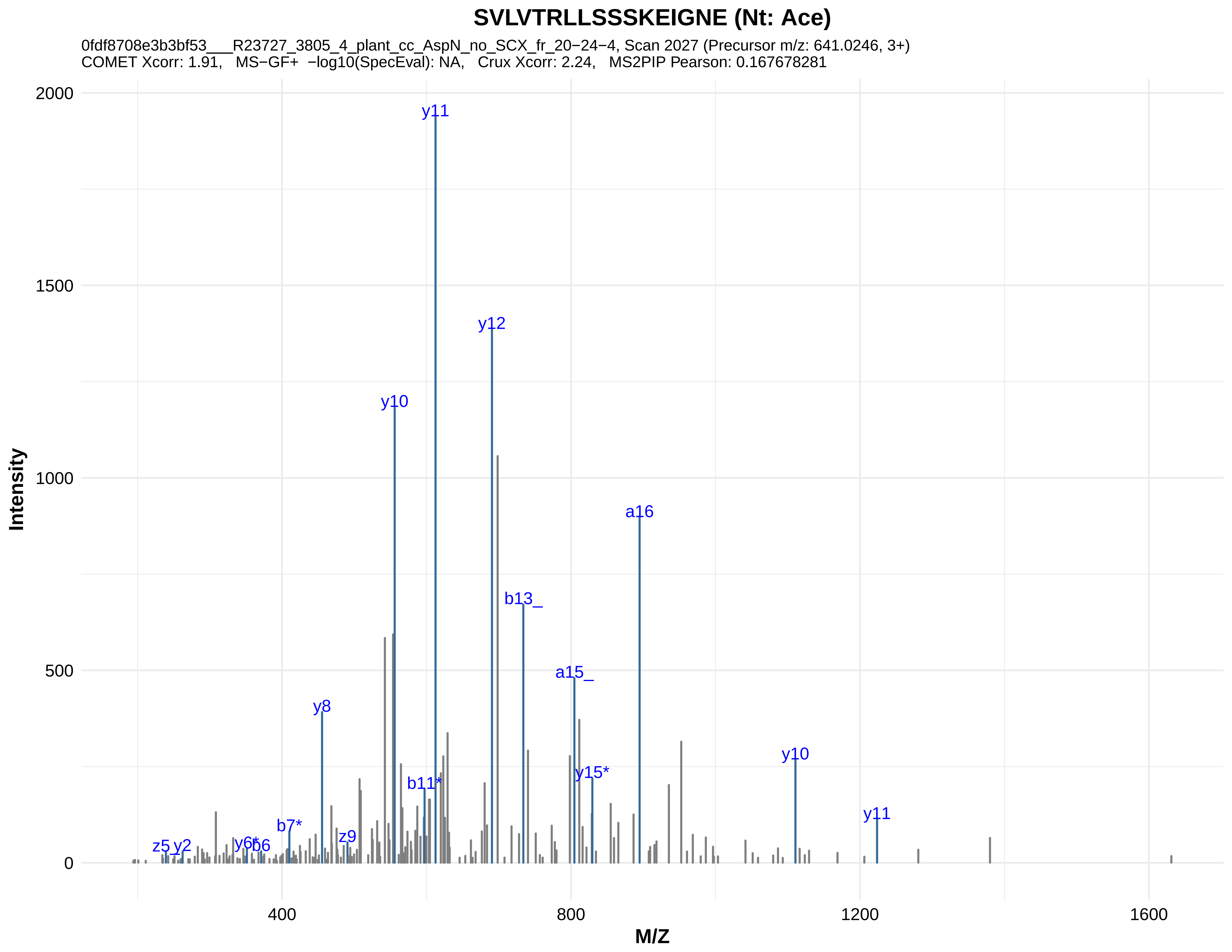

SVLVTRLLSSSKEIGNE (Nt: Ace)
0fdf8708e3b3bf53___R23727_3805_4_plant_cc_AspN_no_SCX_fr_20−24−4, Scan 2027 (Precursor m/z: 641.0246, 3+)
COMET Xcorr: 1.91, MS−GF+ −log10(SpecEval): NA, Crux Xcorr: 2.24, MS2PIP Pearson: 0.167678281
Intensity
M/Z
y11
y12
y10
b13_
a16
a15_
y8
y10
y15*
b11*
y11
b7*
z9
z5
y2
y6*
b6

TVYRIESRPREEHY (Nt: Ace)

bccdd3e533766d9f___R23640_3802_2_plant_cc_chymo_no_SCX_fr_20−24−7, Scan 683 (Precursor m/z: 626.3134, 3+)
COMET Xcorr: 1.96, MS−GF+ −log10(SpecEval): NA, Crux Xcorr: 1.4, MS2PIP Pearson: 0.045743886

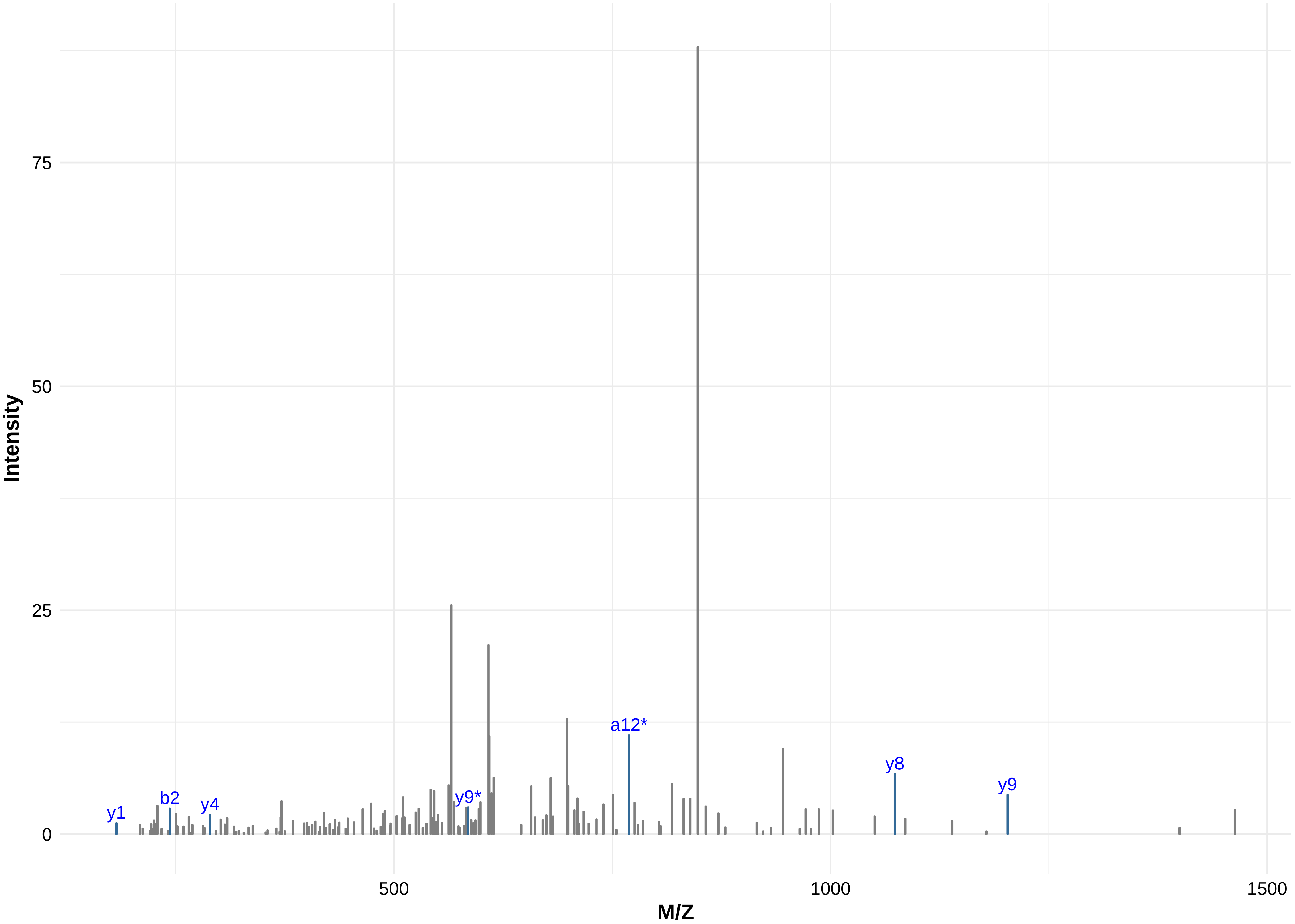

VRQQRASKVHE (Nt: Trideutero)

0fdf8708e3b3bf53___R23727_3805_4_plant_cc_AspN_no_SCX_fr_20−24−4, Scan 275 (Precursor m/z: 477.9419, 3+)
COMET Xcorr: 1.91, MS−GF+ −log10(SpecEval): 6.19, Crux Xcorr: 1.97, MS2PIP Pearson: 0.354427446

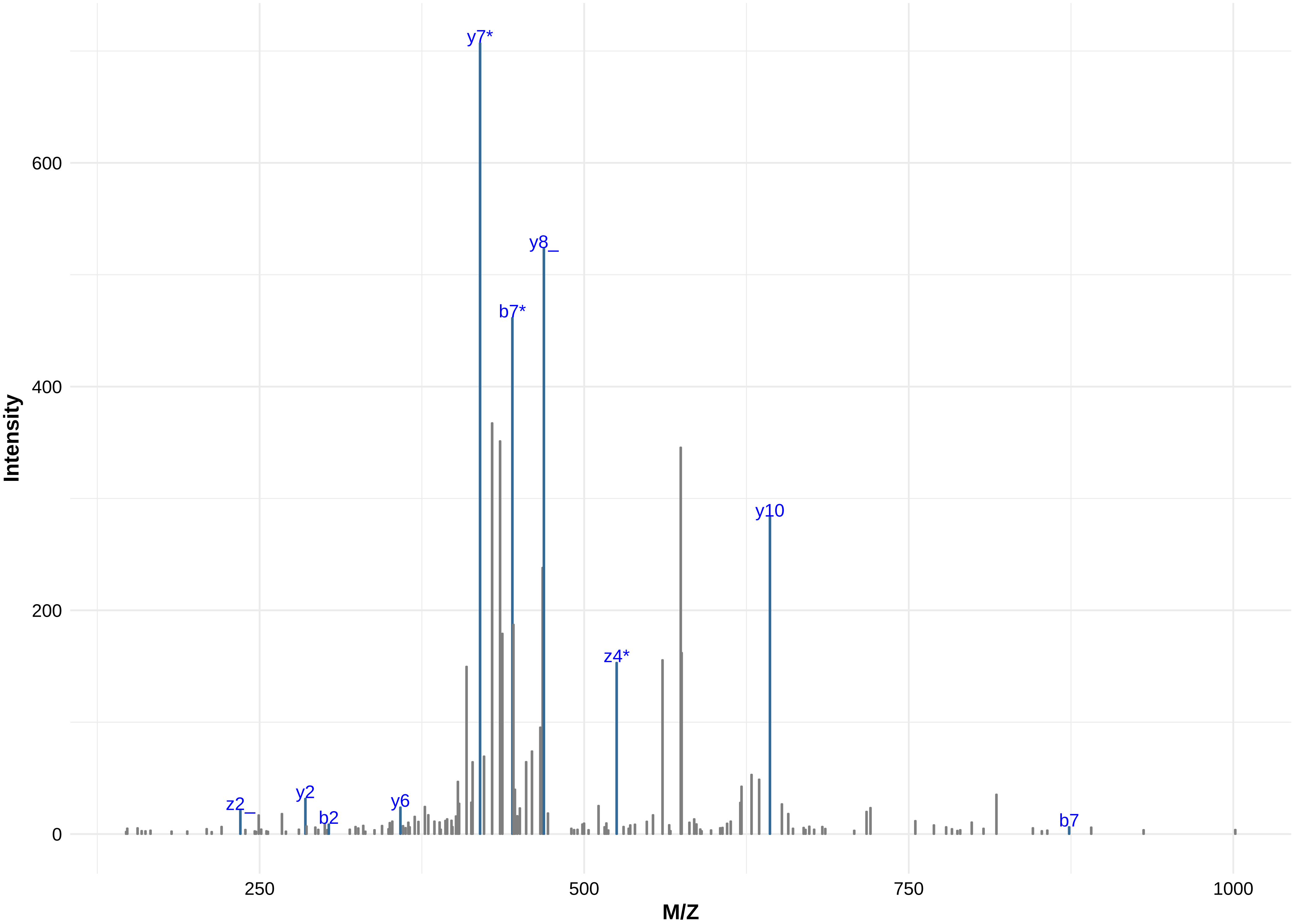

Low-confidence PSMs

No TIS meta-data available (see Supplemental Dataset 3)

and bad MS2PIP correlation (see Supplemental Dataset 1)

AFYTNKPNKLQR (Nt: Trideutero)

8ab0e245ad1979ce___R23570_3801_1_plant_cc_tryp_no_SCX_fr_28−32−12, Scan 560 (Precursor m/z: 540.9757, 3+)
COMET Xcorr: 1.6,   MS−GF+ −log10(SpecEval): NA,   Crux Xcorr: 2.06,   MS2PIP Pearson: 0.416666335

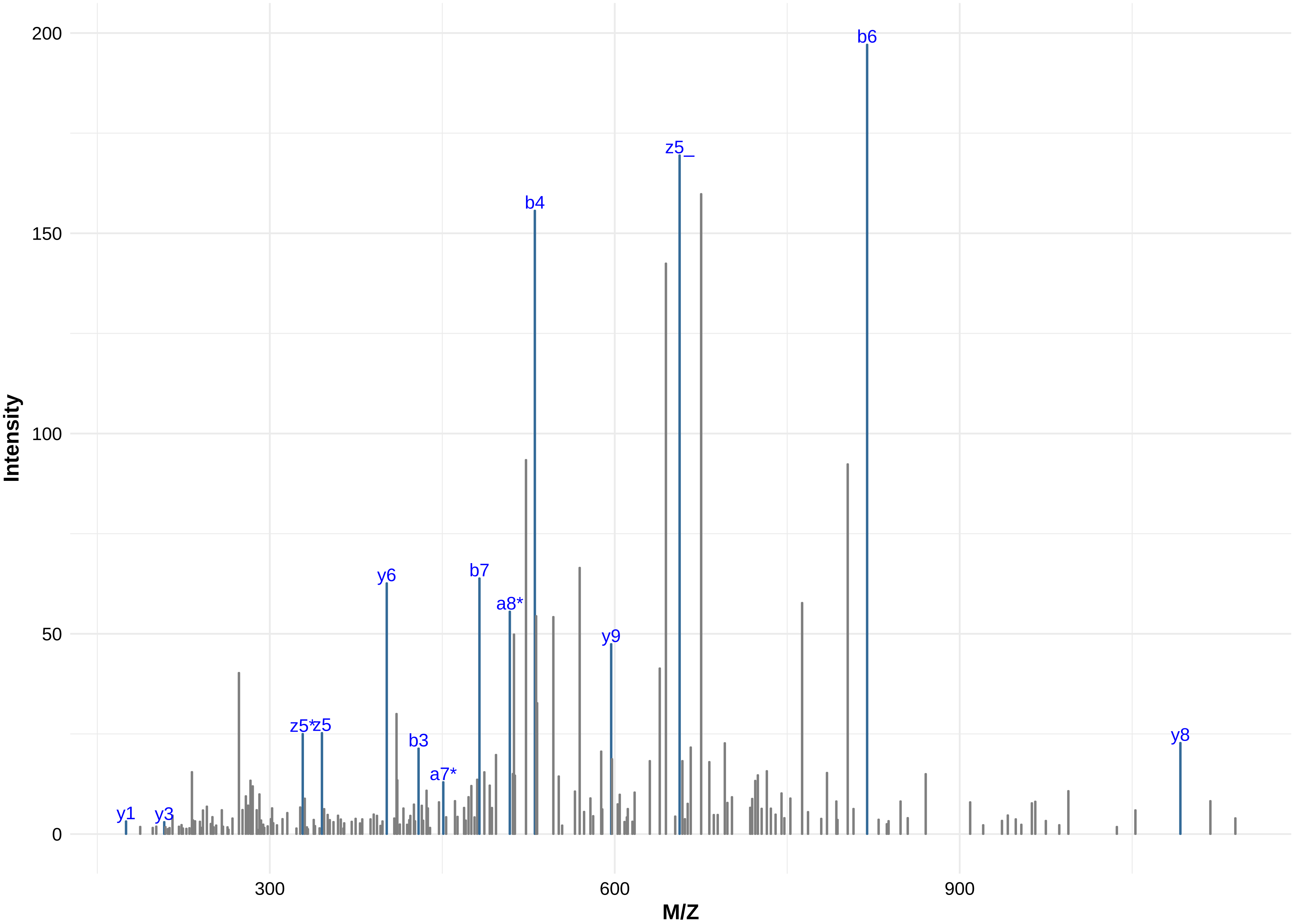

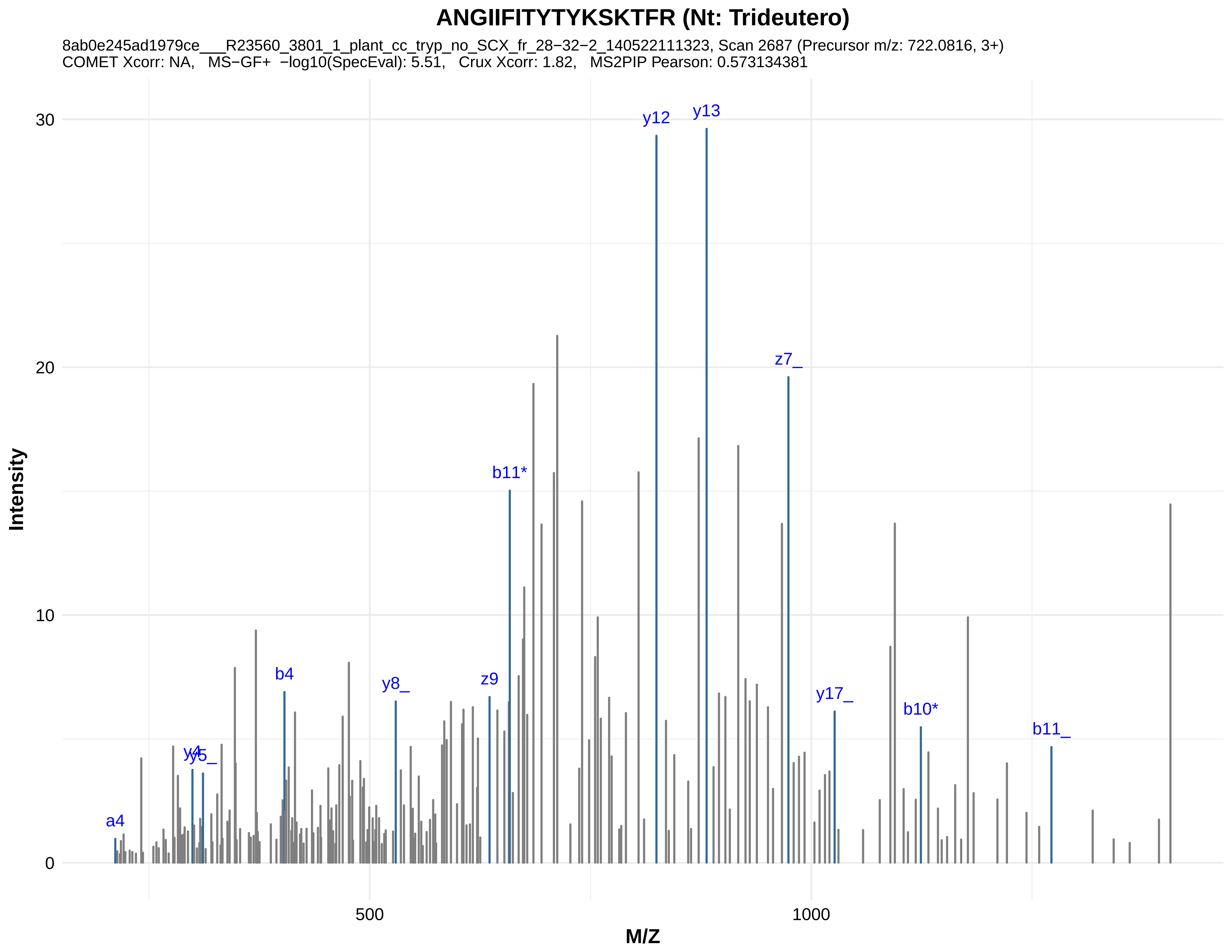
ANGIIFITYTYKSKTFR (Nt: Trideutero)
8ab0e245ad1979ce___R23560_3801_1_plant_cc_tryp_no_SCX_fr_28−32−2_140522111323, Scan 2687 (Precursor m/z: 722.0816, 3+)
COMET Xcorr: NA, MS−GF+ −log10(SpecEval): 5.51, Crux Xcorr: 1.82, MS2PIP Pearson: 0.573134381
y12
y13
z7_
b11*
b4
y8_
z9
y17_
b10*
b11_
y4
y5_
a4
Intensity
M/Z
30
20
10
0
500
1000

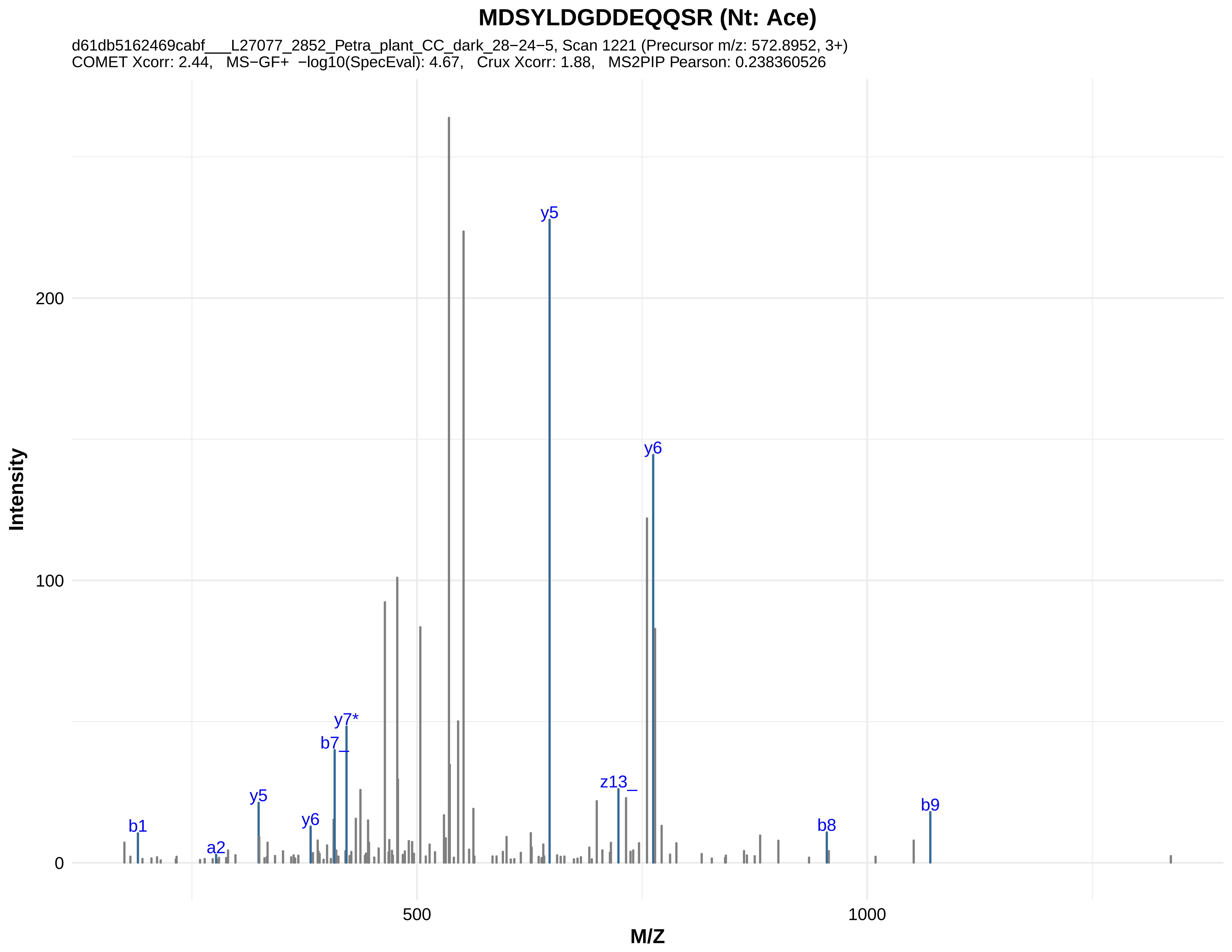

MDSYLDGDDEQQSR (Nt: Ace)
d61db5162469cabf___L27077_2852_Petra_plant_CC_dark_28−24−5, Scan 1221 (Precursor m/z: 572.8952, 3+)
COMET Xcorr: 2.44, MS−GF+ −log10(SpecEval): 4.67, Crux Xcorr: 1.88, MS2PIP Pearson: 0.238360526
Intensity
M/Z
0
100
200
500
1000
b1
a2
y5
y6
b7_
y7*
y5
z13_
y6
b8
b9

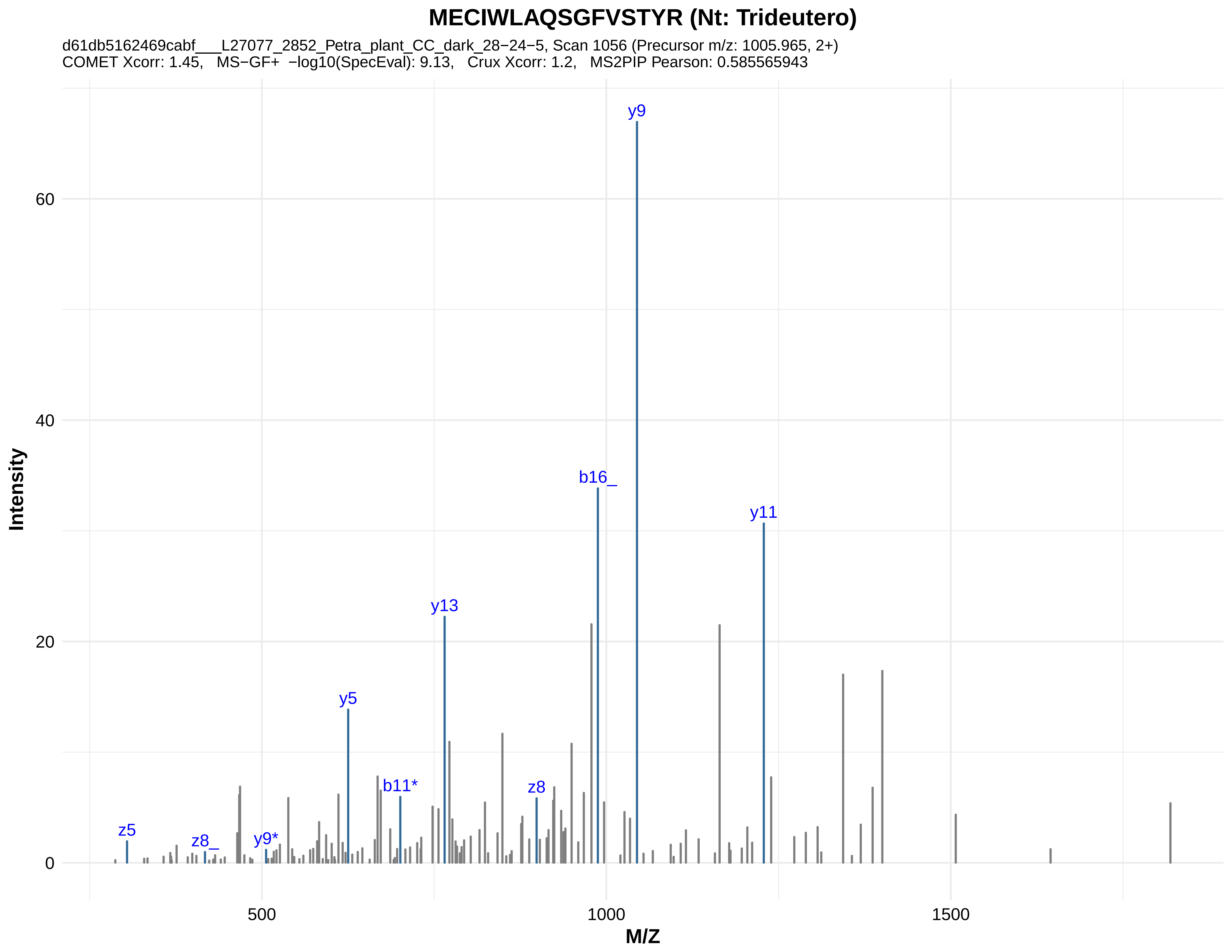

MECIWLAQSGFVSTYR (Nt: Trideutero)
d61db5162469cabf___L27077_2852_Petra_plant_CC_dark_28−24−5, Scan 1056 (Precursor m/z: 1005.965, 2+)
COMET Xcorr: 1.45, MS−GF+ −log10(SpecEval): 9.13, Crux Xcorr: 1.2, MS2PIP Pearson: 0.585565943
Intensity
M/Z
0
20
40
60
500
1000
1500
y9
b16_
y11
y13
y5
b11*
z8
z5
z8_
y9*

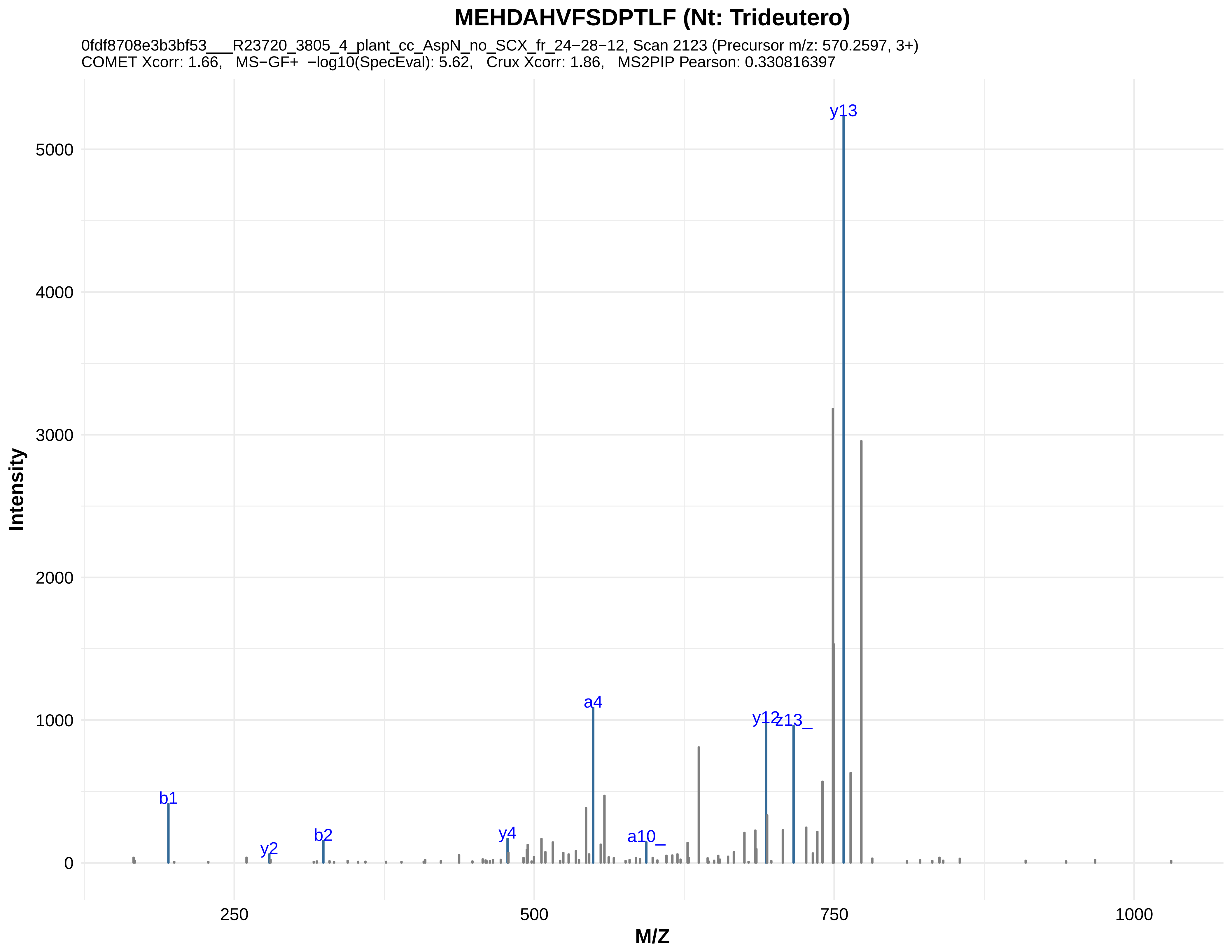
MEHDAHVFSDPTLF (Nt: Trideutero)
0fdf8708e3b3bf53___R23720_3805_4_plant_cc_AspN_no_SCX_fr_24−28−12, Scan 2123 (Precursor m/z: 570.2597, 3+)
COMET Xcorr: 1.66, MS−GF+ −log10(SpecEval): 5.62, Crux Xcorr: 1.86, MS2PIP Pearson: 0.330816397
y13
a4
y12
z13_
b1
y2
b2
y4
a10_
Intensity
M/Z
0
1000
2000
3000
4000
5000
250
500
750
1000

MEIKCSSYPENYLVGYLLKR (Nt: Ace)

d61db5162469cabf___L27061_2852_Petra_plant_CC_dark_32−28−5, Scan 1630 (Precursor m/z: 872.4447, 3+)
COMET Xcorr: 2.04, MS−GF+ −log10(SpecEval): NA, Crux Xcorr: 1.65, MS2PIP Pearson: 0.19341526

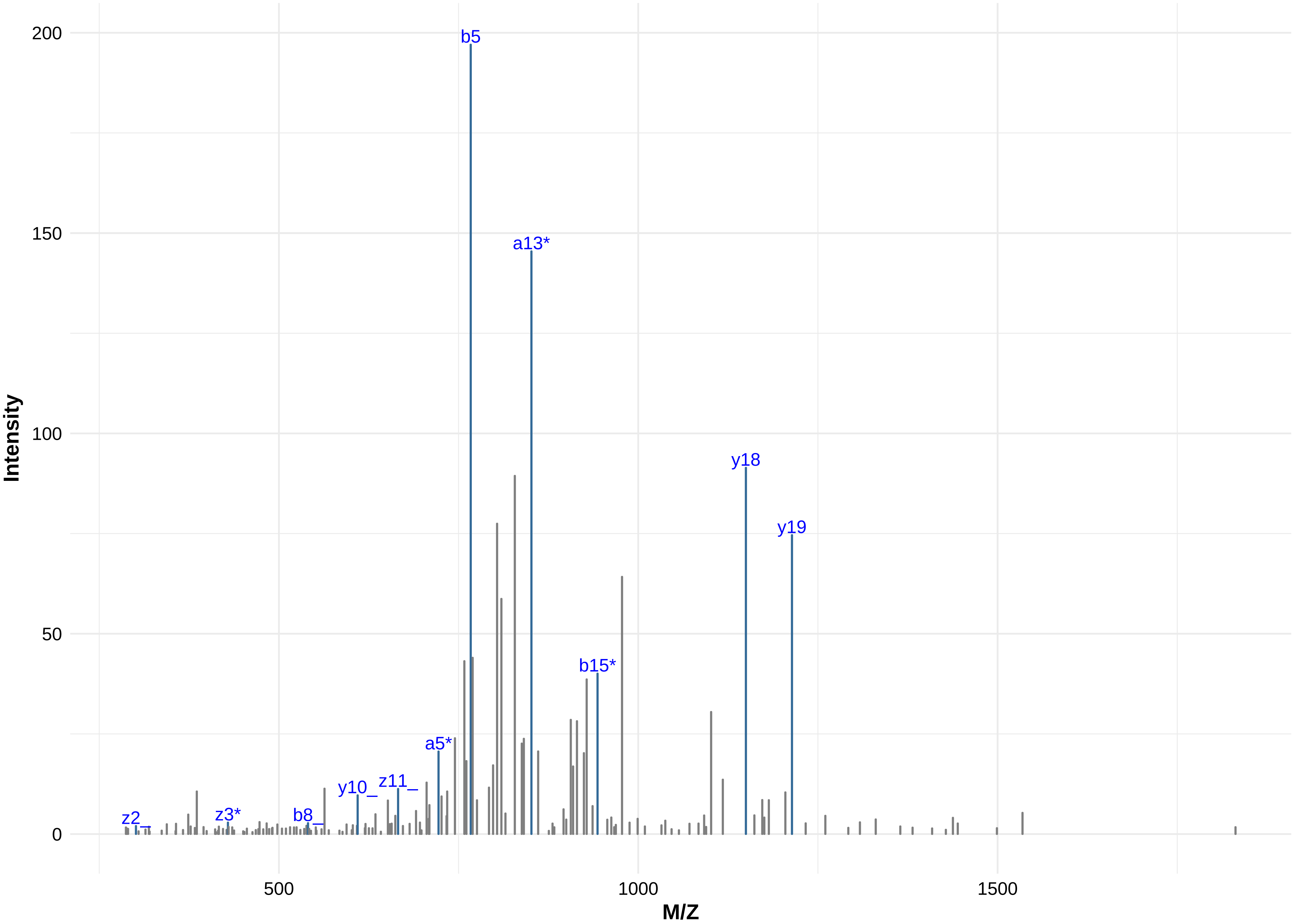

MFFFSSLPILYR (Nt: Ace)

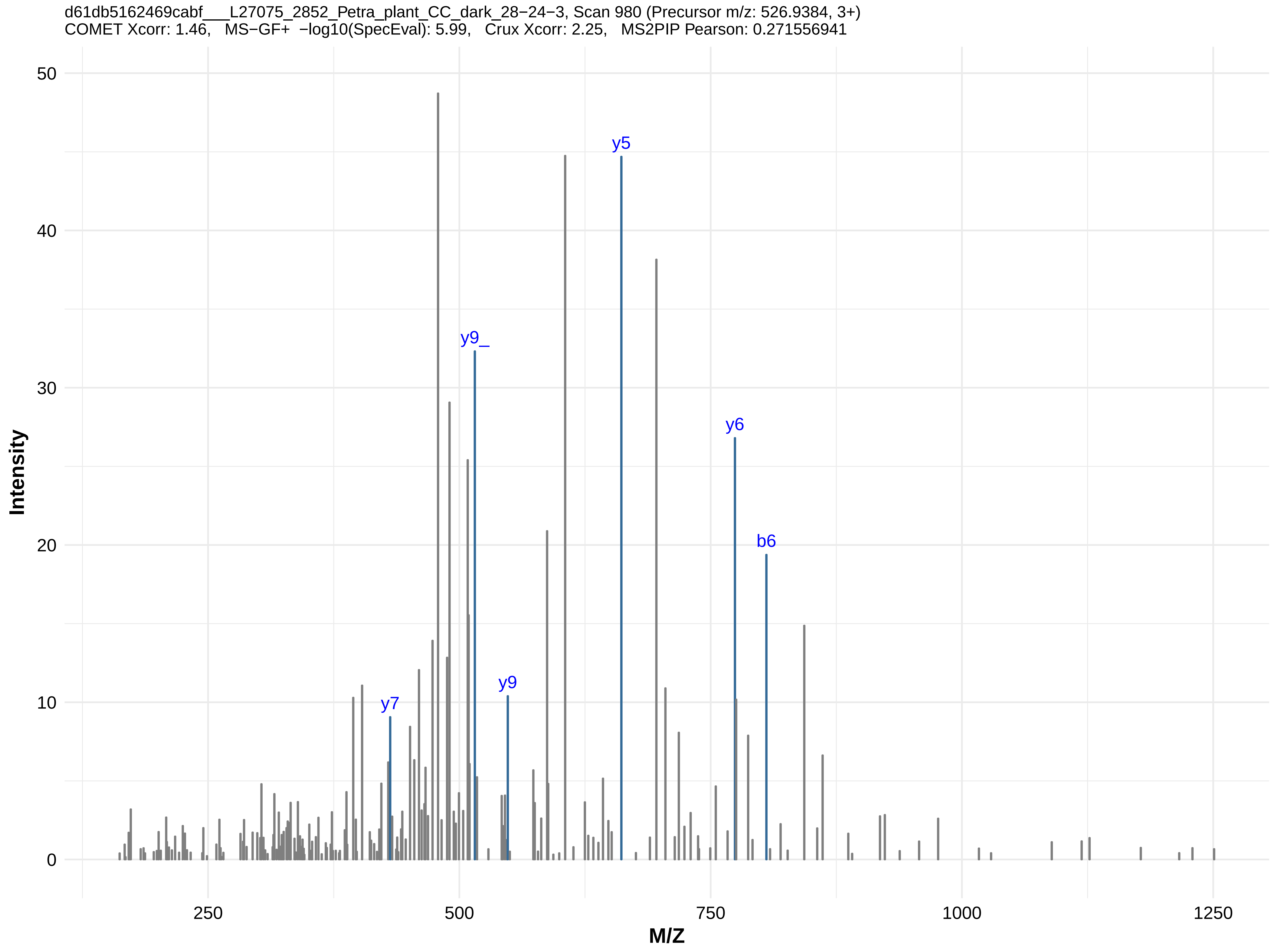

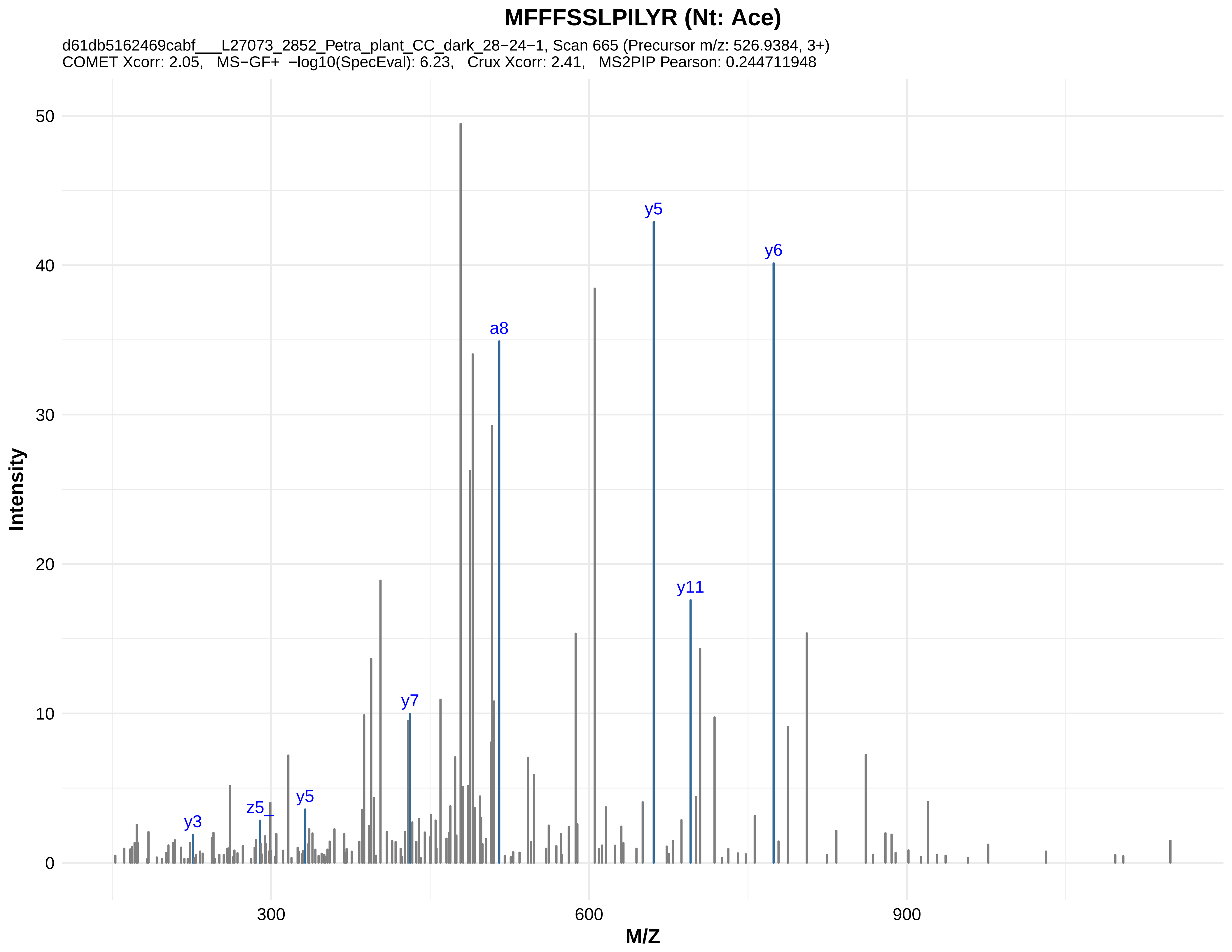
MFFFSSLPILYR (Nt: Ace)
d61db5162469cabf___L27073_2852_Petra_plant_CC_dark_28−24−1, Scan 665 (Precursor m/z: 526.9384, 3+)
COMET Xcorr: 2.05, MS−GF+ −log10(SpecEval): 6.23, Crux Xcorr: 2.41, MS2PIP Pearson: 0.244711948
Intensity
M/Z
0
10
20
30
40
50
300
600
900
y3
z5_
y5
y7
a8
y5
y11
y6

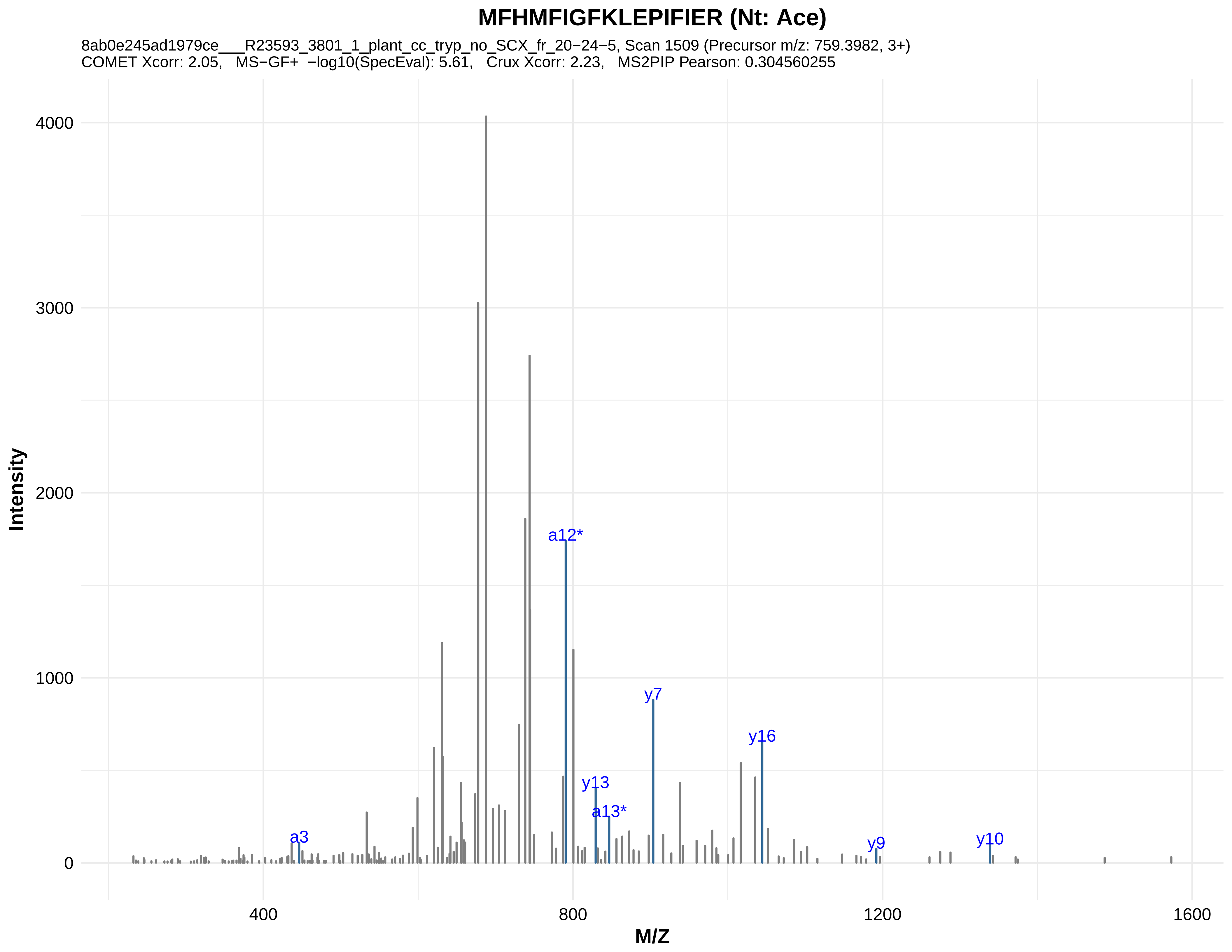
MFHMFIGFKLEPIFIER (Nt: Ace)
8ab0e245ad1979ce___R23593_3801_1_plant_cc_tryp_no_SCX_fr_20−24−5, Scan 1509 (Precursor m/z: 759.3982, 3+)
COMET Xcorr: 2.05, MS−GF+ −log10(SpecEval): 5.61, Crux Xcorr: 2.23, MS2PIP Pearson: 0.304560255
Intensity
M/Z
a12*
y7
y16
y13
a13*
a3
y9
y10
0
1000
2000
3000
4000
400
800
1200
1600

MFLSQWLR (Nt: Ace)

8ab0e245ad1979ce___R23568_3801_1_plant_cc_tryp_no_SCX_fr_28−32−10, Scan 366 (Precursor m/z: 569.7863, 2+)
COMET Xcorr: 1.49, MS−GF+ −log10(SpecEval): 7.9, Crux Xcorr: NA, MS2PIP Pearson: 0.678030335

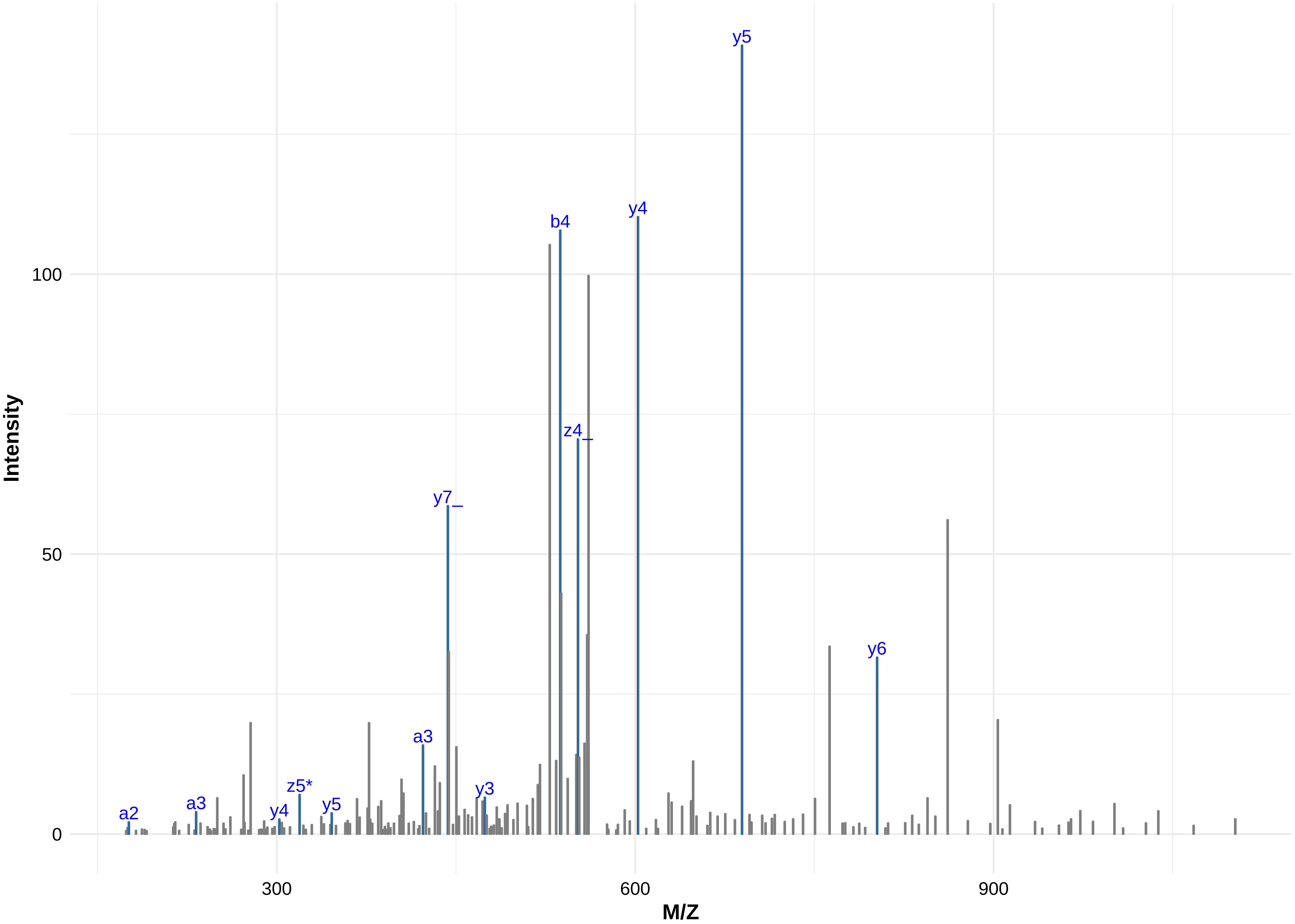

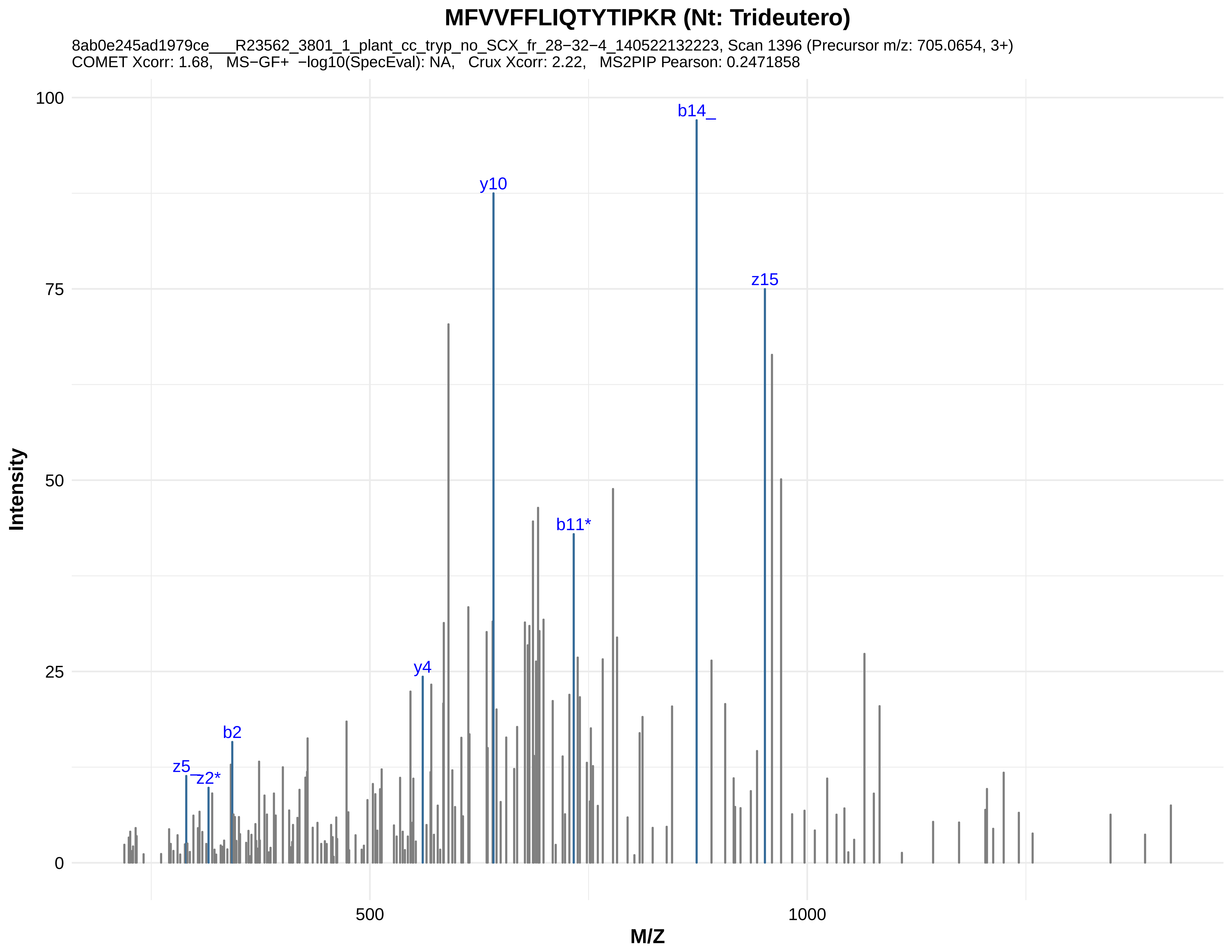

MFVVFFLIQTYTIPKR (Nt: Trideutero)
8ab0e245ad1979ce___R23562_3801_1_plant_cc_tryp_no_SCX_fr_28−32−4_140522132223, Scan 1396 (Precursor m/z: 705.0654, 3+)
COMET Xcorr: 1.68, MS−GF+ −log10(SpecEval): NA, Crux Xcorr: 2.22, MS2PIP Pearson: 0.2471858
Intensity
M/Z
b14_
y10
z15
b11*
y4
b2
z5
z2*

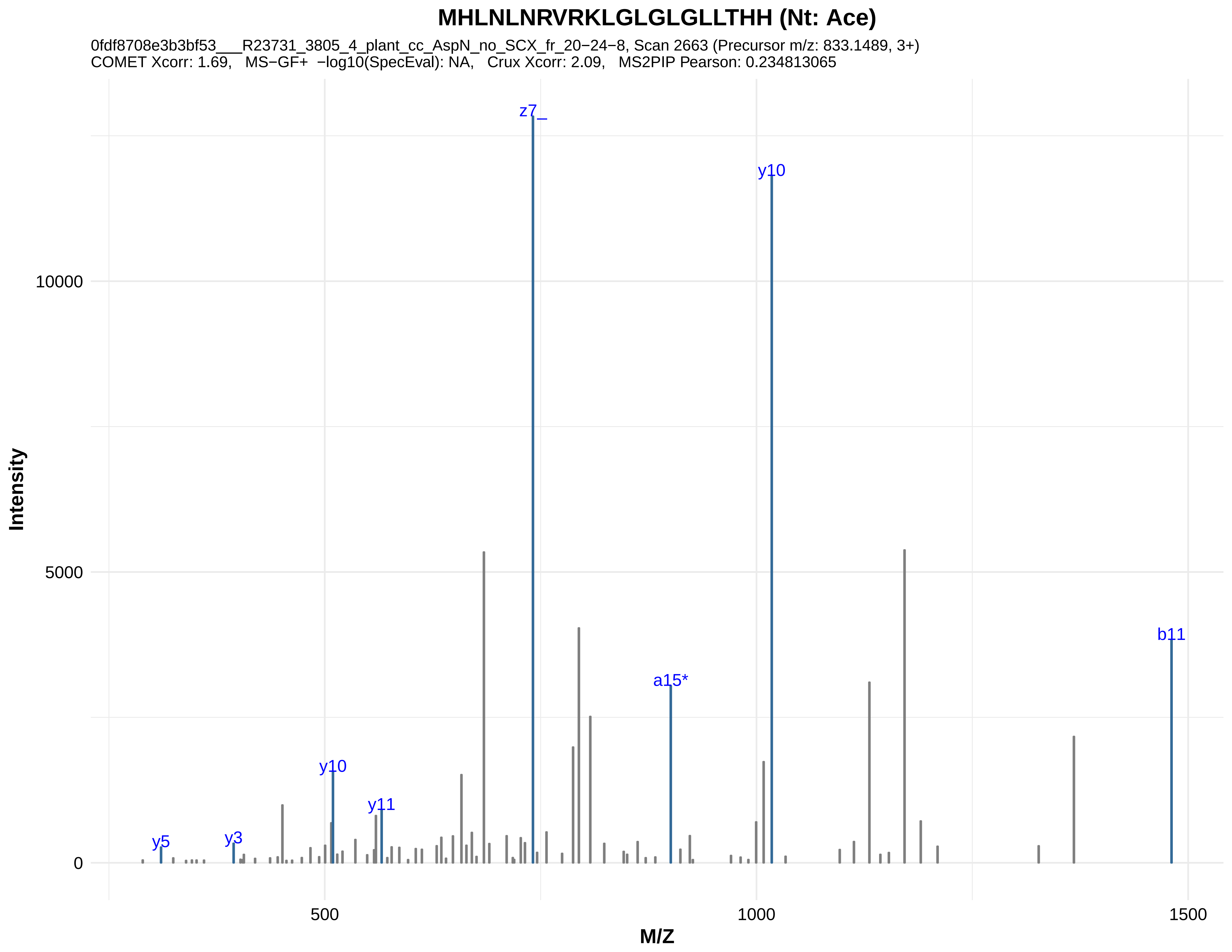
MHLNLNRVRKLGLGLGLLTHH (Nt: Ace)
0fdf8708e3b3bf53___R23731_3805_4_plant_cc_AspN_no_SCX_fr_20−24−8, Scan 2663 (Precursor m/z: 833.1489, 3+)
COMET Xcorr: 1.69, MS−GF+ −log10(SpecEval): NA, Crux Xcorr: 2.09, MS2PIP Pearson: 0.234813065
Intensity
M/Z
z7_
y10
b11
a15*
y10
y11
y5
y3
0
5000
10000
500
1000
1500

MHLNLNRVRKLGLGLGLLTHH (Nt: Ace)

0fdf8708e3b3bf53___R23732_3805_4_plant_cc_AspN_no_SCX_fr_20−24−9, Scan 2602 (Precursor m/z: 833.1482, 3+)
COMET Xcorr: 1.85, MS−GF+ −log10(SpecEval): NA, Crux Xcorr: 1.94, MS2PIP Pearson: 0.241646334

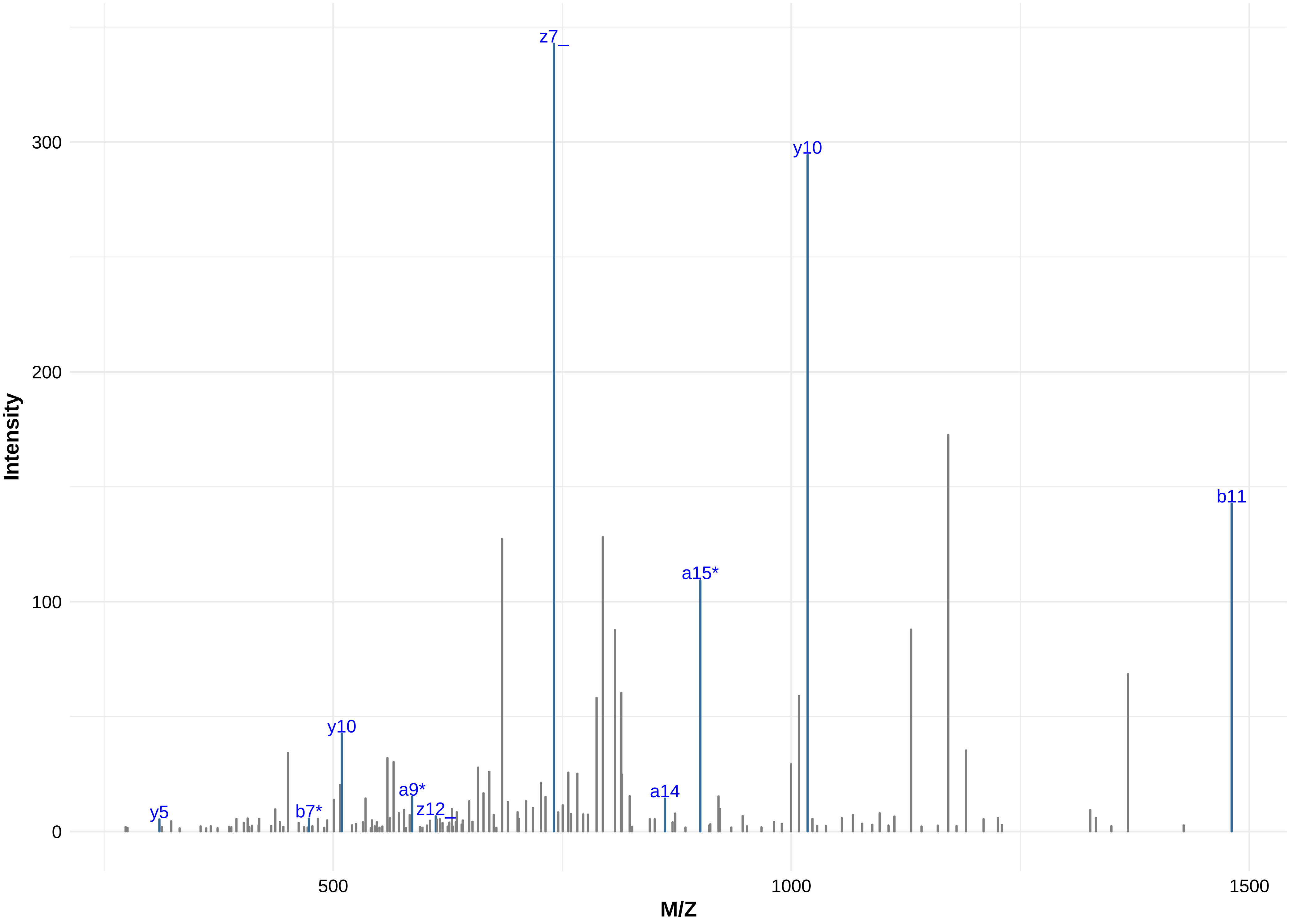

MHRRTINELGF (Nt: Trideutero)

bccdd3e533766d9f___R23626_3802_2_plant_cc_chymo_no_SCX_fr_24−28−8, Scan 641 (Precursor m/z: 479.585, 3+)
COMET Xcorr: 2.22, MS−GF+ −log10(SpecEval): 6.84, Crux Xcorr: 2.83, MS2PIP Pearson: 0.419850805

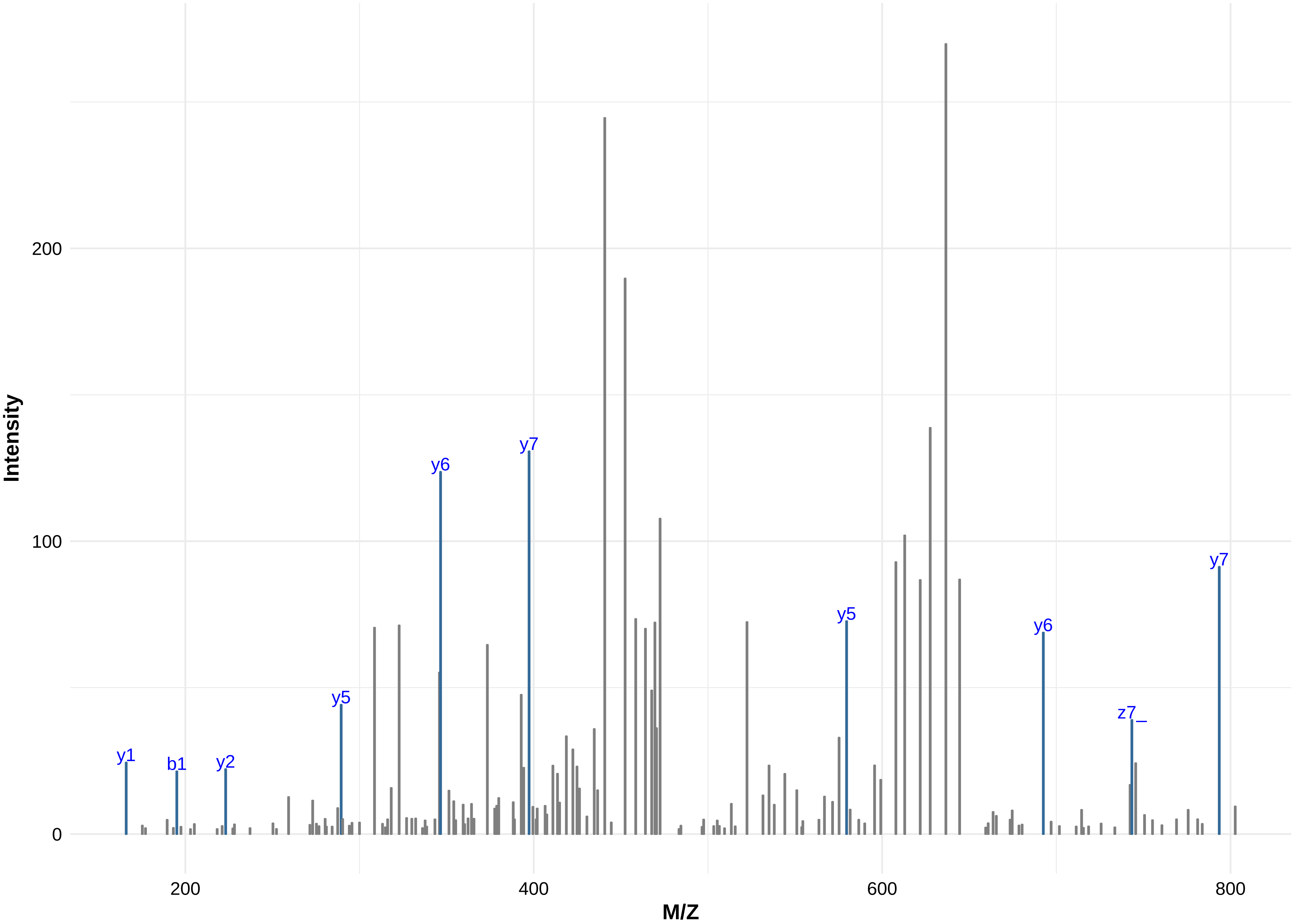

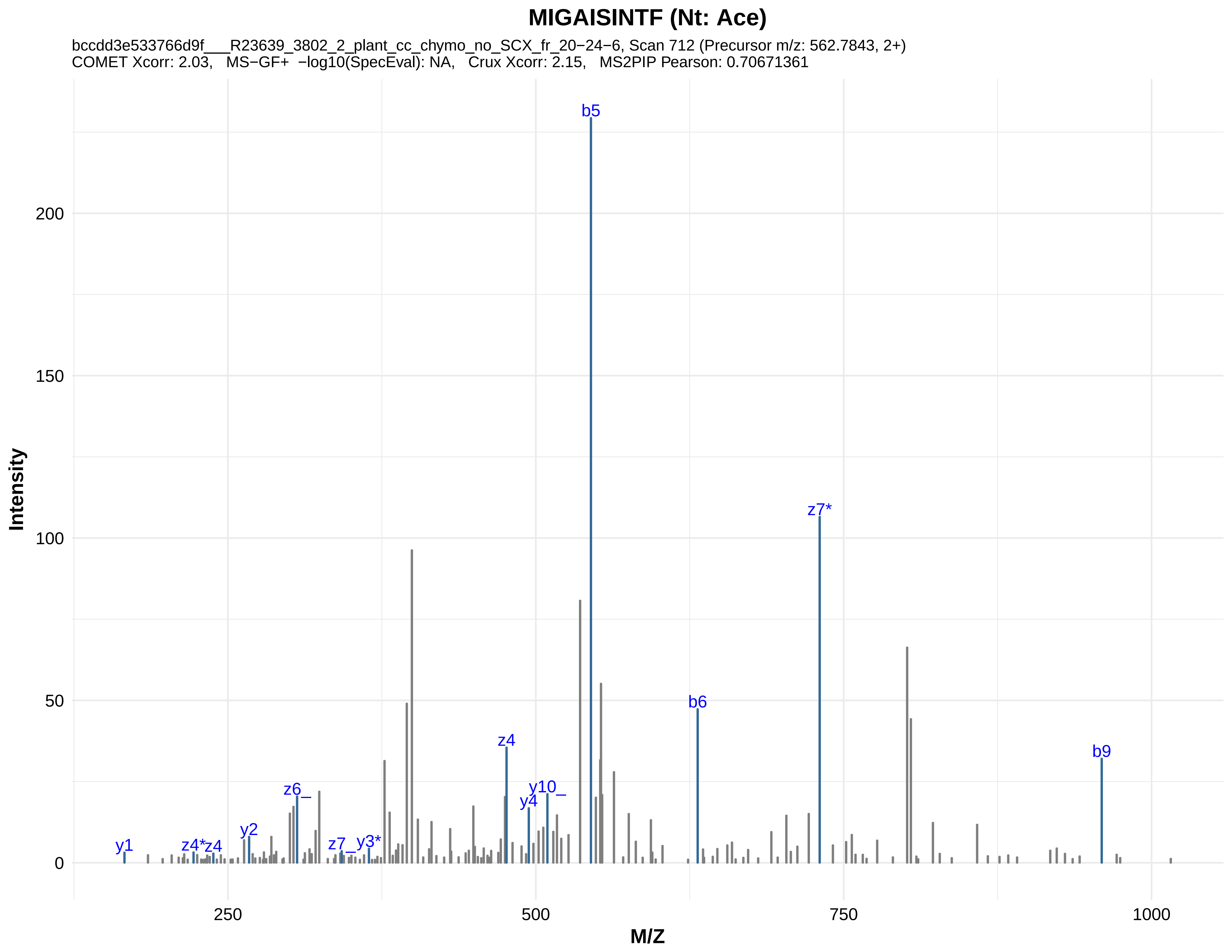

MIGAISINTF (Nt: Ace)
bccdd3e533766d9f___R23639_3802_2_plant_cc_chymo_no_SCX_fr_20−24−6, Scan 712 (Precursor m/z: 562.7843, 2+)
COMET Xcorr: 2.03, MS−GF+ −log10(SpecEval): NA, Crux Xcorr: 2.15, MS2PIP Pearson: 0.70671361
b5
z7*
b6
z4
b9
y10_
y4
z6_
y2
y1
z4*
z4
z7_
y3*
Intensity
M/Z
0
50
100
150
200
250
500
750
1000

MILLNGTEIALLSGLYFTACA (Nt: Ace)

0fdf8708e3b3bf53___R23727_3805_4_plant_cc_AspN_no_SCX_fr_20−24−4, Scan 2046 (Precursor m/z: 777.0663, 3+)
COMET Xcorr: 2.2,   MS−GF+ −log10(SpecEval): NA,   Crux Xcorr: 1.48,   MS2PIP Pearson: 0.306995263

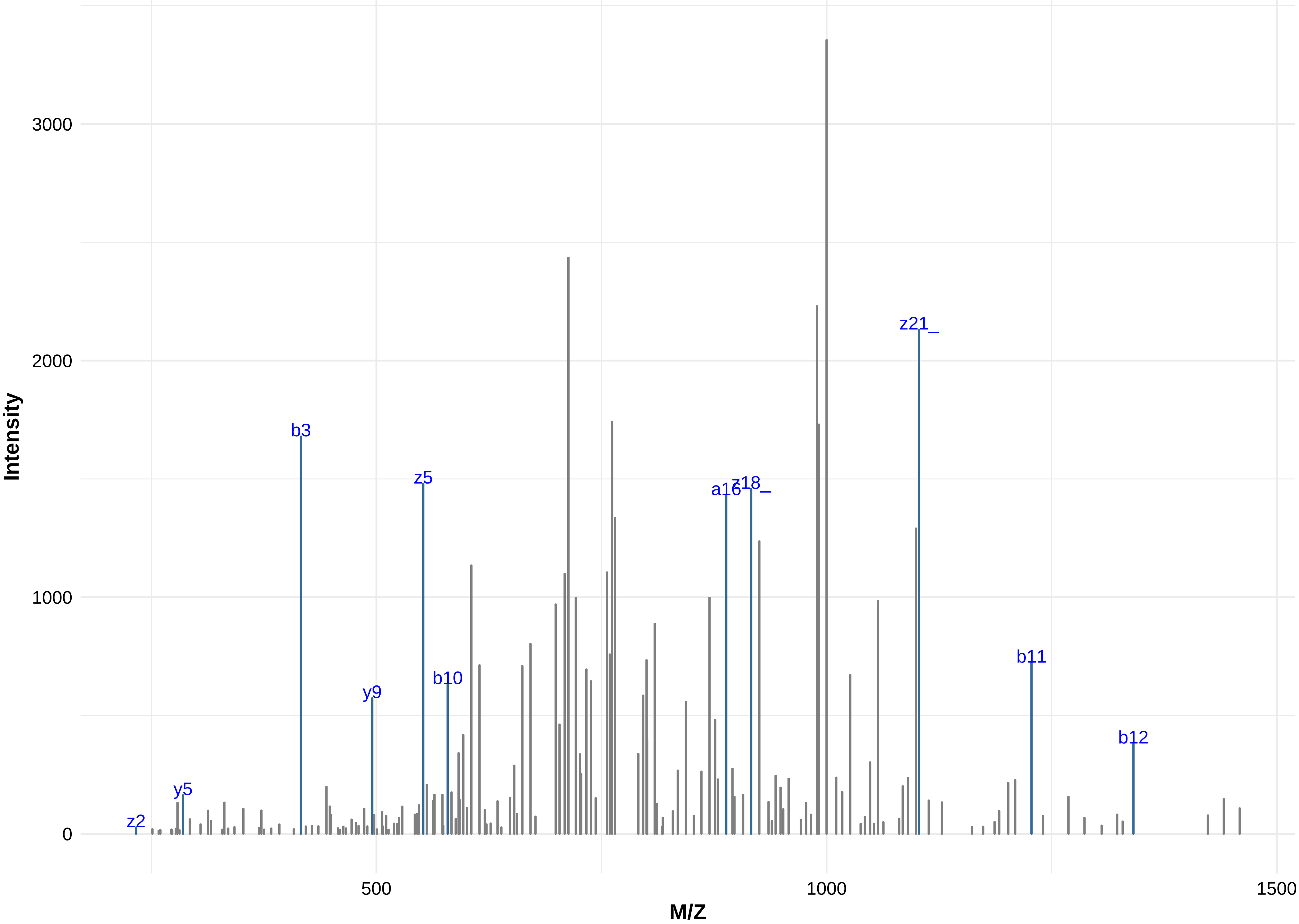

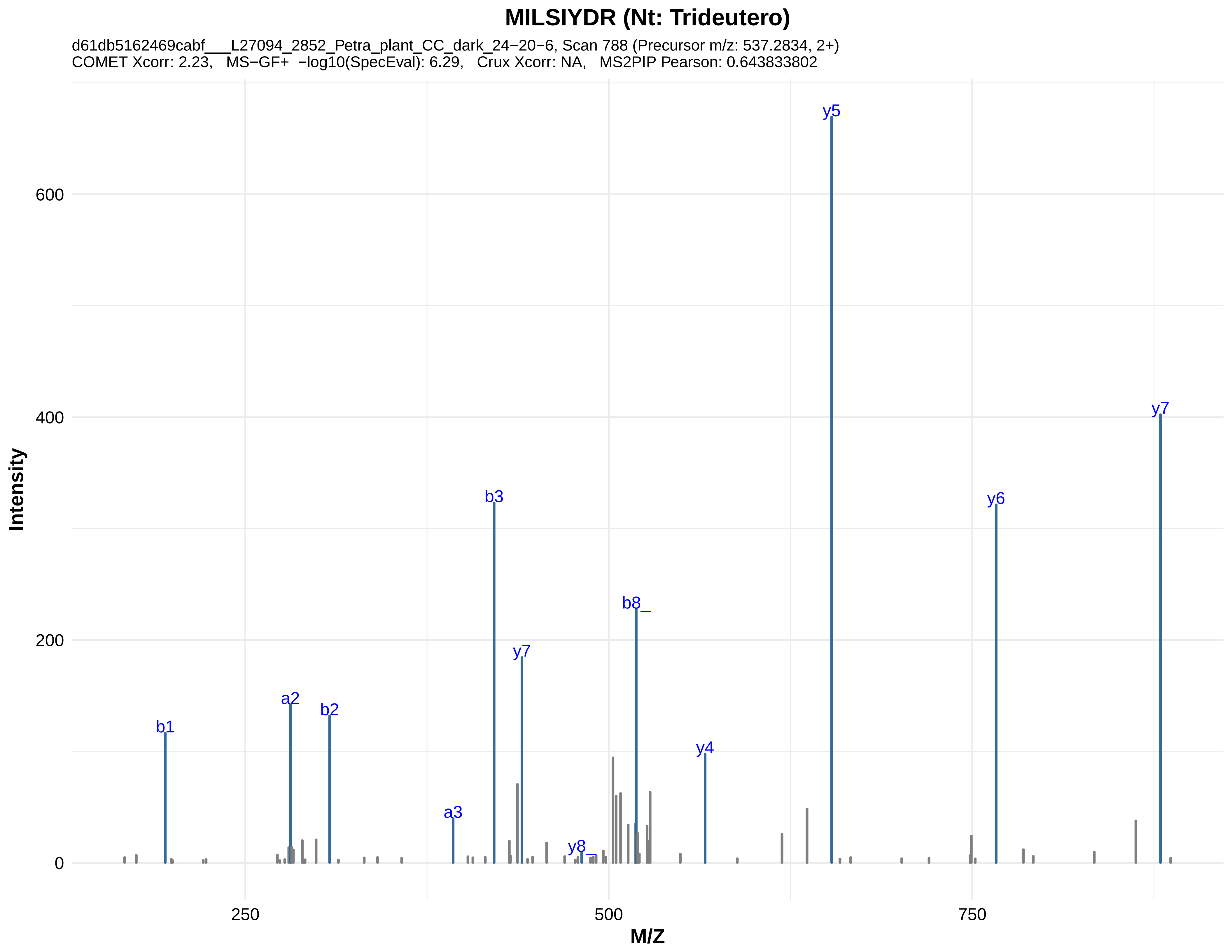
MILSIYDR (Nt: Trideutero)
d61db5162469cabf___L27094_2852_Petra_plant_CC_dark_24−20−6, Scan 788 (Precursor m/z: 537.2834, 2+)
COMET Xcorr: 2.23, MS−GF+ −log10(SpecEval): 6.29, Crux Xcorr: NA, MS2PIP Pearson: 0.643833802
Intensity
M/Z
0
200
400
600
250
500
750
y5
y7
b3
y6
b8_
y7
a2
b2
b1
y4
a3
y8_

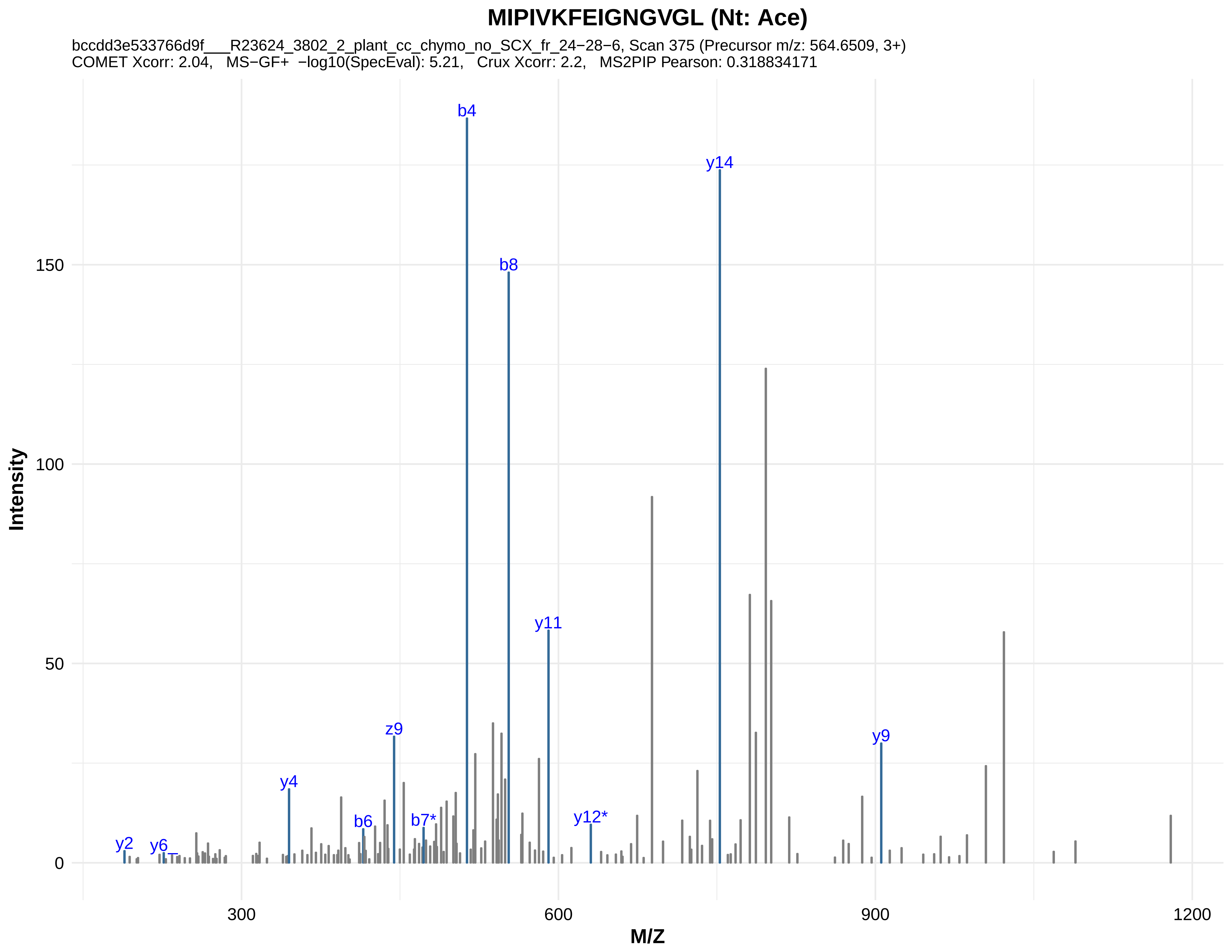
MIPIVKFEIGNGVGL (Nt: Ace)
bccdd3e533766d9f___R23624_3802_2_plant_cc_chymo_no_SCX_fr_24−28−6, Scan 375 (Precursor m/z: 564.6509, 3+)
COMET Xcorr: 2.04, MS−GF+ −log10(SpecEval): 5.21, Crux Xcorr: 2.2, MS2PIP Pearson: 0.318834171
Intensity
M/Z
b4
y14
b8
y11
z9
y9
y4
b6
b7*
y12*
y2
y6
0
50
100
150
300
600
900
1200

MISDYKER (Nt: Ace)

d61db5162469cabf___L27091_2852_Petra_plant_CC_dark_24−20−3, Scan 481 (Precursor m/z: 573.7766, 2+)
COMET Xcorr: 2.03, MS−GF+ −log10(SpecEval): 6.88, Crux Xcorr: 2.03, MS2PIP Pearson: 0.645310215

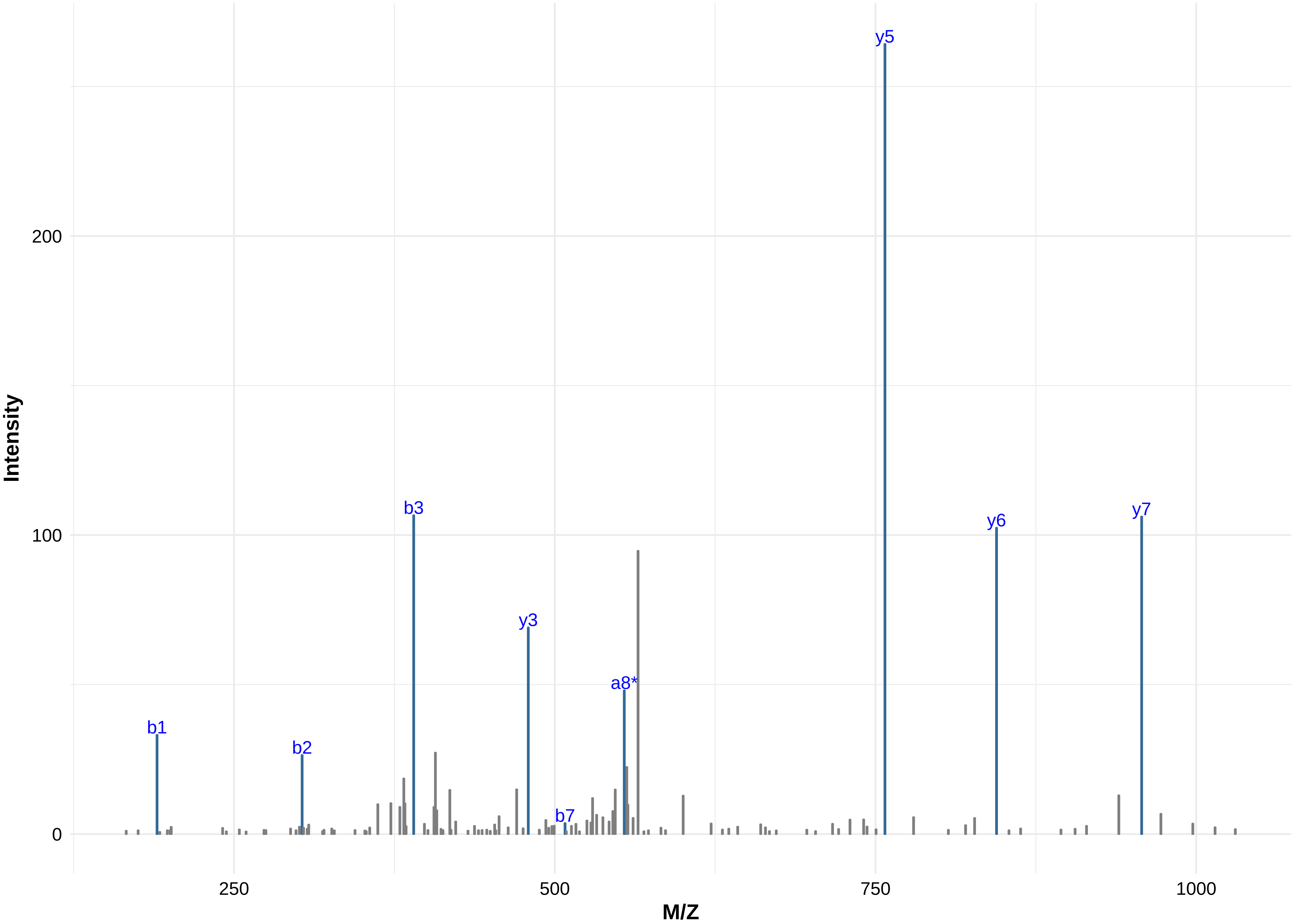

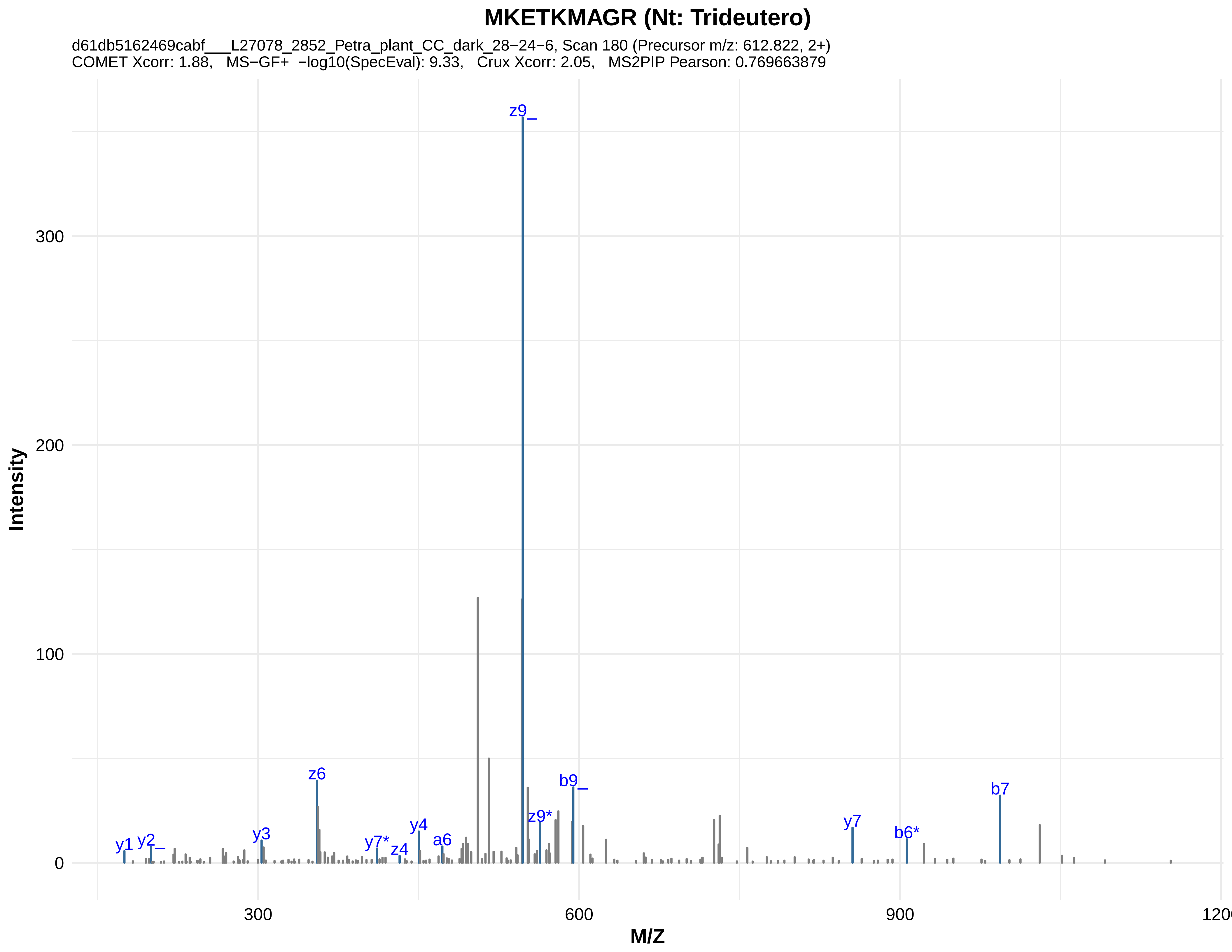

MKETKMAGR (Nt: Trideutero)
d61db5162469cabf___L27078_2852_Petra_plant_CC_dark_28−24−6, Scan 180 (Precursor m/z: 612.822, 2+)
COMET Xcorr: 1.88, MS−GF+ −log10(SpecEval): 9.33, Crux Xcorr: 2.05, MS2PIP Pearson: 0.769663879
Intensity
M/Z
z9_
z6
b9_
b7
z9*
y7
b6*
y4
y3
y7*
z4
a6
y1
y2_
0
100
200
300
300
600
900
1200

MKIAVFHSIE (Nt: Trideutero)

a9eeb67742df5dfc___R23667_3803_3_plant_cc_GluC_no_SCX_fr_24−28−4, Scan 2539 (Precursor m/z: 642.8468, 2+)
COMET Xcorr: 2.42, MS−GF+ −log10(SpecEval): 6.45, Crux Xcorr: 2.38, MS2PIP Pearson: 0.602067704

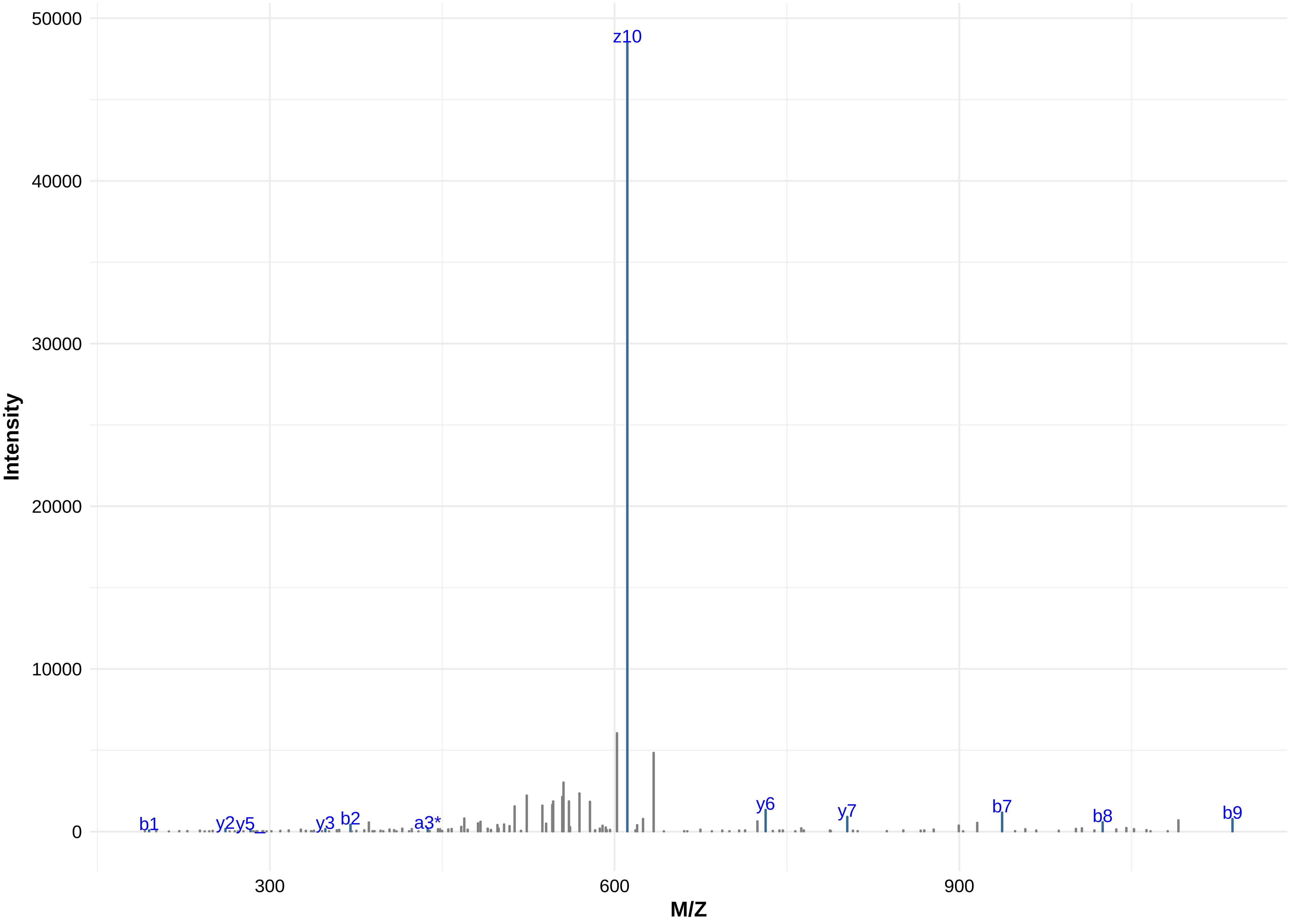

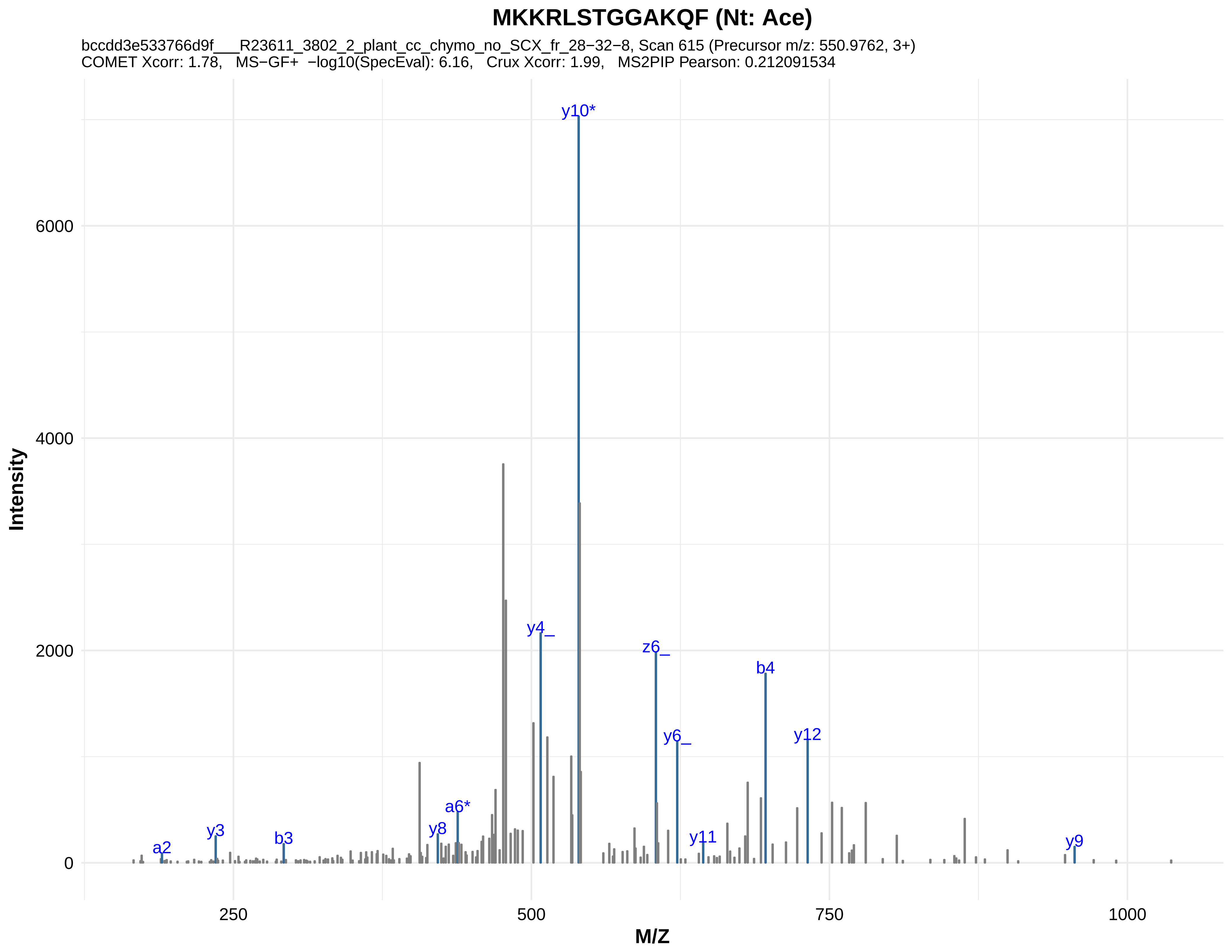

MKKRLSTGGAKQF (Nt: Ace)
bccdd3e533766d9f___R23611_3802_2_plant_cc_chymo_no_SCX_fr_28−32−8, Scan 615 (Precursor m/z: 550.9762, 3+)
COMET Xcorr: 1.78, MS−GF+ −log10(SpecEval): 6.16, Crux Xcorr: 1.99, MS2PIP Pearson: 0.212091534
y10*
y4_
z6_
b4
y6_
y12
a6*
y8
y3
b3
a2
y11
y9
Intensity
0
2000
4000
6000
250
500
750
1000
M/Z

MKNKSDKEPKSKNISEICLL (Nt: Ace)

bccdd3e533766d9f___R23628_3802_2_plant_cc_chymo_no_SCX_fr_24−28−10, Scan 1667 (Precursor m/z: 885.8185, 3+)
COMET Xcorr: 2.43, MS−GF+ −log10(SpecEval): 4.27, Crux Xcorr: 1.4, MS2PIP Pearson: 0.306663671

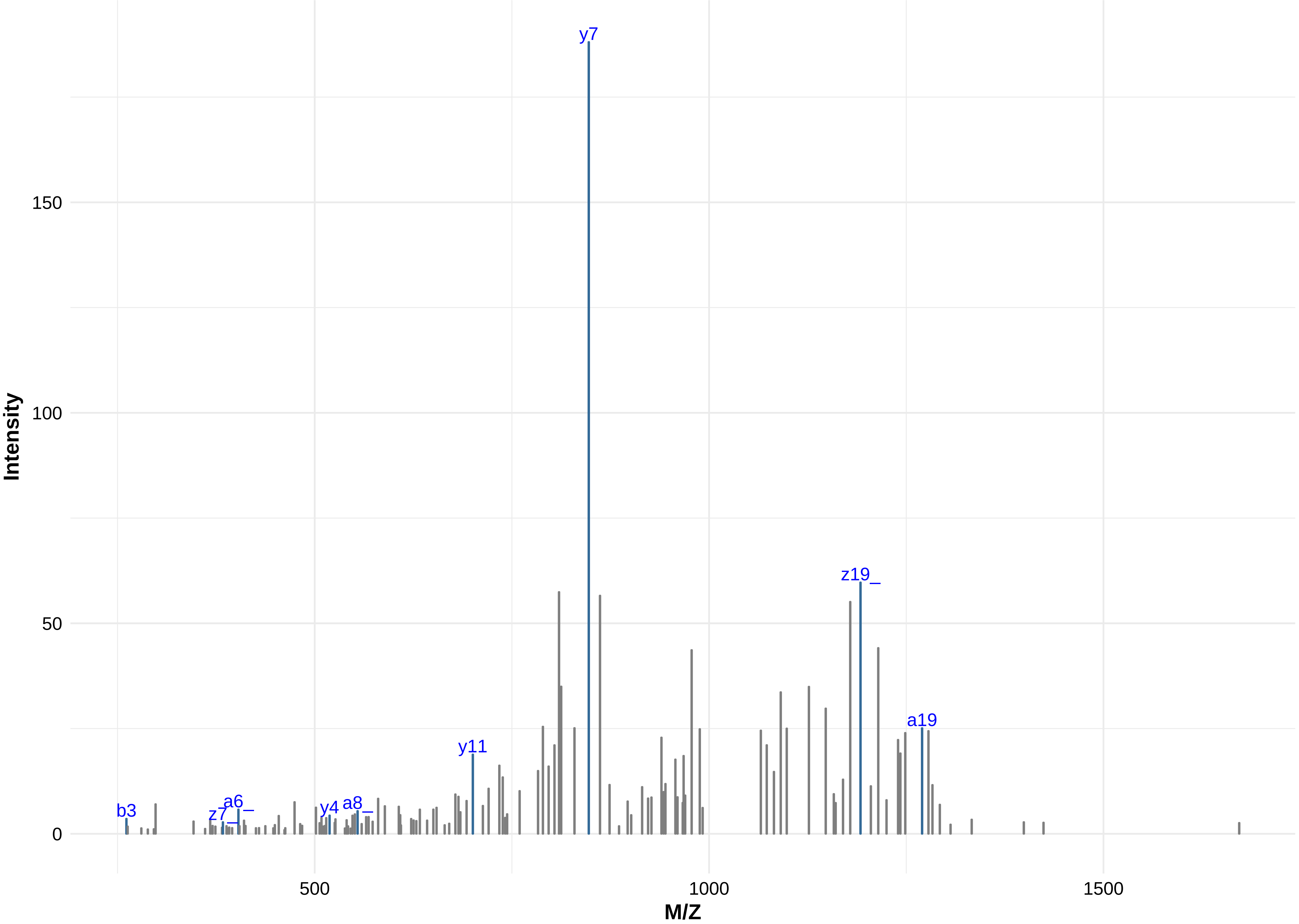

MKTKVGNIVLNSKSSRGAMQSNGE (Nt: Trideutero)

a9eeb67742df5dfc___R23679_3803_3_plant_cc_GluC_no_SCX_fr_20−24−1, Scan 2246 (Precursor m/z: 919.4865, 3+)
COMET Xcorr: 1.74, MS−GF+ −log10(SpecEval): NA, Crux Xcorr: 2, MS2PIP Pearson: 0.288699907

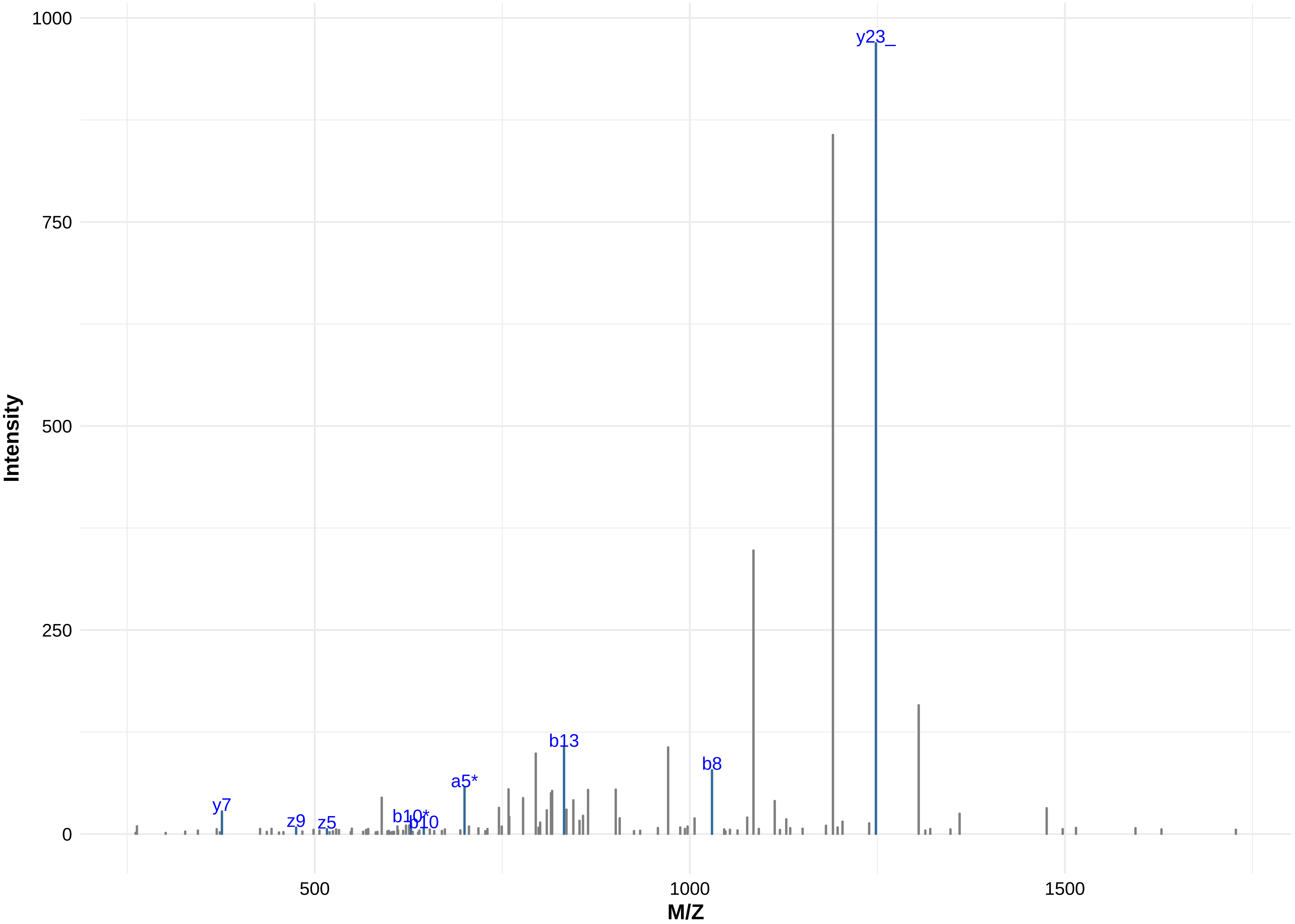

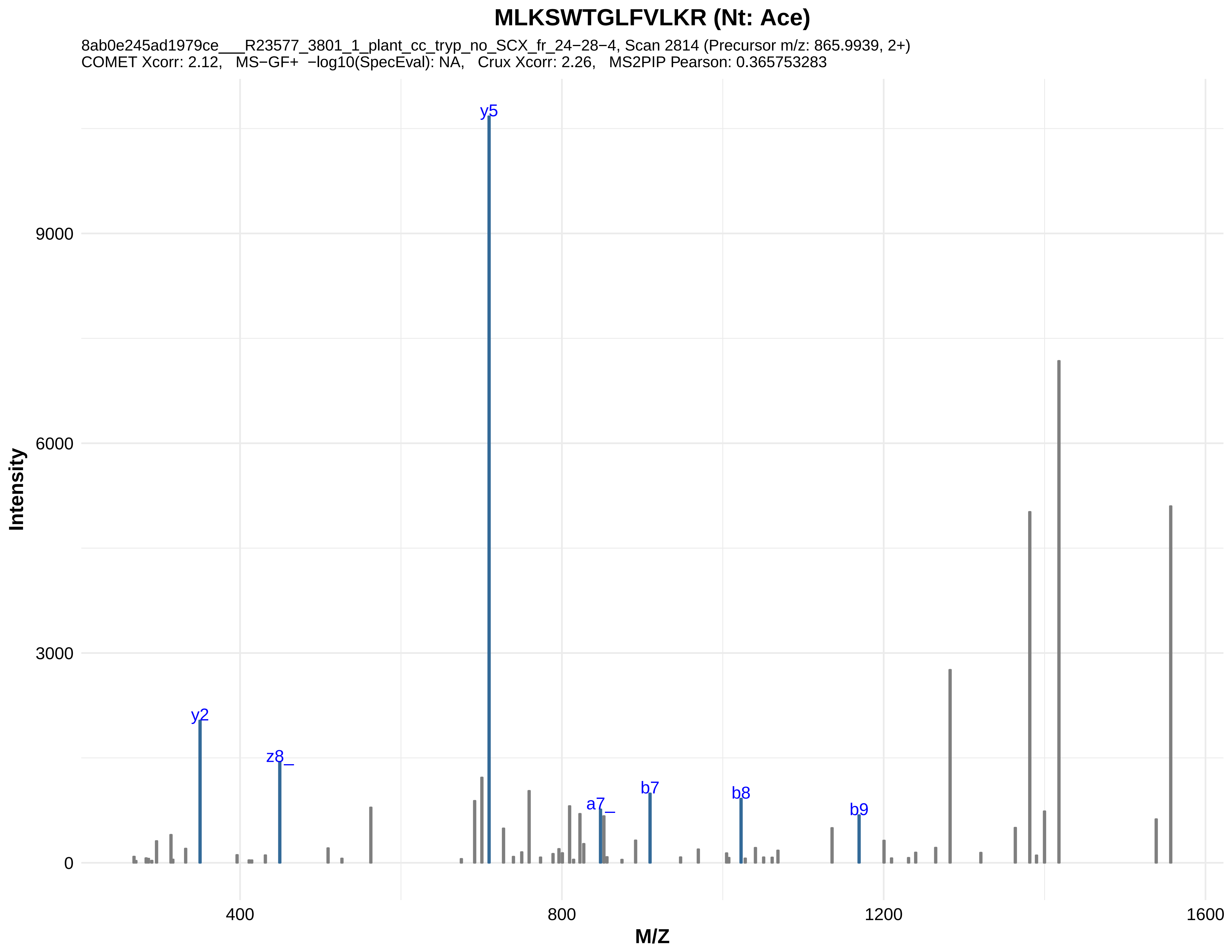

MLKSWTGLFVLKR (Nt: Ace)
8ab0e245ad1979ce___R23577_3801_1_plant_cc_tryp_no_SCX_fr_24−28−4, Scan 2814 (Precursor m/z: 865.9939, 2+)
COMET Xcorr: 2.12, MS−GF+ −log10(SpecEval): NA, Crux Xcorr: 2.26, MS2PIP Pearson: 0.365753283
Intensity
9000
6000
3000
0
y5
y2
z8_
a7_
b7
b8
b9
400
800
1200
1600
M/Z

MLQEHCINQMTFFYKQKFHK (Nt: Trideutero)

0fdf8708e3b3bf53___R23696_3805_4_plant_cc_AspN_no_SCX_fr_28−32−3, Scan 2077 (Precursor m/z: 960.1454, 3+)
COMET Xcorr: 1.53,   MS−GF+ −log10(SpecEval): NA,   Crux Xcorr: 1.95,   MS2PIP Pearson: 0.133402618

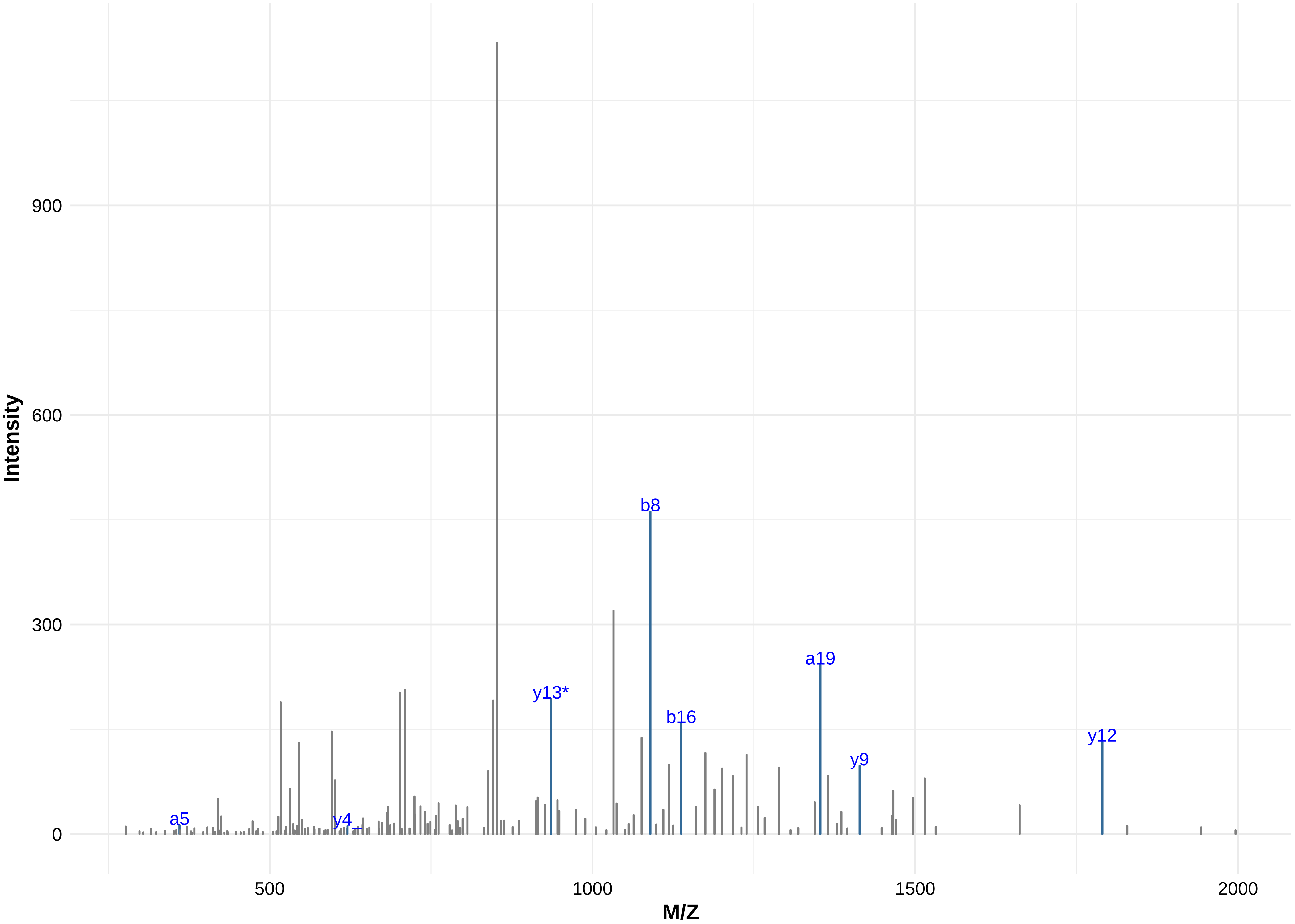

MLRSVAFDDFR (Nt: Trideutero)

0fdf8708e3b3bf53___R23694_3805_4_plant_cc_AspN_no_SCX_fr_28−32−1, Scan 897 (Precursor m/z: 473.9024, 3+)
COMET Xcorr: 2.09,   MS−GF+ −log10(SpecEval): 5.33,   Crux Xcorr: 1.82,   MS2PIP Pearson: 0.451884639

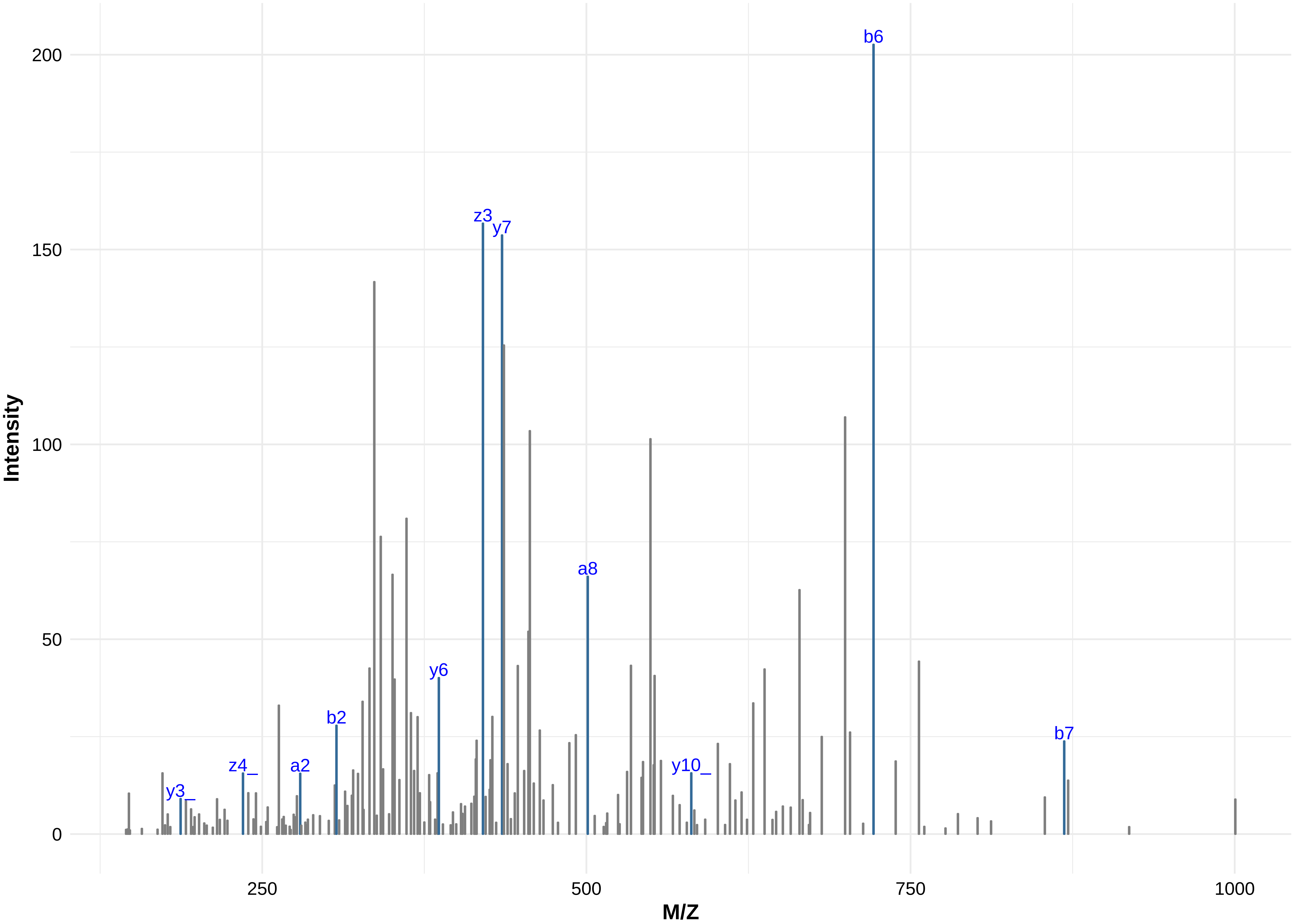

MLSLQLINHK (Nt: Trideutero)

0fdf8708e3b3bf53___R23709_3805_4_plant_cc_AspN_no_SCX_fr_24−28−1, Scan 2425 (Precursor m/z: 653.872, 2+)
COMET Xcorr: 2.13, MS−GF+ −log10(SpecEval): 5.26, Crux Xcorr: 1.92, MS2PIP Pearson: 0.460313542

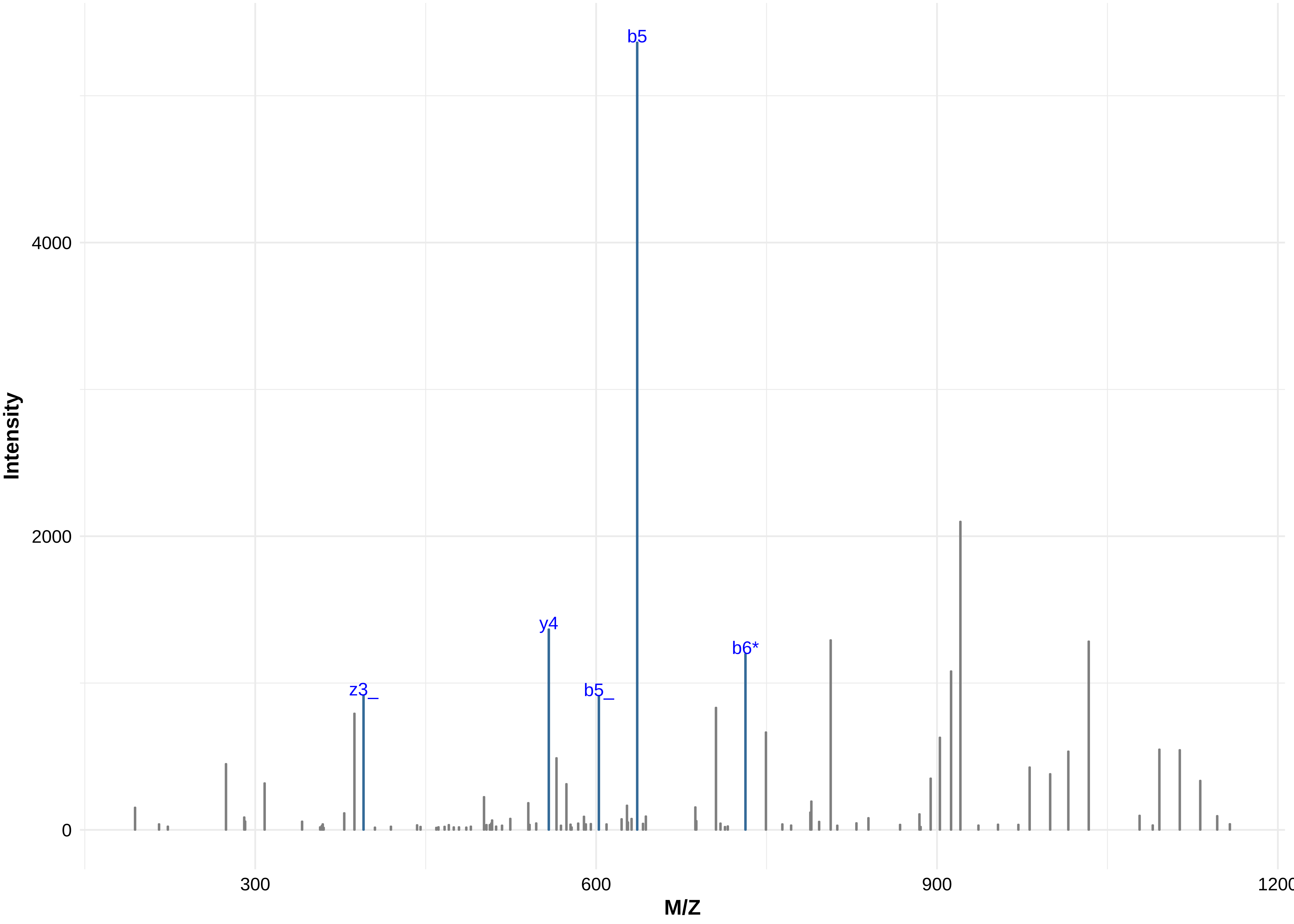

MLTVAMKRLVGWISVKED (Nt: Ace)

a9eeb67742df5dfc___R23685_3803_3_plant_cc_GluC_no_SCX_fr_20−24−7, Scan 963 (Precursor m/z: 748.7459, 3+)
COMET Xcorr: 1.2, MS−GF+ −log10(SpecEval): 5.27, Crux Xcorr: 2.25, MS2PIP Pearson: 0.350918257

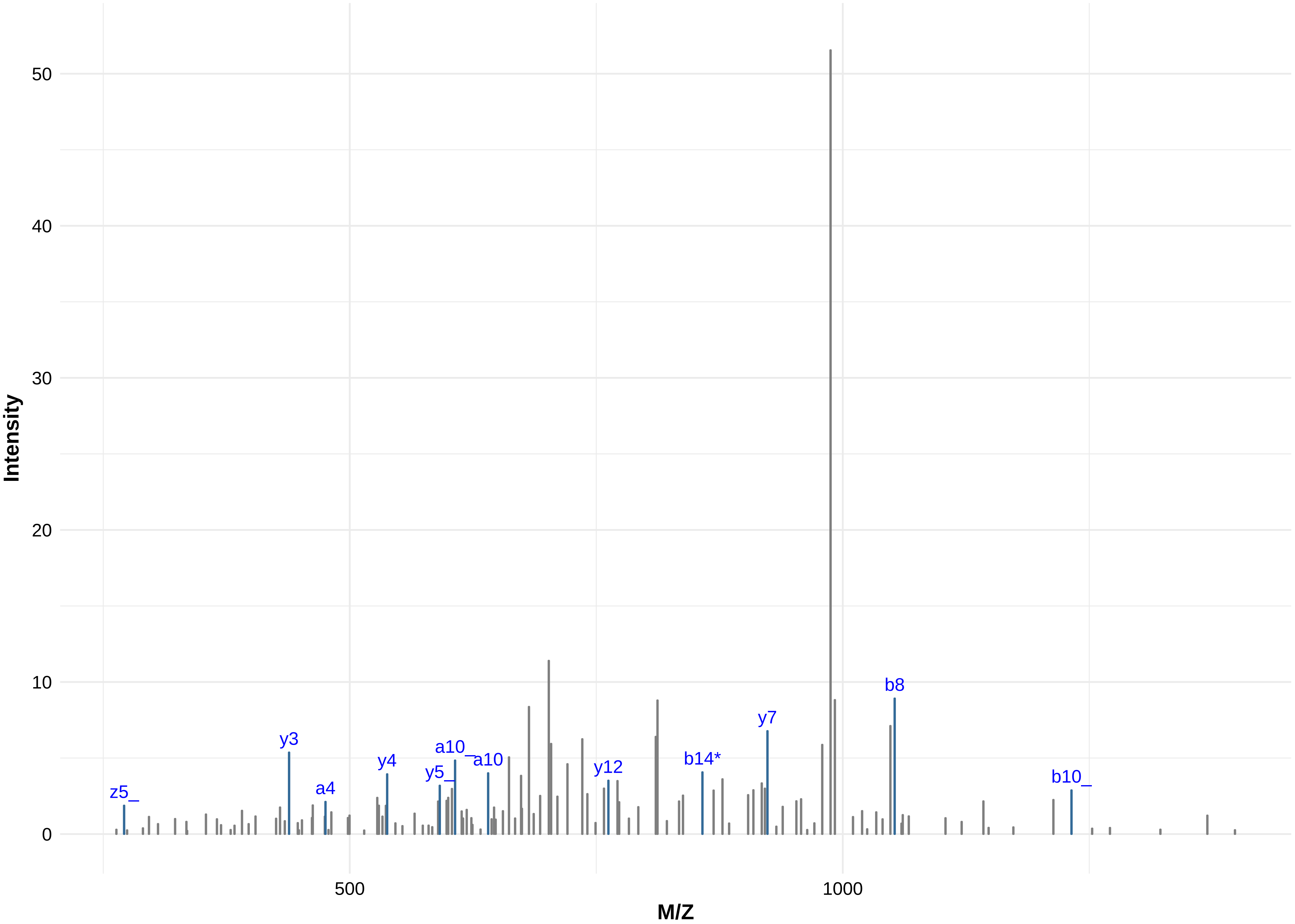

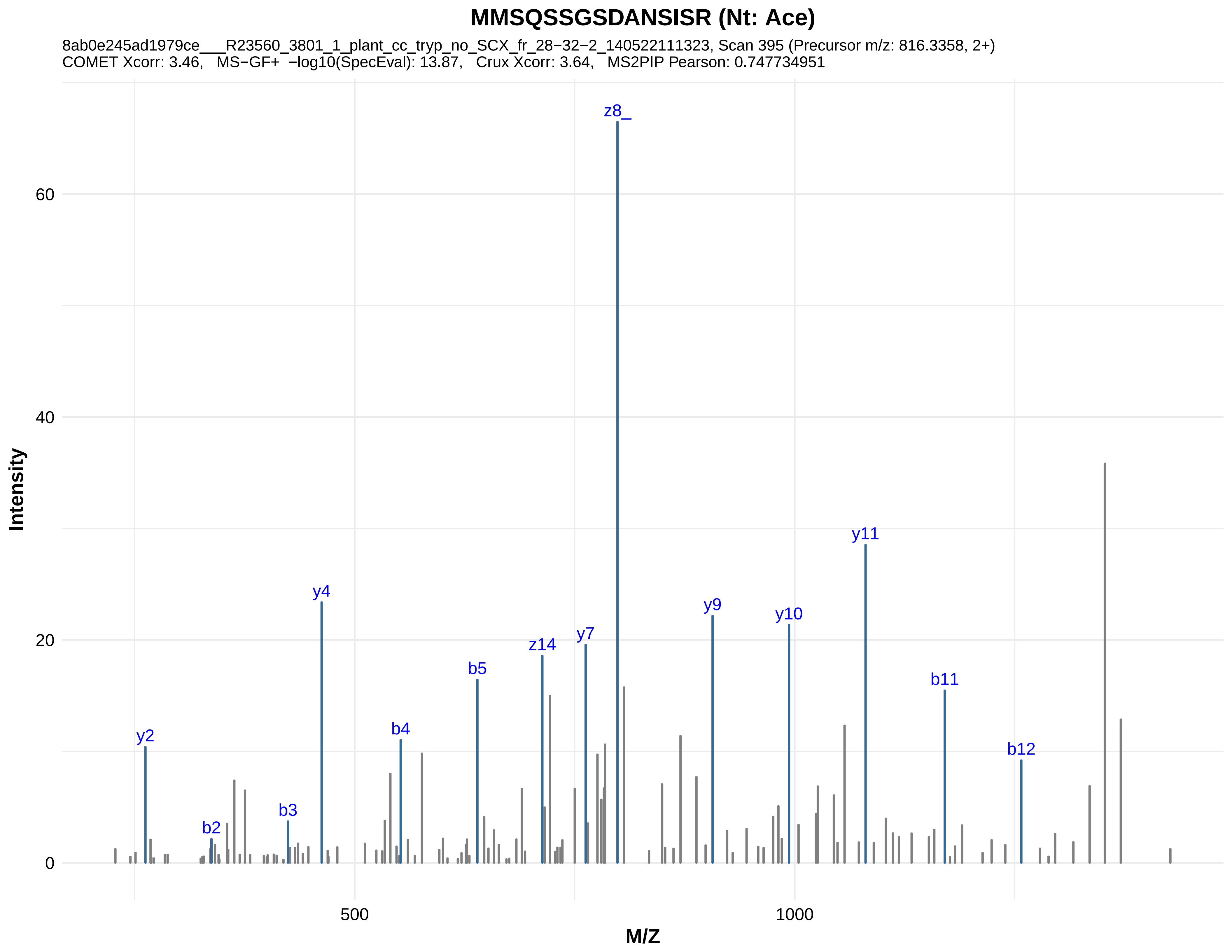
MMSQSSGSDANSISR (Nt: Ace)
8ab0e245ad1979ce___R23560_3801_1_plant_cc_tryp_no_SCX_fr_28−32−2_140522111323, Scan 395 (Precursor m/z: 816.3358, 2+)
COMET Xcorr: 3.46, MS−GF+ −log10(SpecEval): 13.87, Crux Xcorr: 3.64, MS2PIP Pearson: 0.747734951
z8_
y11
y4
y9
y10
z14
y7
b5
b11
b4
b12
y2
b3
b2
Intensity
M/Z
0
20
40
60
500
1000

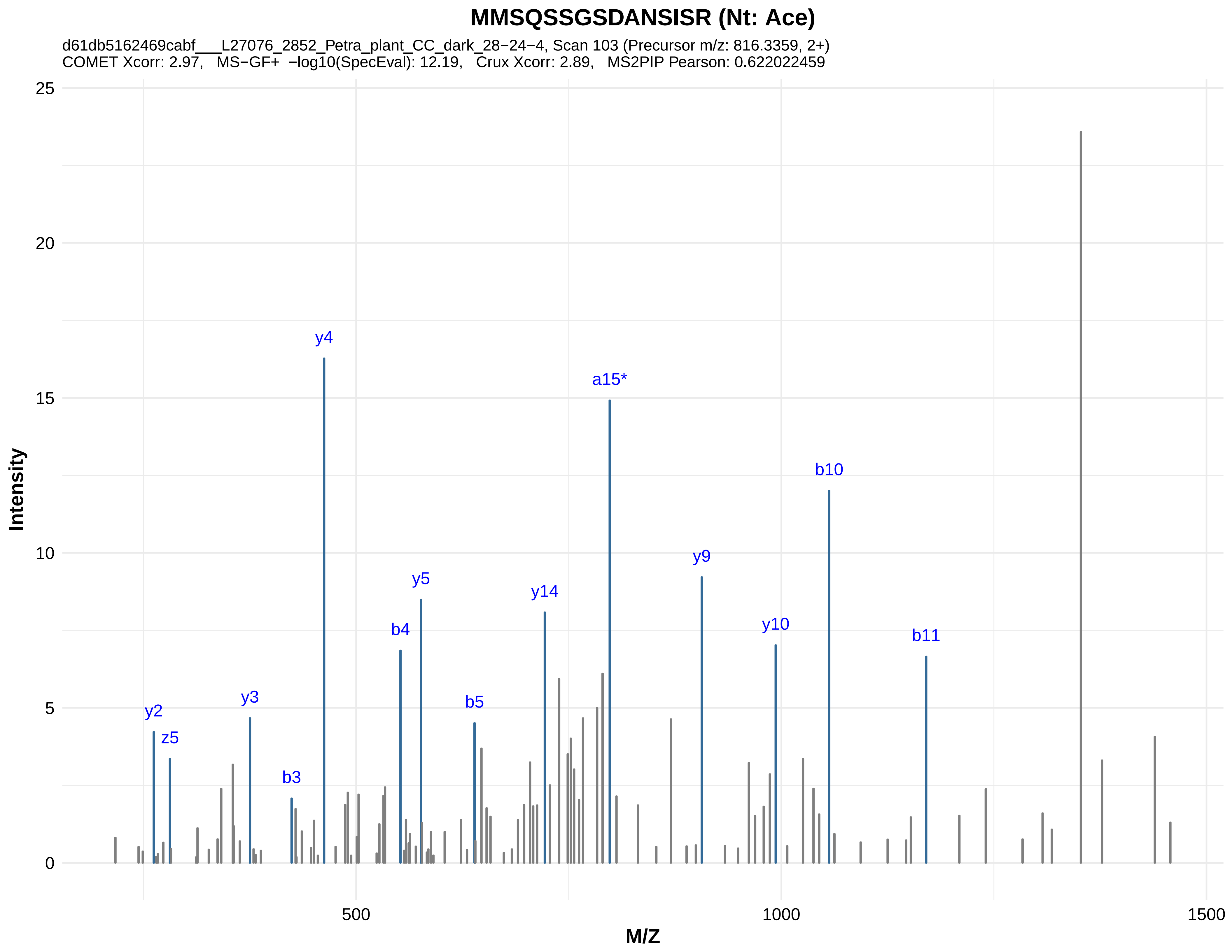
MMSQSSGSDANSISR (Nt: Ace)
d61db5162469cabf___L27076_2852_Petra_plant_CC_dark_28−24−4, Scan 103 (Precursor m/z: 816.3359, 2+)
COMET Xcorr: 2.97, MS−GF+ −log10(SpecEval): 12.19, Crux Xcorr: 2.89, MS2PIP Pearson: 0.622022459
Intensity
M/Z
y2
z5
y3
b3
y4
b4
y5
b5
y14
a15*
y9
y10
b10
b11
0
5
10
15
20
25
500
1000
1500

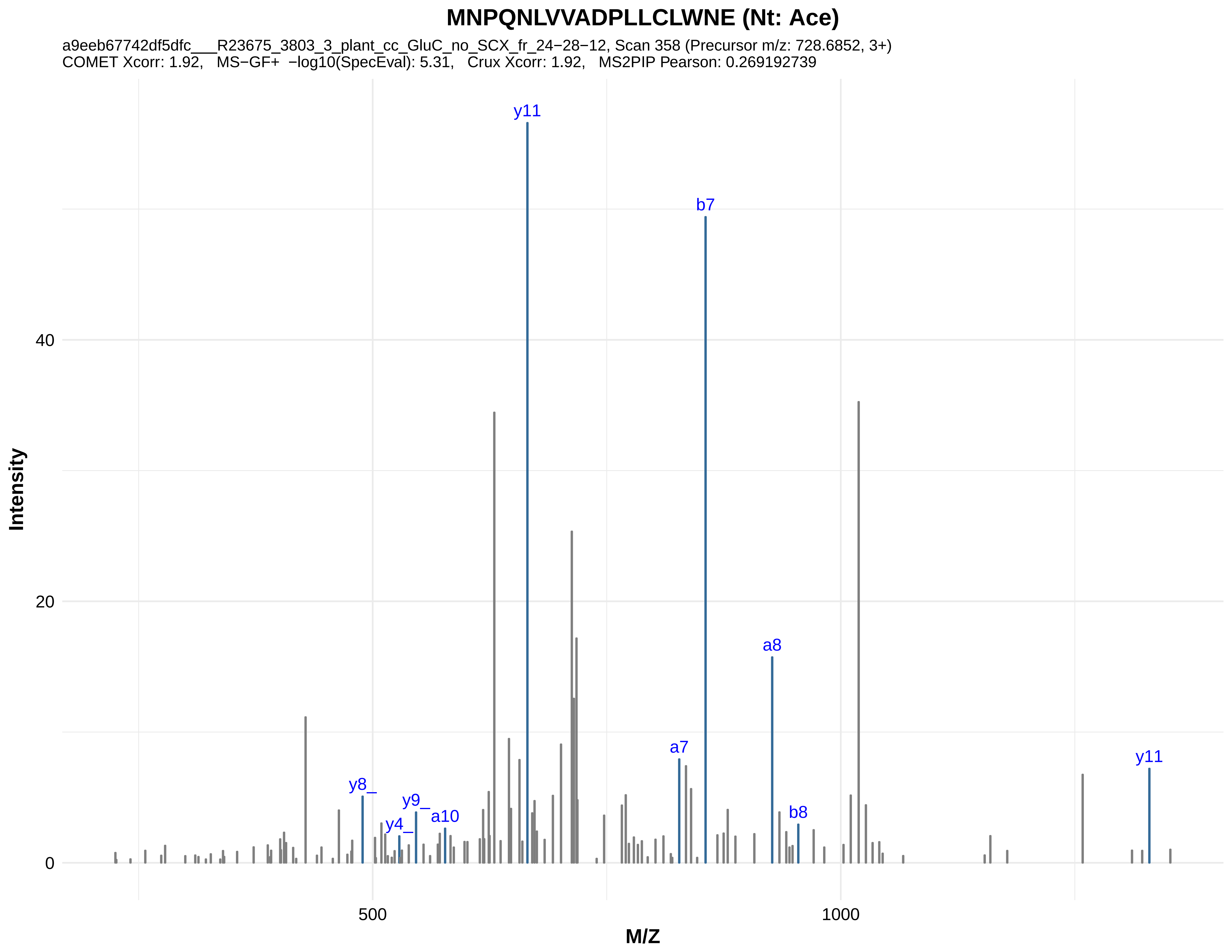

MNPQNLVVADPLLCLWNE (Nt: Ace)
a9eeb67742df5dfc___R23675_3803_3_plant_cc_GluC_no_SCX_fr_24−28−12, Scan 358 (Precursor m/z: 728.6852, 3+)
COMET Xcorr: 1.92, MS−GF+ −log10(SpecEval): 5.31, Crux Xcorr: 1.92, MS2PIP Pearson: 0.269192739
y11
b7
a8
a7
y11
y8_
y9_
y4_
a10
b8
Intensity
40
20
0
500
1000
M/Z

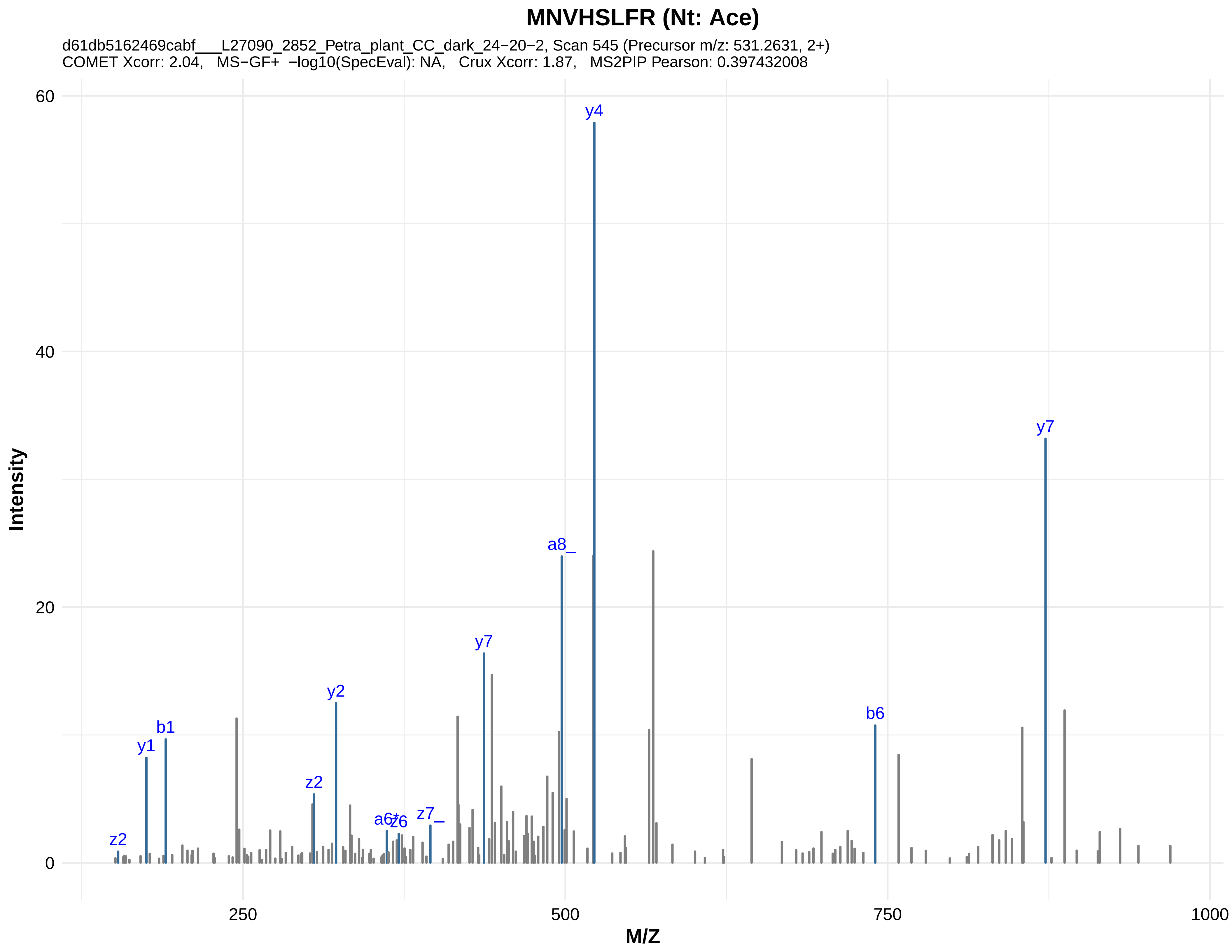

MNVHSLFR (Nt: Ace)
d61db5162469cabf___L27090_2852_Petra_plant_CC_dark_24−20−2, Scan 545 (Precursor m/z: 531.2631, 2+)
COMET Xcorr: 2.04, MS−GF+ −log10(SpecEval): NA, Crux Xcorr: 1.87, MS2PIP Pearson: 0.397432008
Intensity
M/Z
y4
y7
a8_
y7
y2
b1
y1
b6
z2
z2
a6*
z6
z7_
0
20
40
60
250
500
750
1000

MQFQFVVWGLFNTIQYTYIVI (Nt: Trideutero)

0fdf8708e3b3bf53___R23703_3805_4_plant_cc_AspN_no_SCX_fr_28−32−10, Scan 2770 (Precursor m/z: 891.7959, 3+)
COMET Xcorr: 2.02,   MS−GF+ −log10(SpecEval): NA,   Crux Xcorr: 1.65,   MS2PIP Pearson: 0.216877876

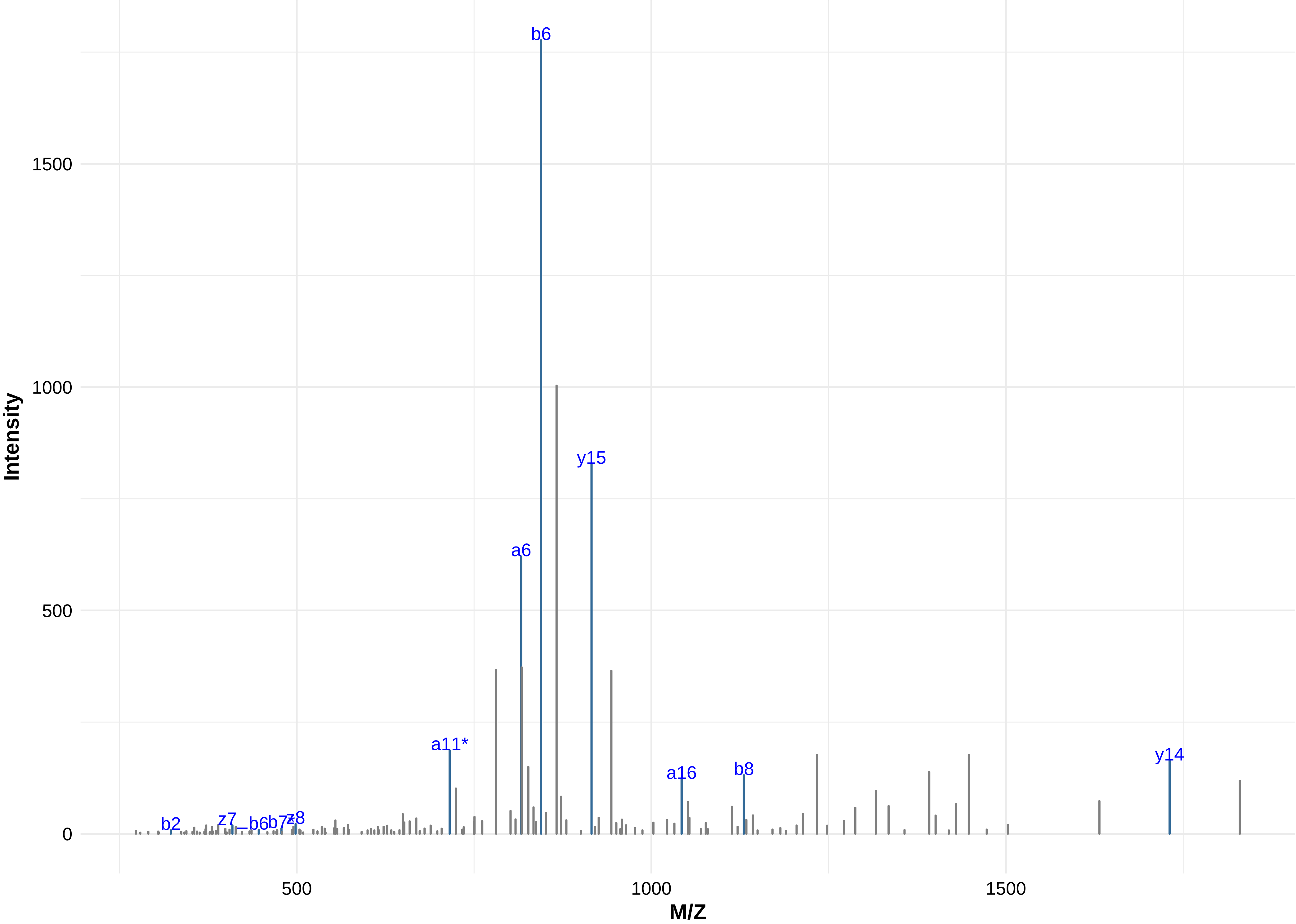

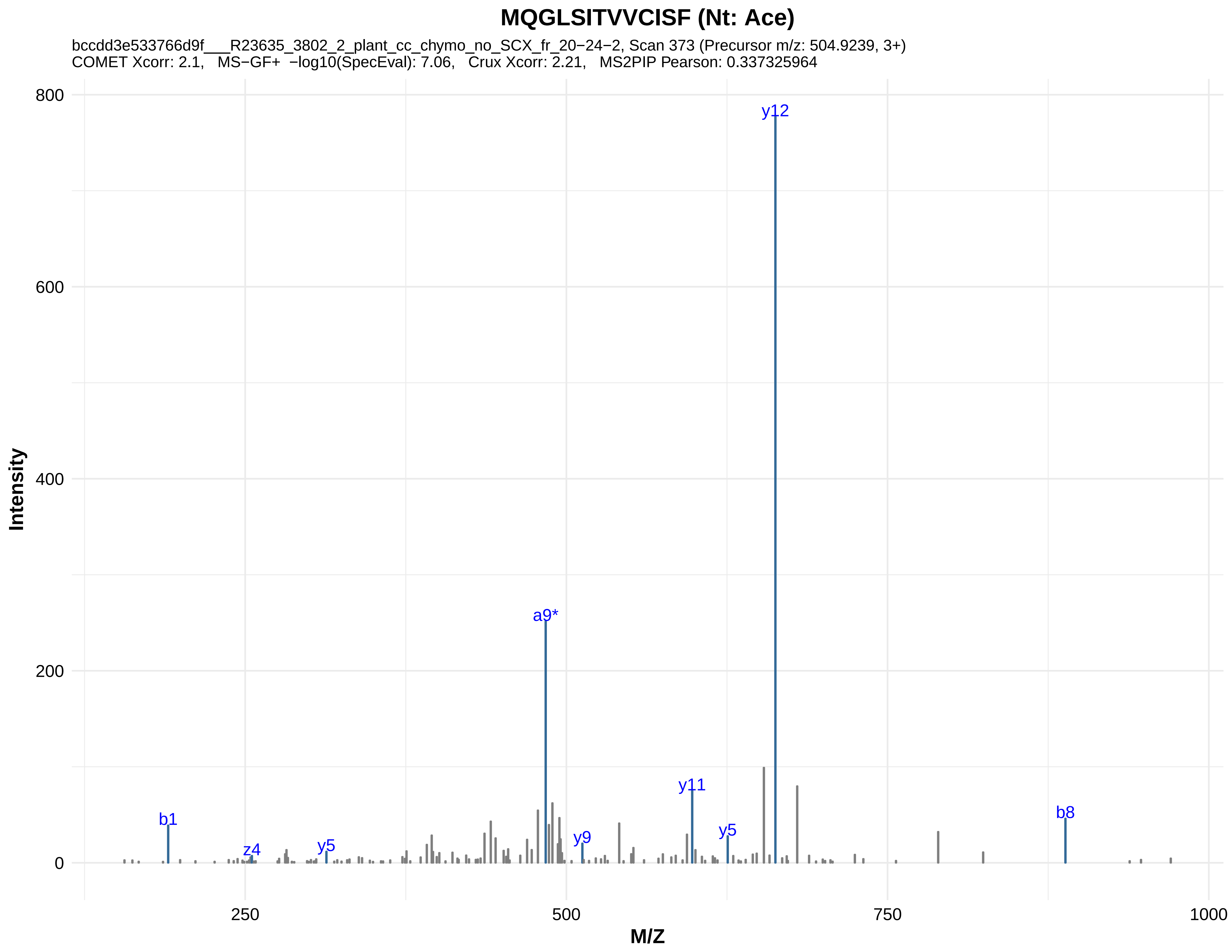
MQGLSITVVCISF (Nt: Ace)
bccdd3e533766d9f___R23635_3802_2_plant_cc_chymo_no_SCX_fr_20−24−2, Scan 373 (Precursor m/z: 504.9239, 3+)
COMET Xcorr: 2.1, MS−GF+ −log10(SpecEval): 7.06, Crux Xcorr: 2.21, MS2PIP Pearson: 0.337325964
Intensity
M/Z
0
200
400
600
800
250
500
750
1000
y12
a9*
y11
b8
b1
z4
y5
y9
y5

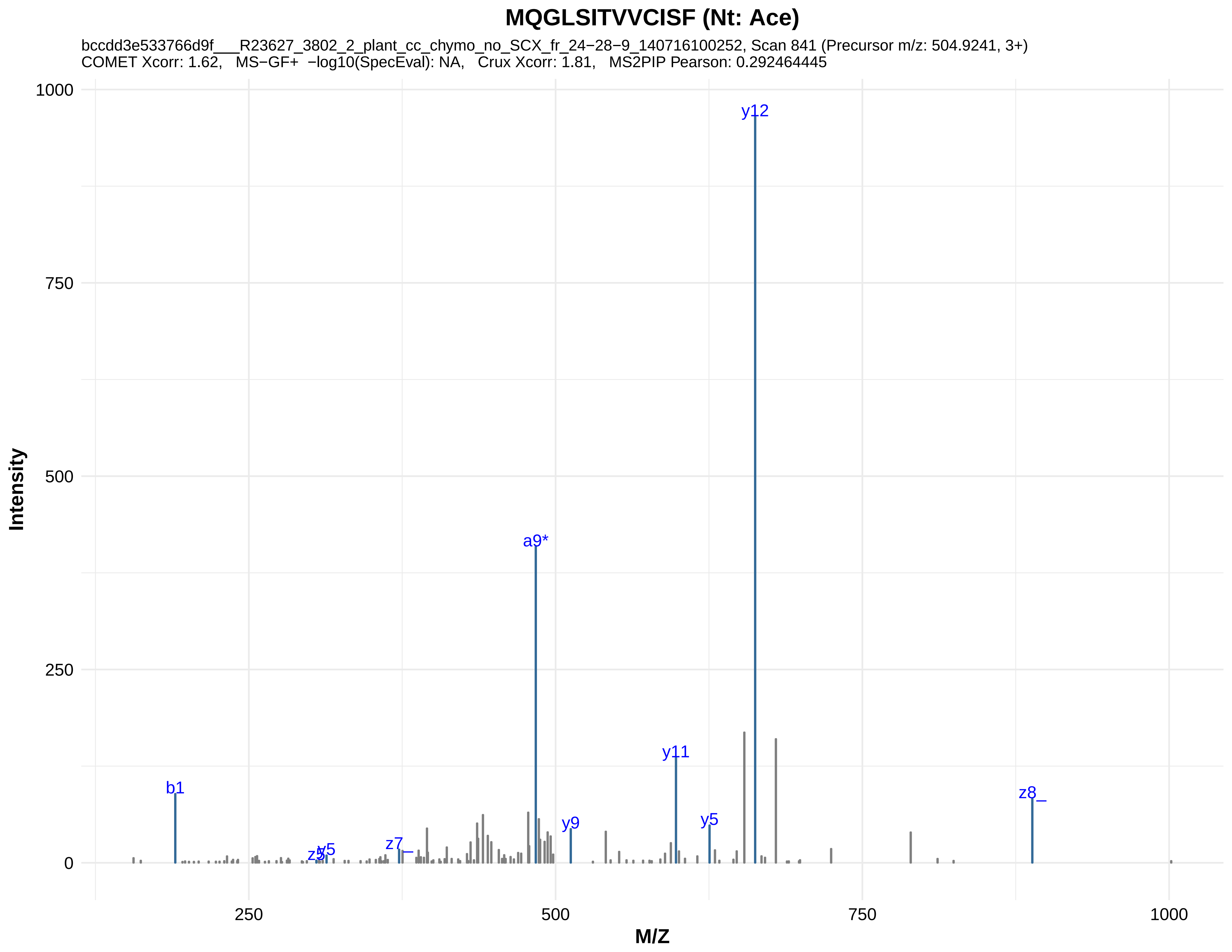

MQGLSITVVCISF (Nt: Ace)
bccdd3e533766d9f___R23627_3802_2_plant_cc_chymo_no_SCX_fr_24−28−9_140716100252, Scan 841 (Precursor m/z: 504.9241, 3+)
COMET Xcorr: 1.62, MS−GF+ −log10(SpecEval): NA, Crux Xcorr: 1.81, MS2PIP Pearson: 0.292464445
Intensity
M/Z
0
250
500
750
1000
y12
a9*
y11
b1
z8_
y9
y5
z7_

MRNENIRKTFENFIND (Nt: Ace)

a9eeb67742df5dfc___R23665_3803_3_plant_cc_GluC_no_SCX_fr_24−28−2, Scan 2503 (Precursor m/z: 716.0175, 3+)
COMET Xcorr: 2.13,   MS−GF+ −log10(SpecEval): NA,   Crux Xcorr: 1.51,   MS2PIP Pearson: 0.225609938

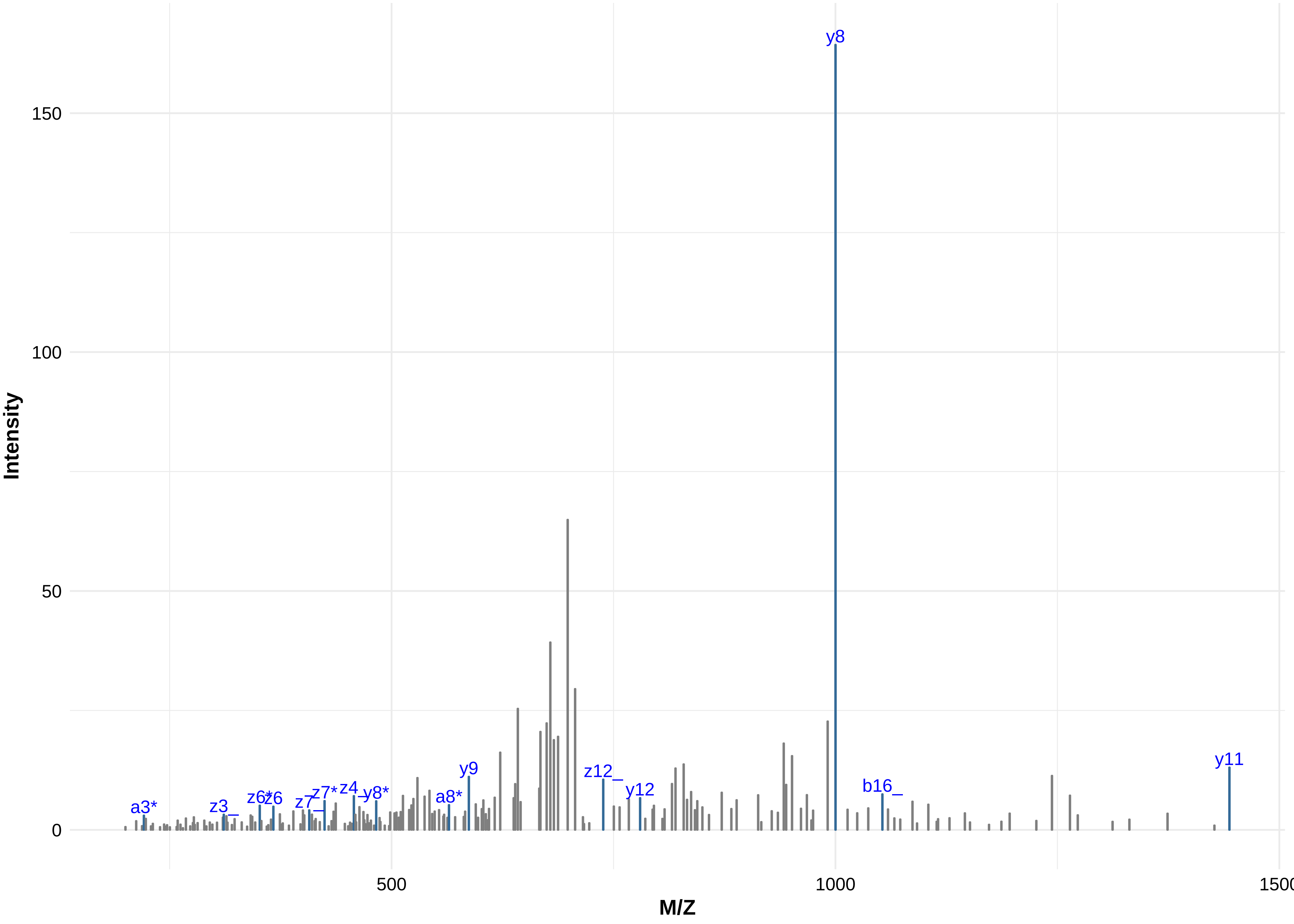

MRQEQKLNKTNCNISSRL (Nt: Ace)

bccdd3e533766d9f___R23623_3802_2_plant_cc_chymo_no_SCX_fr_24−28−5, Scan 1494 (Precursor m/z: 791.4065, 3+)
COMET Xcorr: 2.46,   MS−GF+ −log10(SpecEval): NA,   Crux Xcorr: 2.79,   MS2PIP Pearson: 0.274158273

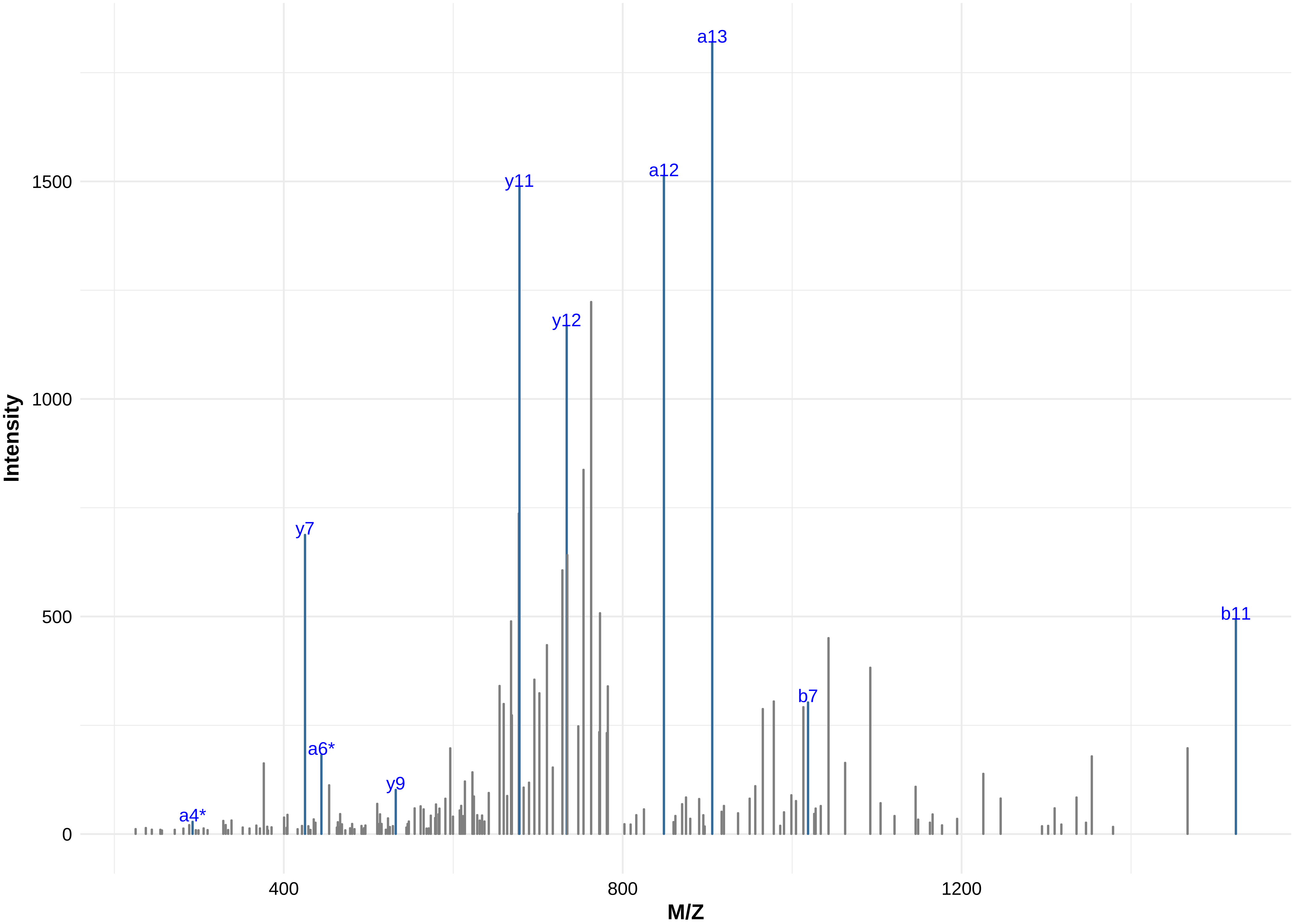

MRRKTNNAESREMW (Nt: Ace)

bccdd3e533766d9f___R23608_3802_2_plant_cc_chymo_no_SCX_fr_28−32−5_140715131822, Scan 300 (Precursor m/z: 483.2258, 4+)
COMET Xcorr: 2.19,   MS−GF+ −log10(SpecEval): NA,   Crux Xcorr: 1.59,   MS2PIP Pearson: −0.429294925

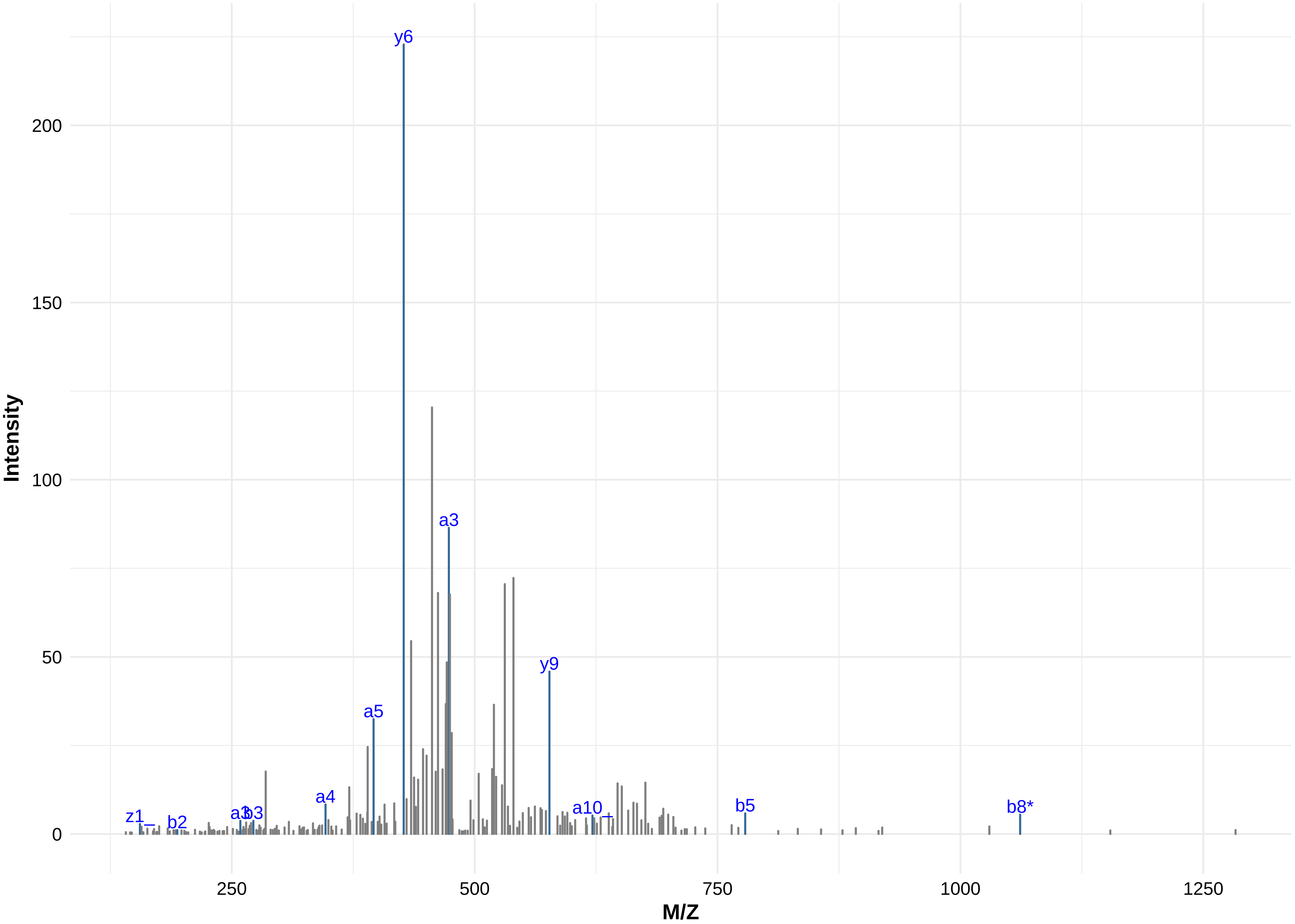

MYEVPLETFSISNLIYWVCDTQLFPR (Nt: Ace)

8ab0e245ad1979ce___R23590_3801_1_plant_cc_tryp_no_SCX_fr_20−24−2, Scan 1118 (Precursor m/z: 1093.86, 3+)
COMET Xcorr: 2.01,   MS−GF+ −log10(SpecEval): 4.66,   Crux Xcorr: 2.46,   MS2PIP Pearson: −0.011121498

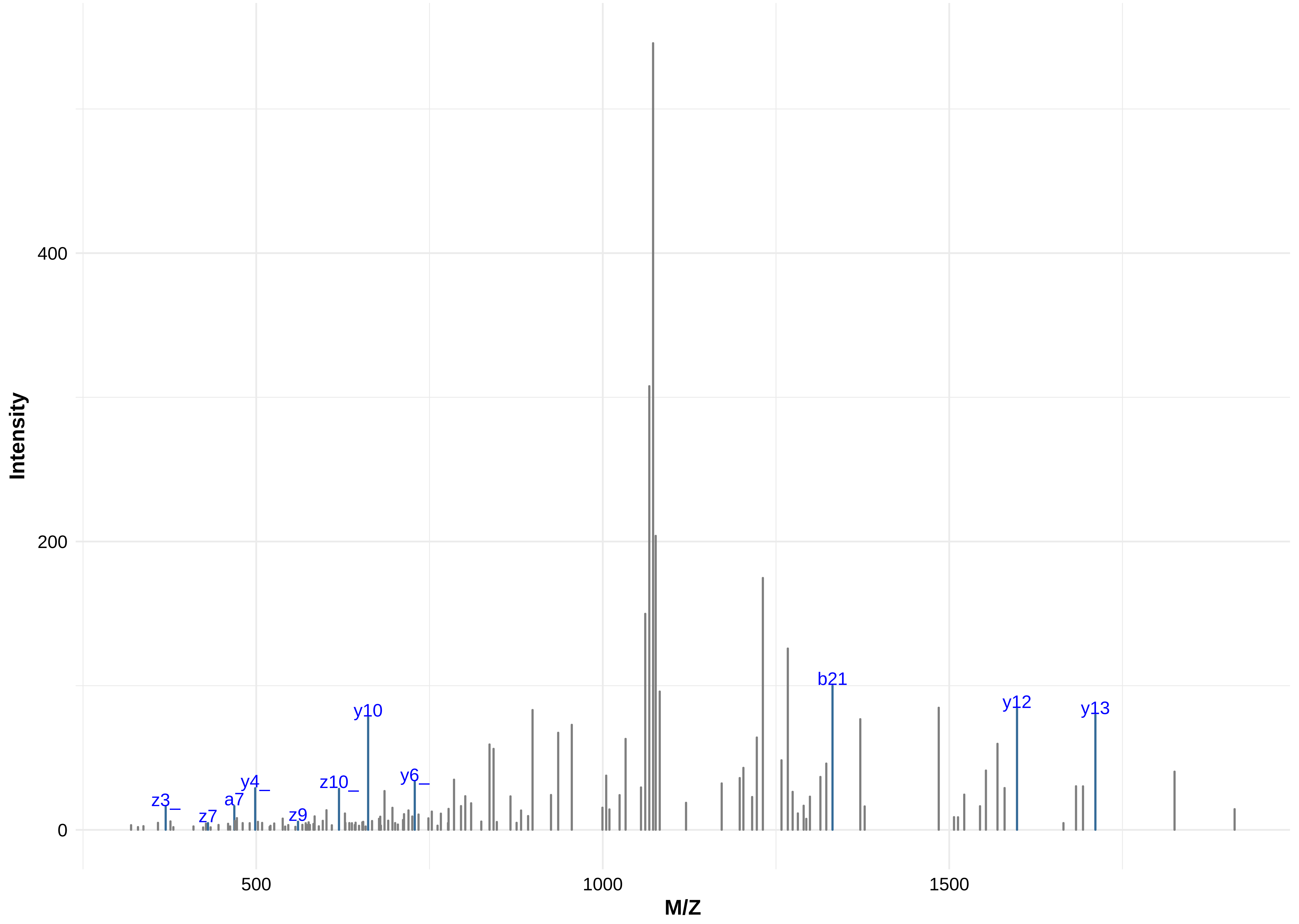

MYIGIVRAM (Nt: Trideutero)

bccdd3e533766d9f___R23640_3802_2_plant_cc_chymo_no_SCX_fr_20−24−7, Scan 252 (Precursor m/z: 543.273, 2+)
COMET Xcorr: 1.78,   MS−GF+ −log10(SpecEval): 7.6,   Crux Xcorr: NA,   MS2PIP Pearson: 0.454821907

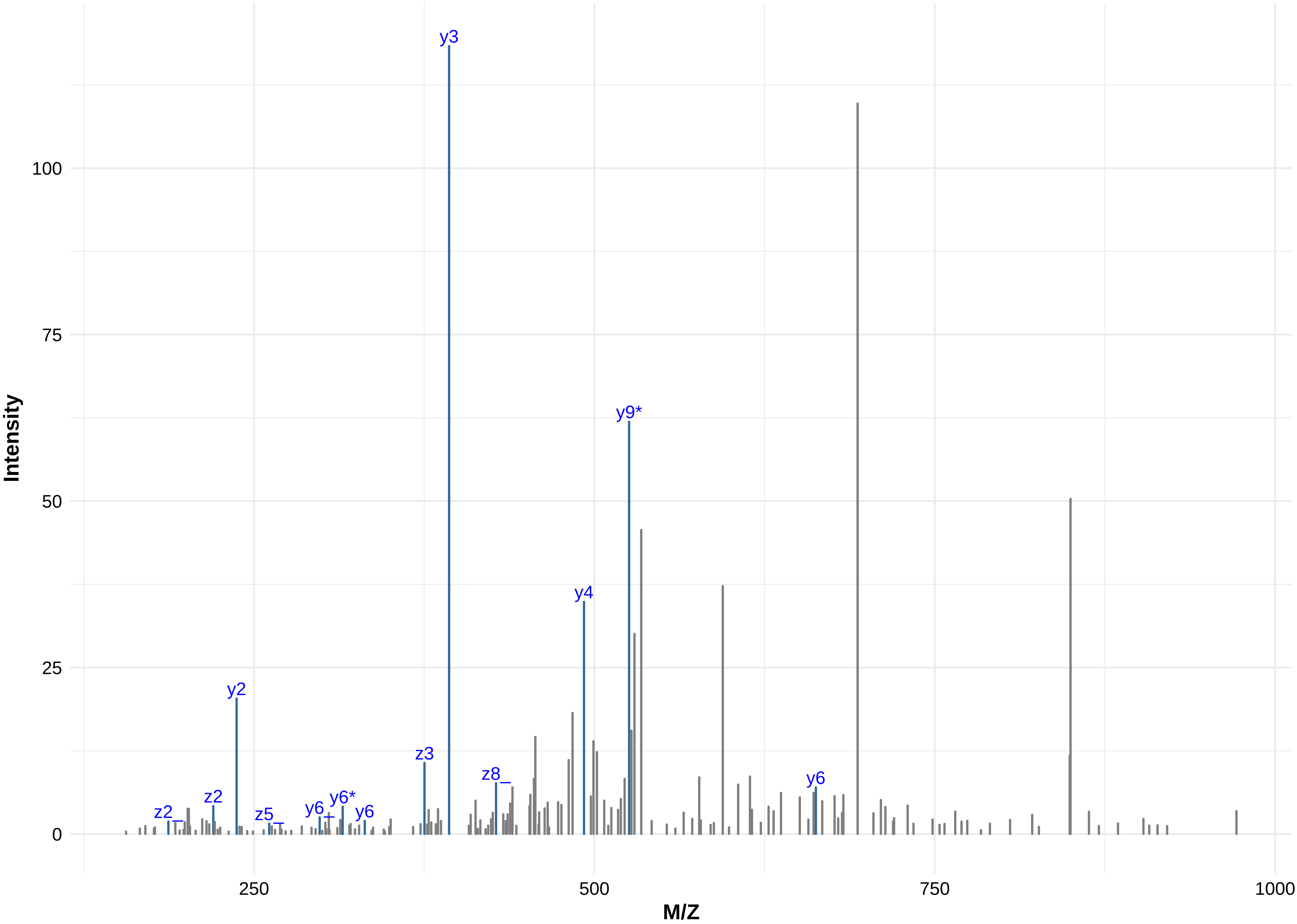

MYISHAEESLR (Nt: Trideutero)

8ab0e245ad1979ce___R23570_3801_1_plant_cc_tryp_no_SCX_fr_28−32−12, Scan 1305 (Precursor m/z: 635.3176, 2+)
COMET Xcorr: 1.82, MS−GF+ −log10(SpecEval): 8.66, Crux Xcorr: 1.53, MS2PIP Pearson: 0.371515055

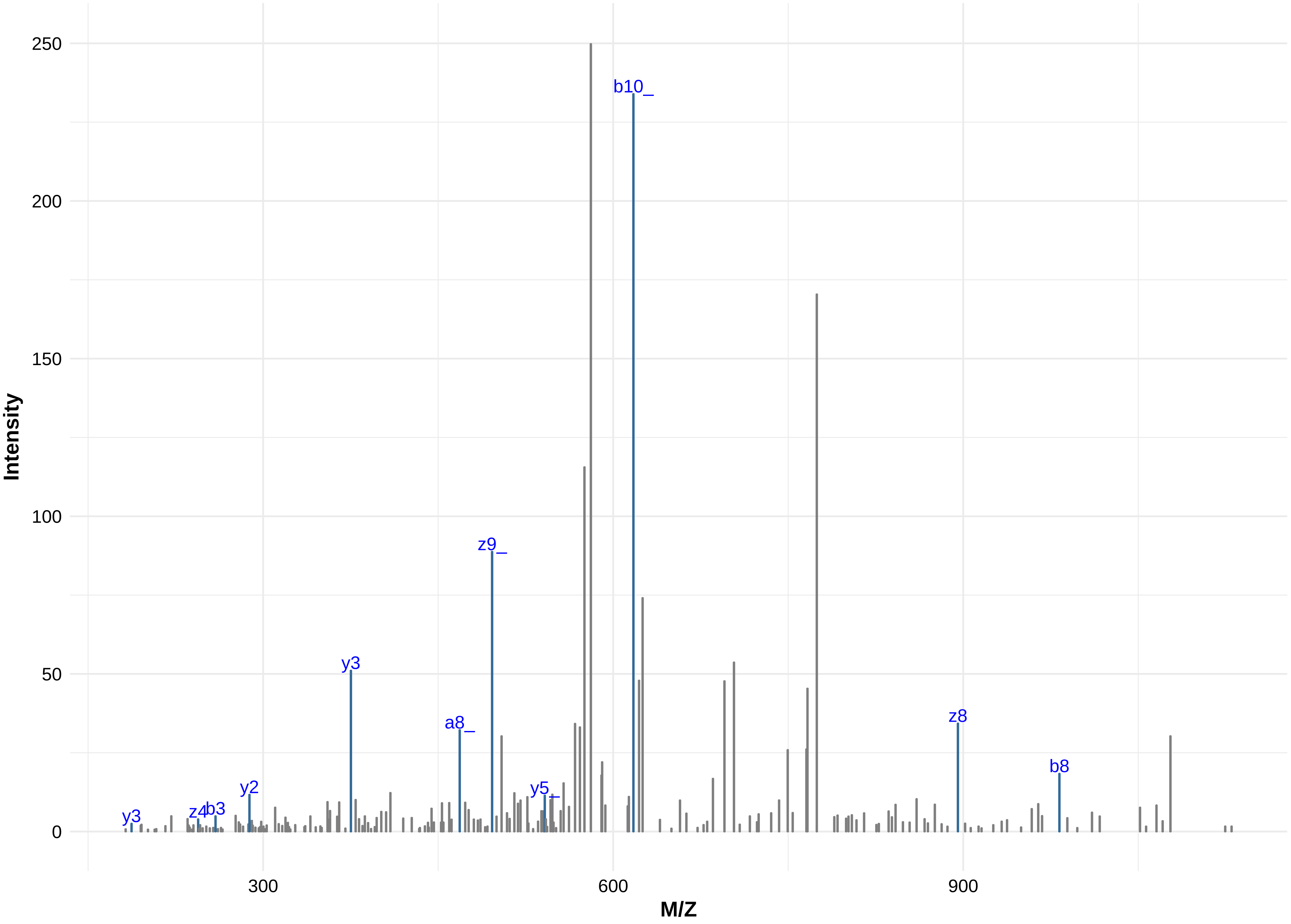

MYQSHETAAAM (Nt: Trideutero)

bccdd3e533766d9f___R23638_3802_2_plant_cc_chymo_no_SCX_fr_20−24−5, Scan 718 (Precursor m/z: 659.773, 2+)
COMET Xcorr: 1.82, MS−GF+ −log10(SpecEval): NA, Crux Xcorr: 1.67, MS2PIP Pearson: 0.606031881

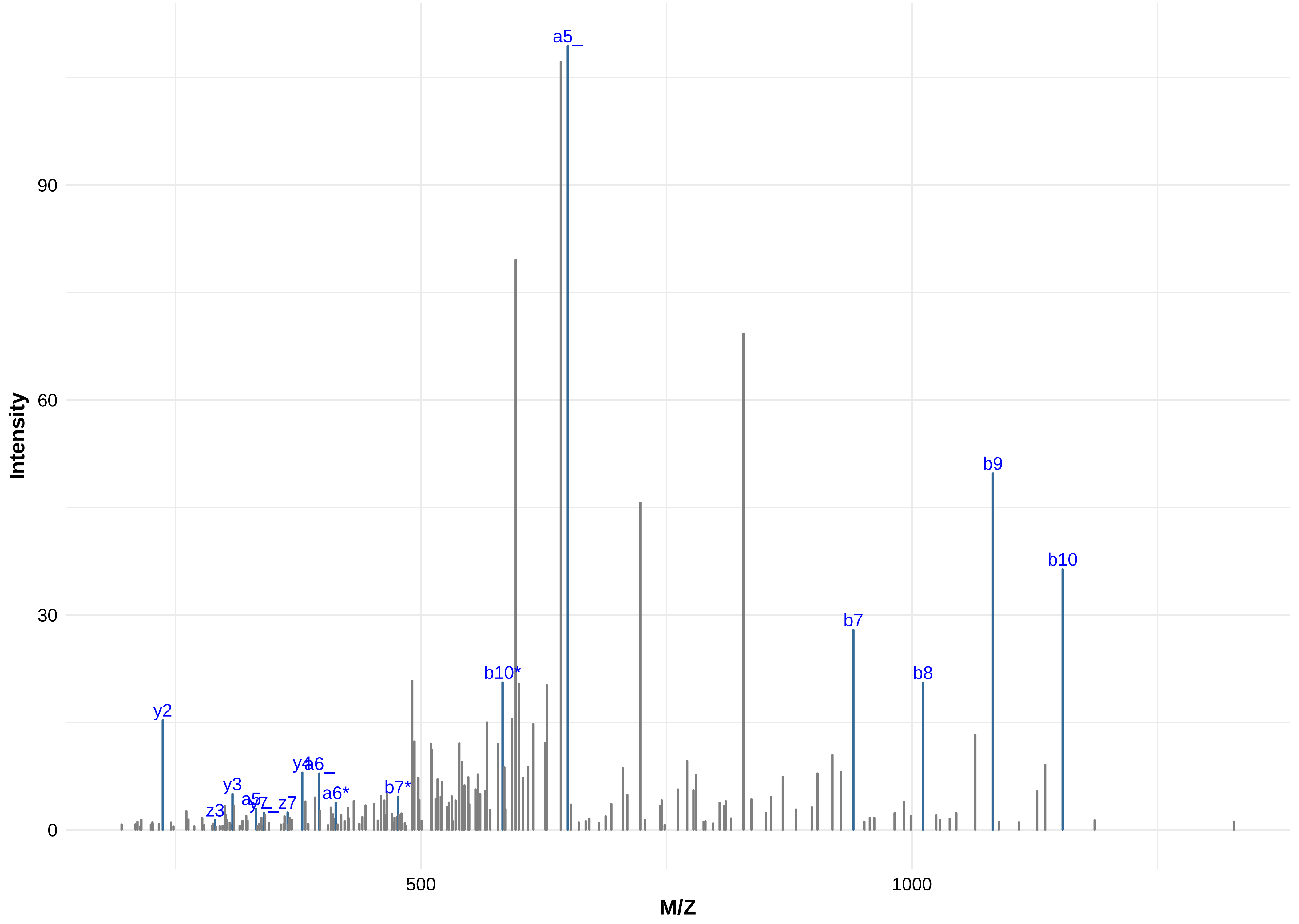

MYTNEISIKSPAL (Nt: Trideutero)

bccdd3e533766d9f___R23641_3802_2_plant_cc_chymo_no_SCX_fr_20−24−8, Scan 227 (Precursor m/z: 526.278, 3+)
COMET Xcorr: 1.8,   MS−GF+ −log10(SpecEval): 5.36,   Crux Xcorr: 2.02,   MS2PIP Pearson: 0.360419172

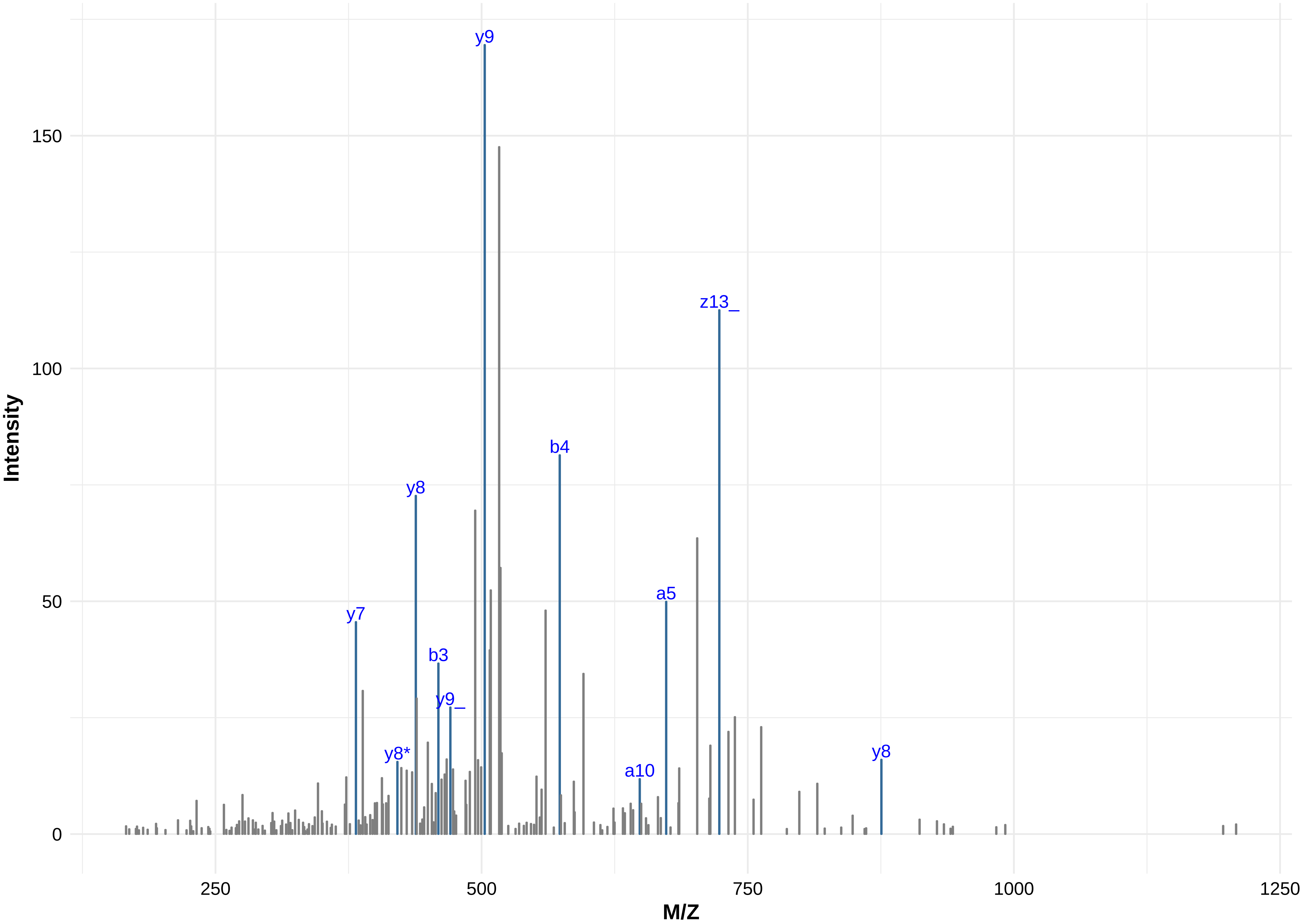

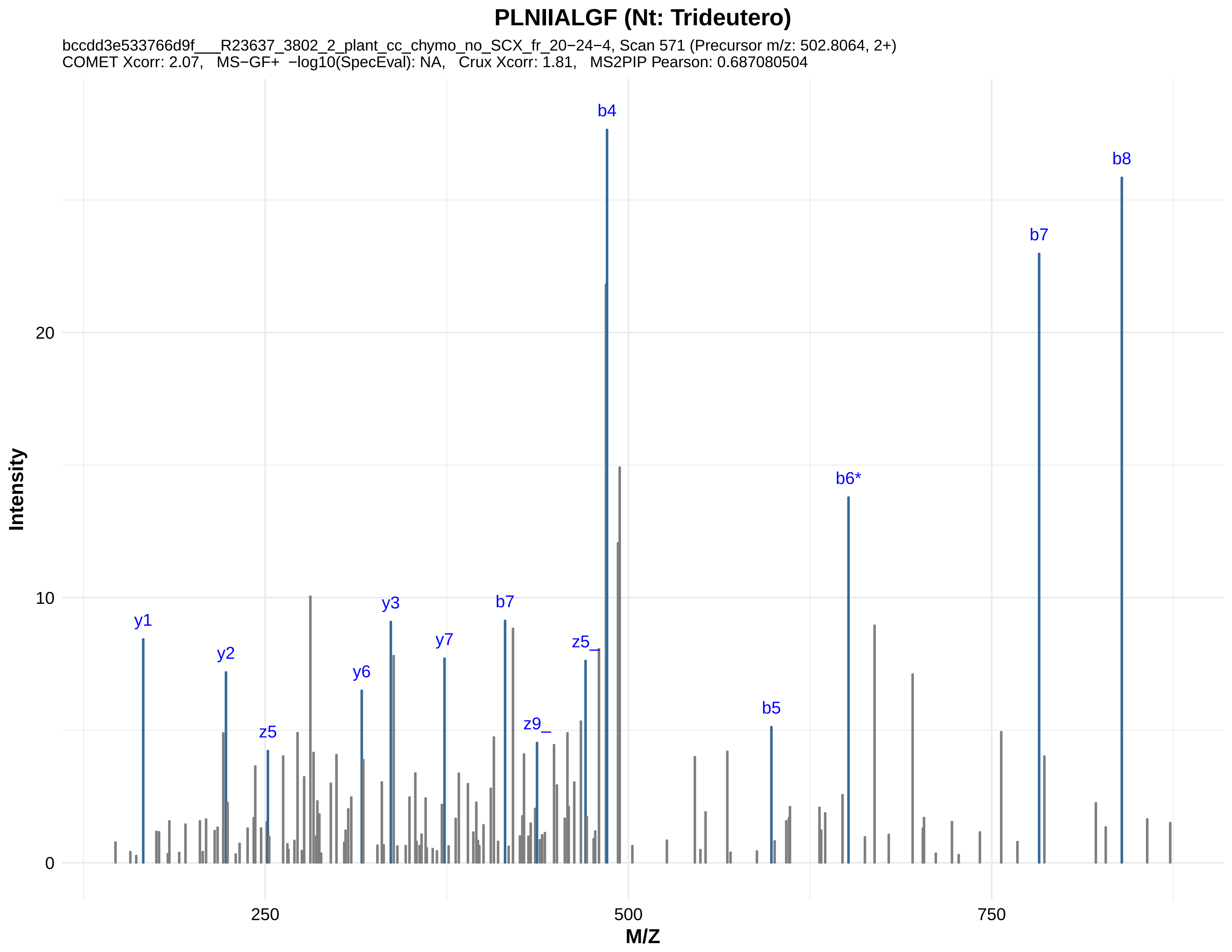
PLNIIALGF (Nt: Trideutero)
bccdd3e533766d9f___R23637_3802_2_plant_cc_chymo_no_SCX_fr_20−24−4, Scan 571 (Precursor m/z: 502.8064, 2+)
COMET Xcorr: 2.07, MS−GF+ −log10(SpecEval): NA, Crux Xcorr: 1.81, MS2PIP Pearson: 0.687080504
Intensity
M/Z
0
10
20
250
500
750
b4
b8
b7
b6*
b5
y1
y2
z5
y6
y3
y7
b7
z9_
z5_

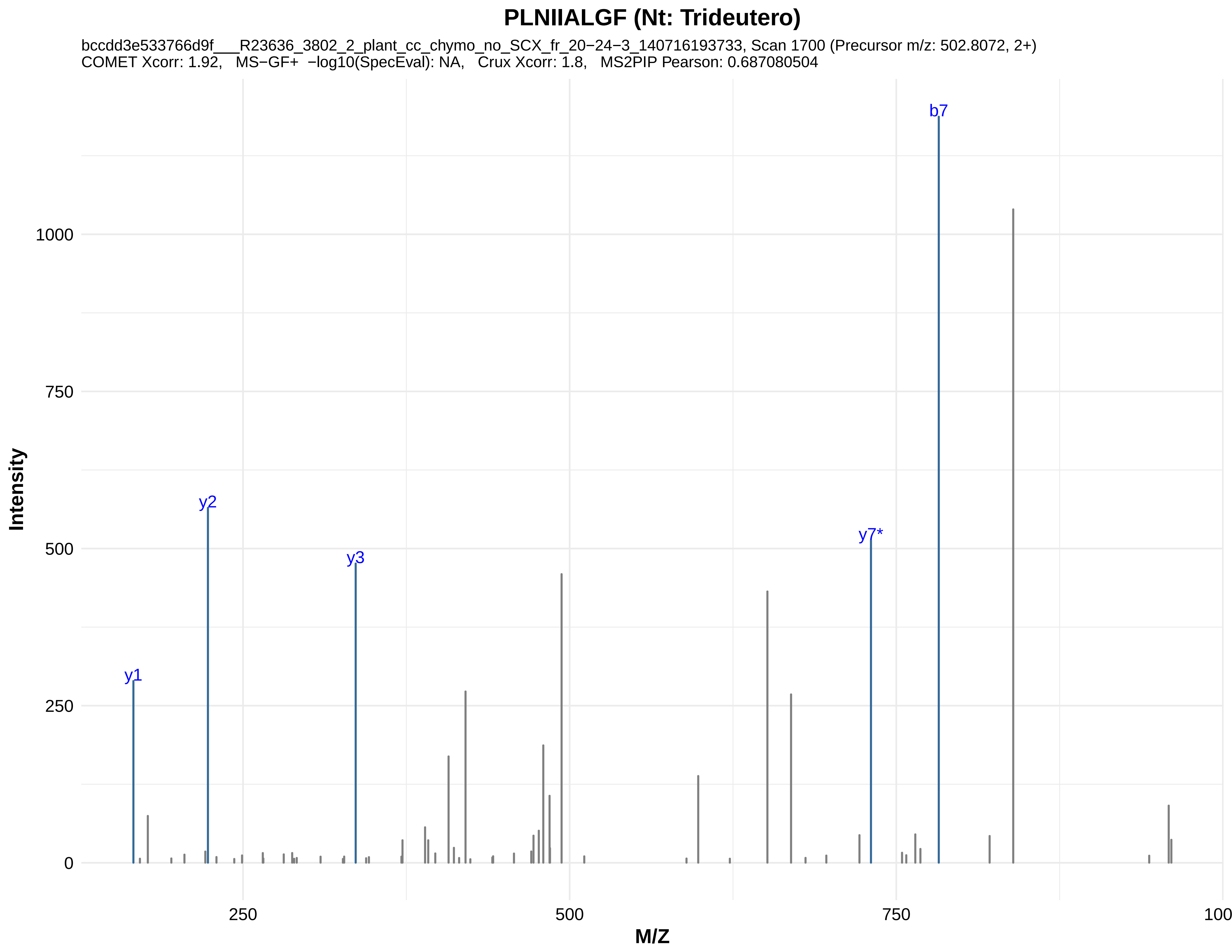
PLNIIALGF (Nt: Trideutero)
bccdd3e533766d9f___R23636_3802_2_plant_cc_chymo_no_SCX_fr_20−24−3_140716193733, Scan 1700 (Precursor m/z: 502.8072, 2+)
COMET Xcorr: 1.92, MS−GF+ −log10(SpecEval): NA, Crux Xcorr: 1.8, MS2PIP Pearson: 0.687080504
Intensity
M/Z
0
250
500
750
1000
250
500
750
1000
y1
y2
y3
y7*
b7

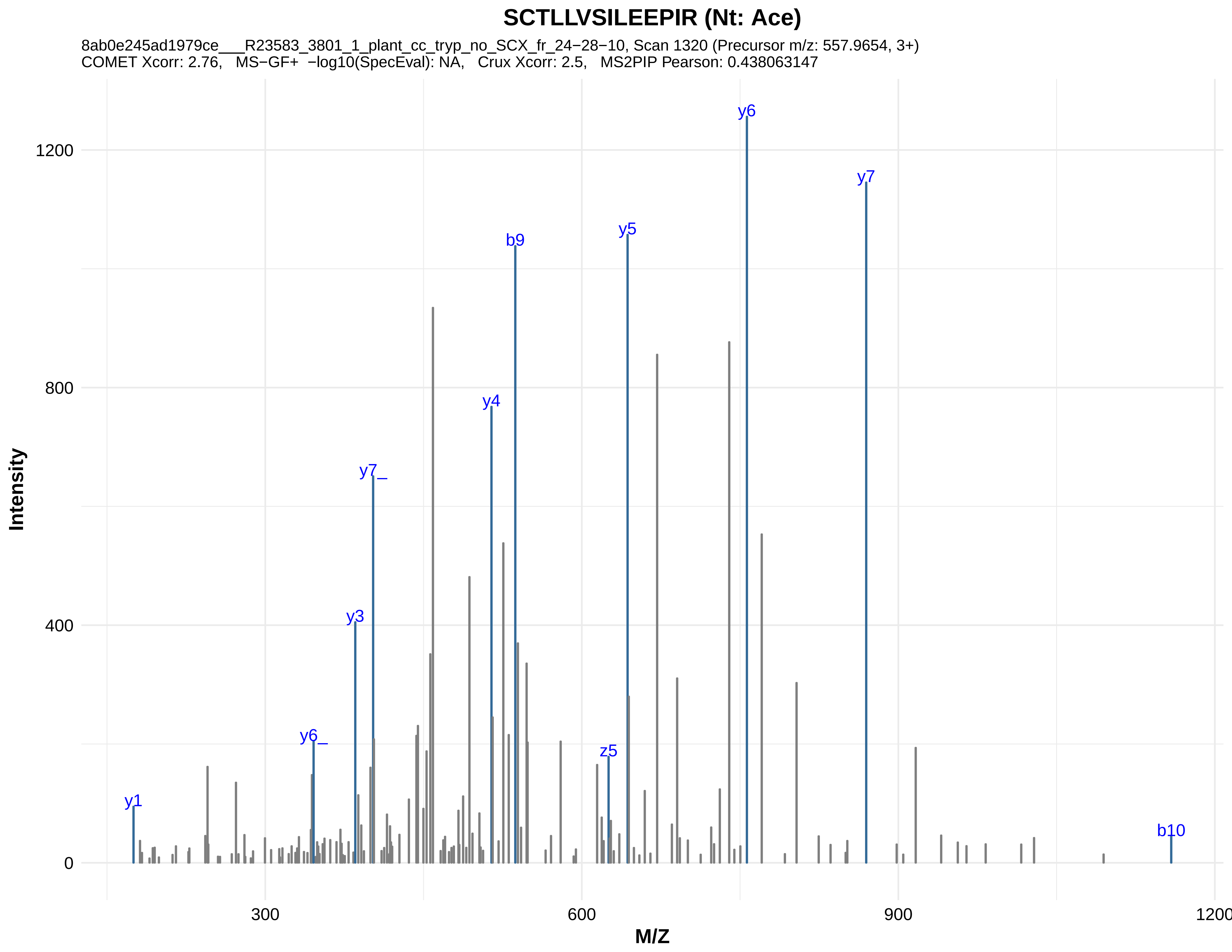
SCTLLVSILEEPIR (Nt: Ace)
8ab0e245ad1979ce___R23583_3801_1_plant_cc_tryp_no_SCX_fr_24−28−10, Scan 1320 (Precursor m/z: 557.9654, 3+)
COMET Xcorr: 2.76, MS−GF+ −log10(SpecEval): NA, Crux Xcorr: 2.5, MS2PIP Pearson: 0.438063147
Intensity
M/Z
y6
y7
y5
b9
y4
y7_
y3
y6_
z5
y1
b10
0
400
800
1200
300
600
900
1200

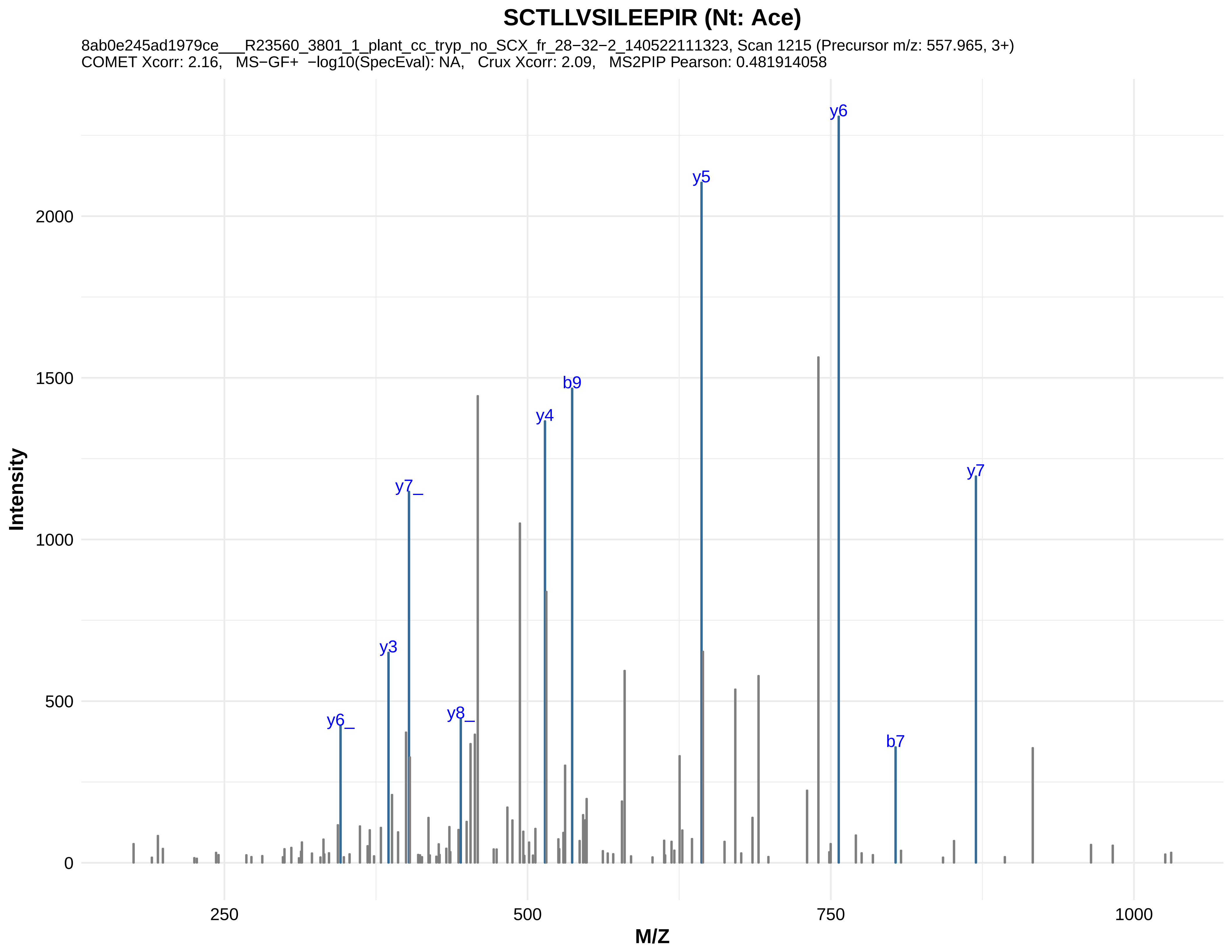
SCTLLVSILEEPIR (Nt: Ace)
8ab0e245ad1979ce___R23560_3801_1_plant_cc_tryp_no_SCX_fr_28−32−2_140522111323, Scan 1215 (Precursor m/z: 557.965, 3+)
COMET Xcorr: 2.16, MS−GF+ −log10(SpecEval): NA, Crux Xcorr: 2.09, MS2PIP Pearson: 0.481914058
Intensity
M/Z
0
500
1000
1500
2000
250
500
750
1000
y6
y5
b9
y4
y7_
y7
y3
y6_
y8_
b7

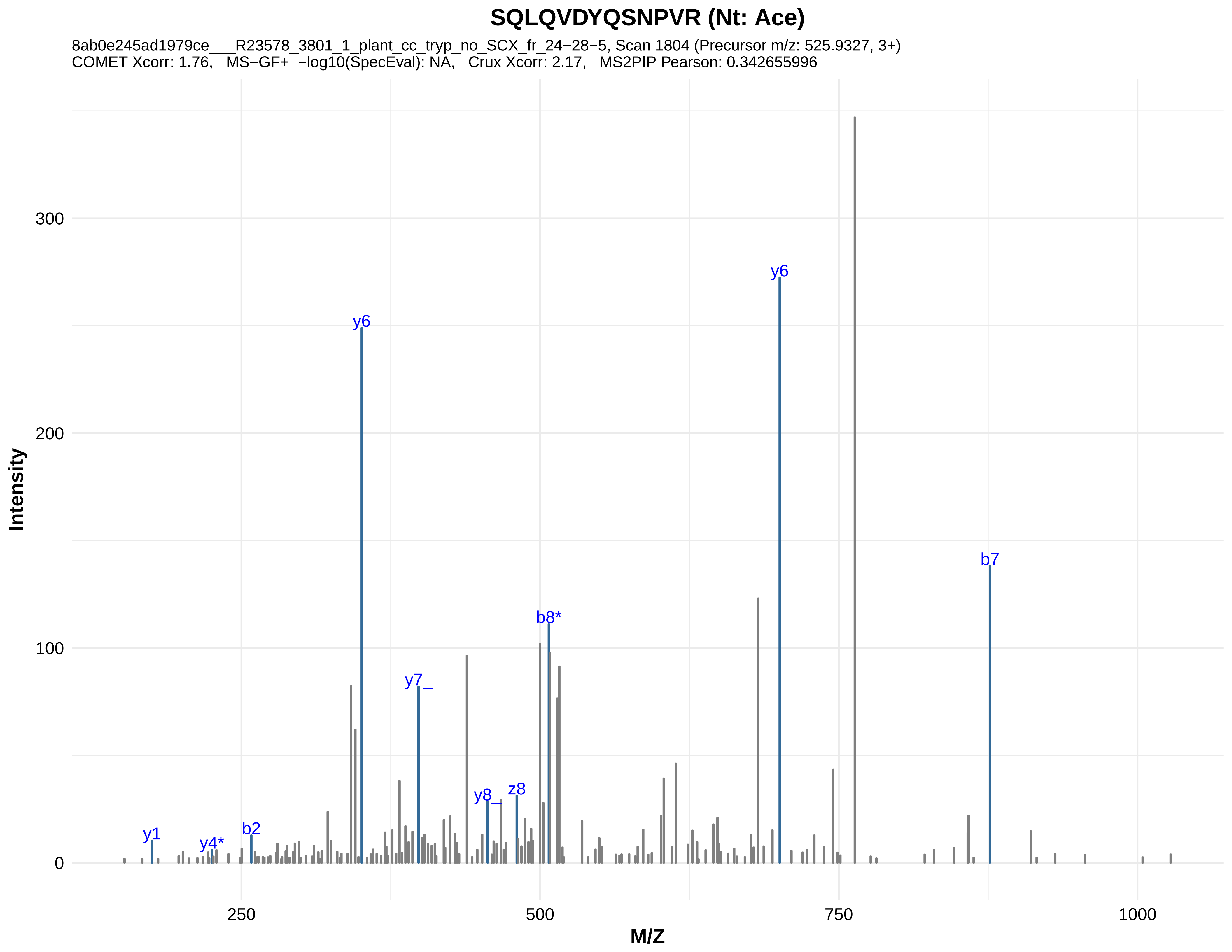
SQLQVDYQSNPVR (Nt: Ace)
8ab0e245ad1979ce___R23578_3801_1_plant_cc_tryp_no_SCX_fr_24−28−5, Scan 1804 (Precursor m/z: 525.9327, 3+)
COMET Xcorr: 1.76, MS−GF+ −log10(SpecEval): NA, Crux Xcorr: 2.17, MS2PIP Pearson: 0.342655996
Intensity
M/Z
y1
y4*
b2
y6
y7_
y8_
z8
b8*
y6
b7
0
100
200
300
250
500
750
1000

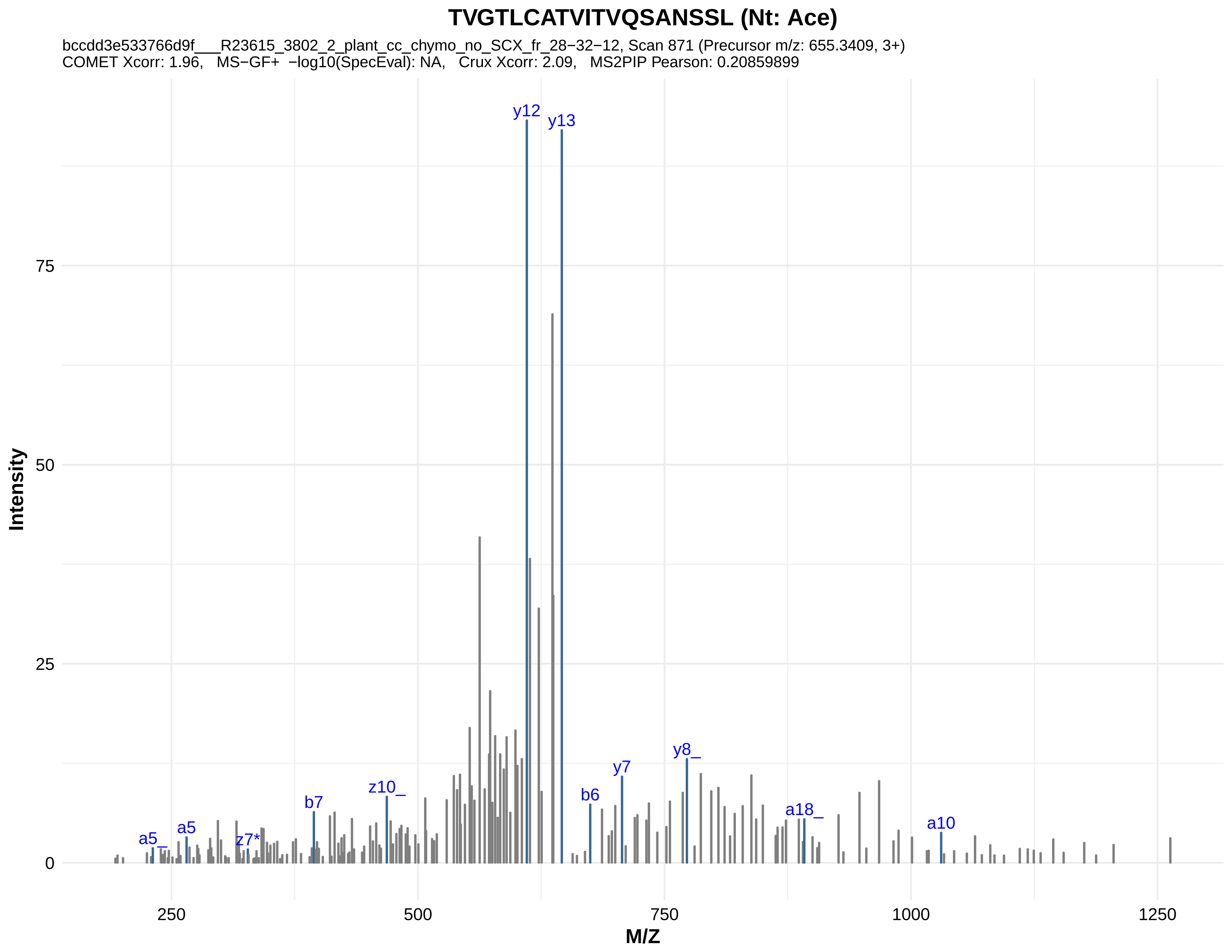
TVGTLCATVITVQSANSSL (Nt: Ace)
bccdd3e533766d9f___R23615_3802_2_plant_cc_chymo_no_SCX_fr_28−32−12, Scan 871 (Precursor m/z: 655.3409, 3+)
COMET Xcorr: 1.96, MS−GF+ −log10(SpecEval): NA, Crux Xcorr: 2.09, MS2PIP Pearson: 0.20859899
Intensity
M/Z
0
25
50
75
250
500
750
1000
1250
y12
y13
a5_
a5
z7*
b7
z10_
b6
y7
y8_
a18_
a10

VCFNLIVVVSVKHLGFI (Nt: Trideutero)

0fdf8708e3b3bf53___R23701_3805_4_plant_cc_AspN_no_SCX_fr_28−32−8, Scan 2560 (Precursor m/z: 680.0654, 3+)
COMET Xcorr: 2,   MS−GF+ −log10(SpecEval): NA,   Crux Xcorr: 1.51,   MS2PIP Pearson: 0.14202493

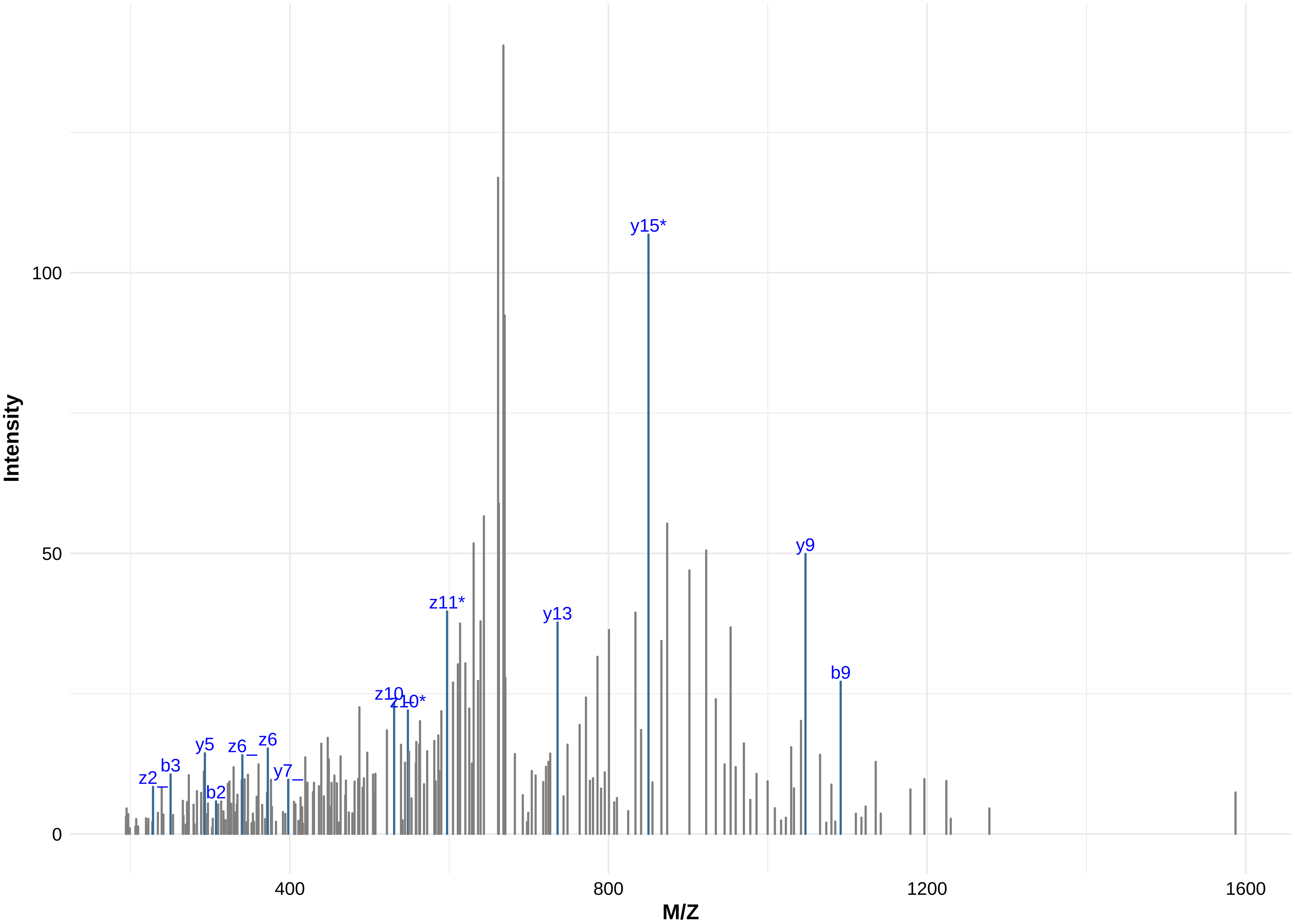

Supplement: Supplemental Data [file 10.1074_M116.066662_mcp.M116.066662-3.pdf]
